# Thermal Truncation of Heptamethine Cyanine Dyes

**DOI:** 10.1021/jacs.4c02116

**Published:** 2024-07-12

**Authors:** Jana Okoročenkova, Josef Filgas, Nasrulla Majid Khan, Petr Slavíček, Petr Klán

**Affiliations:** † Department of Chemistry, Faculty of Science, Masaryk University, Kamenice 5, 625 00 Brno, Czech Republic; ‡ RECETOX, Faculty of Science, Masaryk University, Kamenice 5, 625 00 Brno, Czech Republic; § Department of Physical Chemistry, University of Chemistry and Technology, Technická 5, 16628 Prague 6, Czech Republic

## Abstract

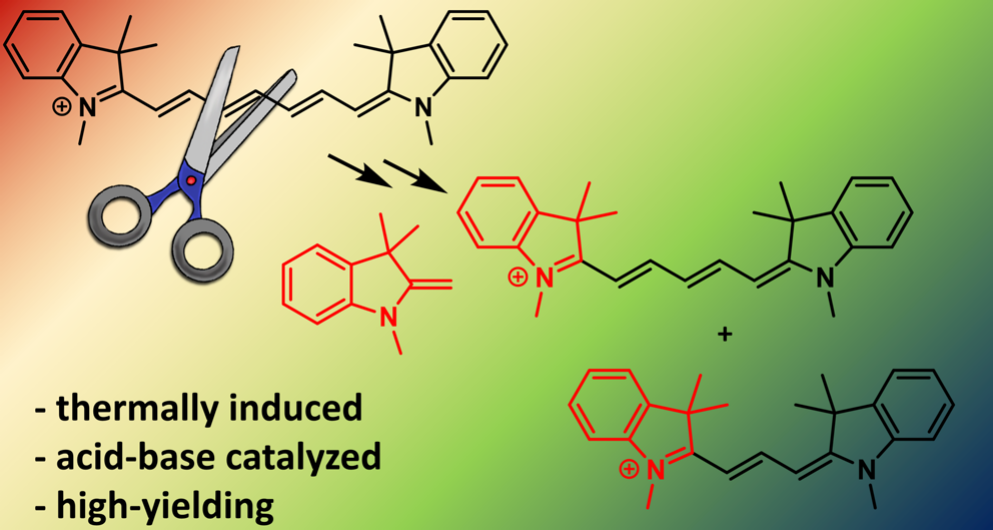

Cyanine dyes are a class of organic, usually cationic molecules containing two nitrogen centers linked through conjugated polymethine chains. The synthesis and reactivity of cyanine derivatives have been extensively investigated for decades. Unlike the recently described phototruncation process, the thermal truncation (chain shortening) reaction is a phenomenon that has rarely been reported for these important fluorophores. Here, we present a systematic investigation of the truncation of heptamethine cyanines (Cy7) to pentamethine (Cy5) and trimethine (Cy3) cyanines via homogeneous, acid–base-catalyzed nucleophilic exchange reactions. We demonstrate how different substituents at the C3′ and C4′ positions of the chain and different heterocyclic end groups, the presence of bases, nucleophiles, and oxygen, solvent properties, and temperature affect the truncation process. The mechanism of chain shortening, studied by various analytical and spectroscopic techniques, was verified by extensive *ab initio* calculation, implying the necessity to model catalytic reactions by highly correlated wave function-based methods. In this study, we provide critical insight into the reactivity of cyanine polyene chains and elucidate the truncation mechanism and methods to mitigate side processes that can occur during the synthesis of cyanine derivatives. In addition, we offer alternative routes to the preparation of symmetrical and unsymmetrical *meso*-substituted Cy5 derivatives.

## Introduction

Cyanine dyes have been found to be widely used as fluorescent probes for labeling nucleic acids and proteins and as photosensitizers in photodynamic therapy, biosensors, and imaging agents.^1−4^ For example, near-infrared fluorescent pentamethine (Cy5) and heptamethine (Cy7) cyanine dyes have shown promise as a tool for cancer imaging and targeted therapy.^5,6^ Changing the structures and thus physicochemical properties of Cy5 derivatives affect biodistribution, allowing tissue-specific targeting.^7,8^ Synthetic strategies toward the modification of cyanines generally rely on an early-stage introduction of various functional groups into the heterocyclic terminal groups or the heptamethine chain.^9−12^

**Scheme 1:**
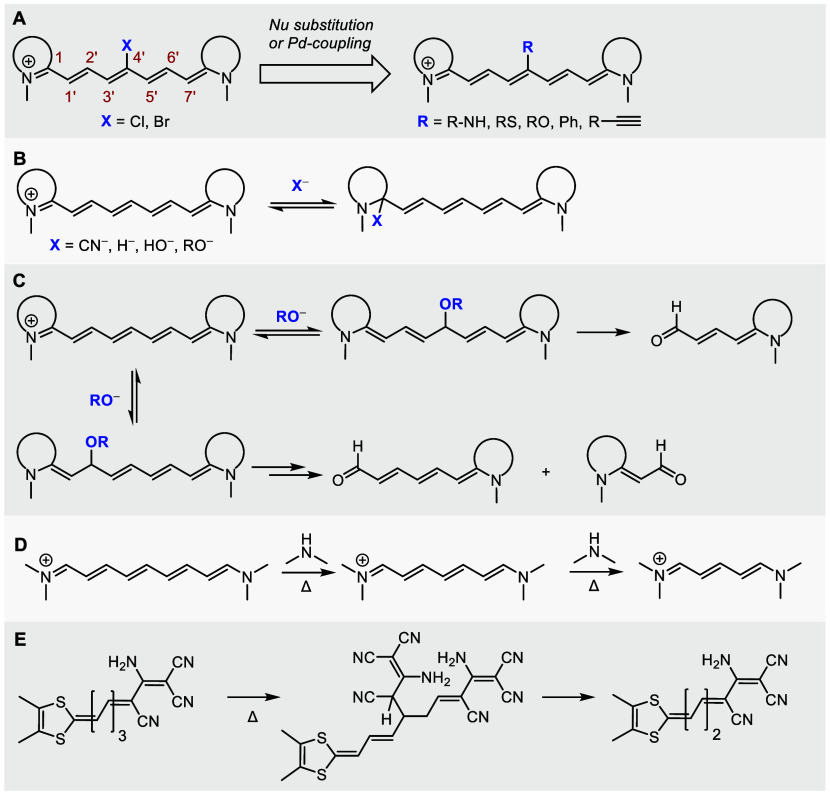
Reported Reactions of Polymethine Chains with Nucleophiles: (A) Nucleophilic Substitution of Halogen Atoms or Pd-Coupling Reactions, (B) Reversible Addition of Nucleophiles to the Iminium C1 Atom, (C) Addition of Methoxide to the C2′ or C4′ Chain Positions with Subsequent Fragmentation, (D) Aminolysis of the Polymethine Chain Resulting in Truncation, (E) Truncation of Merocyanines in the Presence of a Nucleophile

Further synthetic modifications of cyanines, especially on their polyene chains, are a poorly explored area. Common strategies in Cy7 dyes involve the modification of the chain *meso*-position by nucleophilic substitution of the C4′-chloro substituents by N, S, and O nucleophiles via an S_RN_1 reaction^13−16^ or Pd-catalyzed Suzuki^17^ or Sonogashira^10^ coupling reactions (Scheme 1A). Due to a positive charge delocalization along the conjugated π-system, hydroxide,^18^ alkoxide,^18^ cyano,^19−21^ and hydride^22^ ions can reversibly attack the iminium C1 atom (Scheme 1B). The latter two processes have been utilized in the biosensing of cyanide anions^19−21^ and reactive oxygen species (ROS).^22^ Few studies reported the addition of hydroxide or alkoxide to the alternating polyene double bonds,^18,23^ such as the methoxide addition to the C2′ or C4′ chain position of Cy7 or Cy5, followed by spontaneous oxidative fragmentation to aldehydes in aerated solutions (Scheme 1C).^23^ It was also demonstrated that the aminolysis of nonamethinecyanines (Cy9) by secondary amines leads to the chain shortening (truncation) to give cyanines Cy7 that subsequently degrade to Cy5 derivatives when treated with amines for a longer time (Scheme 1D).^24^ Similarly, unexpected chain shortening during the preparation of merocyanine dyes was observed via the attack of a *C*-nucleophile (Scheme 1E).^25,26^ The polyene chain shortening products were also occasionally observed as side products during the synthesis of polymethine derivatives;^27,28^ however, the reactions have never been systematically studied.

In conjunction with applications of cyanine dyes in optical imaging and photochemical drug delivery,^2,29−31^ Schnermann and co-workers described that excitation of Cy7 can also lead to the formation of truncated cyanine derivatives (Cy5), involving a singlet oxygen-initiated multistep process.^32−34^ Because the absorption maxima of truncated products are hypsochromically shifted,^35,36^ the phototruncation reaction is termed photoblueing. The photoconversion of Cy5 to Cy3 has also been reported.^37^

**Scheme 2:**
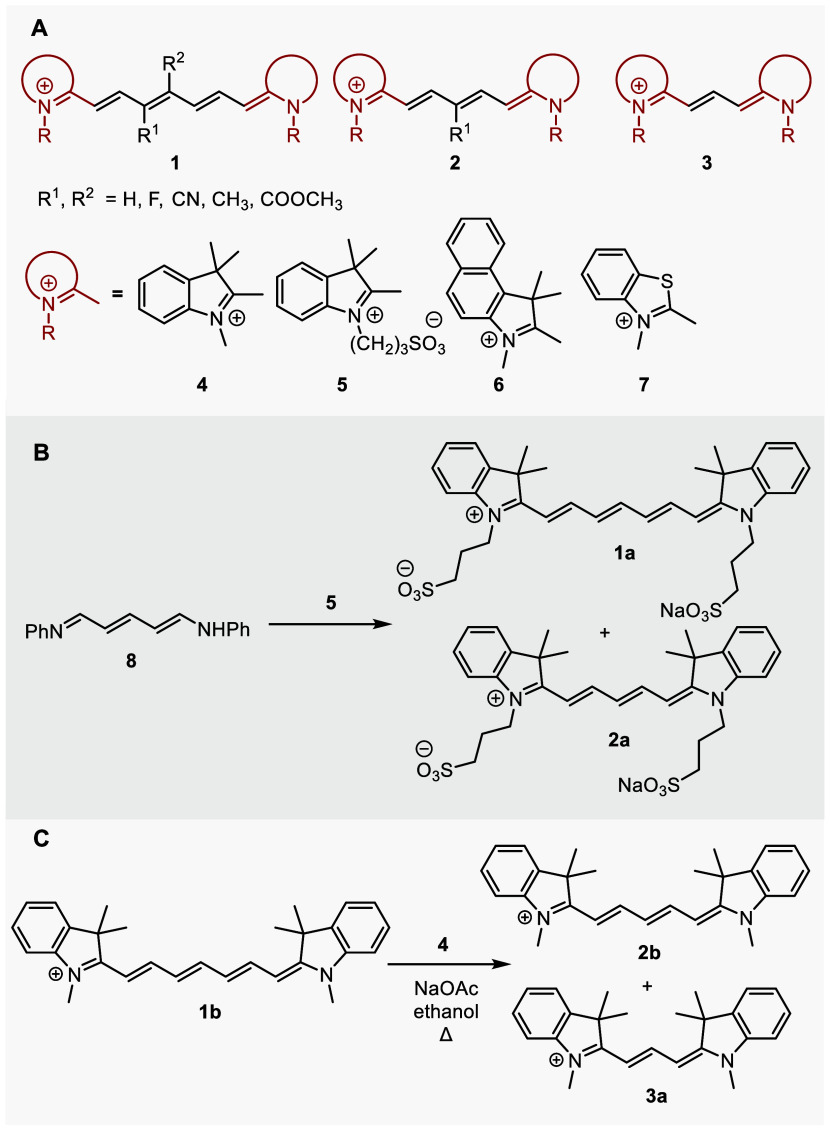
(A) Cyanines Used in This Study, (B) Unexpected Formation of the Cy5 Product **2a** Observed during the Synthesis of **1a**, (C) Formation of Cy5 and Cy3 Products during Heating of Heptamethine Cyanine **1b** in the Presence of Indolinium Salt **4** and Sodium Acetate

As a part of our initial investigations of cyanine dyes (general structures **1**–**3** refer to heptamethine, pentamethine, and trimethine cyanines, respectively, that bear diverse heterocyclic ends **4**–**7**),^11^ we initially aimed to prepare a series of chain-substituted Cy7s to examine their physicochemical properties (Scheme 2A). However, during the preparation of **1a** from [5-(phenylimino)penta-1,3-dien-1-yl]aniline (**8**) and indolinium salt **5** in the presence of sodium acetate in ethanol,^38−40^ we noticed the formation of pentamethine cyanine **2a** with a truncated methine chain as an unexpected side product (Scheme 2B and Figures S4–S6 and S55). Truncated polyene products were also obtained by heating Cy7 **1b** in the presence of indolinium salt **4** and sodium acetate in ethanol (Scheme 2C and Figures S7 and S8). These results encouraged us to explore the thermal chain shortening reactions of the cyanines. We studied how different substituents at the C3′ and C4′ chain positions, different end heterocycles, the presence of bases, nucleophiles, and oxygen, solvent properties, and temperature affect the truncation process of Cy7 dyes. UV–vis spectroscopy, high-performance liquid chromatography (HPLC), high-resolution mass spectrometry (HRMS), and nuclear magnetic resonance (NMR) analyses were used to develop an overall mechanistic picture. This reaction served as a prime example of a complex reaction facilitated by homogeneous catalysis, the mechanism of which was supported by our *ab initio* calculations. As the reliability of these calculations in the field of homogeneous catalysis has recently become the subject of wide debate:^41,42^ here we demonstrate the feasibility of performing highly accurate coupled-cluster calculations, even for reactions as complex as this one.

The primary objective of our work was to elucidate the truncation mechanism and methods to mitigate side processes during the synthesis of cyanines. Nevertheless, we showcase that this process can serve as an alternative method for their preparation and that Cy5 derivatives can be efficiently transformed into other Cy5 derivatives through a heterocyclic exchange process, yielding both symmetrical and unsymmetrical structures.

Coincidentally, we have become aware of an excellent work by the team of Babak Borhan and James E. Jackson (Michigan State University), which describes a very similar discovery of cyanine thermal truncation and is being submitted to this journal for review at the same time as our paper.

## Results and Discussion

### Effects of Base, Solvent, Temperature, and Stoichiometry

To evaluate the scope of the truncation reaction, we prepared heptamethine cyanine **1c** (Table 1) on a multigram scale from 3-fluoropyridinium salt and indolinium iodide according to the previously published procedures,^11,12^ which was chosen because it bears an unsymmetrically placed C3′-fluorine atom of the heptamethine chain. It allowed us to identify which part of the chain remains in the truncated product(s). The effects of a solvent (polar protic ethanol or methanol vs aprotic acetonitrile), base, and temperature on the reaction of **1c** heated with indolinium iodide **4A**, that is, the acid form of **4B** (conjugate base), are summarized in Table 1. Cy7s generally showed better solubility in acetonitrile than in methanol; bases such as NaOAc, *t*-BuOK, or MeONa were soluble only in ethanol. The reaction mixture was stirred continuously and heated at 50 or 80 °C for 21 h. Careful analyses of the reaction mixture (HPLC, HRMS, NMR) revealed that 3′-fluoropentamethine cyanine **2c** is always the major product, whereas Cy3 (**3a**; <2%) and nonsubstituted Cy5 (**2b**; <2%) were formed in negligible amounts.

**Table 1:**
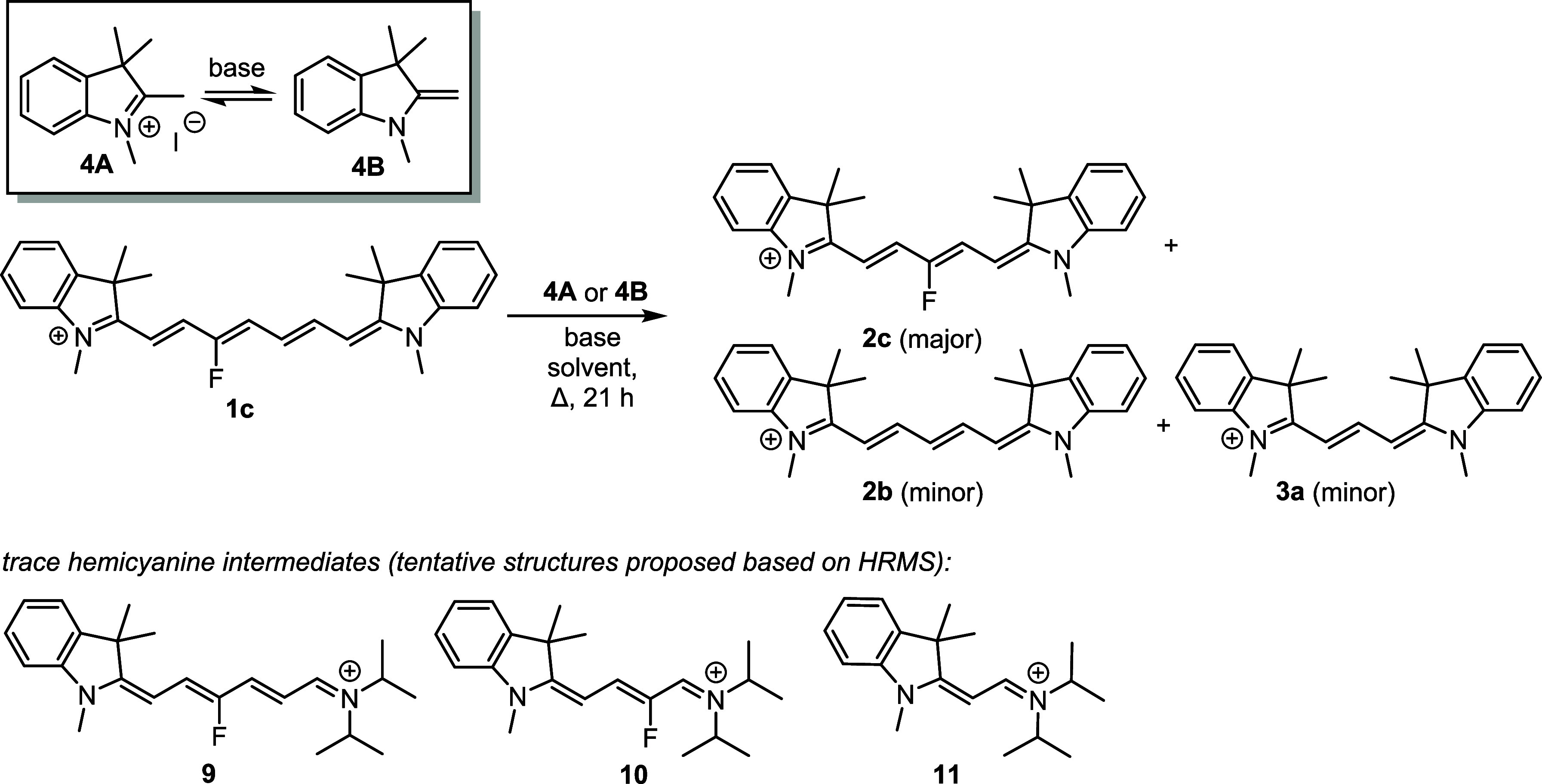
Effects of a Base, Solvent, Temperature, Oxygen, and Water^a^

solvent	*T*/°C	**4A** or **4B** (equiv)	base (equiv)	conversion of **1c**/%	yield of **2c**/%^b^	yield of **3a**/%
EtOH	80	**4A** (2.5)	DBU (5)	75	5	1
EtOH	80	**4A** (2.5)	NaOAc (5)	>99	22	1
EtOH	80	**4A** (2.5)	DIPEA (5)	>99	28	2
EtOH	80	**4A** (2.5)	DIPA (5)	>99	30	2
ACN	80	**4A** (2.5)	DIPA (5)	>99	33	2
ACN	50	**4A** (2.5)	piperidine (5)	98	37	1
ACN	50	**4A** (2.5)	DIPA (5)	96	38	1
ACN	20	**4A** (2.5)	DIPA (5)	45	10	2
ACN	50	**4A** (2.5)	−	18	0	0
ACN	50	**4A** (1)	DIPA (1)	30	6	1
ACN^c^	50	**4A** (1)	DIPA (2.5)	96	40	2
ACN^d^	50	**4A** (1)	DIPA (2.5)	97	48	2
ACN	50	**4A** (1)	DIPA (5)	93	40	1
ACN	50	**4A** (5)	DIPA (5)	93	24	1
ACN	80	−	DIPA (5)	>99	8	0
ACN	80	−	DIPEA (5)	>99	7	0
ACN	50	**4B** (1)	−	51	6	2
ACN	80	**4B** (10)	−	95	31	5
ACN	50	**4B** (1)	DIPA (2.5)	89	38	1
MeOH	50	**4A** (1)	*t-*BuOK (2.5)	72	8	1
MeOH	50	**4A** (1)	MeONa (2.5)	40	4	1
MeOH	50	**4A** (1)	pyridine (2.5)	<1	0	0

a [**1c**] = 72 mM, reaction time = 21 h, iodide as a counteranion is omitted for clarity; ACN = acetonitrile. The standard deviation from 5 independent measurements was below 2%.

b HPLC yields (determined using authentic standards; calculated assuming 100% conversion of **1c**).

c Degassed with a freeze–pump–thaw method.

d Dry (anhydrous) acetonitrile, freshly distilled DIPA; degassed with a freeze–pump–thaw method in a glovebox.

Because the sum of chemical yields at complete conversion was always below 50%, we concluded that other degradation pathways were responsible for the mass loss. Indeed, trace amounts of hemicyanines **9, 10**, and **11**, and the adducts of diisopropylamine (DIPA) and cyanine subunits detected in acetonitrile under different reaction conditions, whose structures were proposed from the HRMS data (Figure S12), represent some possible reaction intermediates or side products. The stoichiometric amounts of a base, such as sodium acetate, *N*,*N*-diisopropylethylamine (DIPEA), or DIPA, and a higher temperature (≥50 °C) were found to be indispensable for high truncation yields (up to ∼50% of **2c** in both solvents). In contrast, the addition of 1,8-diazabicycloundec-7-ene (DBU) as a non-nucleophilic base provided very low **2c** yields despite a high reaction conversion. Using strong bases, such as *t*-BuOK and MeONa, significantly decreased the **2c** yields. Pyridine as a weak base (p*K*_a_ = 5.2) did not mediate any reaction. This suggested that the initial formation of 1,3,3-trimethyl-2-methyleneindoline **4B** (Fischer’s base) is crucial for the reaction; the p*K*_a_ of **4A** (conjugate acid) in water was estimated to be ∼8.5 (see the Supporting Information). The [**2c**]/[**2b**] concentration ratio in acetonitrile decreased from 99:1 at 50 °C to 10:1 at 80 °C. The absence of water enhanced the **2c** formation (48%), whereas removing oxygen from the solution had no effect. Higher stoichiometric amounts of the starting material resulted in slightly higher **2c** yields. No truncation was observed for **4A** in the absence of a base, even in the presence of tetrapropylammonium bromide used to increase the ionic strength of the solution. When Fischer’s base **4B** instead of indolinium **4A** was used without a base, the **2c** yields were high (30%) only at elevated concentrations of **4B** and a higher temperature (80 °C). Cyanine **2c** was formed in higher yields in the presence of a base with **4B**. Interestingly, **2c** also was formed in a low but still significant yield (8%) without indolinium **4A** at full conversion.

### Effects of Heptamethine Chain Substituents

We evaluated the truncation of Cy7s substituted at the C3′ or C4′ positions of the chain with either electron-withdrawing (EWG) or electron-donating (EDG) substituents (Table 2). Only pentamethines **2** substituted in the *meso*-position or unsubstituted trimethine cyanine **3a** were detected as products. The highest yields of **2** were obtained from Cy7, bearing strong electron-withdrawing groups (F or CN) at the C3′ position (Table 2, entries 2 and 4); a higher reaction temperature enhanced truncation only in the case of **1e** (Table 2, entry 4). The yields of **3a** were negligible for **1c** and **1e**. Cy7s **1b** and **1d** provided lower Cy5 yields at both temperatures (Table 2, entries 1 and 3), but the formation of trimethine **3a** from **1d** was the most efficient (28%, Table 2, entry 3). Cy7 substituted in the C4′ position (**1g** and **1h**) did not give Cy5 products in detectable amounts (Table 2, entries 6 and 7), whereas **3a** was obtained in a low yield (Table 2, entry 1).

**Table 2:**
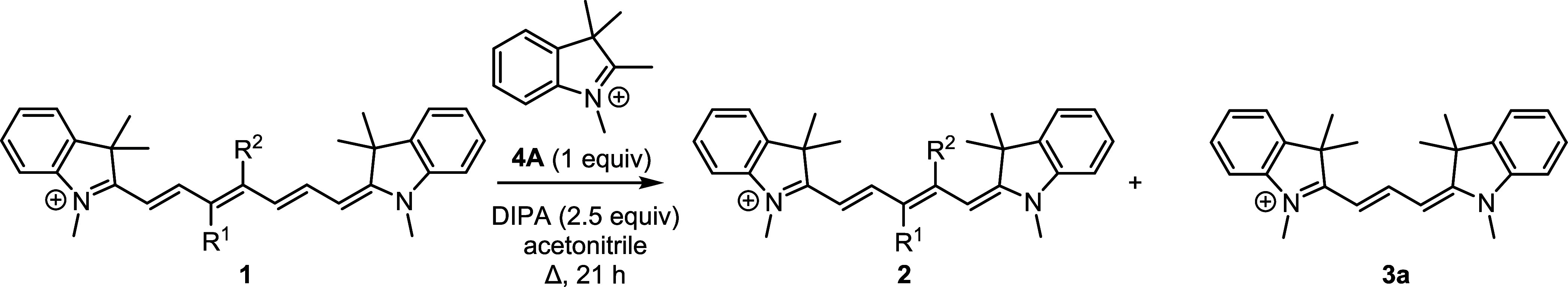
Effects of Chain Substituents^a^

entry	Cy7	R^1^	R^2^	conversion of **1**/% at 50 °C or 80 °C	yield of **2**/%^b^ at 50 °C or 80 °C	yield of **3a**/%^b^ at 50 °C or 80 °C
1	**1b**	H	H	57 (80)	**2b**, 15 (15)	1 (4)
2	**1c**	F	H	94 (100)	**2c**, 38 (33)^c^	0.2 (1)
3	**1d**	COOCH_3_	H	97 (100)	**2d**, 16 (15)	28 (22)
4	**1e**	CN	H	52 (99)	**2e**, 15 (30)^c^	1.5 (4)
5	**1f**	CH_3_	H	32 (98)	**2f**, 1 (2)	4 (15)
6	**1g**	H	CN	93	**2g**, 0	0.3
7	**1h**	H	COOCH_3_	98	**2h**, 0	7

a [**1**] = 72 mM, reaction time = 21 h. Reactions carried out at 50 or 80 °C (in parentheses), iodide as a counteranion is omitted for clarity. The standard deviation from 5 independent measurements was below 2%.

b HPLC yields (calculated assuming 100% conversion of **1**).

c Isolated by precipitation.

**Scheme 3:**
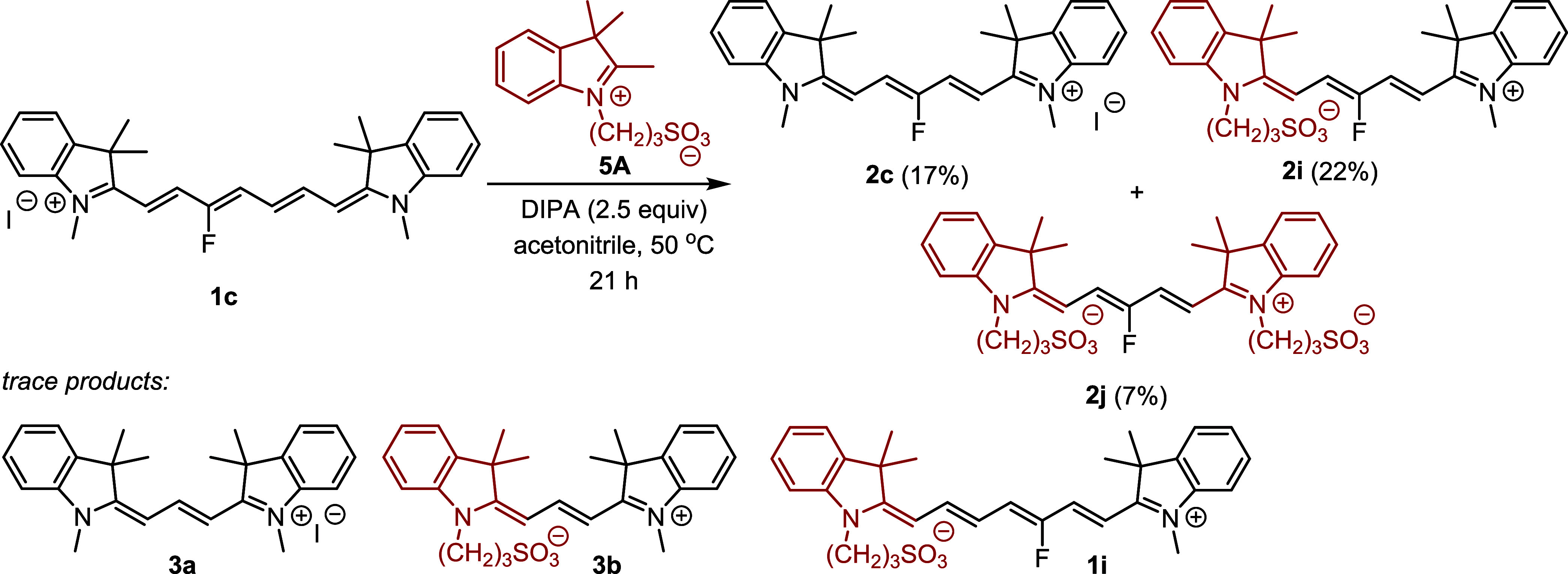
Truncation Reaction Using Different Indolinium Derivatives

We evaluated the reaction of **1c** with *N*-substituted indolinium **5** in acetonitrile at 50 °C to follow its incorporation into the products (Scheme 3). All three possible Cy5 derivatives, symmetrical **2c** and **2j**, and nonsymmetrical **2i**, were formed in high yields. The formation of the double-exchange product **2j** was the least effective, as anticipated, because it involves at least two subsequent reactions. Trace amounts (<2%) of heptamethine and trimethine cyanine derivatives were also detected, as indicated by the HRMS data (Scheme 3 and Figure S23).

### Reactions of Cy5 Derivatives

Because Cy5 derivatives are the primary products of the Cy7 truncation, we examined their stability and reactivity under all reaction conditions. Heating Cy5s **2** in the presence of different indolinium derivatives (**4**–**7**) and DIPA resulted only in Cy5 with exchanged terminal indolinium end groups; thus, the pentamethine chain was preserved (Table 3). Their relative yields were dependent on the starting material stoichiometry, whereas the quality of the indolinium precursor played an only minor role. When large amounts of Fischer’s base **6B** instead of indolinium **6A** were used in the absence of a base, the terminal group exchange was also very efficient. Only traces of Cy3 products were detected (<0.1%, HPLC) in all cases. The presence of an electron-withdrawing group (F, CN) at the *meso*-position was found to be important for indolinium exchange. Unsubstituted pentamethine derivatives were unreactive under the given conditions.

**Table 3:**
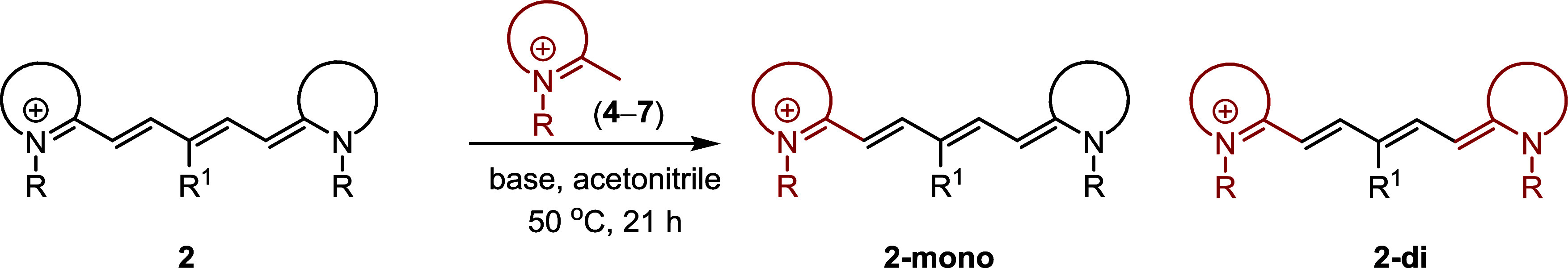

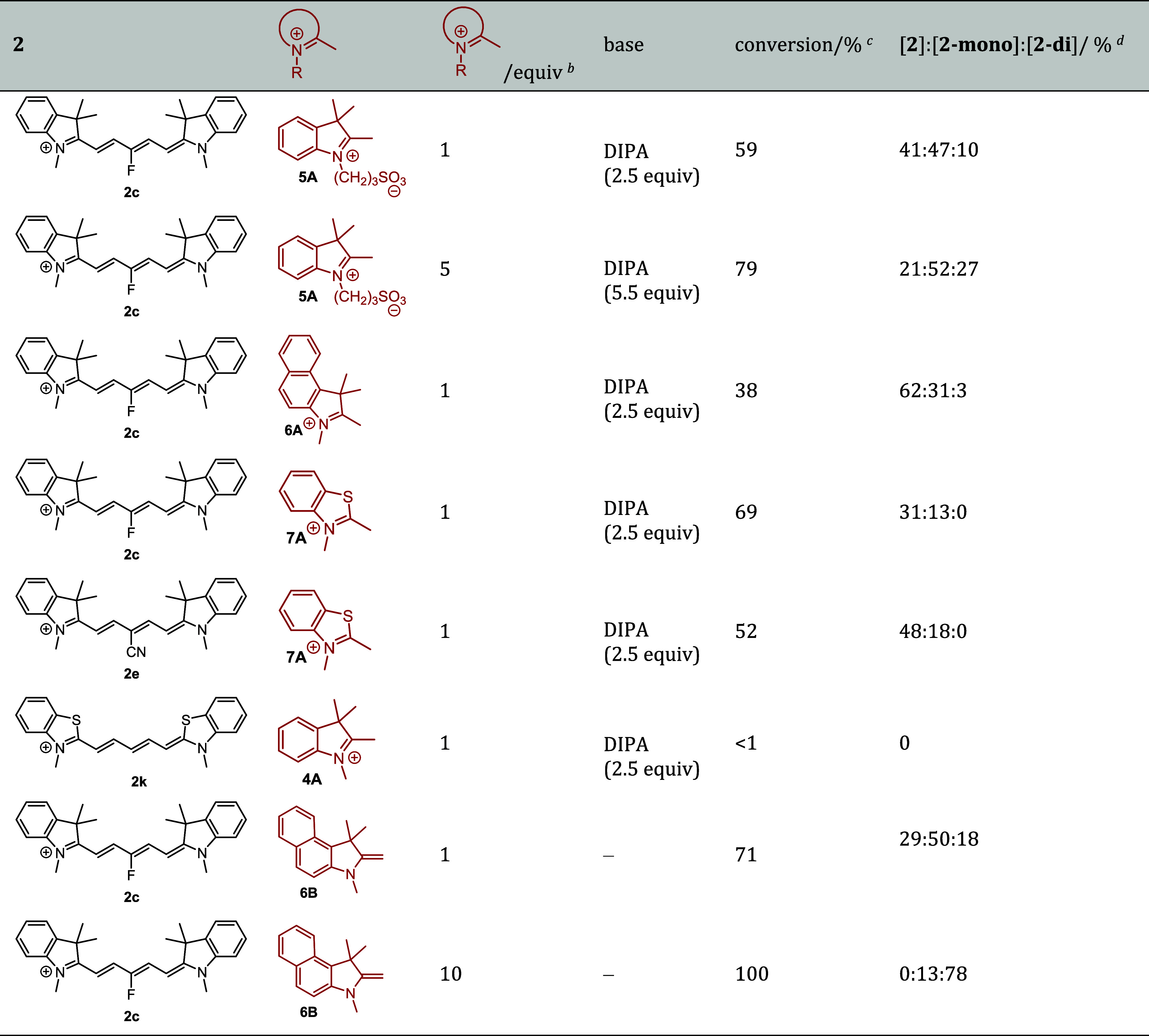
Reactions of Cy5 Derivatives **2**^a^

a [**2**] = 36 mM, reaction time = 21 h; iodide as a counteranion is omitted for clarity. The standard deviation from 5 independent measurements was below 3%.

b The amount of **4**–**7**.

c Reaction conversion.

d HPLC yields (calculated for 100% conversion of **2**).

### Truncation Mechanism of **1c**

**Scheme 4:**
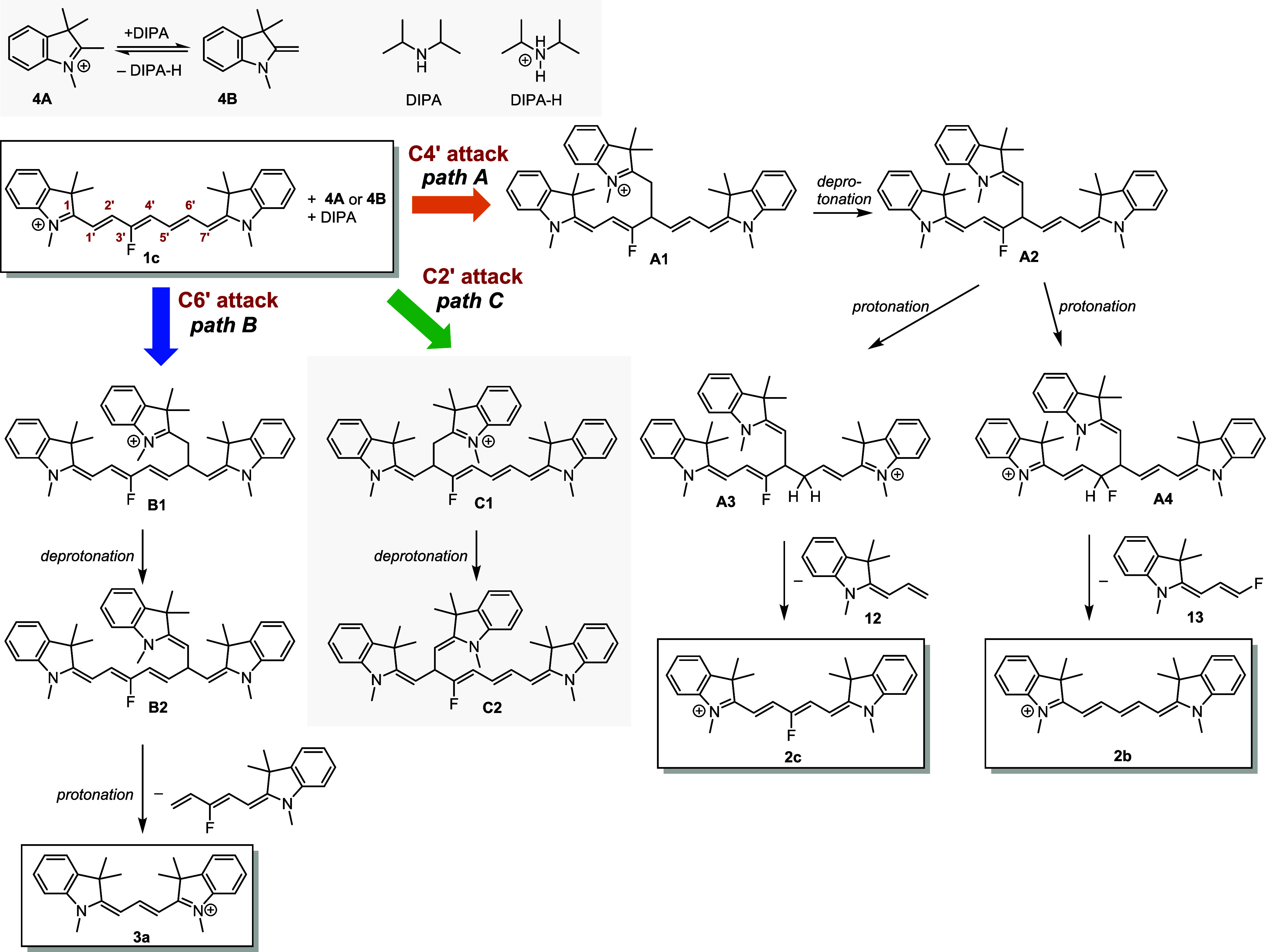
Mechanism of **1c** Truncation in the Presence of **4**

Based on the known Cy7 reactivity^23,24^ and our initial experimental observations, we assumed that the primary reaction step for Cy7 truncation is the nucleophilic addition of an indolinium nucleophile **4B** to the Cy7 polyene chain. Fisher’s base **4B** was generated *in situ* in the presence of DIPA from **4A** in all investigated solvents, ethanol, methanol, and acetonitrile (Figures S71 and S72). A strong base was found to be essential for the deprotonation of **4A** (p*K*_a_ = 8.5) in methanol and acetonitrile solutions (Figures S72 and S73). Three possible electrophilic sites for a nonsymmetrical attack of **1c** are the C4′, C6′, and C2′ chain positions to give three charged adducts **A1**, **B1**, and **C1**, respectively (Scheme 4). They can be deprotonated by DIPA to give **A2**, **B2**, and **C2** and subsequently be protonated at various positions to form, for example, **A3** or **A4** from **A2** (path A, Scheme 4). A nucleophile could also attack the electrophilic iminium C1 carbon, although this site is sterically hindered,^43^ while this reaction step does not lead to Cy7 truncation. Indeed, we identified a product of the reversible addition of a strong nucleophile (MeONa) to C1 of **1c** and **2c** at room temperature (Figures S66–S70), although no addition was detected when **4B** was used.

The atmospheric pressure chemical ionization (APCI) HRMS analysis of the crude reaction mixtures of **1c** reaction with **4** (Table 2) revealed the signal at *m*/*z* 600.3750 corresponding to [M]^+^ of the expected intermediates **A1**, **B1**, and **C1** or [M + H]^+^ of **A2**, **B2**, and **C2** (Scheme 4 and Figure S10). Interestingly, electrospray ionization (ESI) HRMS of the same reaction mixture gave the signals at *m*/*z* 598.3580 and 596.3454, which would correspond to [M–H]^+^ and [M–3H]^+^ of intermediates **A2**, **B2**, and **C2**, respectively (Figure S11). Electrochemical oxidation occurring in the electrospray emitter during mass analysis^44^ is probably the reason for different ion signals in the ESI^+^, which was also reported, for example, for bisindolylmethanes^45^ or dihydropyridines.^46^ Analogous intermediates were also identified in the reactions of other heptamethine derivatives **1** (Table 2) using APCI^+^ and ESI^+^ analyses (Table S2). All attempts to isolate the adducts were unsuccessful, probably due to their rapid decomposition during isolation procedures.

To follow path A, adduct **A2**, protonated by **4A** (p*K*_a_ = 8.5), DIPA-H^+^, or any other acid available at the C5′ position, releases an allylideneindoline fragment **12** to give the truncated **2c** derivative as the major product. Electron-rich butadienyl species, probably formed during this transformation, are reported to be very reactive.^27,47^ They could attack an electron-deficient molecule, such as starting **1c**, to form chain-extended nonamethinecyanines. Indeed, a trace mass signal corresponding to the fluorinated nonamethine derivative was detected by ESI-HRMS (Figure S11). On the other hand, protonation of **A2** at the C3′ position and subsequent release of fluoroallylidene fragment **13** leads to the formation of unsubstituted **2b**, observed as a minor product. The **2b** yield increased at elevated temperatures (50 → 80 °C), which could be related to different kinetic factors.

Adducts **B2** and **C2** (paths B and C, Scheme 4) can produce Cy3 **3a** (but not **2c** or **2b**) upon protonation and release of the corresponding indoline fragments. As **2c** and **2b** are the major observed products, the formation of the **B2** and **C2** intermediates must be inefficient.

**Figure 1:**
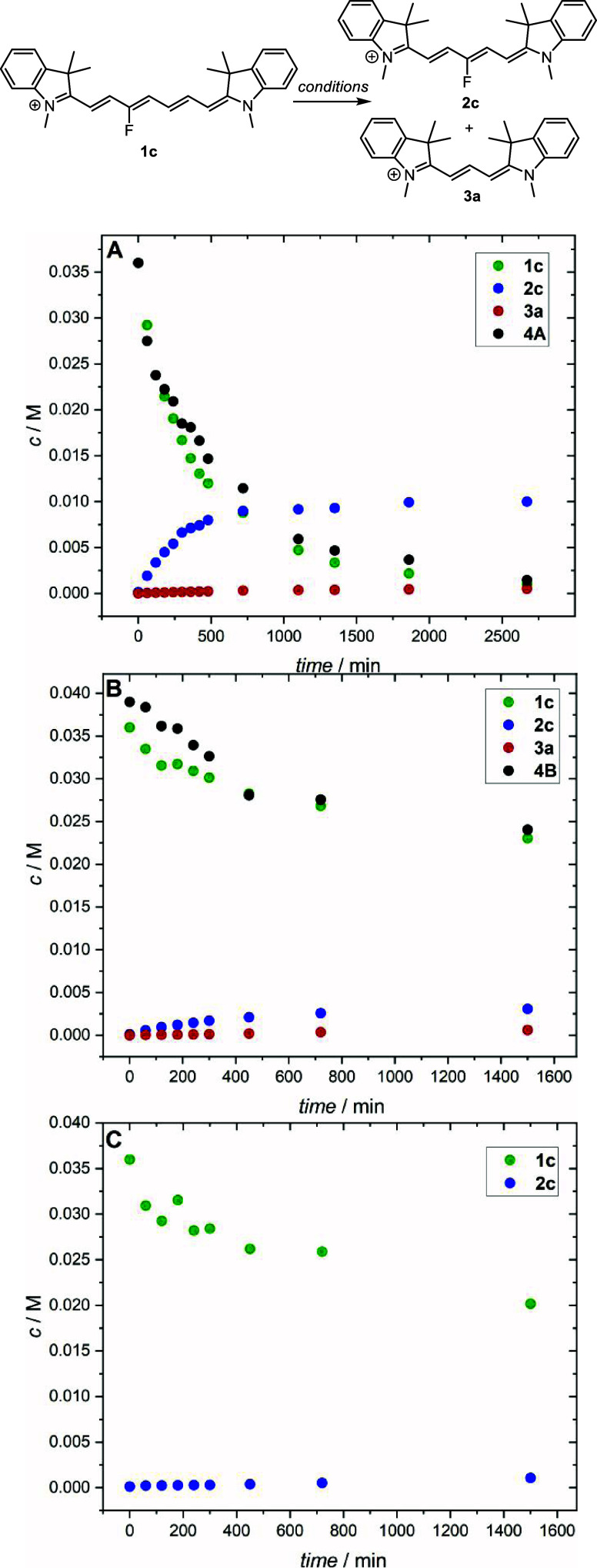
Concentration profiles for the reactions of **1c**. Reaction conditions: (A) **1c** (36 mM), **4A** (36 mM), and DIPA (9 mM) in acetonitrile (10 mL) stirred at 50 °C; (B) **1c** (36 mM) and **4B** (36 mM) in acetonitrile (10 mL) stirred at 50 °C; (C) **1c** (36 mM) and DIPA (9 mM) in acetonitrile (10 mL) stirred at 50 °C.

To understand the role of a base (DIPA) in the truncation process, we investigated the reaction kinetics of **1c** in acetonitrile giving products **2c** and **3a** under three different conditions: in the presence of (a) DIPA and indolinium salt **4A**, (b) Fischer’s base **4B** only (no DIPA), and (c) DIPA only (no **4**) (Figure 1). The most efficient product **2c** formation but inefficient **3a** formation was observed under the conditions (a). The kinetic data were fitted to a second-order reaction of **1c** + **4A** → **2c** (Figure S3), and the rate constant of the **2c** formation was essentially the same in methanol ((1.77 ± 0.1) × 10^–3^ M^–1^ s^–1^) and acetonitrile ((1.70 ± 0.1) × 10^–3^ M^–1^ s^–1^). The absence of DIPA in (b) did not stop the truncation process; the reaction was only slowed down with an estimated rate constant of the **2c** formation of (2.0 ± 0.2) × 10^–4^ M^–1^ s^–1^ in acetonitrile (when Fischer’s base **4B** was used in the absence of a base, the truncation was always less efficient, Table 1). The reaction (c) led to an insignificant conversion of the starting material to give **2c** with a reaction rate constant of (5.7 ± 0.7) × 10^–6^ s^–1^ in acetonitrile; the formation of **2c** was negligible. We conclude that DIPA must be productively involved in **4A**/**4B** conversion and subsequent acid–base equilibria. Since the truncated reaction is very complex and the yields strongly depend on the reaction conditions and competing processes, we performed the following quantum-chemical calculations to elucidate in detail the elementary steps of the productive pathways.

### Quantum-Chemical Calculation: Energies vs Free Energies

The above-suggested mechanism (Scheme 4) poses multiple challenges for theoretical treatment. First, the catalytic steps involve changes in the molecularity; thus, we can expect a significant contribution from entropic and solvent effects. The proper modeling of these effects is a matter of heated discussions, mainly revolving around the coverage of translational entropy.^41,42,48−50^

There is extensive empirical evidence that full inclusion of the translational entropy with most standard density functional theory (DFT) functionals leads to an overestimation of activation energies by ∼0.3 eV for bimolecular transition states.^51^ Some researchers have suggested that the effect of translational entropy on free energy should be ignored entirely in solutions.^52,53^ However, this approach is difficult to justify. The experimental data in organic chemistry were also reproduced with concepts based on the free volume that effectively reduces the contribution of translational entropy.^49^ A similar effect was achieved within the explicit scaling approaches, with only a fraction of the calculated translational entropy included.^50^ As an example, Singleton has successfully applied the Δ*G*_50%_ method for modeling the Morita–Baylis–Hillman reaction with similar acid–base catalytic cycles considered in this work.^41^ Again, we see a minimal theoretical foundation for the latter treatment; however, the method works well in practice. Sunoy and co-workers argued that the free energy surface matching the experimental data for the same reaction could be obtained with no translational entropy scaling or any other *ad hoc* treatment, providing that high-level electronic structure methods are used and solvent entropy is adequately described.^42^ Perrin and co-workers then strongly support the view that the full contribution of translational entropy from the Sackur–Tetrode equation should be included.^48^ The results presented here are consistent with their conclusions.

**Figure 2:**
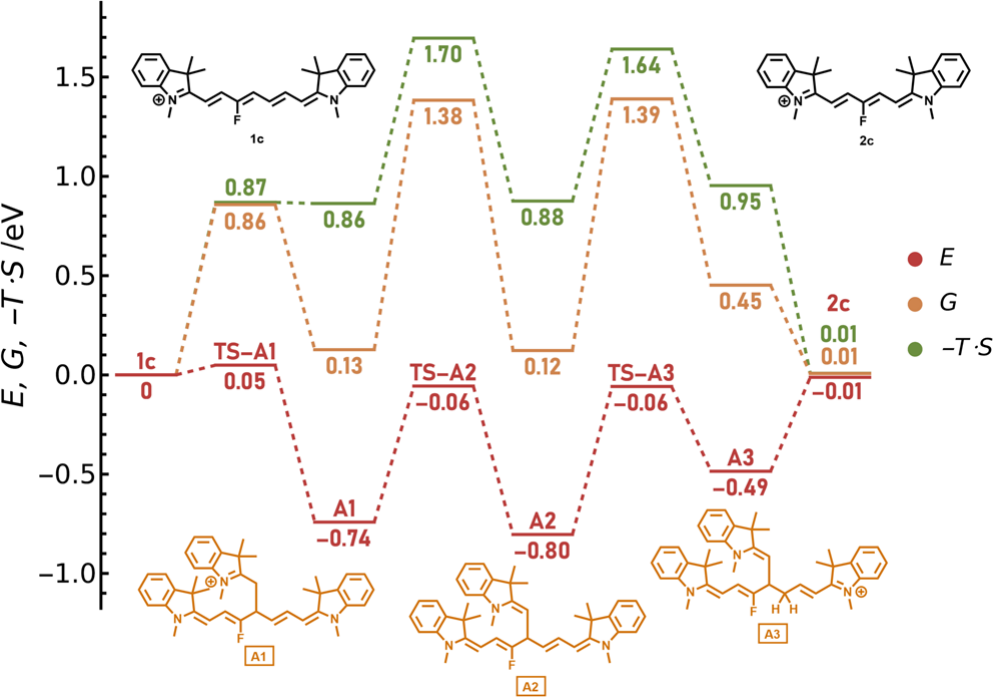
Calculated electronic (red) and Gibbs (orange) free energy profiles and entropic contributions (green) at 358 K for a path following the attack of **4B** to C4′ of **1c** (Scheme 4, path A). “TS” stands for the corresponding transition state. The single-point electronic energies were calculated at the DLPNO–CCSD(T)/cc-pVTZ level in ethanol within the polarizable continuum model (PCM). The picture of the process did not change much with the solvent. Thermal corrections were evaluated in the gas phase at the PBE0/def2-TZVP level with a D3BJ dispersion correction. The inclusion of solvation at the DLPNO–CCSD(T) level is described in the Methodology section (Supporting Information). The structures were optimized at the same level as for the frequency calculations.

The role of the entropic effects in our systems is demonstrated on the **1c** truncation through the addition of **4B** to C4′ (path A, Scheme 4). The electronic energy profile (*i.e.*, the energetics ignoring thermal and entropic contributions) (red line, Figure 2) indicates that the main products should be two intermediates, **A1** and **A2**, whereas the final product **2c** is destabilized by 0.79 eV against the lowest-energy structure. The highest calculated activation barrier is 0.74 eV. From a naïve perspective, one would expect that the reaction leading to the trimolecular complex **A2** mixed with the **A1** adduct is relatively fast.

The Gibbs free energies (orange line, Figure 2) differ significantly from the electronic energies thanks to entropic effects (green line, Figure 2). The calculated free energy profile perfectly agrees with the experimental observations, including the observed products and the reaction kinetics. The electronic energies were calculated with a high-level *ab initio* DLPNO–CCSD(T)/cc-pVTZ method (Figures 2 and S75), and no correction for the translational entropy was needed (see the Supporting Information for further discussion). We can compare these results with the free energies evaluated at the density functional level (PBE0/def2-TZVP/D3BJ, Figure S76). While the entropy addition correctly disfavors the intermediates, the calculated barriers at the DFT level are, in contrast to DLPNO-CCSD(T) results, too large to expect the reaction to occur within tens of hours. The Δ*G*_50%_ correction results are then consistent with those of the experiment (Figure S76). Our results thus seem to support the views of Perrin and Sunoy mentioned above;^42,48^ that is, no further corrections to the entropic terms are needed when an appropriate level of theory is used. At the same time, when common DFT calculations are used, the scaling approaches represent an approach to model catalytic reactions even if they are based on error cancelation. The DLPNO–CCSD(T) method^54,55^ is a pragmatic way to achieve coupled-cluster quality results at an affordable computational cost, even for relatively complex systems. We performed all our calculations using the ORCA 5.0.3 package.^56^

**Figure 3:**
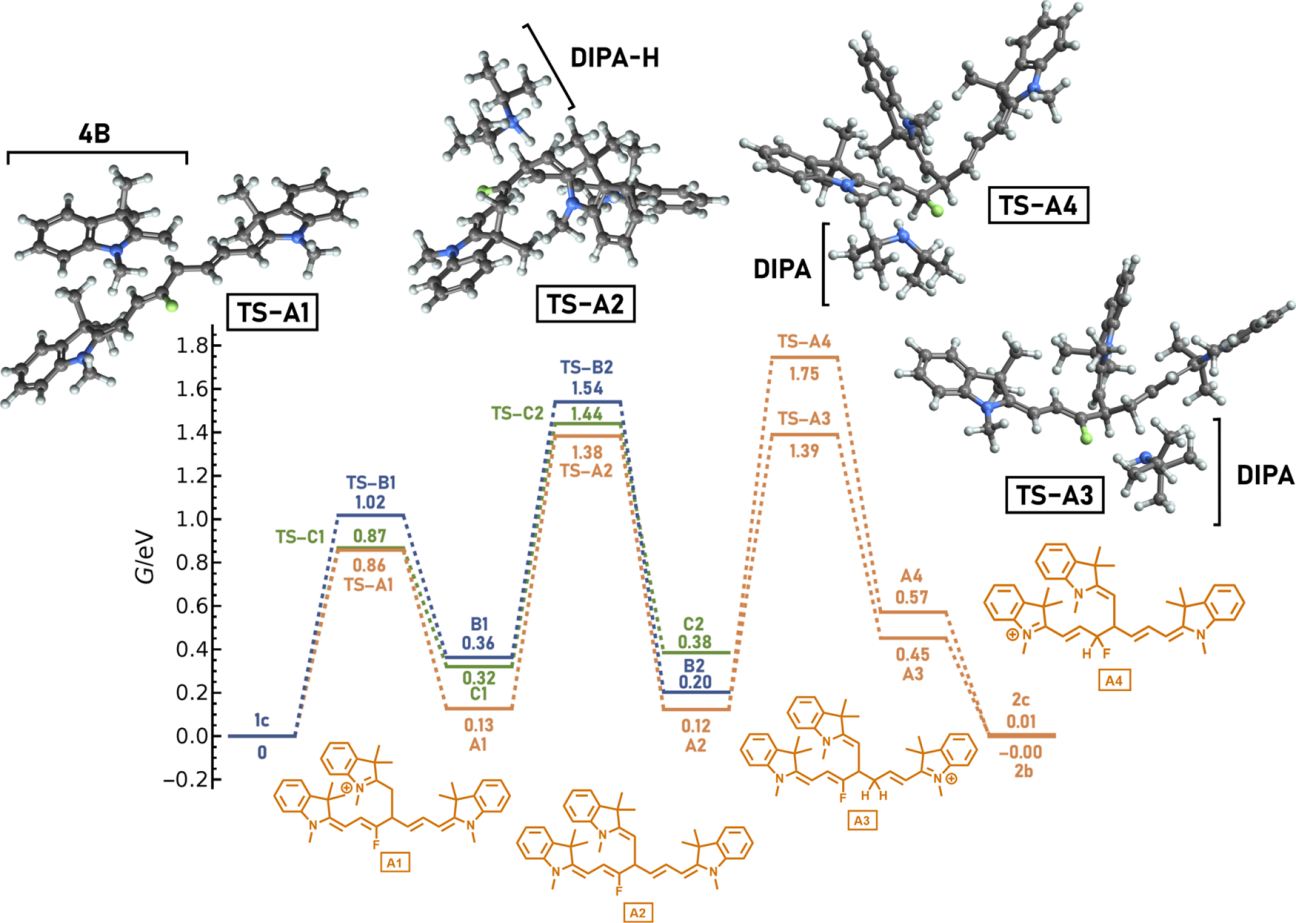
Gibbs free energies of the addition of **4B** to **1c** (Scheme 4) in ethanol at the standard state of 1 M at 358 K. The structures were optimized in the gas phase at the PBE0/def2-TZVP level with the D3BJ dispersion correction. Single-point energies were calculated at the DLPNO–CCSD(T)/cc-pVTZ level. Frequency calculations were conducted at the same level as optimization with the inclusion of solvation (at the DLPNO–CCSD(T)/cc-pVTZ level) described in the Methodology section (Supporting Information).

Further effects influencing the Gibbs free energy profile and reaction rates were considered. The reaction rate for a path following the attack of **4B** to **1c** is slightly increased thanks to hydrogen tunneling, which causes a decrease in the apparent activation energies. The accelerating effect of hydrogen tunneling occurs because the rate-limiting step is proton transfer. The corrections were calculated according to Bell’s method.^57^ For deprotonation by DIPA (**A1** → **A2**), the decrease in the activation Gibbs free energy by a quantum tunneling correction is up to 0.08 eV, whereas protonation by DIPA-H (**A2** → **A3**) gives the Gibbs free energy lowered by 0.04 eV. We also found that deprotonation of **A1** by DIPA can occur through four different transition states with almost identical energies. This leads to further decreased activation entropy, resulting in the activation Gibbs free energy dropping by 0.04 eV at 358 K. All of these effects are included in the energetic profiles in Figures 2, 3, S75, and S76.

In the presence of DIPA, **4A** is deprotonated to **4B**, which is accompanied by a decrease in the Gibbs free energy by 0.17 eV, implying an equilibrium constant of *K* = 260 (at the DLPNO–CCSD(T)/cc-pVTZ level in ethanol with thermal corrections from the PBE0/def2-TZVP/D3BJ level using a standard state of 1 M at 358 K). The C4′ attack of **4B** to **1c** (leading to **A1**) has the lowest transition state (Figure 3) when compared to the C2′ and C6′ attacks (giving **C1** and **B1**, respectively). Moreover, **A1** has the lowest free energy among the three resulting adducts **A1**, **B1**, and **C1**, with the other two being positioned about 0.2 eV higher. Thus, the C4′ attack is both kinetically and thermodynamically preferred over the C2′ and C6′ attacks, as supported by the experimental results (see above). The C4′ adduct has the lowest activation Gibbs free energy for the subsequent deprotonation step with DIPA (related to starting **1c**) to form **A2**. This deprotonated product is then the most stable compared to the other two concurrent deprotonated products **B2** and **C2**, obtained from the C6′ and C2′ adducts, respectively.

From this point on, we discuss only the calculation of the C4′ attack pathway. The pathway to the truncated fluorinated **2c** product leads through the protonation of **A2** at the C5′ carbon (here, by DIPA-H), forming **A3**, which then decomposes to **2c**. These steps are energetically more favorable than the C3′ protonation pathway, which requires a free energy higher by 0.36 eV to cross the activation barrier. This pathway then leads to an intermediate **A4**, which is by 0.12 eV less stable than **A3**. The final product is then **2b**, which has almost the same relative energy as **2c**. The steps to form **2b** and **2c** require cleavage of the C–C bond. However, the bond is weakened by protonation on one of the carbon atoms. The higher activation energy for C3′ protonation corresponds to the observed temperature dependence of the **2b**/**2c** product ratio discussed above. As both species have the same free energy, their yields must be kinetically controlled.

Secondary (DIPA) or tertiary (DIPEA) amines such as **4B** can serve as bases. When only **4B** is used (i.e., no DIPA or DIPEA), the Gibbs free energy change for the deprotonation of **A1** to give **A2** in the presence of **4B** is 0.18 eV. A comparison of the value for deprotonation with DIPA as a base (0.01 eV) provides one of the possible reasons for the lower truncation yields when **4B** is used alone. Note that the product is almost isoenergetic with respect to the reactants. This would indicate the formation of an equimolar mixture of both species. However, the reaction proceeds irreversibly because the other product (**12**) is probably very reactive and reacts further, as mentioned above.

### Reactions of Other Cy7 Derivatives

The presence of an electron-withdrawing group (F, CN, and COOMe) in **1** or the absence of substituents are favorable for the formation of **2**, whereas a negligible amount of the truncation product was observed for **1f** (3′-methyl derivative; Table 2). This is most likely related to the chain activation for nucleophilic attack. It is possible that **A1** and **C1** and the subsequent intermediates are energetically sterically disfavored by more sterically demanding substituents (COOCH_3_ in **1d**) on the C3′ carbon. On the other hand, any substituent in the C4′ position of **1** (Table 2) has a negative effect on truncation, which may, at least partially, be associated with a higher steric demand at the C4′ position. Indeed, the calculated activation free energy of **4B** attack to the C4′ of **1g** is 0.96 eV, which is higher than that for **1c** by 0.1 eV. The resulting adduct is less energetically stable than the **A1** adduct by 0.28 eV (marked as F1 in Figure S77).

### Truncation with Secondary Amine

**Scheme 5:**
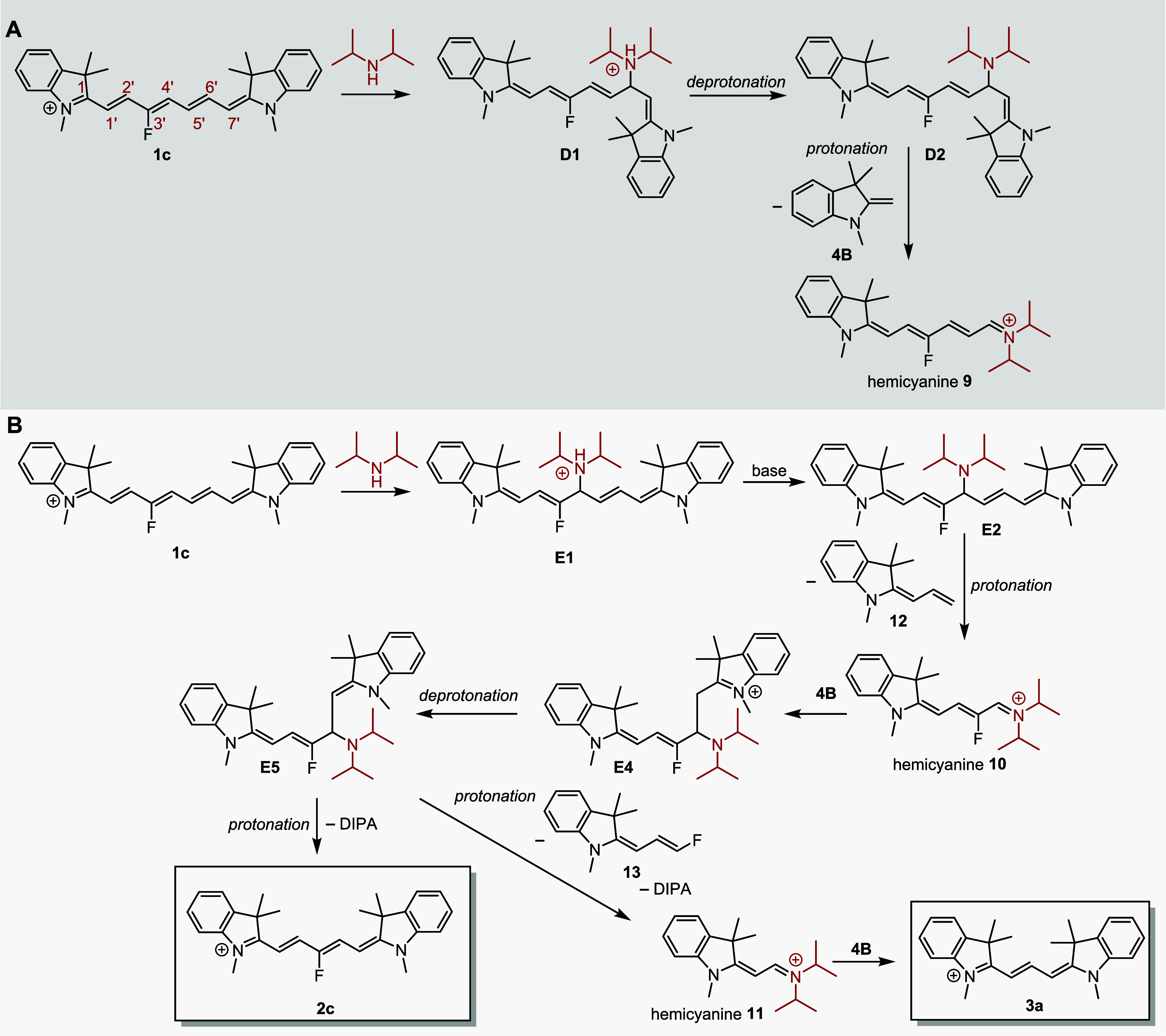
Truncation of **1c** with DIPA

The reaction of **1c** in the absence of Fischer’s base but in the presence of a secondary amine (DIPA) still leads to truncation, although with very low yields (Table 1). Therefore, we considered the reaction mechanism in which DIPA acts as a nucleophile, attacking the cyanine chain. The addition to the C6′ position resulted in the formation of hemicyanine **9** and the release of product **4B**, the masses of which were confirmed by HRMS (Scheme 5A and Figure S38). The released **4B** could subsequently attack the starting **1c** to give **2c** via the mechanism described above (Scheme 4). The addition of DIPA to C4′ would provide hemicyanine **10**, which could lead to **2c** in the presence of released indolinium (Scheme 5B). As shown above (Figure 1c), this is a considerably slower process than the addition of **4A** or **4B** to **1c**. However, as the presence of a tertiary amine (DIPEA) leads to the same results as DIPA (Table 1), indolinium could also be generated by an alternative degradation pathway, which we did not investigate further. In agreement with the experimental data, the calculation of the pathway shown in Scheme 5B provided high Gibbs free energy values for the suggested adduct intermediates, which indicates a low probability of this reaction step (Figure S78).

### Exchange of Terminal Heterocycles in Cy5

**Scheme 6:**
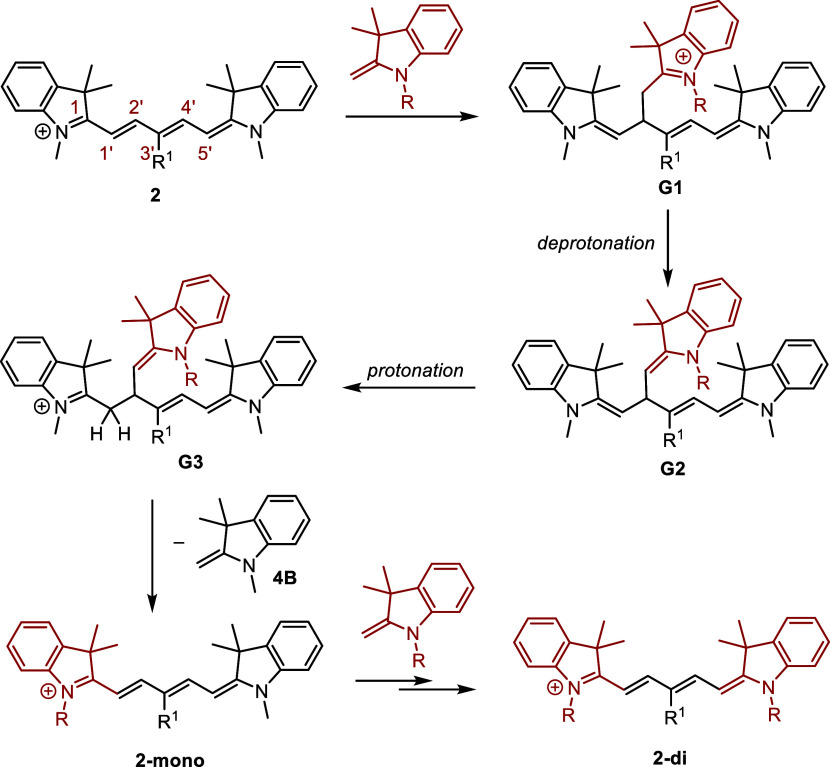
Exchange of Terminal Heterocycles in **2**

Pentamethine cyanines do not undergo truncation to give Cy3 derivatives under the studied conditions. However, the exchange of heterocyclic ends was observed in Cy5s substituted with EWG at the C3′ position (Table 3). The presence of an EWG in the polyene chain increases its electrophilicity, making the nucleophilic attack of indolinium more probable. The mechanism involving several steps identical to those in the Cy7 → Cy5 conversion is shown in Scheme 6. The reaction proceeds efficiently with Fischer’s base in the absence of a base (the reaction with **6B**, Table 3); therefore, the primary and probably only productive role of DIPA in all other processes is the deprotonation of the starting indolinium salt.

## Conclusions

We found that heptamethine cyanines can be cleaved in various positions of the polymethine chain and converted to pentamethine cyanines in the presence of heterocyclic nucleophiles such as an indolinium salt at elevated temperatures in both protic and aprotic solvents under abiotic conditions. This truncation reaction is efficient and is generally applicable to different Cy7 derivatives and nucleophiles.

Our mechanistic analysis revealed that truncation is strongly affected by the quality of nucleophiles and reaction conditions and that electron-withdrawing groups significantly enhance the reactivity of the polyene chain. The resulting Cy5 products do not undergo chain shortening under the given conditions, but instead they exhibit the exchange of heterocyclic end groups via the attack of a nucleophile to the C2′ position. The suggested mechanisms were supported by computational analyses, providing activation barriers consistent with the experimentally observed rates and amounts of identified intermediates. This work expands knowledge about the reactivity of cyanine dyes, which can contribute to the suppression of side processes that often occur during the synthesis of these dyes.

Our work can also contribute to the discussion about the role of computational chemistry in revealing the mechanisms of homogeneous catalytic reactions. While Singleton has expressed a somewhat skeptical view on the fundamental importance of the *ab initio* computational predictions,^41^ Harvey and his co-workers responded more optimistically.^42^ Our present case supports Harvey’s views on the role of computational chemistry and the technology that should be utilized. The truncation reaction mechanisms discussed in this work were not suggested based on computational studies; instead, they were proposed through intuition and experience of organic chemists. Yet computational techniques can distinguish between different pathways and support the quantitatively consistent mechanism with the experimental observations. Our work suggests that computational techniques beyond the density functional theory family represent a safe choice. The coupled-cluster calculations can be efficiently performed with locally correlated techniques for medium-sized molecules, and this technique provides quantitative treatment of catalytic reactions.

## Supporting Information Available

Materials and methods; synthesis; NMR, absorption, and emission data; and quantum-chemical calculations (PDF)
