# Supplementary material for: Thermal Truncation of Heptamethine Cyanine Dyes
Source: J Am Chem Soc. 2024 Jul 12;146(29):19768–81. doi: 10.1021/jacs.4c02116 (PMC11273355; doi:10.1021/jacs.4c02116)
Supplement: Supplementary file 1 — ja4c02116_si_001.pdf [file ja4c02116_si_001.pdf]

Supporting Information

Thermal Truncation of Heptamethine Cyanine Dyes

Jana Okoročenkova,[†,‡] Josef Filgas,[¶] Nasrulla Majid Khan,[†,‡] Petr Slavíček,[¶,*] Petr Klán[†,‡,*]

[†] Department of Chemistry, Faculty of Science, Masaryk University, Kamenice 5, 625 00, Brno, Czech Republic.
[‡] RECETOX, Faculty of Science, Masaryk University, Kamenice 5, 625 00, Brno, Czech Republic.
[¶] Department of Physical Chemistry, University of Chemistry and Technology, Technická 5, 16628 Prague 6, Czech Republic.

* Petr Slavíček: Petr.Slavicek@vscht.cz; Petr Klán: klan@sci.muni.cz

Contents

Materials and Methods S2
Synthesis S2
Kinetic Measurements S6
HPLC and HRMS data S8
NMR Spectra S46
Computational Analyses S82
References S143

Materials and Methods

Reagents and solvents of the highest purity available were used as purchased unless stated otherwise. The synthetic procedures were performed under an ambient atmosphere unless stated otherwise. Column chromatography was performed using silica gel 60. ^{1}H, ^{13}C, ^{19}F NMR spectra were measured at ambient temperature in CD_3OD at 300 and 500 MHz. ^{1}H chemical shifts are reported in ppm relative to tetramethylsilane (δ = 0.00 ppm), residual CD_3OD (3.31 ppm for ^{1}H NMR and 49.0 ppm for ^{13}C NMR), and internal $CFCl_3$ (0 ppm for ^{19}F NMR); coupling constants (J) are reported in Hz.

Absorption spectra were obtained with matched 1.0 cm quartz cuvettes using a UV-vis spectrometer. Molar absorption coefficients were determined from the absorption spectra (the average values were obtained from three independent measurements with solutions of different concentrations). Fluorescence was measured on an automated luminescence spectrometer in 1.0 cm quartz fluorescence cuvettes at 23 ± 1 °C; sample concentrations with absorbance below 0.1 at the excitation wavelength were used. Fluorescence quantum yields were determined on a fluorimeter as absolute values using an integrating sphere. The quantum yields were measured five times and averaged for each sample. The solution concentrations were adjusted to have absorbance below 0.15.

The exact masses of synthesized compounds were obtained using a triple quadrupole electrospray ionization (ESI) mass spectrometer in a positive or negative mode coupled with direct inlet or liquid chromatography (nitrogen flow 5 L min^{-1}, gas temperature: 325 °C, nebulizer 45 psig, skimmer 65 V, V_{cap} –2500 V, fragmentor: 60 V; in methanol) and atmospheric pressure chemical ionization (APCI) mass spectrometer in a positive or negative mode coupled with direct inlet or liquid chromatography (nitrogen flow 5 L min^{-1}, gas temperature: 325 °C, nebulizer 45 psig, skimmer 65 V, vaporizer 200 °C, fragmentor: 60 V; in dichloromethane).

HPLC analyses were carried out on a chromatograph equipped with a photodiode array detector (200–900 nm) using Zorbax SB-Aq C18 column at 30 °C and flow rate of 1 mL min^{-1} (3.5 µm, 4.6 × 150 mm). The mobile phase was 0.1% trifluoroacetic acid in water (A) and acetonitrile (B), programmed from 10% to 90% of B over 6 min, from 90% to 95% of B over 0.5 min, kept 95% B for 0.5 min, and re-equilibrated with 10% of solution B for 3 min.

The HPLC-MS analyses were carried out on a UHPLC-MS system consisting of a UHPLC chromatograph equipped with a photodiode array detector (200–600 nm) and triple quadrupole electrospray ionization mass spectrometer using a Zorbax SB-Aq C18 column at 30 °C and flow rate of 1 mL min^{-1} (3.5 µm, 4.6 × 150 mm). The mobile phase was 0.1% trifluoroacetic acid in water (A) and acetonitrile (B), programmed from 10% to 90% B over 6 min, from 90% to 95% B over 0.5 min, kept 95% B for 0.5 min and re-equilibrated with 10% of solution B for 3 min.

The pK_a value of **4A** was estimated by titration of **4A** in d_4-CD_3OD:D_2O/d_6-DMSO (1:1:0.05) using diisopropylamine (DIPA), following the formation of **4B** by ^{1}H NMR (Figure S74). The amount od DIPA required for the 50% conversion of **4A** to **4B** was calculated; then, the corresponding solution of **4A**/DIPA in a methanol-water-DMSO (1:1:0.05) mixture was prepared, and its pH value was measured and calculated using a correction factor according to the reported method.[1]

Synthesis

Heptamethine cyanine dyes **1a**–**h**,[2-3] pentamethine cyanine **2b**,[4] trimethine cyanine **3a**,[5] heterocyclic salts **4A**,[6] **6**,[6] **7**,[6] **5**,[7] and **6B**[8] were prepared according to previously published procedures.

General Procedure for the Synthesis of Pentamethine Cyanine Dyes from Heptamethine Cyanines. The corresponding heptamethine cyanine dye **1** (1 eq; 72 mM), heterocyclic salt **4A** (1 eq), and diisopropylamine (2.5 eq) were dissolved in acetonitrile, and the mixture was stirred at 50–80 °C for 21 h (Figure S1, Table S1). The reaction mixture was analyzed by HPLC and HRMS (Figure S12-S22). HPLC yields of cyanines were determined using calibration curves for **1b**, **2b**, and **3a** and recalculated with molar absorption coefficients (ε_{max}/mol^{-1} dm^3 cm^{-1}) of the corresponding compound.

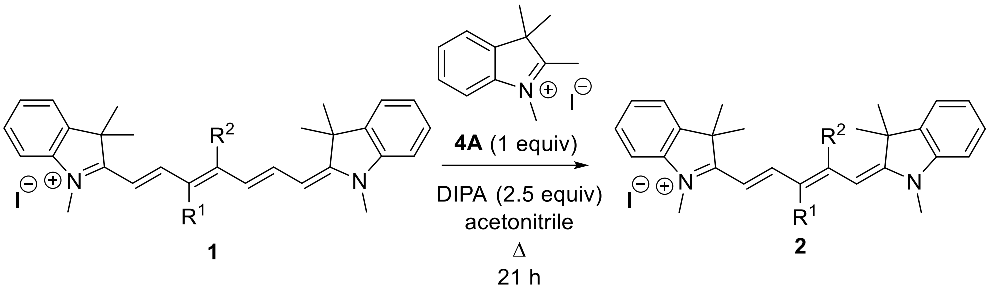

Figure S1. Synthesis of pentamethine cyanine dyes from heptamethine cyanine dyes.

Table S1. Summary of the prepared pentamethine cyanine dyes.

**1**	**R^1**	**R^2**	**2**
**1b**	H	H	**2b**
**1c**	F	H	**2c**
**1d**	$COOCH_3$	H	**2d**
**1e**	CN	H	**2e**
**1f**	CH_3	H	**2f**

2-((1*E*,3*Z*)-3-Fluoro-5-((*E*)-1,3,3-trimethylindolin-2-ylidene)penta-1,3-dien-1-yl)-1,3,3-trimethyl-3*H*-indol-1-ium iodide (2c): **1c** (504 mg; 0.91 mmol), **4A** (301 mg, 1.0 mmol), and diisopropylamine (328 μL, 2.28 mmol) were dissolved in acetonitrile (12.5 mL), and the mixture was stirred at 50 °C for 21 h The reaction mixture was concentrated under vacuum, and cold diethyl ether (0 °C, 50 mL) was added. The resulting precipitate was filtered off, washed with water (3 × 20 mL) and diethyl ether (3 × 20 mL), and dried on air. Yield: 173 mg (36% yield). Blue solid. ^{1}H NMR (300 MHz, d_4–CD_3OD): δ (ppm) 7.83 (dd, J = 28.5, 14.2 Hz, 2H), 7.25–7.55 (m, 8H), 6.28 (d, J = 14.2 Hz, 2H), 3.67 (s, 6H), 1.74 (s, 12H); ^{19}F NMR (300 MHz, d_4–CD_3OD) δ (ppm) –133.75 (t, 1F, J = 28.5); ^{13}C NMR (500 MHz, d_4–CD_3OD): δ (ppm) 175.4, 144.2, 142.7, 137.2, 129.8, 126.7, 123.3, 112.2, 97.8, 50.7, 31.7, 27.4 (Figures S39–S45). HRMS (ESI$^+$) *m/z* calcd for $C_{27}H_{30}FN_2^+$ [M – I$^-$]$^+$ 401.2388, found 401.2390. Optical properties (in MeOH): λ_{max}(abs) = 652 nm (ε_{max} = 2.00 × 10^5 mol^{-1} dm^3 cm^{-1}), λ_{max}(em) = 678 nm (Φ_F = 0.07).

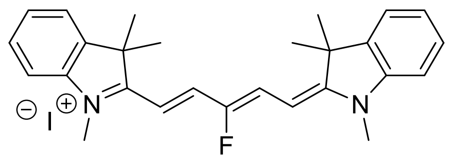

2-((1*E*,3*Z*)-3-Cyano-5-((*E*)-1,3,3-trimethylindolin-2-ylidene)penta-1,3-dien-1-yl)-1,3,3-trimethyl-3*H*-indol-1-ium iodide (2e): 1e (202 mg; 0.36 mmol), **4A** (120 mg, 0.4 mmol), and diisopropylamine (130 μL, 0.9 mmol) were dissolved in acetonitrile (5 mL), and the

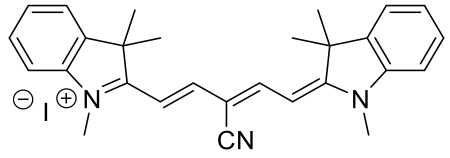

mixture was stirred at 80 °C for 21 h The reaction mixture was concentrated under vacuum, and cold diethyl ether (0 °C, 30 mL) was added. The resulting precipitate was filtered off, washed with water (3 × 10 mL) and diethyl ether (3 × 10 mL), and dried on air. The crude product was purified using column chromatography (CH_2Cl_2/CH_3OH = 20 : 1). Yield: 27 mg (14% yield). Blue solid. ^{1}H NMR (300 MHz, d_4–CD_3OD) δ (ppm) 8.51 (d, J = 14.6 Hz, 2H), 7.38–7.62 (m, 8H), 6.45 (d, J = 14.6 Hz, 2H), 3.79 (s, 6H), 1.79 (s, 12H); ^{13}C NMR (500 MHz, d_4–CD_3OD): δ (ppm) 177.1, 153.4, 142.4, 141.8, 128.6, 126.3, 122.1, 115.3, 111.7, 101.3, 99.2, 50.1, 30.8, 25.9 (Figures S46–S49). HRMS (ESI$^+$) m/z calcd for $C_{28}H_{30}N_3^+$ [M – I$^-$]$^+$ 408.2434, found 408.2430. Optical properties (in MeOH): λ_{max}(abs) = 613 nm (ε_{max} = 1.19 × 10^5 mol^{-1} dm^3 cm^{-1}), λ_{max}(em) = 633 nm (Φ_F = 0.15).

Synthesis of Unsymmetrical Pentamethine Cyanines 2c, 2i, 2j from Heptamethine Cyanine 1c. Heptamethine cyanine dye **1c** (1 eq; 18–72 mM), heterocyclic salt **5** (1.1 eq), and diisopropylamine (2.5 eq) were dissolved in acetonitrile, and the mixture was stirred at 50 °C for 21 h. The reaction mixture was analyzed by HPLC and HRMS (Figure S23-S25).

General Procedure for the Synthesis of Pentamethine Cyanines 2 by Heterocyclic End Exchange. The corresponding pentamethine cyanine dye **2** (1 eq; 18–72 mM), heterocyclic salt **4-7** (1 eq) and diisopropylamine (2.5 eq) were dissolved in acetonitrile, and the mixture was stirred at 50 °C for 21 h (Figure S2). The resulting products were analyzed by HRMS and HPLC (Figures S26–S37).

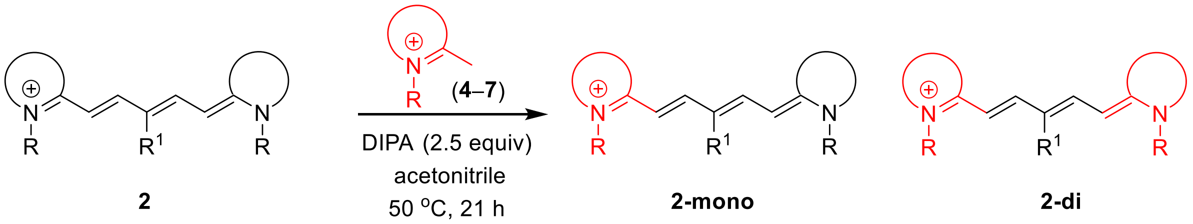

Figure S2. Synthesis of pentamethine cyanine dyes by heterocyclic end exchange.

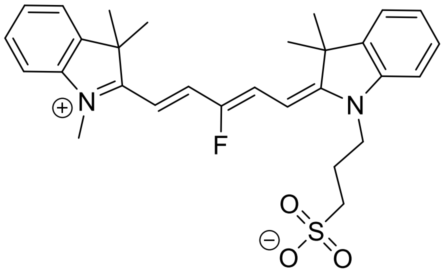

3-((*E*)-2-((2*Z*,4*E*)-3-Fluoro-5-(1,3,3-trimethyl-3*H*-indol-1-ium-2-yl)penta-2,4-dien-1-ylidene)-3,3-dimethylindolin-1-yl)propane-1-sulfonate (2i). The compound was prepared from **2c** and heterocyclic salt **5A** according to a general procedure and purified by flash column chromatography (CH_2Cl_2/CH_3OH = gradually from 20 : 1 to 3 : 1). Yield: 30 mg (18%). Blue solid. ^{1}H NMR (300 MHz, d_4–CD_3OD): δ (ppm) 7.88 (m, 2H), 7.25–7.55 (m, 8H), 6.33 (d, J = 14.1, 2H), 4.32–4.48 (m, 2H), 3.69 (s, 3H), 3.01 (t, J = 6.8 Hz, 2H), 2.29 (q, J = 6.8 Hz, 2H), 1.74 (s, 6H), 1.69 (s, 6H). ^{19}F NMR (300 MHz, d_4-CD_3OD) δ (ppm) –133.04 (t, 1F, J = 28.4). ^{13}C NMR (500 MHz, d_4-CD_3OD): δ (ppm) 174.43, 172.82, 153.00, 151.04, 142.74, 142.29, 141.48, 140.94, 136.70, 136.60, 135.63, 135.54, 128.51, 128.39, 125.43, 125.13, 122.08, 121.96, 110.95, 110.91, 96.95, 96.89, 95.80, 49.47, 49.42, 43.07, 30.44, 26.18, 26.03, 22.69, 17.94 (Figures S52–S56). HRMS (ESI$^+$) m/z calcd for $[C_{29}H_{34}FN_2O_3S]^+$ [M + H]$^+$ 509.2269, found 509.2266. HRMS (ESI$^+$) m/z calcd for $[C_{35}H_{49}FN_3O_3S]^+$ [M – I]$^+$ 610.3473, found 610.3472. Optical properties (in MeOH): λ_{max}(abs) = 656 nm (ε_{max} = 1.72 × 10^5 mol^{-1} dm^3 cm^{-1}), λ_{max}(em) = 679 nm (Φ_F = 0.09).

2-((1*E*,3*Z*)-3-Fluoro-5-((*E*)-1,3,3-trimethylindolin-2-ylidene)penta-1,3-dien-1-yl)-1,1,3-trimethyl-1*H*-benzo[e]indol-3-ium iodide (2k). The compound was prepared from **2c** and heterocycle **6B** according to a general procedure and purified by flash column chromatography (CH_2Cl_2/CH_3OH = gradually from 20 : 1 to 3 : 1). Yield: 10 mg (20%). Blue solid. ^1H NMR (300 MHz, d_4–CD_3OD): δ (ppm) 8.26 (d, J = 8.5, 2H), 8.04 (dd, J = 14.2, 8.5 4H), 7.92 (dd, J = 28.5, 14.2, 2H), 7.70–7.60 (m, 4H), 7.52 (m, 2H), 6.35 (d, J = 14.2, 2H) 3.80 (s, 6H), 2.04 (s, 12H). ^{19}F NMR (300 MHz, d_4-CD_3OD) δ (ppm) –133.77 (t, 1F, J = 28.5). ^{13}C NMR (500 MHz, d_4-CD_3OD: δ (ppm) 175.78, 173.20, 152.87, 150.92, 142.88, 141.13, 140.09, 135.62, 135.53, 134.83, 134.74, 134.20, 132.38, 130.42, 129.75, 128.38, 127.89, 127.47, 125.05, 125.00, 122.06, 121.91, 110.75, 110.51, 96.67, 96.62, 95.85, 95.79, 51.33, 49.11, 48.44, 30.85, 30.17, 26.15, 25.70 (Figures S57–S59). HRMS (ESI$^+$) *m/z* calcd for $[C_{31}H_{32}FN_2]^+$ $[M-I]^+$ 451.2544, found 451.2549. Optical properties (in MeOH): λ_{max}(abs) = 672 nm (ε_{max} = 1.37 × 10^5 mol^{-1} dm^3 cm^{-1}), λ_{max}(em) = 706 nm (Φ_F = 0.04).

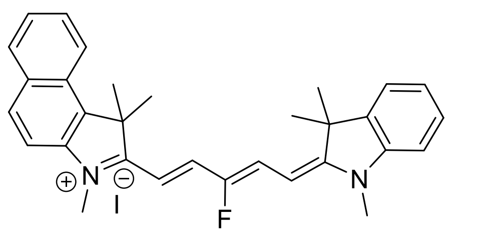

2-((1*E*,3*Z*,5*E*)-3-Fluoro-5-(1,1,3-trimethyl-1,3-dihydro-2*H*-benzo[e]indol-2-ylidene)penta-1,3-dien-1-yl)-1,1,3-trimethyl-1*H*-benzo[e]indol-3-ium iodide (2l). The compound was prepared from **2c** and heterocyclic salt **6B** according to a general procedure and purified by flash column chromatography (CH_2Cl_2/CH_3OH = gradually from 20 : 1 to 3 : 1). Yield: 25 mg (38%). Blue solid. ^1H NMR (300 MHz, d_4–CD_3OD): δ (ppm) 8.27 (m, 2H), 8.04 (m, 4H), 7.94 (dd, J = 14.1, 2H), 7.68 (m, 4H), 7.52 (m, 2H), 6.36 (d, J = 14.1, 2H) 3.81 (s, 6H), 2.05 (s, 12H). ^{19}F NMR (300 MHz, d_4-CD_3OD) δ (ppm) –133.83 (t, 1F, J = 28.4). ^{13}C NMR (500 MHz, d_4-CD_3OD): δ (ppm) 175.08, 140.20, 134.73, 134.63, 133.88, 132.27, 130.38, 129.75, 127.96, 127.42, 124.90, 122.01, 110.65, 96.09, 96.04, 51.14, 30.68, 25.78 (Figures S60–S62). HRMS (ESI$^+$) *m/z* calcd for $[C_{35}H_{34}FN_2]^+$ $[M-I]^+$ 501.2701, found 501.2706. Optical properties (in MeOH): λ_{max}(abs) = 692 nm (ε_{max} = 1.17 × 10^5 mol^{-1} dm^3 cm^{-1}), λ_{max}(em) = 723 nm (Φ_F = 0.05).

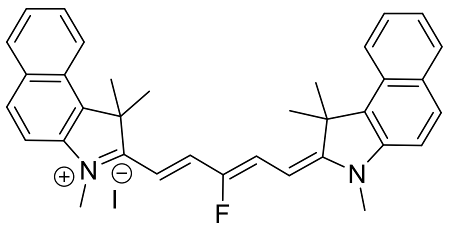

4-((1*E*,3*Z*)-3-Fluoro-5-((*E*)-1,3,3-trimethylindolin-2-ylidene)penta-1,3-dien-1-yl)-1-methylquinolin-1-ium iodide (2m). The pentamethine cyanine dye **2c** (1 eq; 18–72 mM), 1,4-dimethylquinolinium iodide (5 eq) and diisopropylamine (10 eq) were dissolved in acetonitrile, and the mixture was stirred at 80 °C for 17 h. Compound was purified by flash reverse column chromatography (0.01% TFA in water/ACN = gradually from 99 : 1 to 1 : 99). Yield: 12 mg (65%). Blue solid ^1H NMR (300 MHz, d_4–CD_3OD): δ (ppm) 8.64 (dd, J = 17.1, 7.7, 2H), 8.25–8.08 (m, 2H), 8.01 (d. J = 6.8, 1H) 7.95–7.67 (m, 2H), 7.41–7.22 (m, 3H), 7.17–6.93 (m, 3H), 2H), 5.85 (d, J = 13.1, 1H), 4.40 (s, 3H), 3.40 (s, 3H), 1.67 (s, 6H). ^{19}F NMR (300 MHz, d_4-CD_3OD) δ (ppm) –132.72 (dd, 1F, J = 30.0, 28.3). ^{13}C NMR (500 MHz, d_4-CD_3OD): δ (ppm) 165.46, 153.34, 152.54, 151.38, 145.19, 143.99, 139.55, 139.31, 134.35, 133.84, 133.71, 128.22, 127.88, 125.98, 125.33, 123.65, 123.56, 121.94, 121.46, 118.00, 112.87, 109.90, 109.87, 107.84, 90.43, 90.38, 42.94, 26.88 (Figures S63-S65). HRMS (ESI$^+$) *m/z* calcd for $[C_{26}H_{26}FN_2]^+$ $[M-CF_3COO]^+$ 385.2075, found 385.2078. Optical properties (in MeOH): λ_{max}(abs) = 700 nm (ε_{max} = 0.27 × 10^5 mol^{-1} dm^3 cm^{-1}), λ_{max}(em) = 775 nm (Φ_F = 0.02).

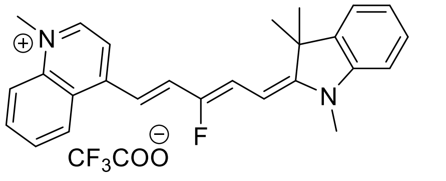

Kinetic Measurements

Kinetic measurements of chain-shortening reactions were performed by taking aliquots from the reaction mixture for HPLC analyses. The kinetic curves were fitted globally using a non-linear least squares algorithm (a Trust Region Reflective method) with the desired kinetic model. The fittings routines were programmed in Python utilizing LMFIT[9] and Scipy[10] packages. The amplitudes of the products (**2c** and **3a**) were allowed to vary during the fitting process because their yields are not quantitative. For the first experiment (Figure S3a), a second-order reaction between **1c** and **4A** was assumed to produce both **2c** and **3a**. The rate constant was determined to be $k_2 = (1.7 \pm 0.1) \times 10^{-3}\ M^{-1}\ s^{-1}$. An analogous process was assumed in Figure S3b, for which **4B** was used instead of **4A** to give $k_2 = (2.0 \pm 0.2) \times 10^{-4}\ M^{-1}\ s^{-1}$. In the last experiment (Figure S3c), it was assumed that **1c** reacts with DIPA under the pseudo-first-order kinetics (DIPA is in excess) to form indoline **4A** and a hemicyanine, which then decomposes to give various products. **1c** is formed by the same second-order process as that in Figure S3a. However, because we detected **4A** only in trace amounts during the course of the reaction, we assumed that **4A** quickly reacts with **1c** to produce **2c**. Therefore, the experimental data of this experiment (Figure S3c) were fitted under the pseudo-first-order kinetics, which corresponds to the formation of indoline (**4A**) and hemicyanine from **1c**. The rate constant was determined to be $k_1 = (5.7 \pm 0.7) \times 10^{-6}\ s^{-1}$.

(a)

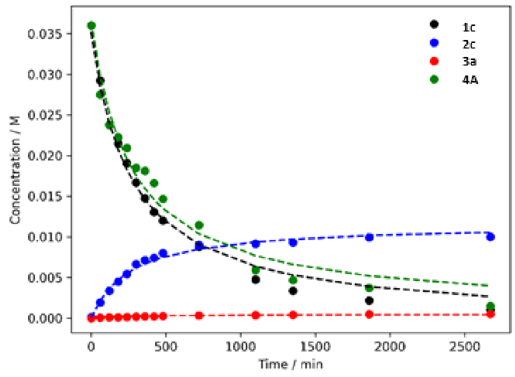

(b)

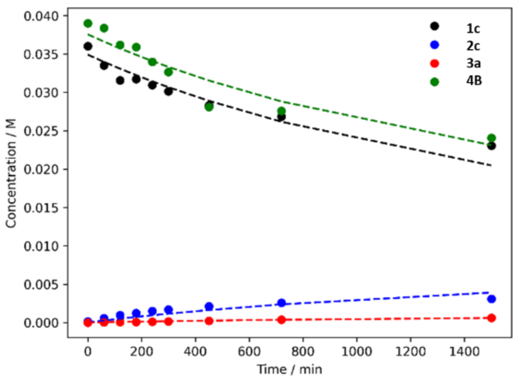

(c)

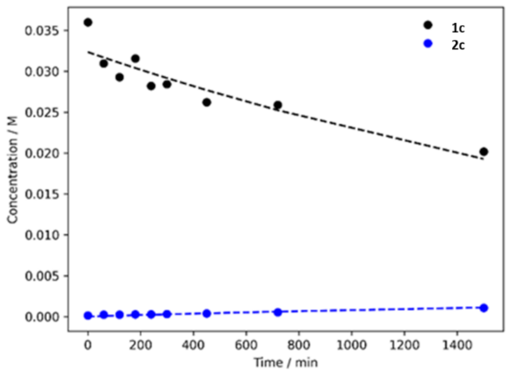

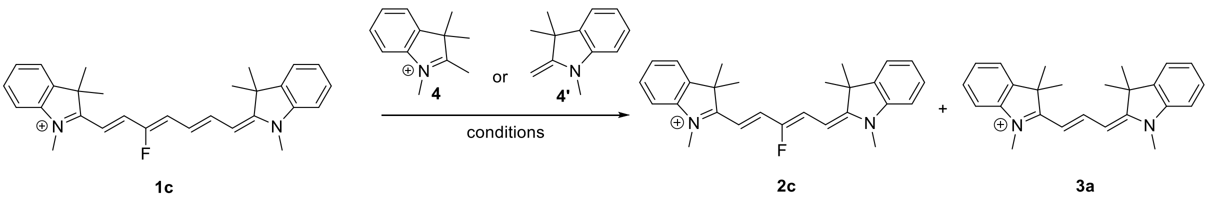

Figure S3. Dotted line: Kinetic curves for the chain shortening reaction. Dashed line: kinetic curves fitted globally using a non-linear least squares algorithm. (a) Cyanine **1c** (36 mM) reacting with indolinium iodide (**4A**, 1 eq) and DIPA (2.5 eq) at 50 °C in acetonitrile; (b) cyanine **1c** (36 mM) reacting with Fisher base (**4B**, 1 eq) at 50 °C in acetonitrile; (c) cyanine **1c** (36 mM) reacting with DIPA (2.5 eq) at 50 °C in acetonitrile. The fitted curves are displayed as dashed lines.

HPLC and HRMS data

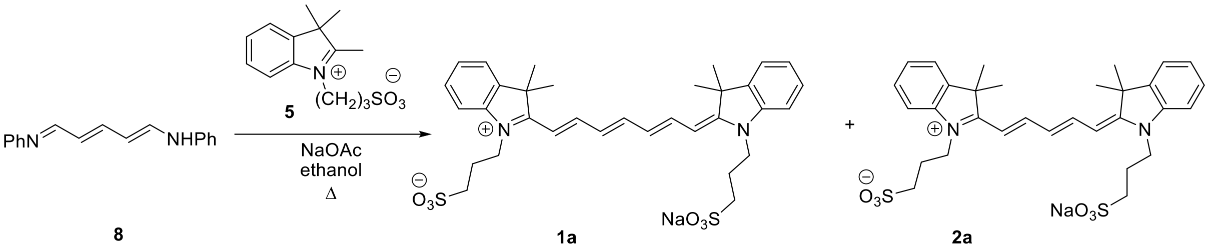

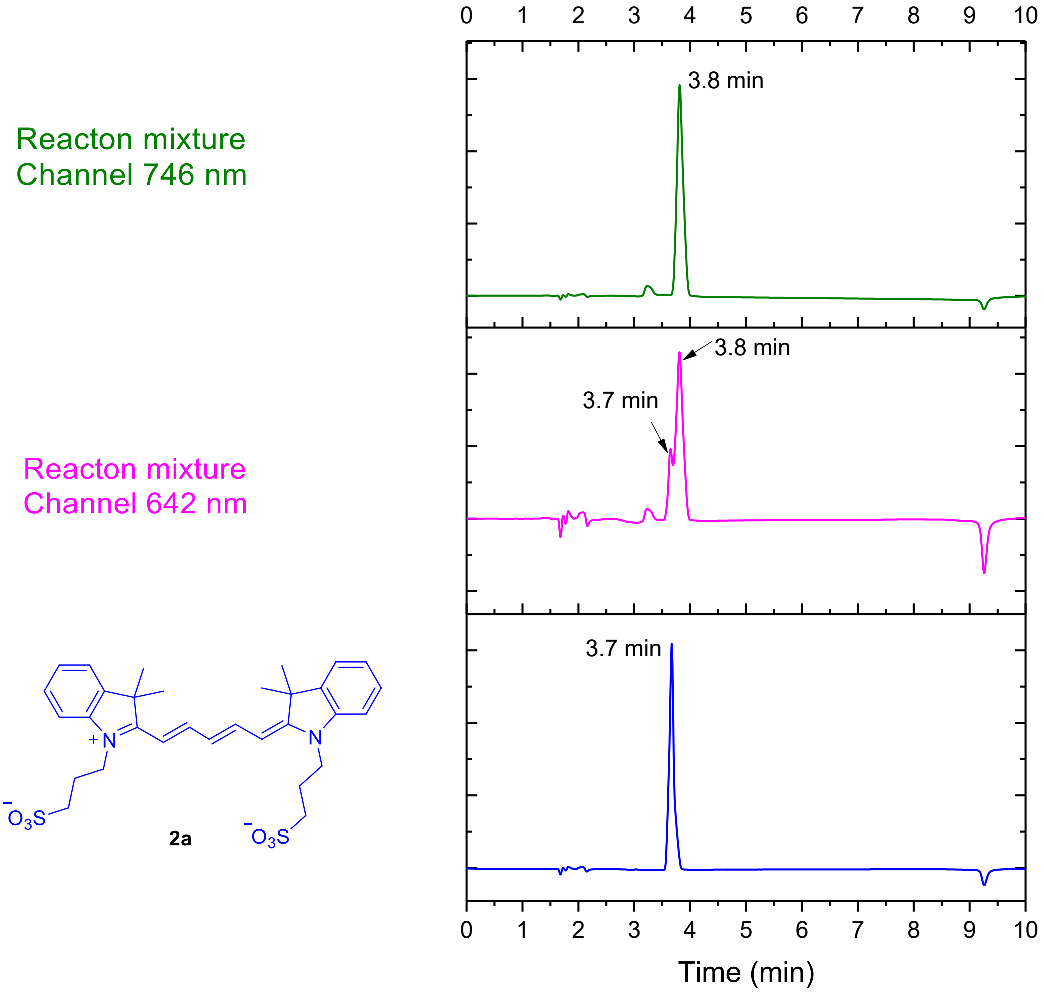

Figure S4. HPLC chromatograms: A reaction mixture of **8** and **5** after stirring at 80 °C for 24 h in ethanol (green solid: 746 nm channel, retention time 3.8 min corresponds to product **1a**, magenta solid: 642 nm channel, retention times 3.7 min corresponds to **2a**, 3.8 min corresponds to **1a**); an independently prepared analytical standard **2a** (blue solid: 642 nm channel, retention time 3.7 min).

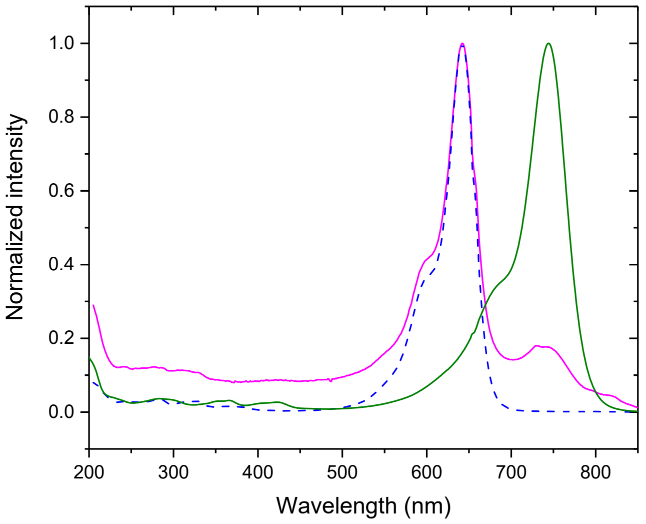

Figure S5. Green solid: absorption spectra of an HPLC peak at 3.8 min corresponding to product **1a** from a reaction mixture containing **8** and **5**. Magenta solid: absorption spectra of an HPLC peak at 3.7 min corresponding to **2a** product from a reaction mixture containing **8** and **5**. Blue dashed: absorption spectra of an HPLC peak at 3.7 min from an independently prepared analytical standard **2a**.

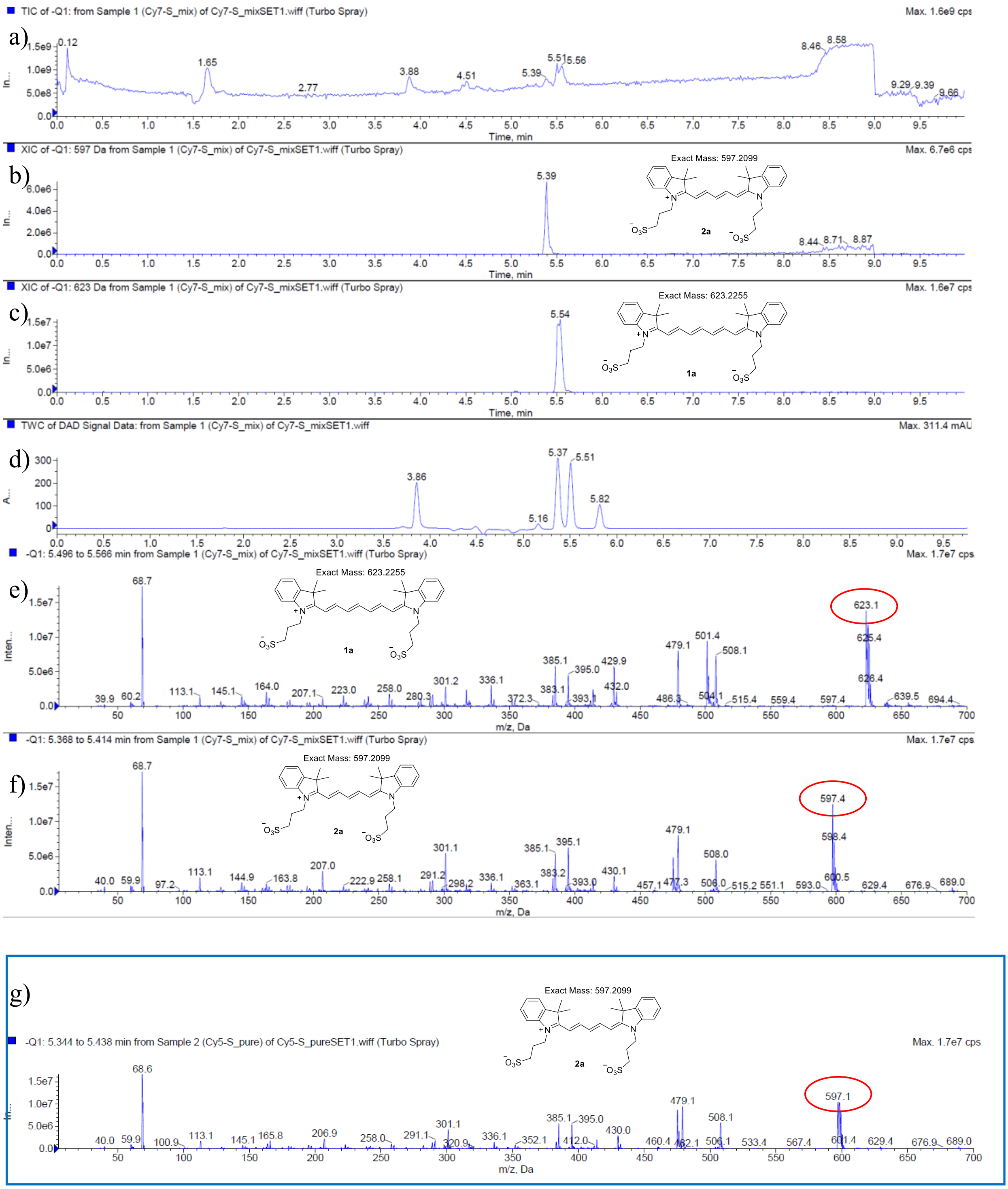

Figure S6. HPLC-MS (ESI$^-$) data of the reaction mixture of **8** and **5** after 24 h stirring at 80 °C in ethanol. a) Total ion chromatogram of the reaction mixture. b) Extracted ion chromatogram of 597 *m/z* corresponding to the **2a** exact mass. c) Extracted ion chromatogram of 623 *m/z* corresponding to the **1a** exact mass. d) Total wavelength chromatogram of the reaction mixture. e) MS spectrum of 623 *m/z* corresponding to product **1a**. f) MS spectrum of 597 *m/z* corresponding to product **2a**. g) MS spectrum of independently prepared analytical standard **2a**.

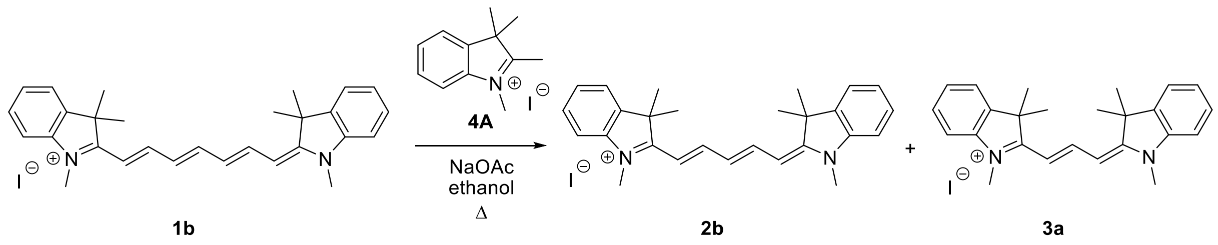

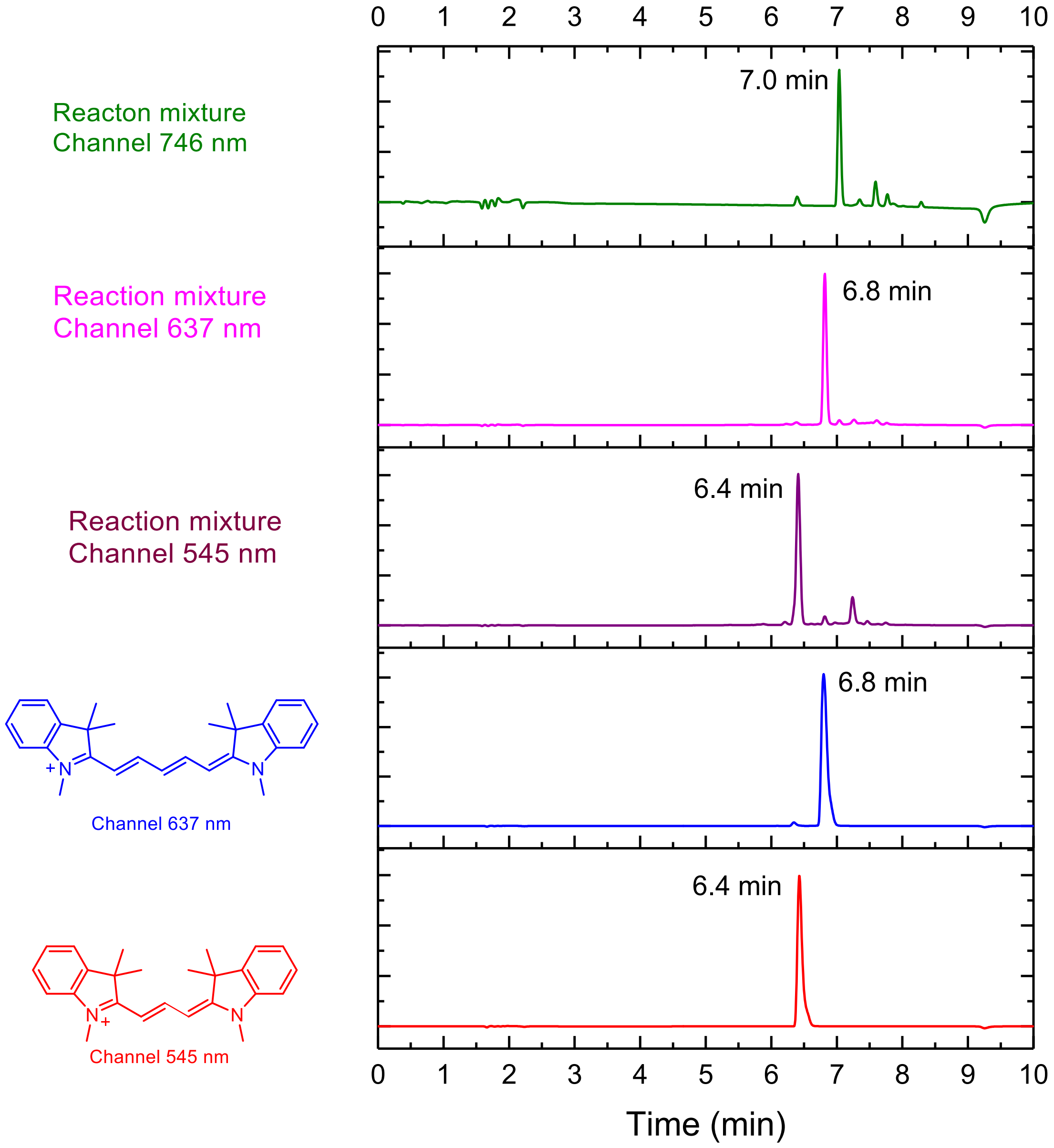

Figure S7. HPLC chromatogram: a reaction mixture of **1b** and **4A** after stirring at 80 °C for 24 h in ethanol (green solid: 746 nm channel, retention time 7.0 min corresponds to product **1b**, magenta solid: 637 nm channel, retention time 6.8 min corresponds to product **2b**, purple solid: 545 nm channel, retention time 6.4 min corresponds to product **3a**); blue solid: an independently prepared analytical standard **2b** (637 nm channel, retention time 6.8 min). Red solid: an independently prepared analytical standard **3a** (545 nm channel, retention time 6.4 min).

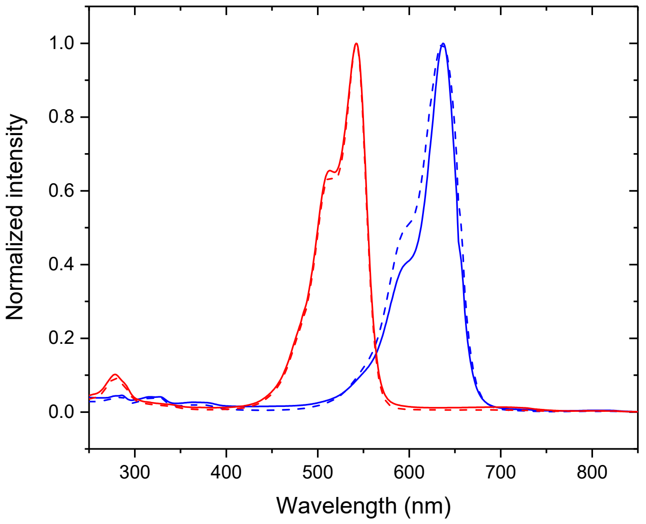

Figure S8. Blue solid: absorption spectra of an HPLC peak at 6.8 min corresponding to product **2b** from a reaction mixture containing **1b** and **4A**. Red solid: absorption spectra of an HPLC peak at 6.8 min corresponding to product **3a** from a reaction mixture containing **1b** and **4A**. Blue dashed: absorption spectra of an HPLC peak at 6.8 min from an independently prepared analytical standard **2b**. Red dashed: absorption spectra of an HPLC peak at 6.4 min from an independently prepared analytical standard **3a**.

(a)

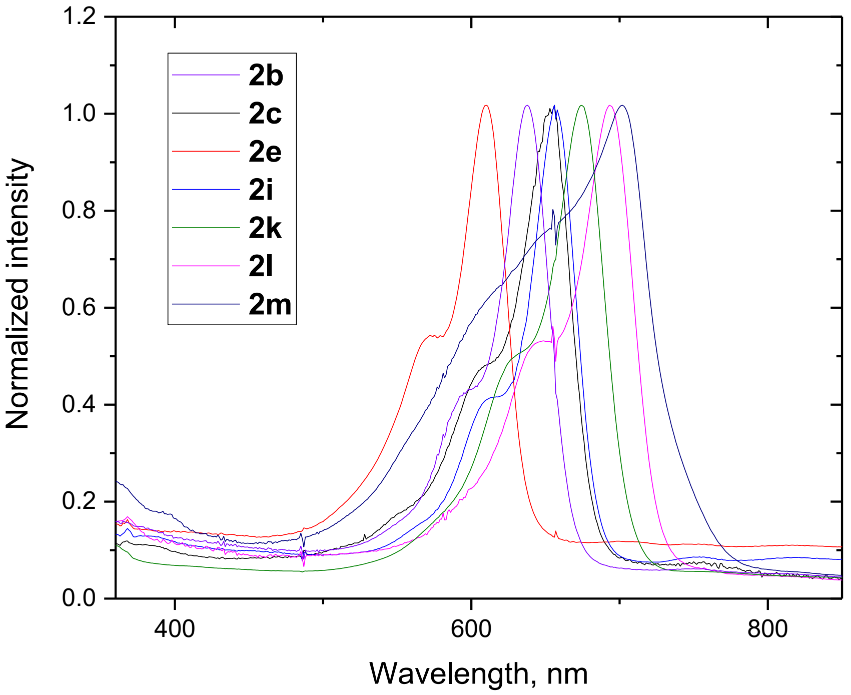

(b)

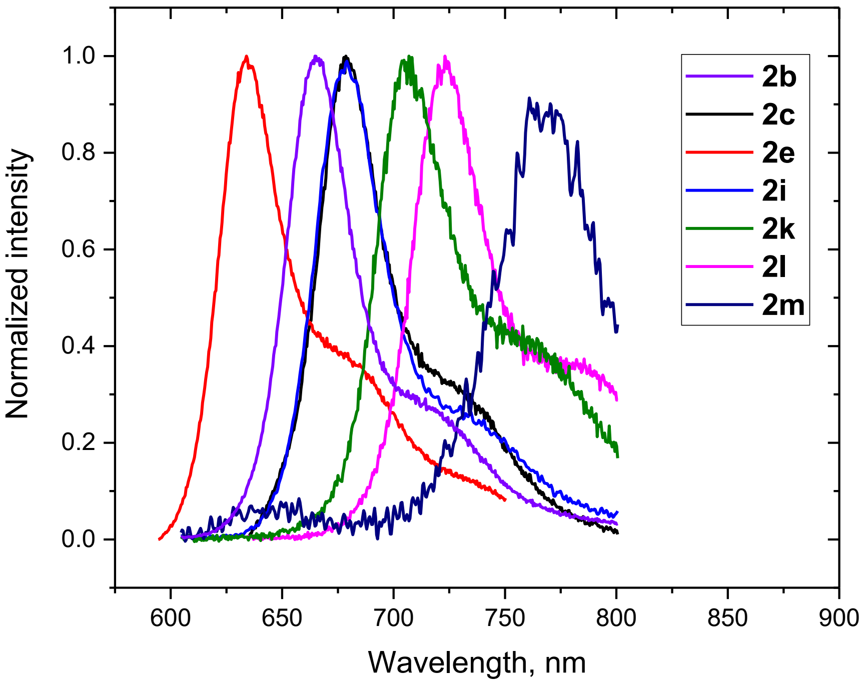

Figure S9. (a) Absorption spectra of **2b, 2c, 2e, 2i, 2k, 2l, 2m** in methanol. (b) Emission spectra of **2b, 2c, 2e, 2i, 2k, 2l, 2m** in methanol.

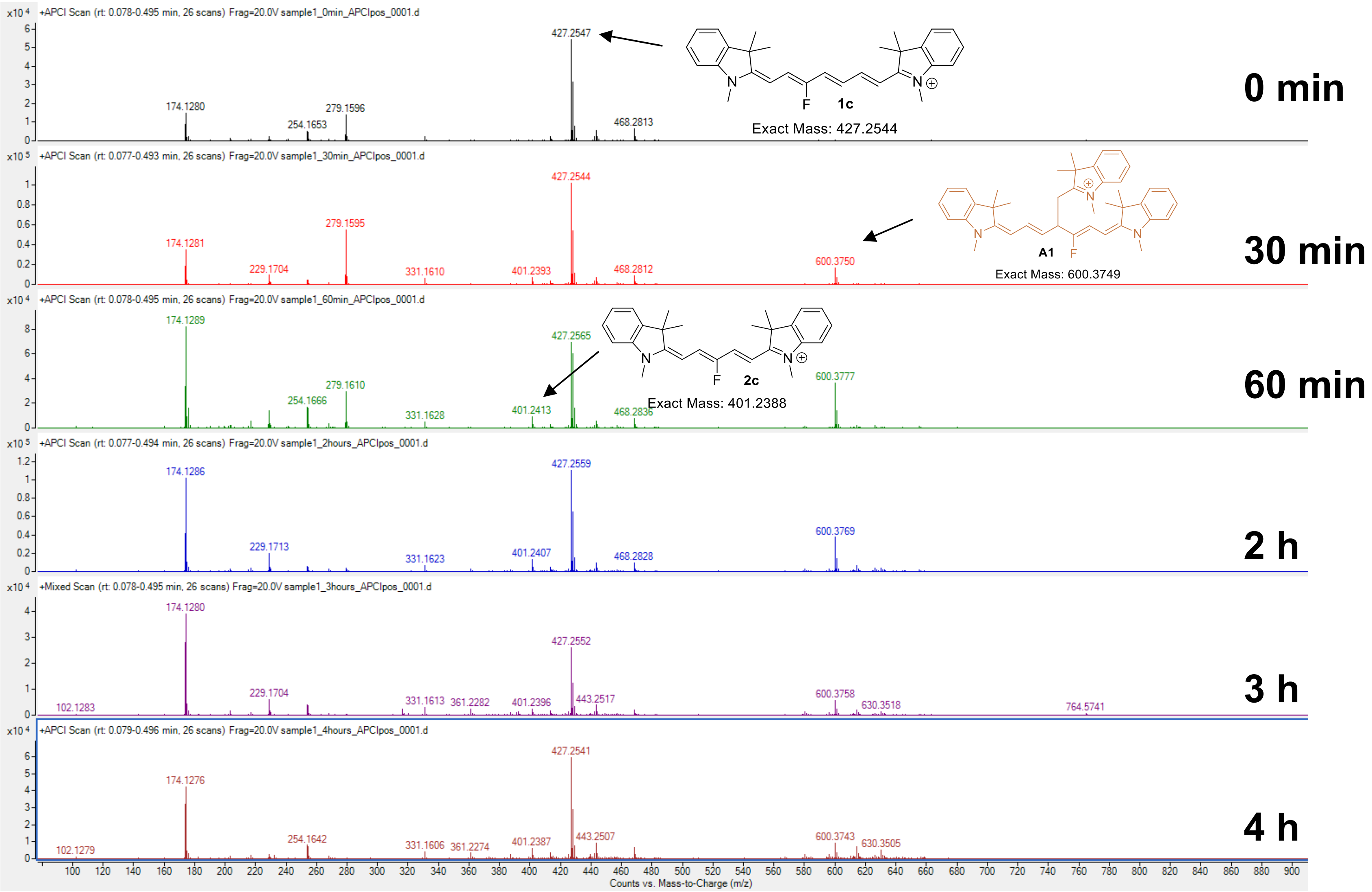

Figure S10. APCI$^+$ HRMS analysis of a chain-shortening reaction of **1c** (18mM) with indolinium iodide **4A** (1 eq), DIPA (2.5 eq) in acetonitrile at 50 °C.

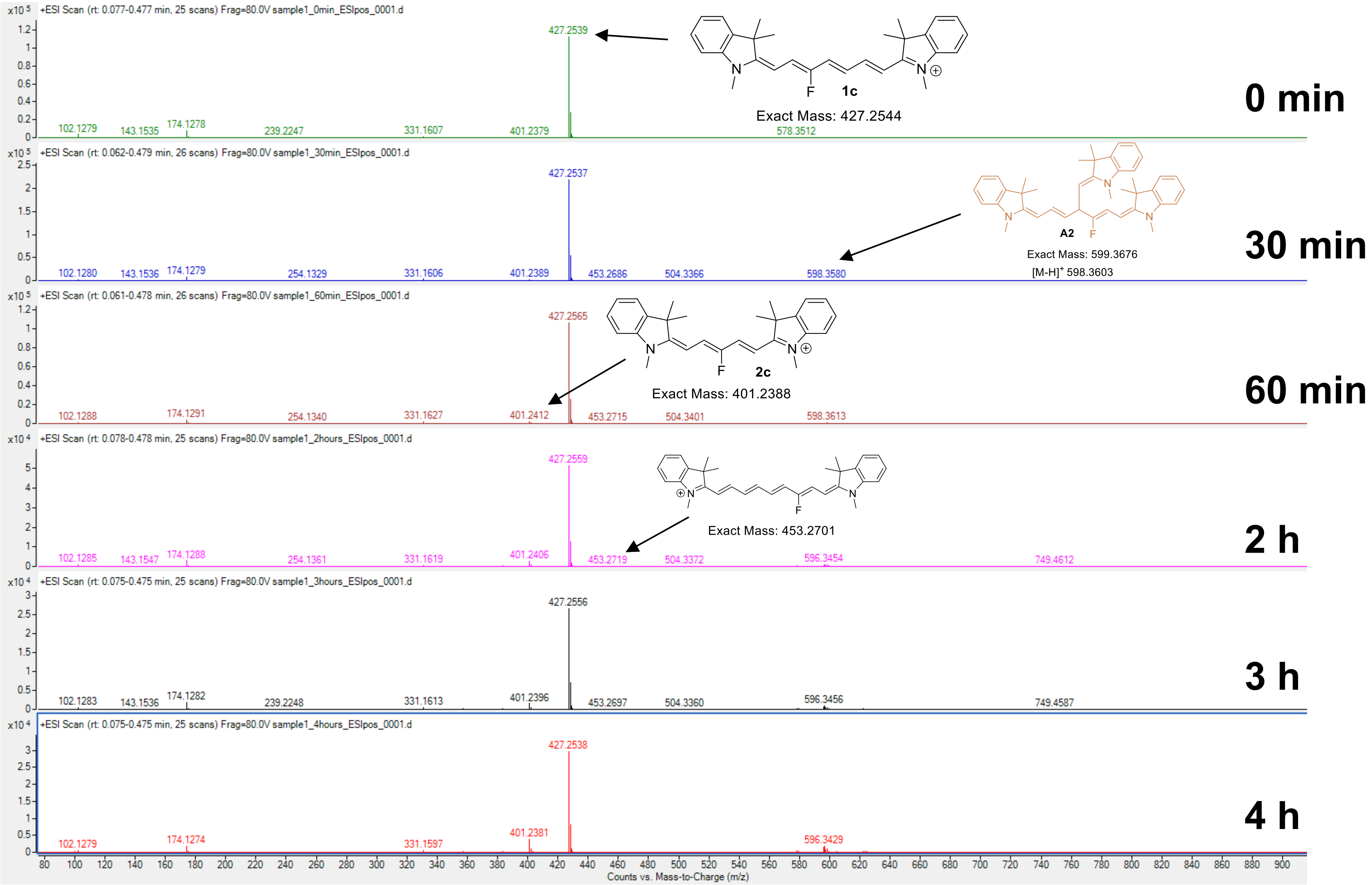

Figure S11. ESI+ HRMS analysis of the chain-shortening reaction of **1c** (18mM) with indolinium iodide **4A** (1 eq), DIPA (2.5 eq) in acetonitrile at 50 °C.

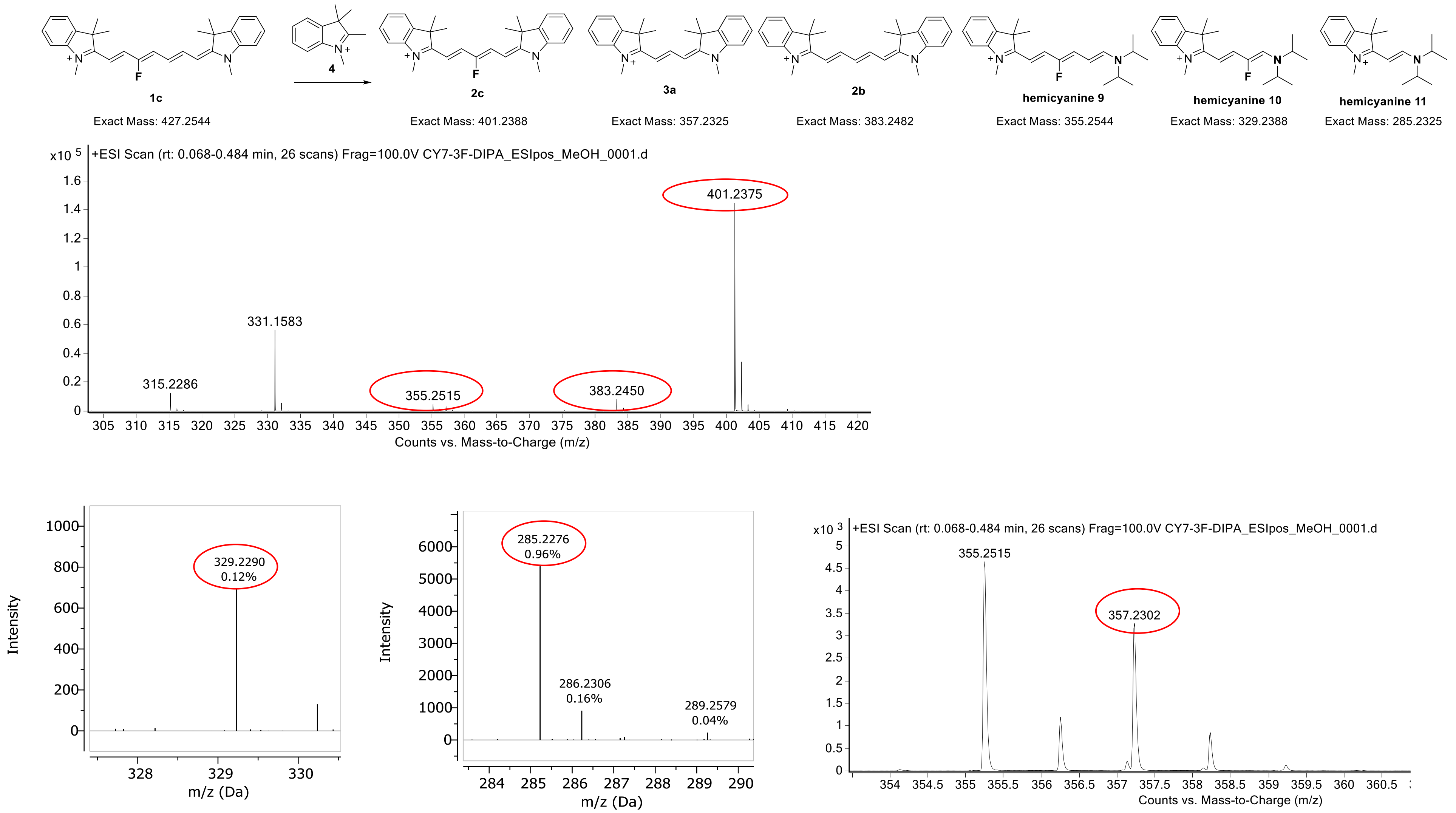

Figure S12. HRMS of the crude reaction mixture of the chain-shortening reaction of **1c** (18 mM) with indolinium iodide **4A** (1 eq) and DIPA (2.5 eq) at 50 °C in acetonitrile for 1 h.

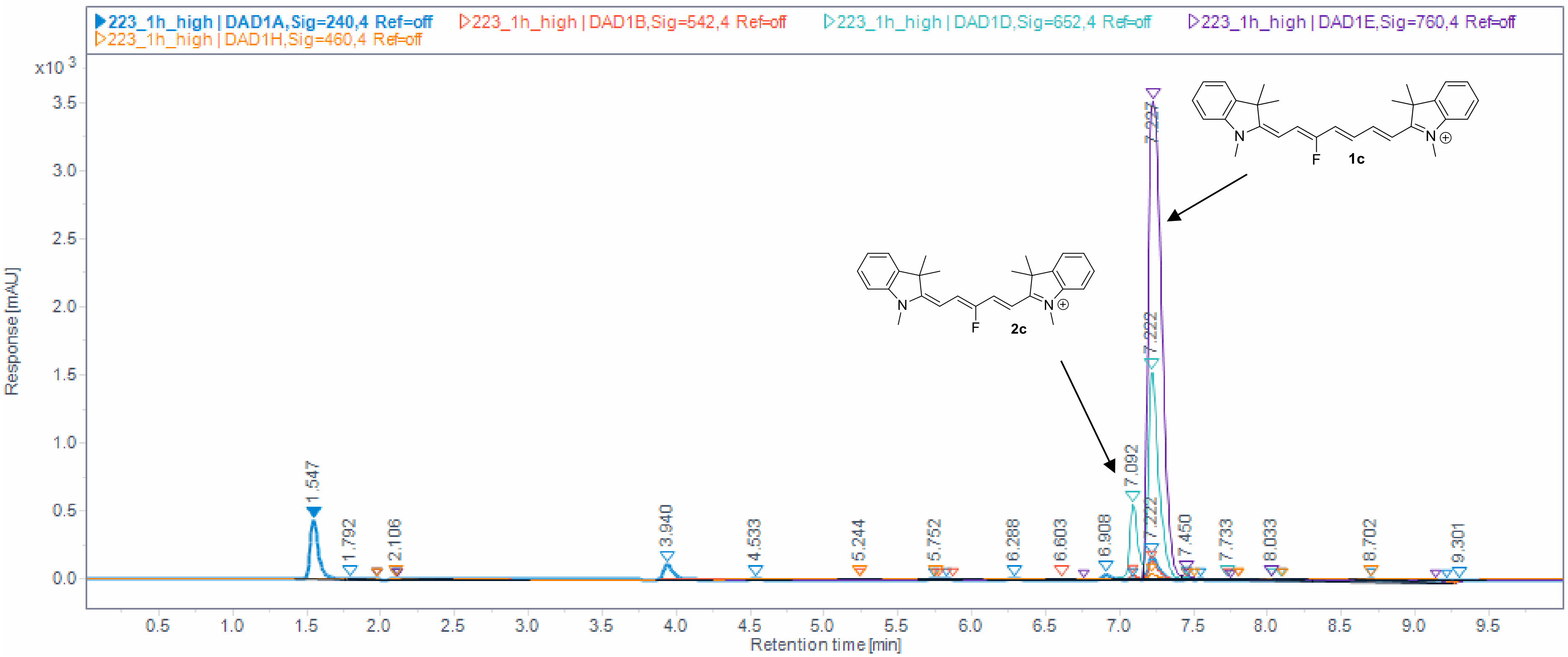

Figure S13. HPLC of the crude reaction mixture of the chain-shortening reaction of **1c** (18 mM) with indolinium iodide **4A** (1 eq) and DIPA (2.5 eq) at 50 °C in acetonitrile for 1 h.

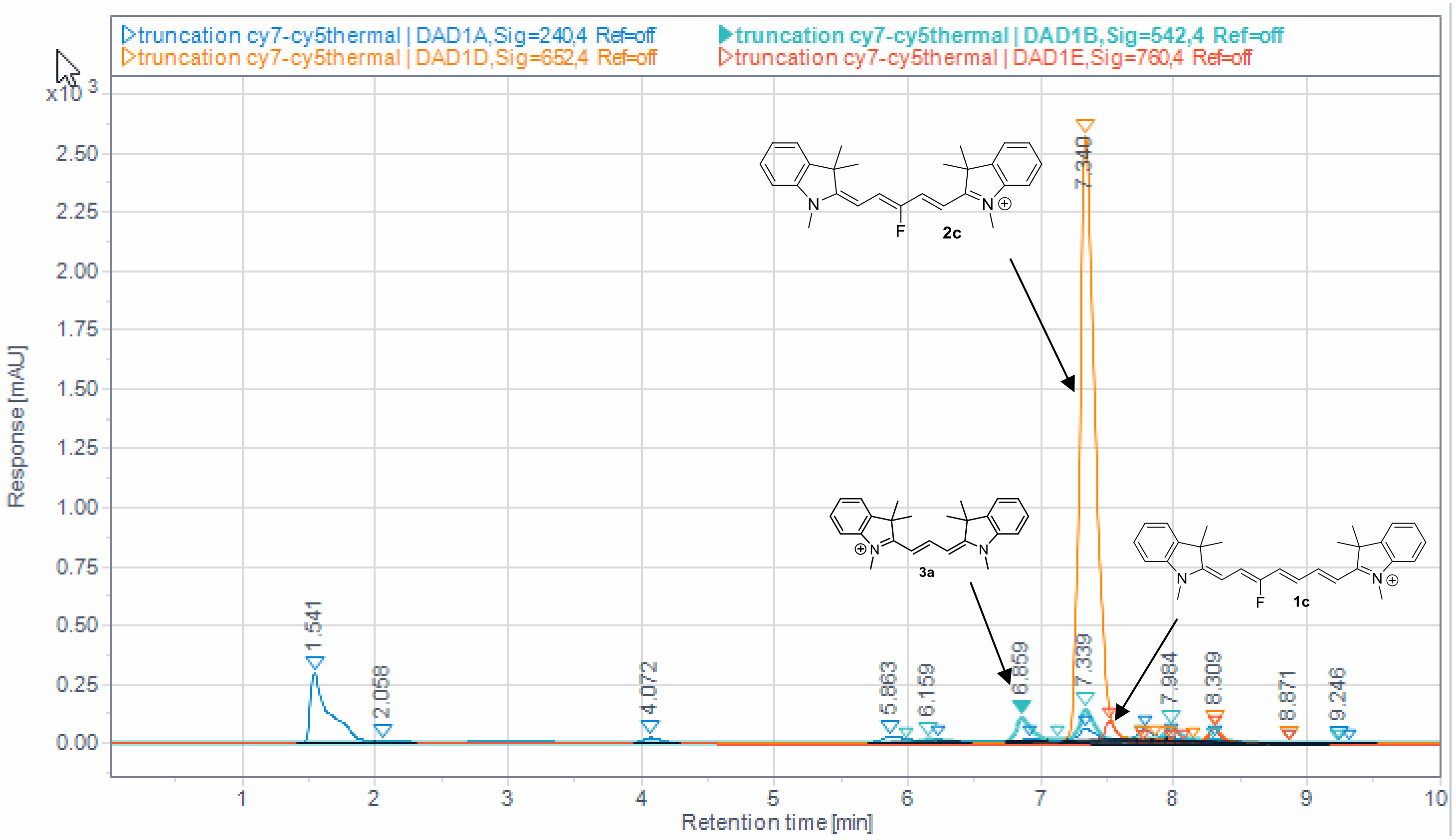

Figure S14. HPLC of the crude reaction mixture of the chain-shortening reaction of **1c** (18 mM) with indolinium iodide **4A** (1 eq) and DIPA (2.5 eq) at 50 °C in acetonitrile for 21 h.

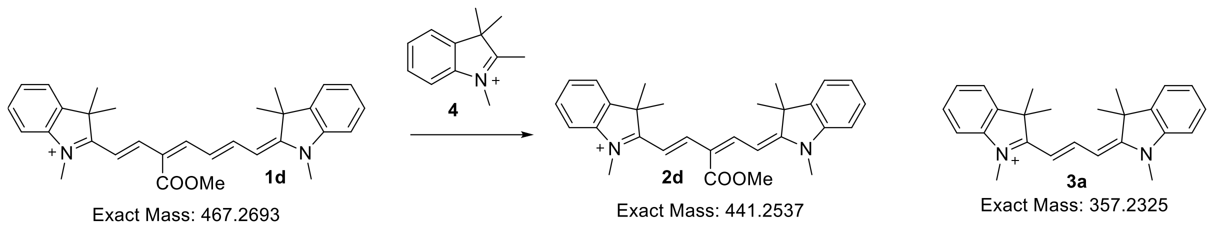

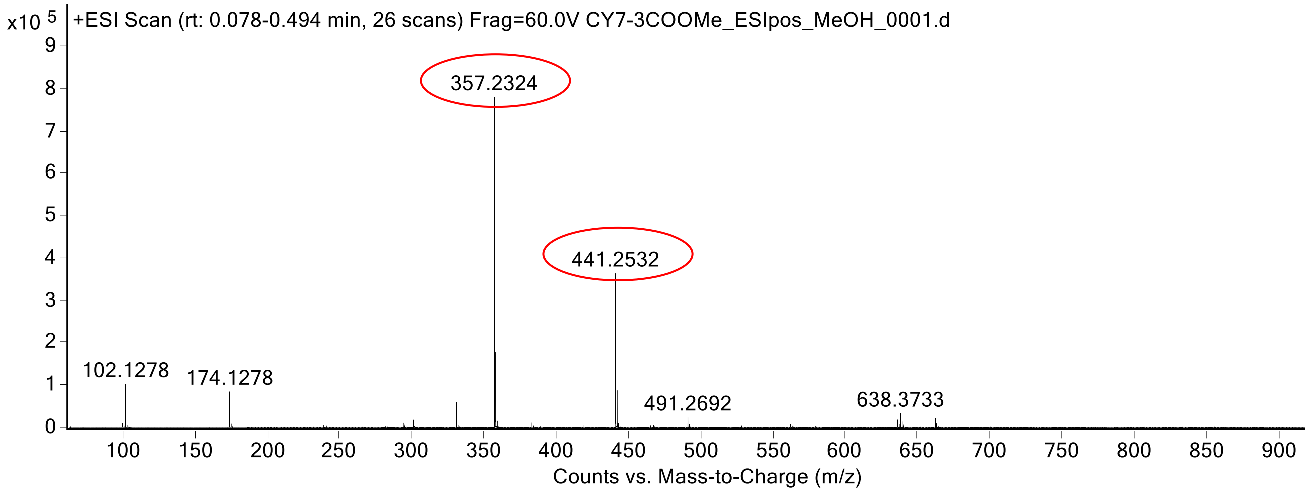

Figure S15. HRMS of the crude reaction mixture of the chain-shortening reaction of **1d** (72 mM) with indolinium iodide **4A** (1 eq) and DIPA (2.5 eq) at 50 °C in acetonitrile after 21 h.

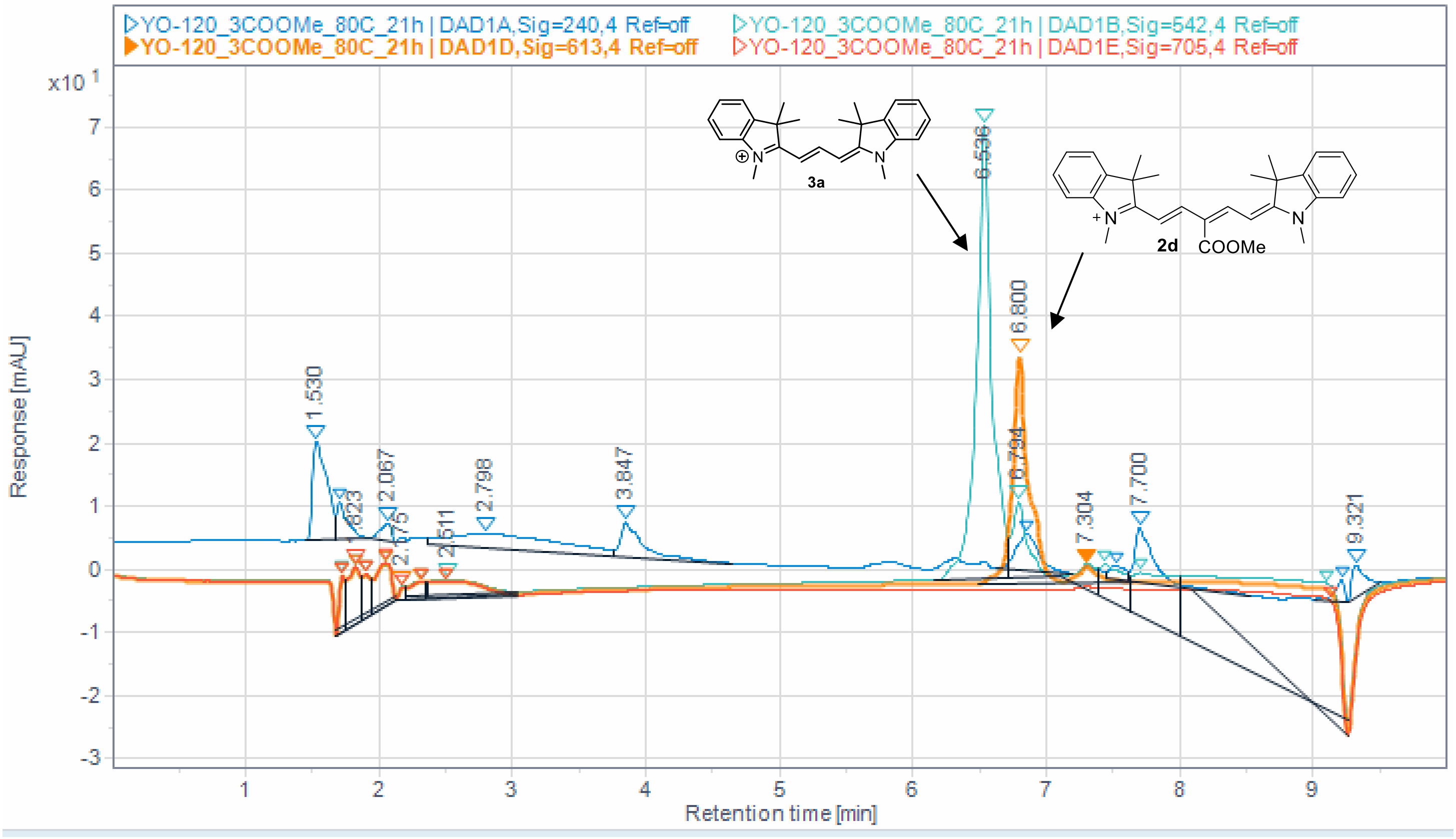

Figure S16. HPLC of the crude reaction mixture of the chain-shortening reaction of **1d** (72 mM) with indolinium iodide **4A** (1 eq) and DIPA (2.5 eq) at 80 °C in acetonitrile after 21 h.

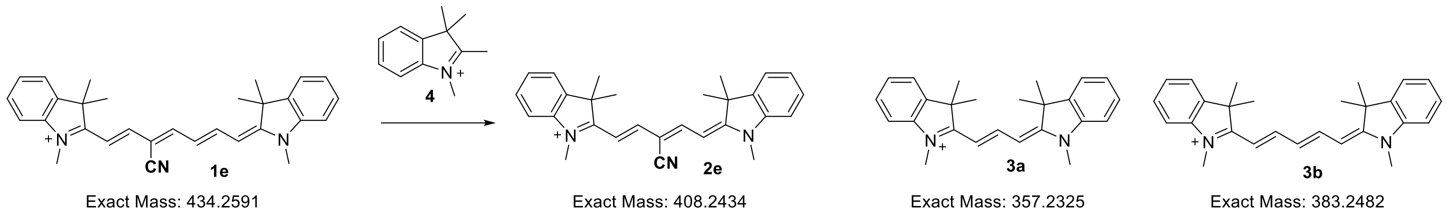

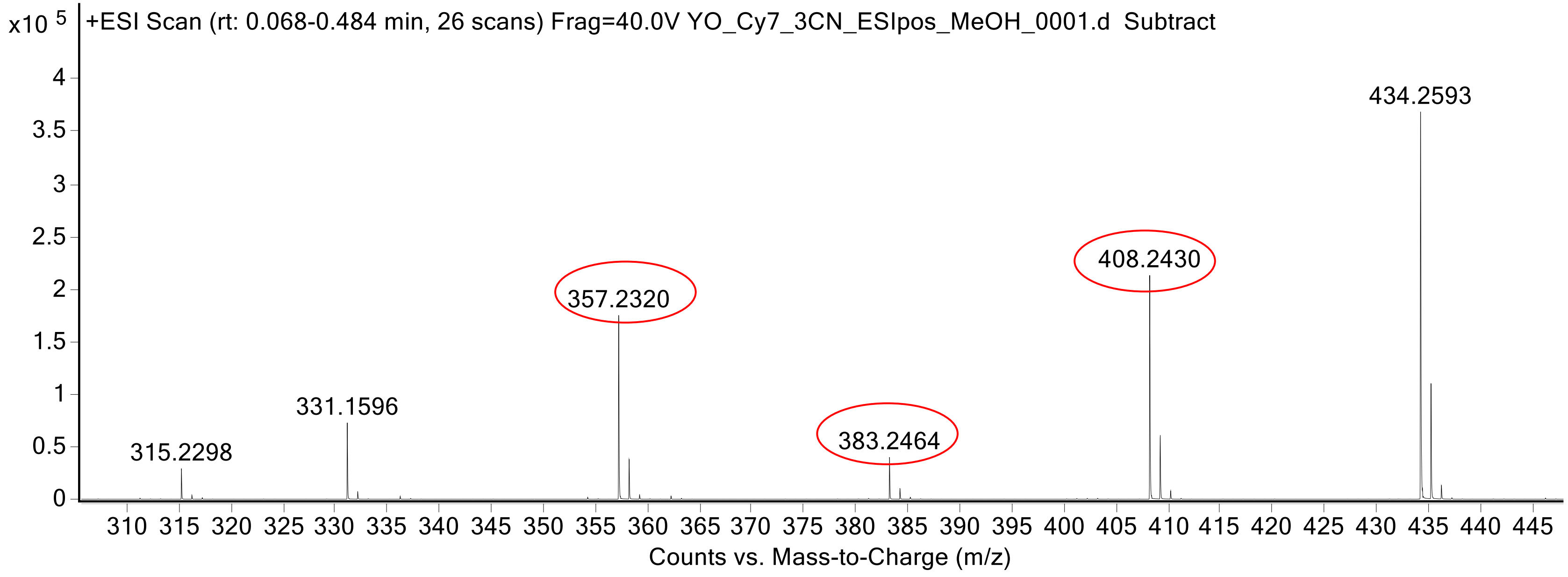

Figure S17. HRMS of the crude reaction mixture of the chain-shortening reaction of **1e** (72 mM) with indolinium iodide **4A** (1 eq) and DIPA (2.5 eq) at 50 °C in acetonitrile after 21 h.

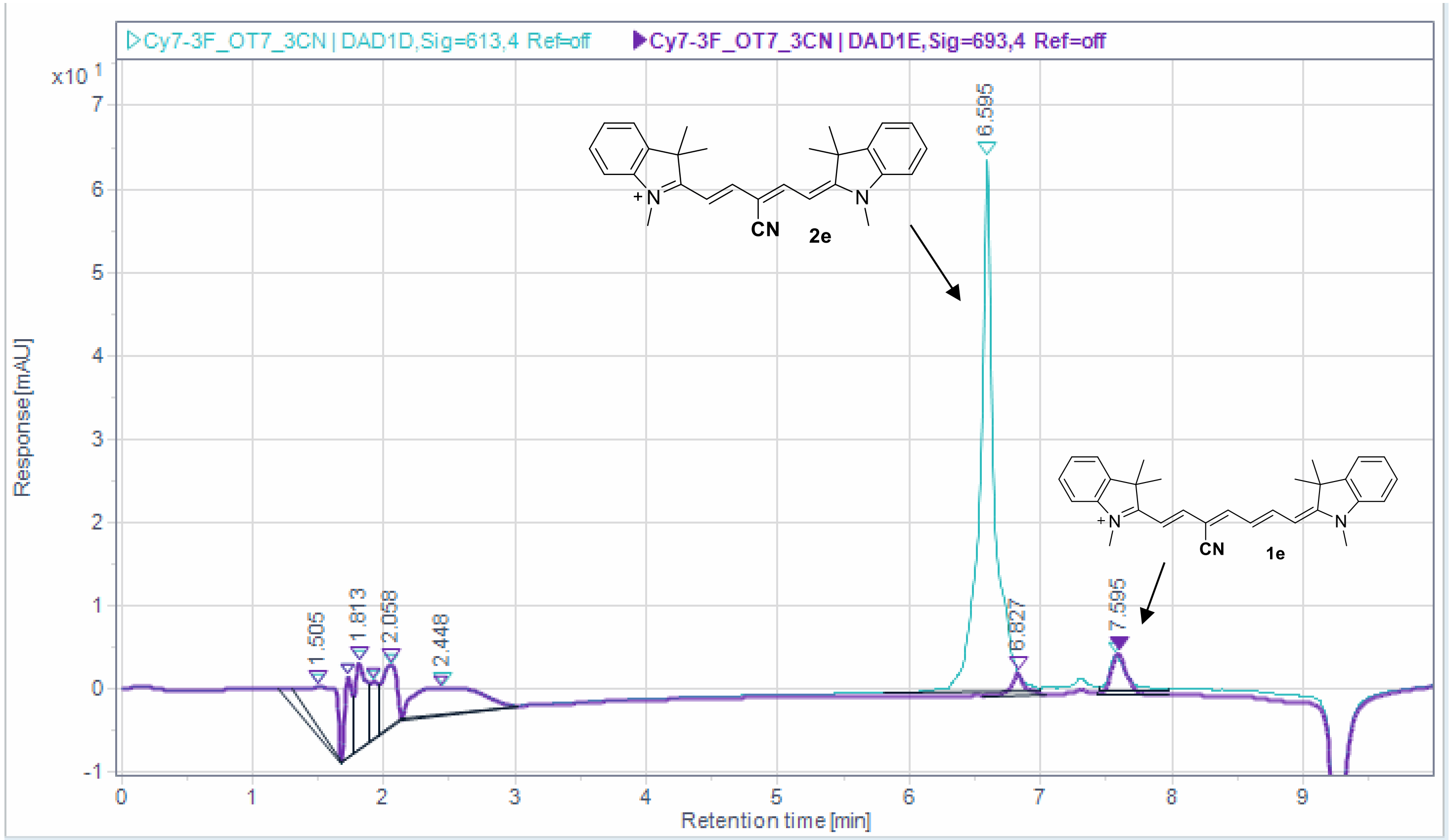

Figure S18. HPLC of the crude reaction mixture of the chain-shortening reaction of **1e** (72 mM) with indolinium iodide **4A** (1 eq) and DIPA (2.5 eq) at 50 °C in acetonitrile after 21 h.

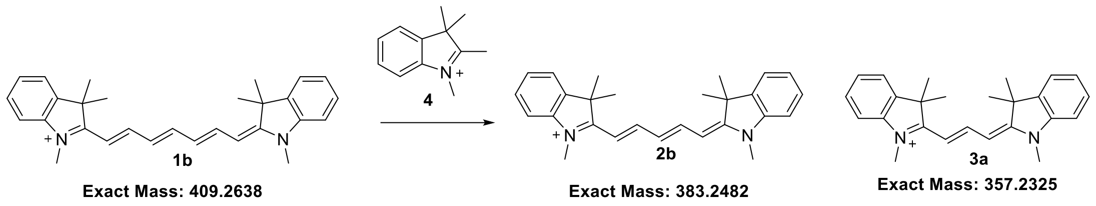

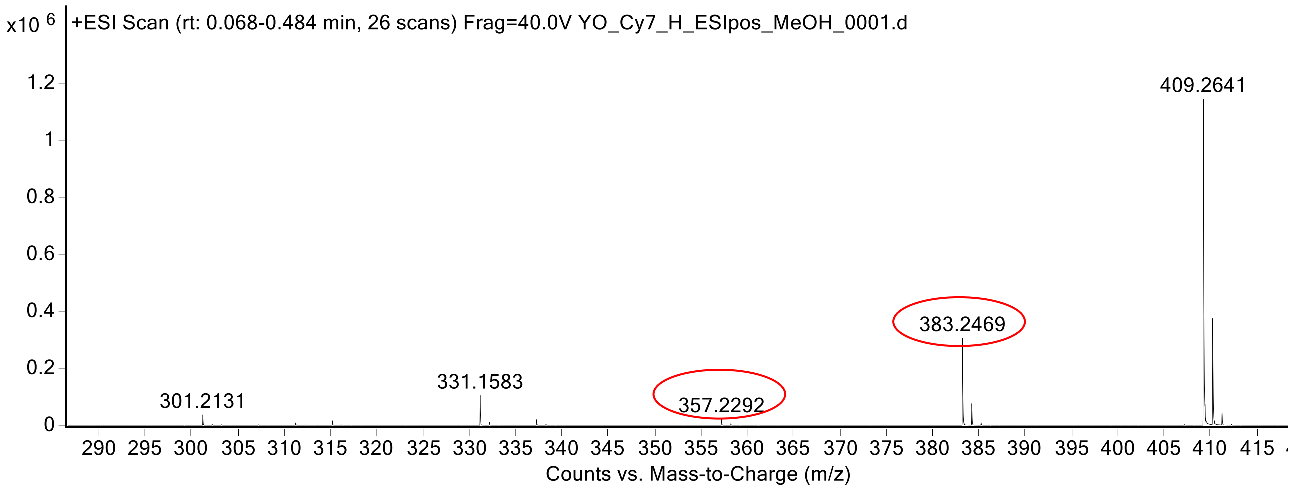

Figure S19. HRMS of the crude reaction mixture of the chain-shortening reaction of **1b** (72 mM) with indolinium iodide **4A** (1 eq) and DIPA (2.5 eq) at 50 °C in acetonitrile after 21 h.

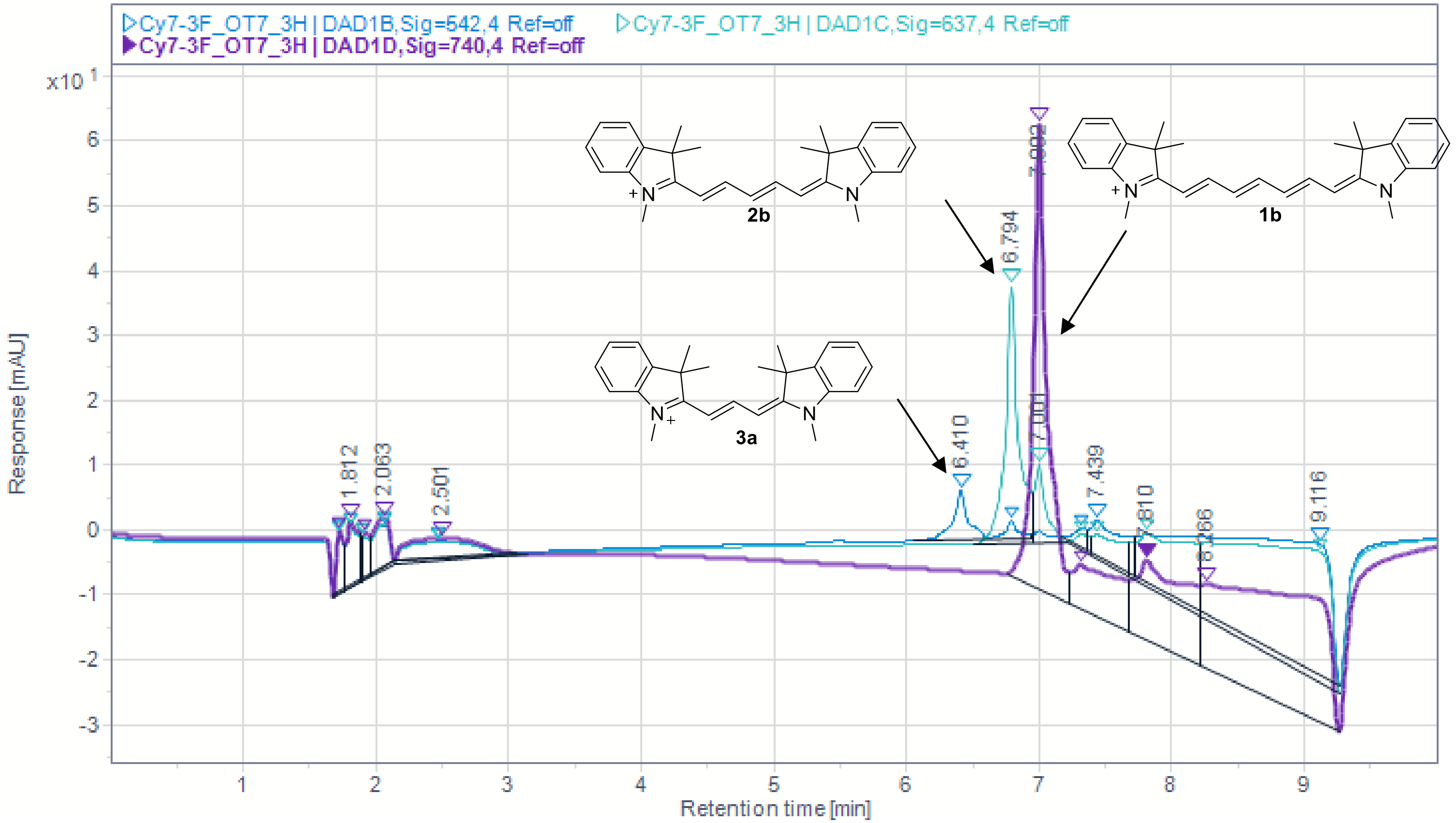

Figure S20. HPLC of the crude reaction mixture of the chain-shortening reaction of **1b** (72 mM) with indolinium iodide **4A** (1 eq) and DIPA (2.5 eq) at 50 °C in acetonitrile after 21 h.

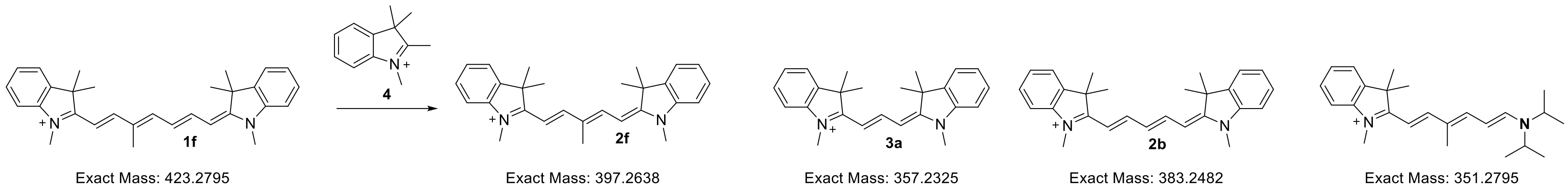

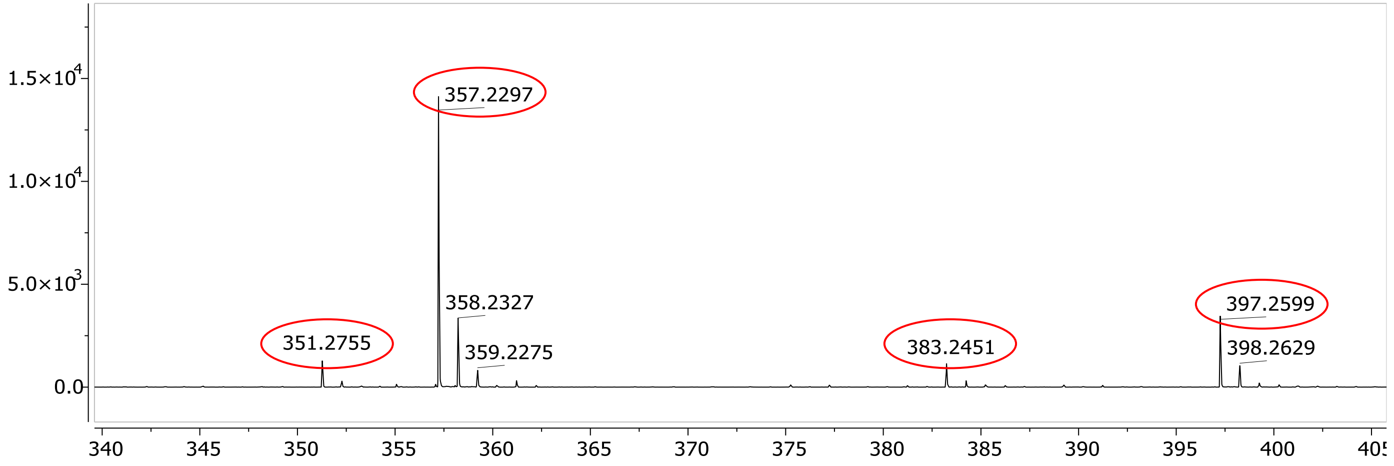

Figure S21. HRMS of the crude reaction mixture of the chain-shortening reaction of **1f** (72 mM) with indolinium iodide **4A** (1 eq) and DIPA (2.5 eq) at 50 °C in acetonitrile after 21 h.

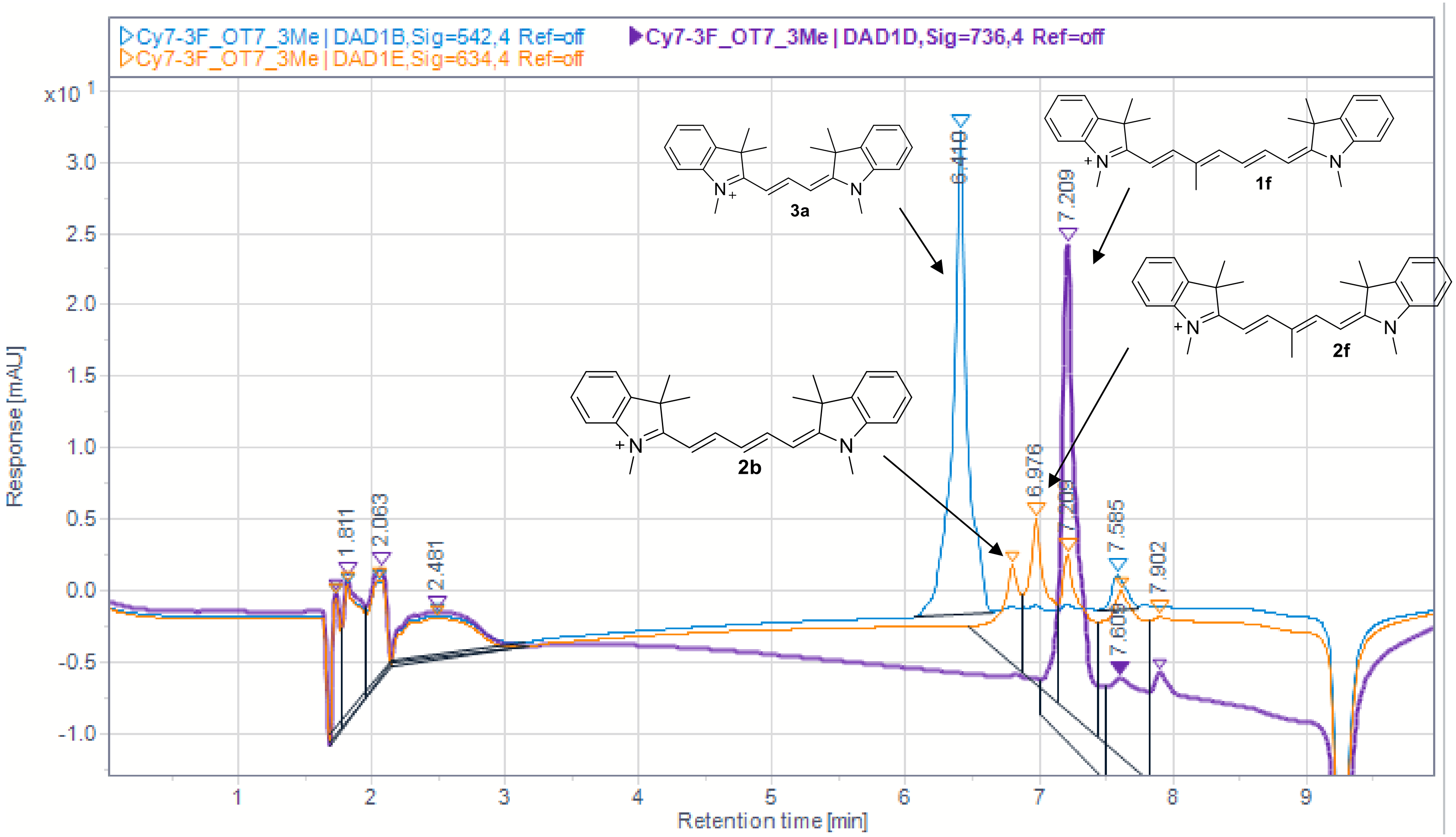

Figure S22. HPLC of the crude reaction mixture of the chain-shortening reaction of **1f** (72 mM) with indolinium iodide **4A** (1 eq) and DIPA (2.5 eq) at 50 °C in acetonitrile after 21 h.

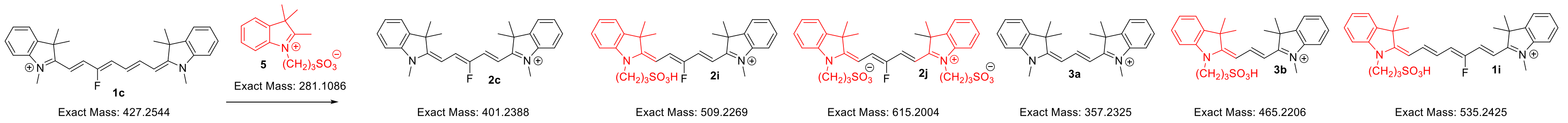

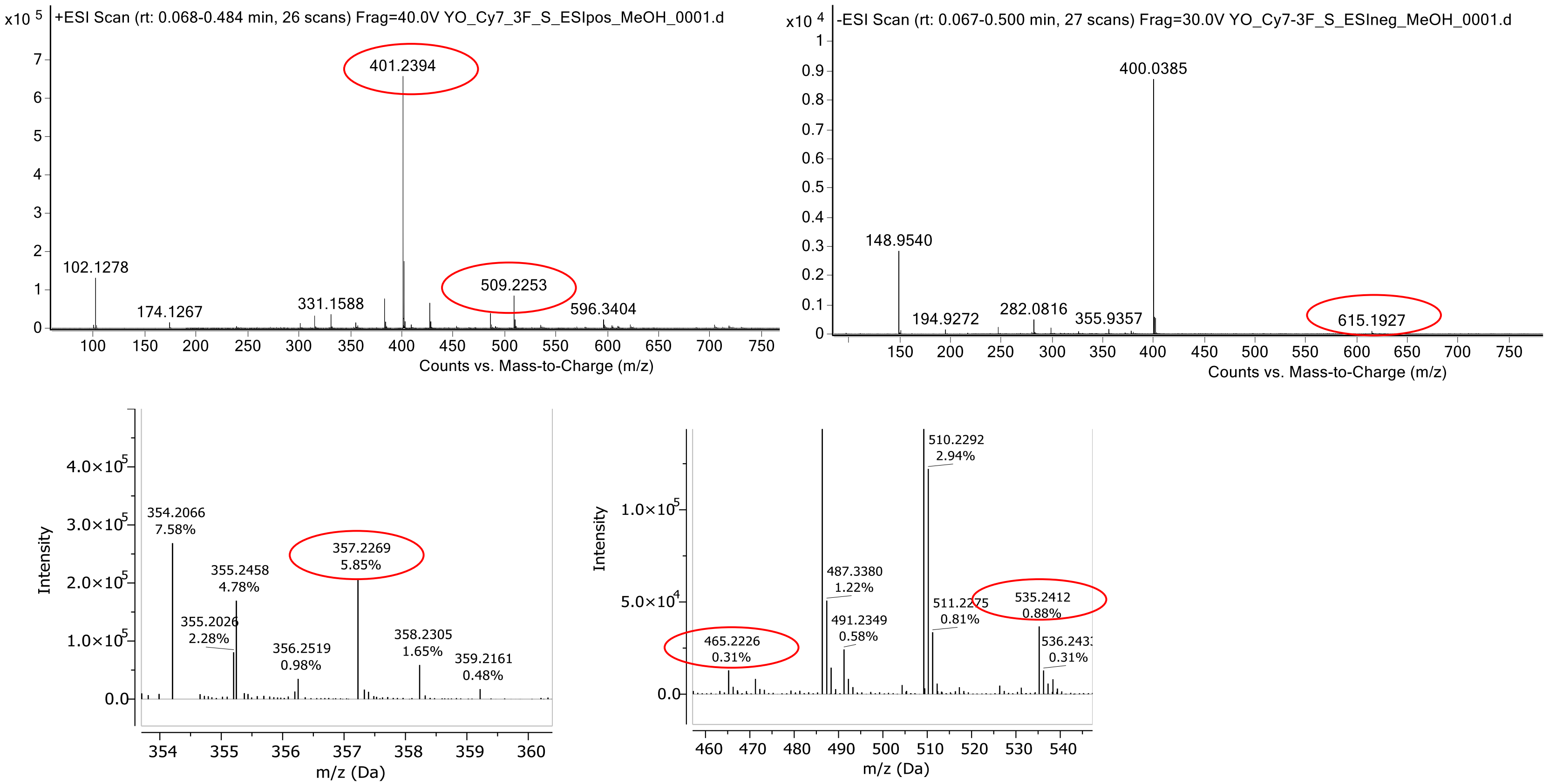

Figure S23. HRMS of the crude reaction mixture of the chain-shortening reaction of **1c** with indolinium salt **5** (1 eq) and DIPA (2.5 eq) at 50 °C in acetonitrile after 21 h.

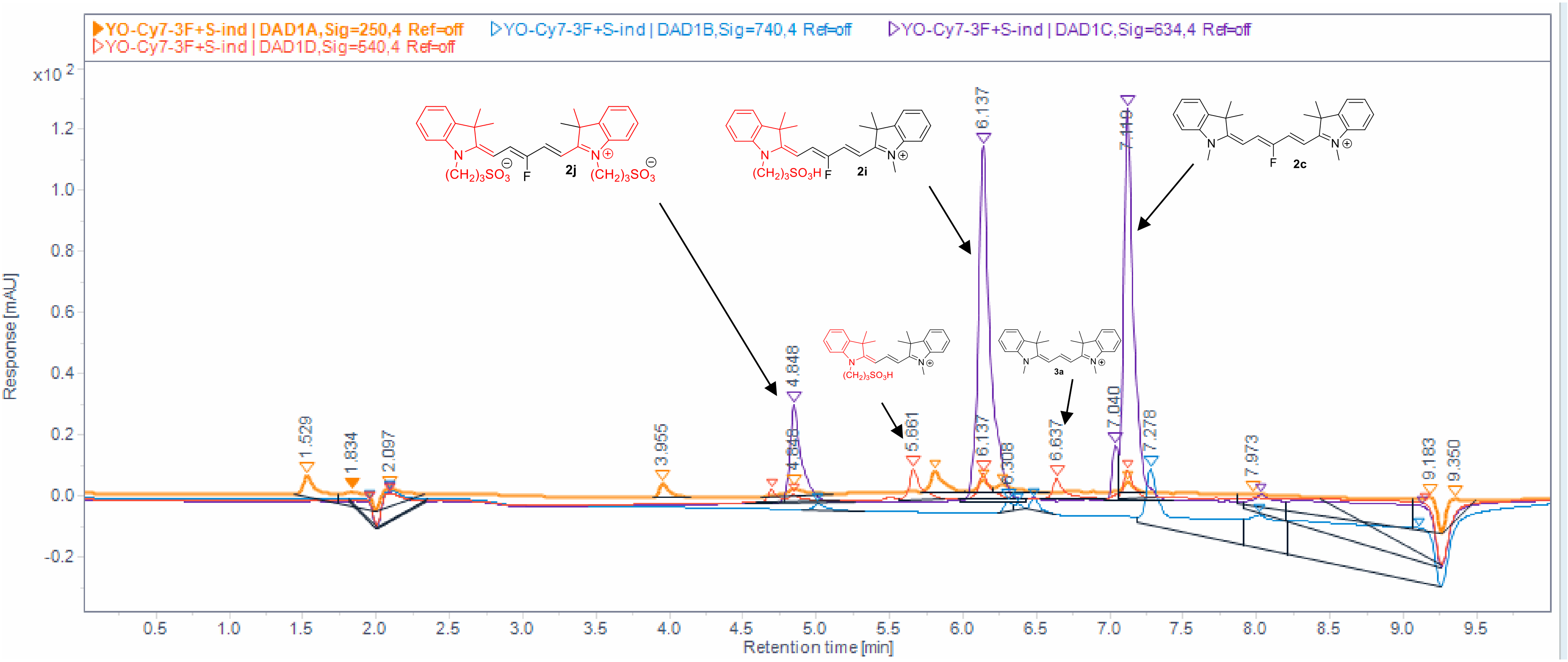

Figure S24. HPLC of the crude reaction mixture of the chain-shortening reaction of **1c** with indolinium salt **5** (1 eq) and DIPA (2.5 eq) at 50 °C in acetonitrile after 21 h.

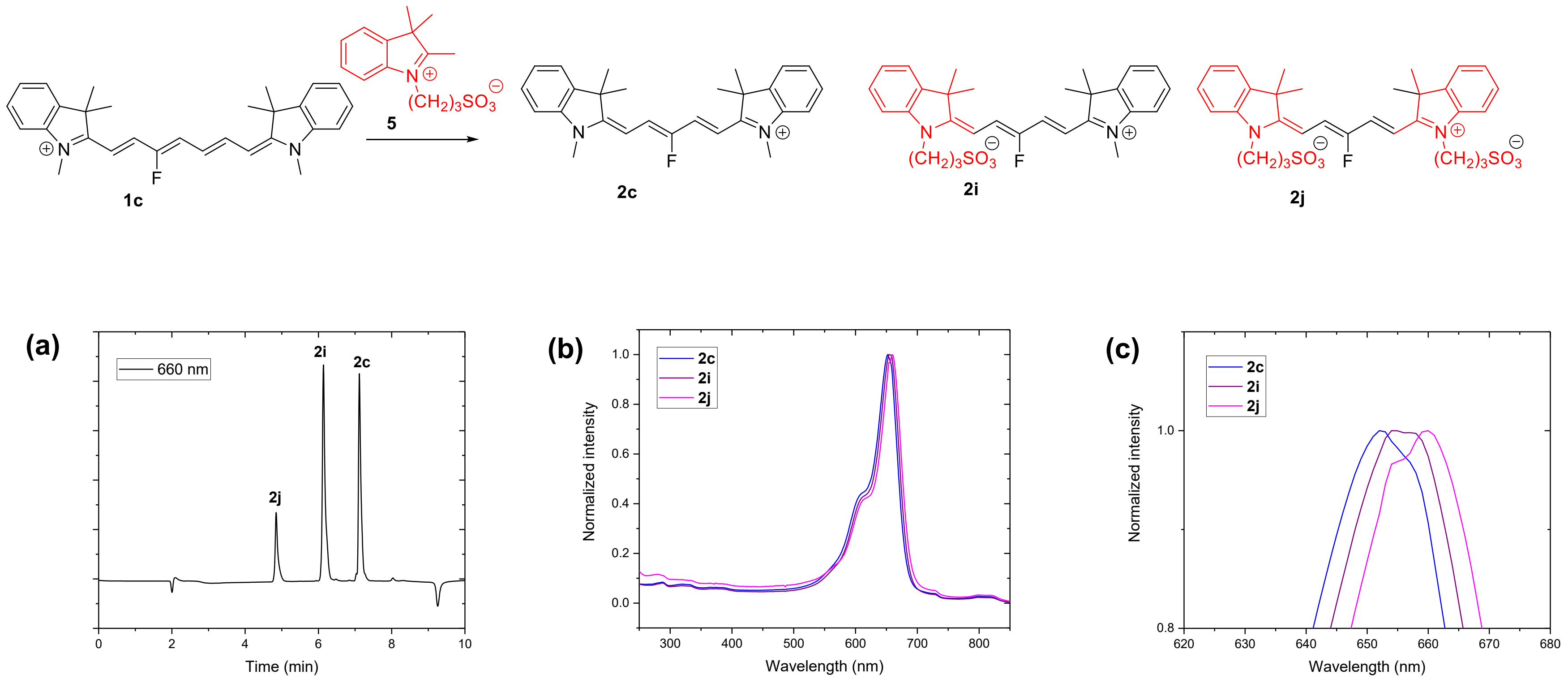

Figure S25. a) HPLC chromatogram of the crude reaction mixture of **1c** with indolinium salt **5** (1 eq), DIPA (2.5 eq) at 50 °C in acetonitrile after 21 h; b) and c) UV-Vis spectra of the truncated products.

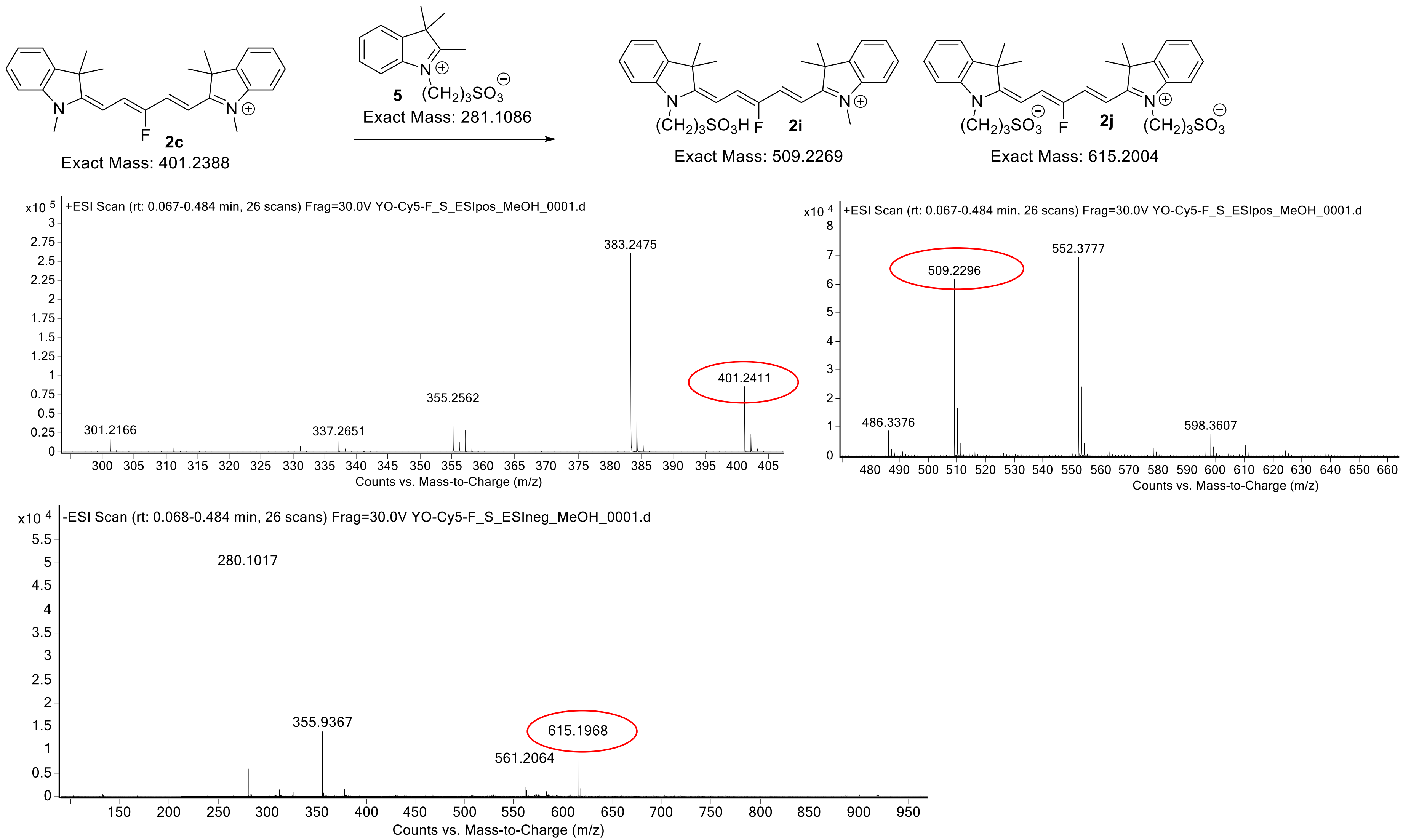

Figure S26. HRMS of the crude reaction mixture of the reaction of **2c** with indolinium salt **5** (1 eq) and DIPA (2.5 eq) at 50 °C in acetonitrile after 21 h.

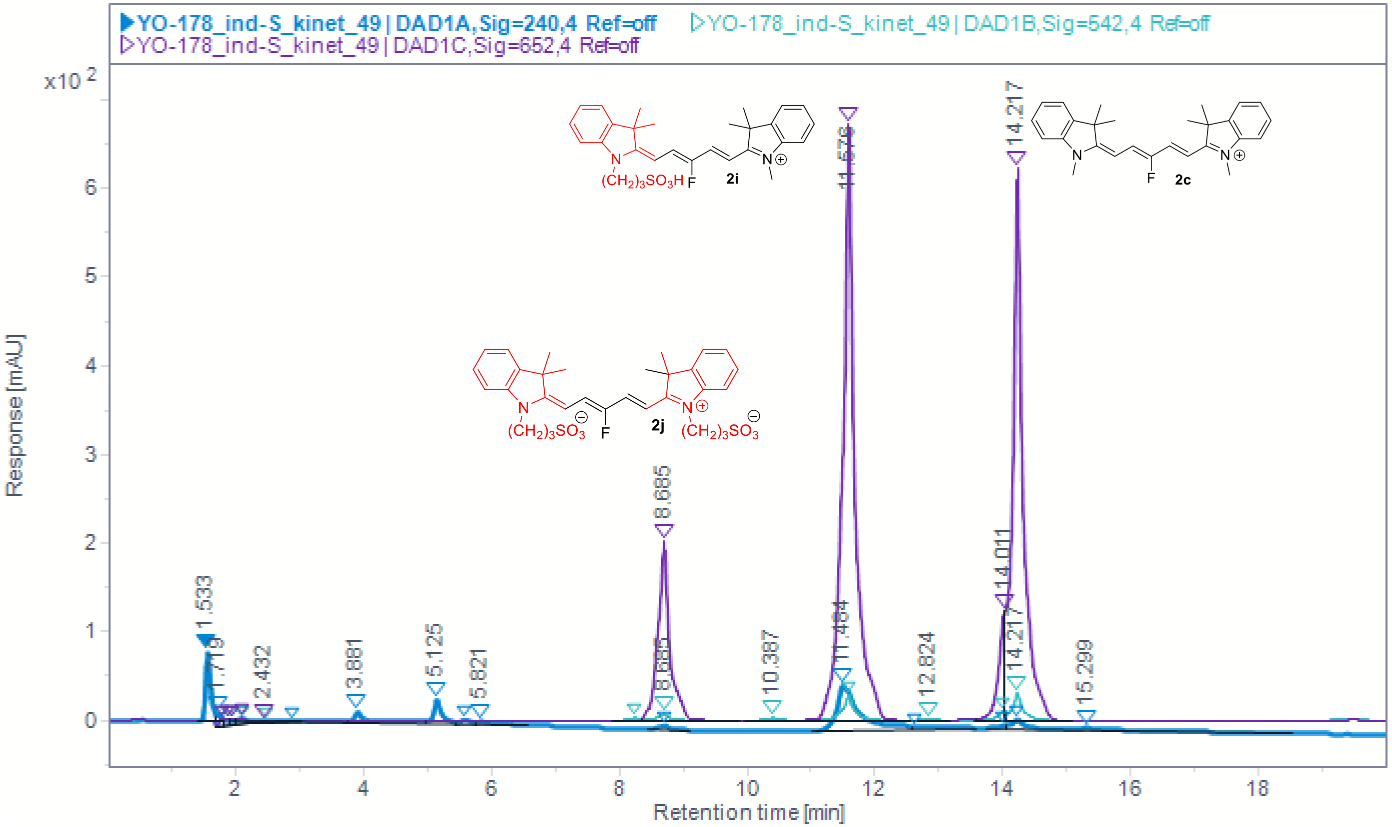

Figure S27. HPLC of the crude reaction mixture of the reaction of **2c** with indolinium salt **5** (1 eq) and DIPA (2.5 eq) at 50 °C in acetonitrile after 21 h.

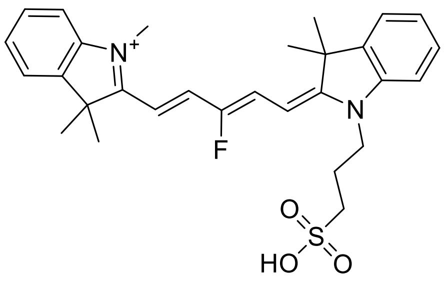

2i

Chemical Formula: $C_{29}H_{34}FN_2O_3S^+$

Exact Mass: 509.2269

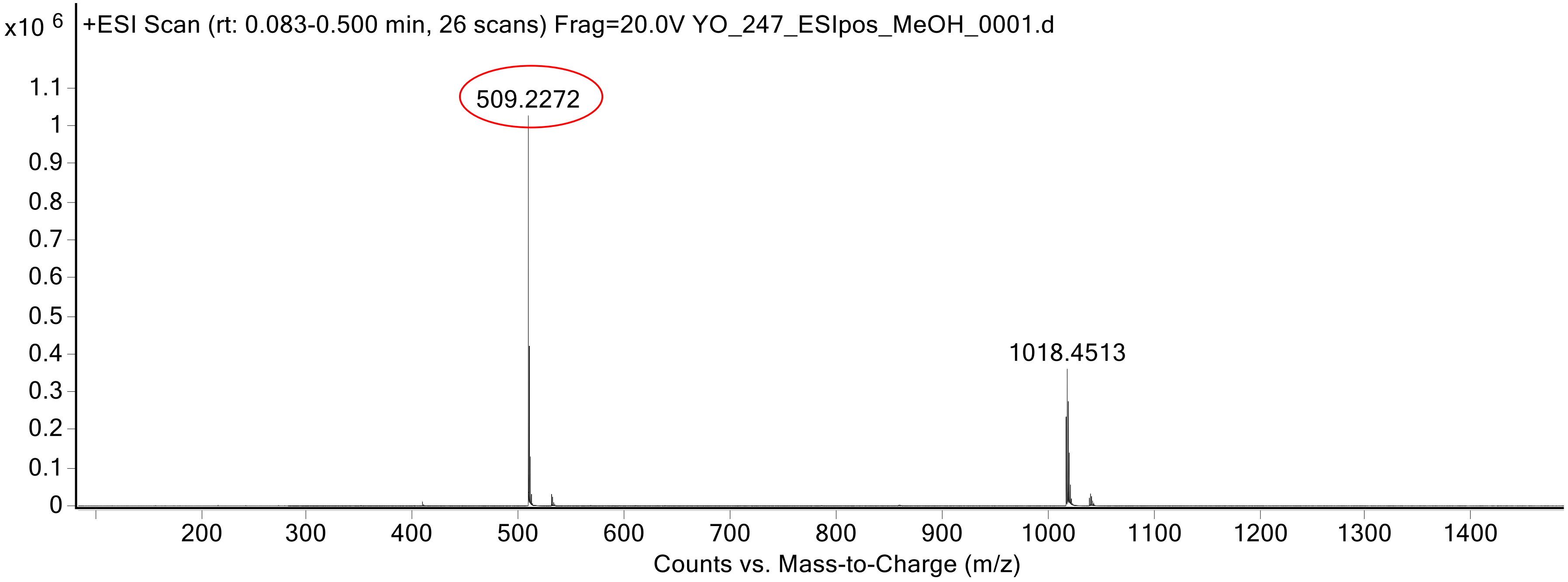

Figure S28. HRMS of an isolated **2i.**

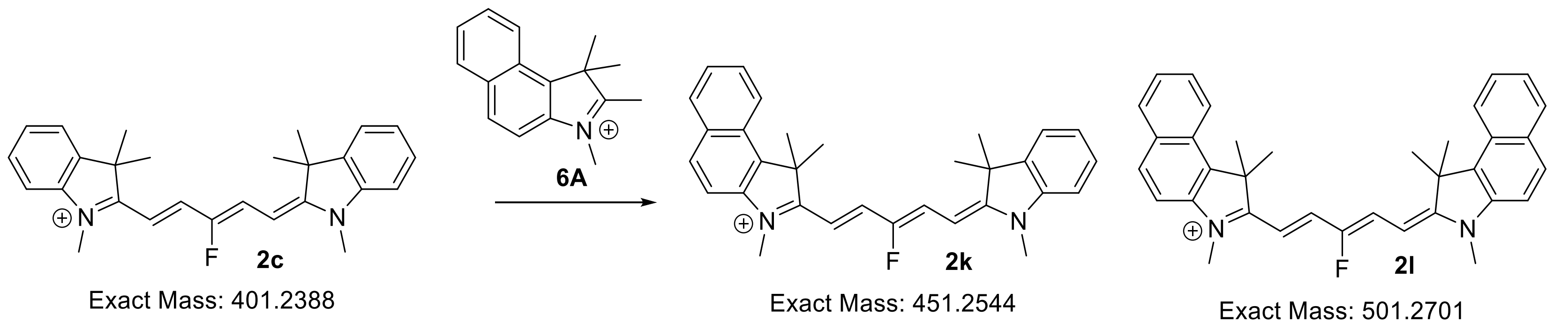

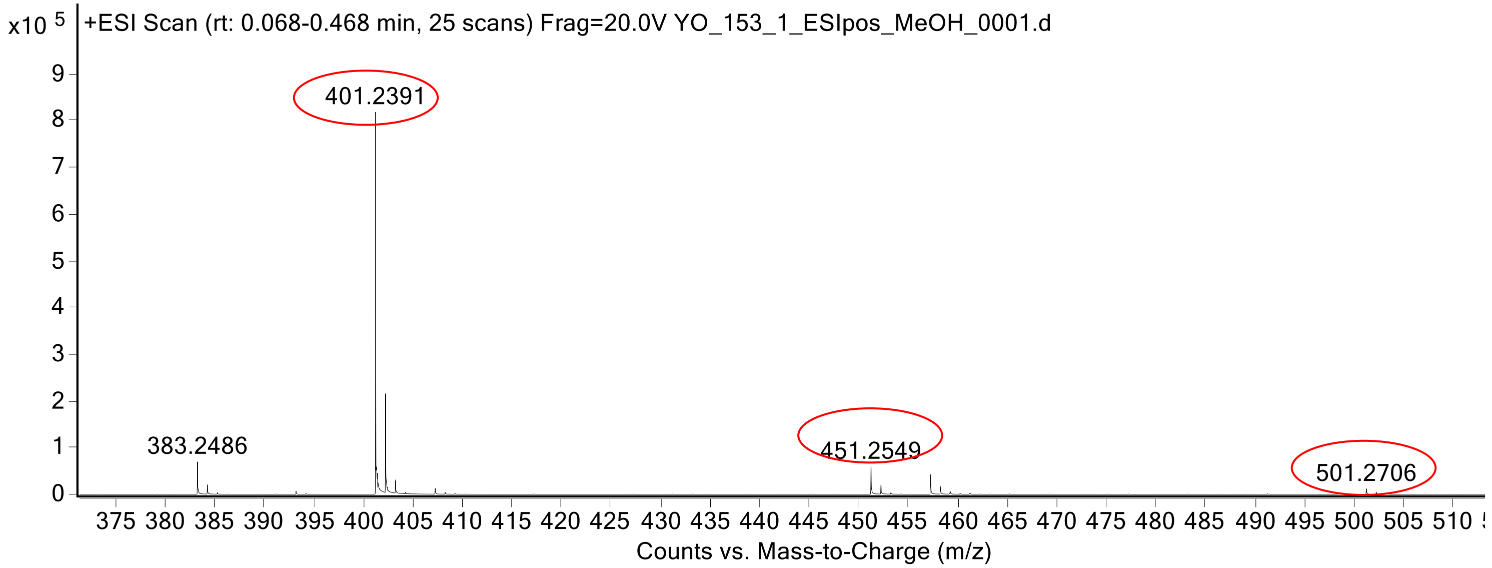

Figure S29. HRMS of the crude reaction mixture of the reaction of **2c** with indolinium salt **6A** (1 eq) and DIPA (2.5 eq) at 50 °C in acetonitrile after 21 h.

(a)

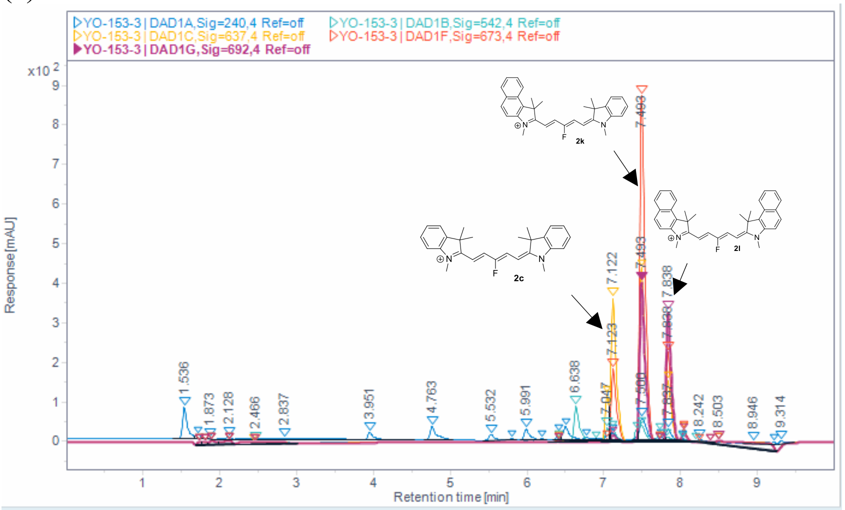

(c)

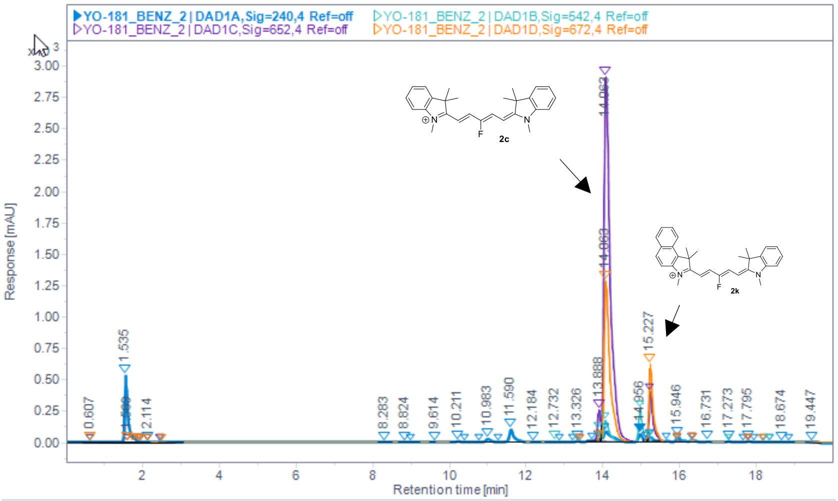

(b)

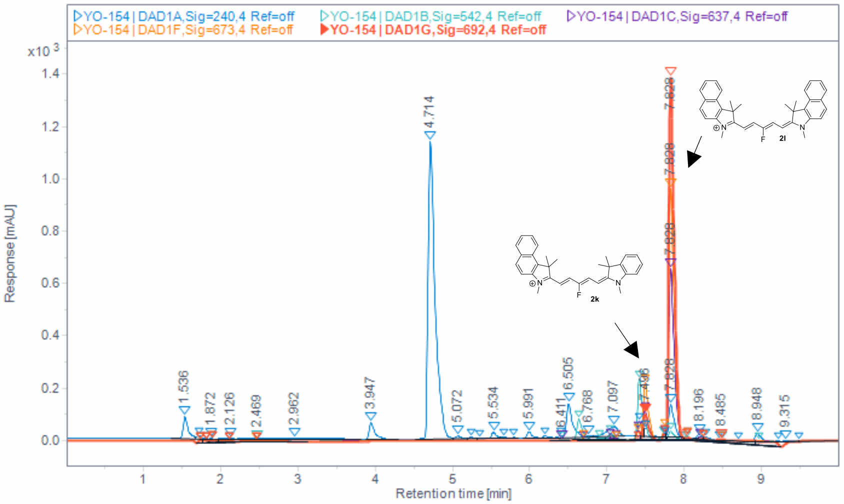

Figure S30. HPLC of the crude reaction mixture of the reaction of (a) **2c** with Fischer's base **6B** (1 eq) at 50 °C in acetonitrile after 21 h; (b) **2c** with Fischer's base **6B** (10) at 50 °C in acetonitrile after 21 h; (c) **2c** with indolinium salt **6A** (1 eq) and DIPA (2.5 eq) at 50 °C in acetonitrile after 21 h

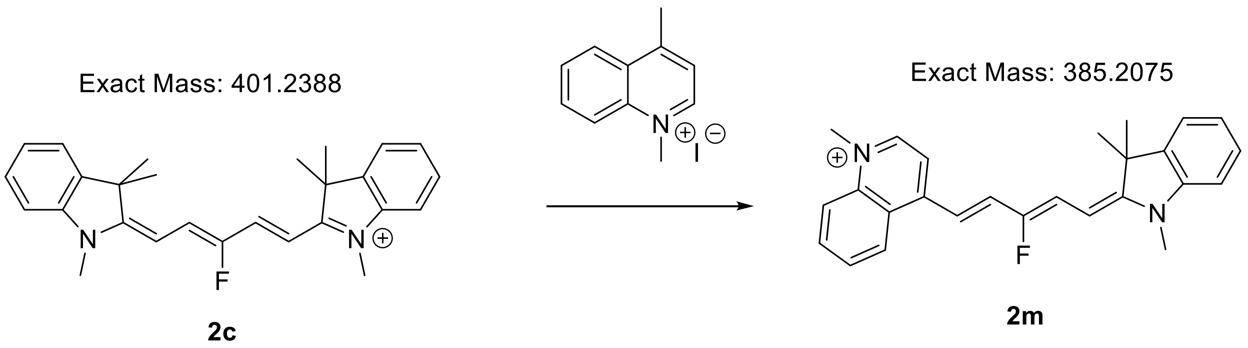

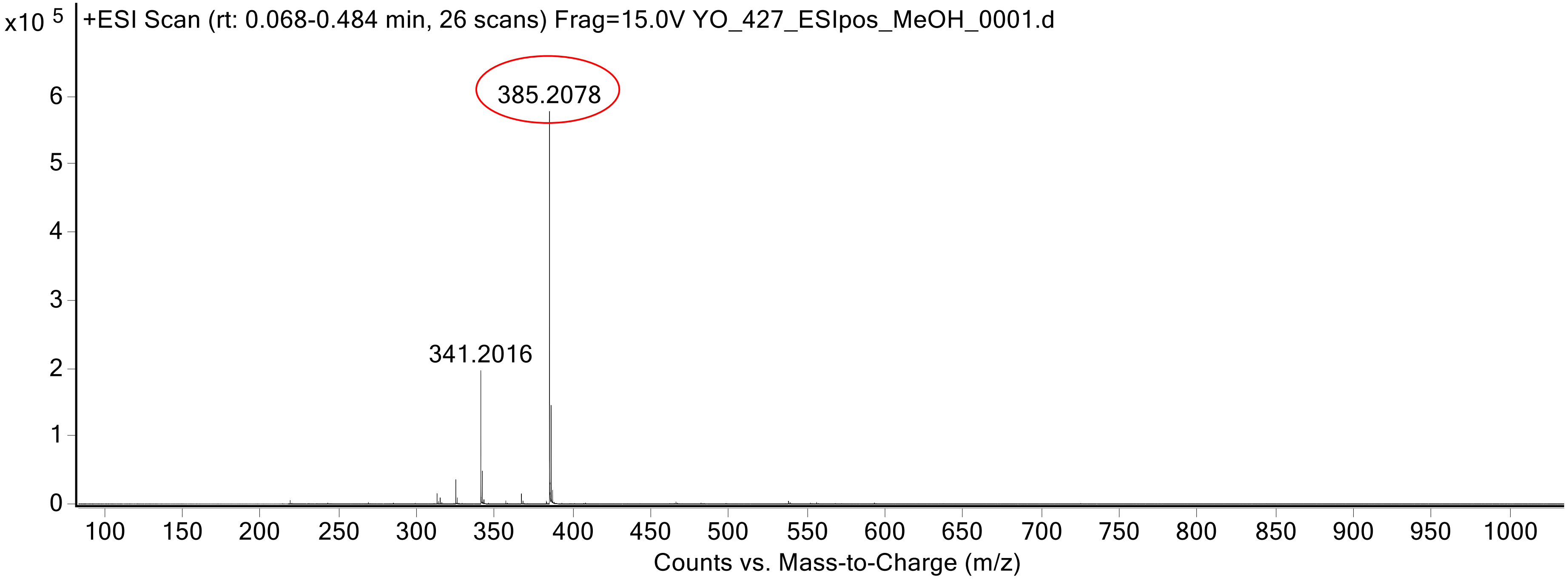

Figure S31. HRMS of the crude reaction mixture of the reaction of **2c** with 1,4-dimethylquinolinium iodide (5 eq), DIPA (10 eq) at 80 °C in acetonitrile after 17h.

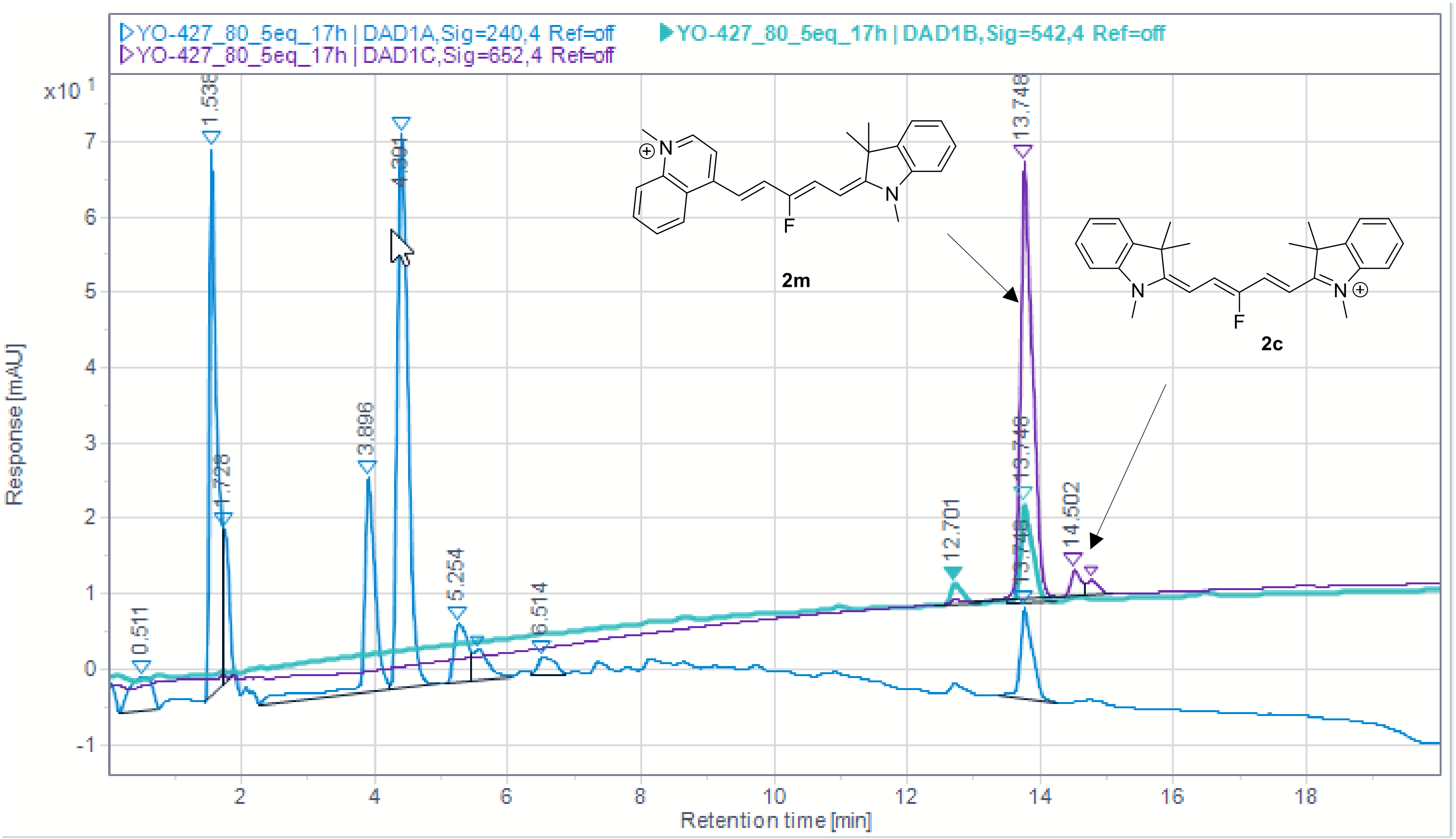

Figure S32. HRMS of the crude reaction mixture of the reaction of **2c** with 1,4-dimethylquinolinium iodide (5 eq), DIPA (10 eq) at 80 °C in acetonitrile after 17 h.

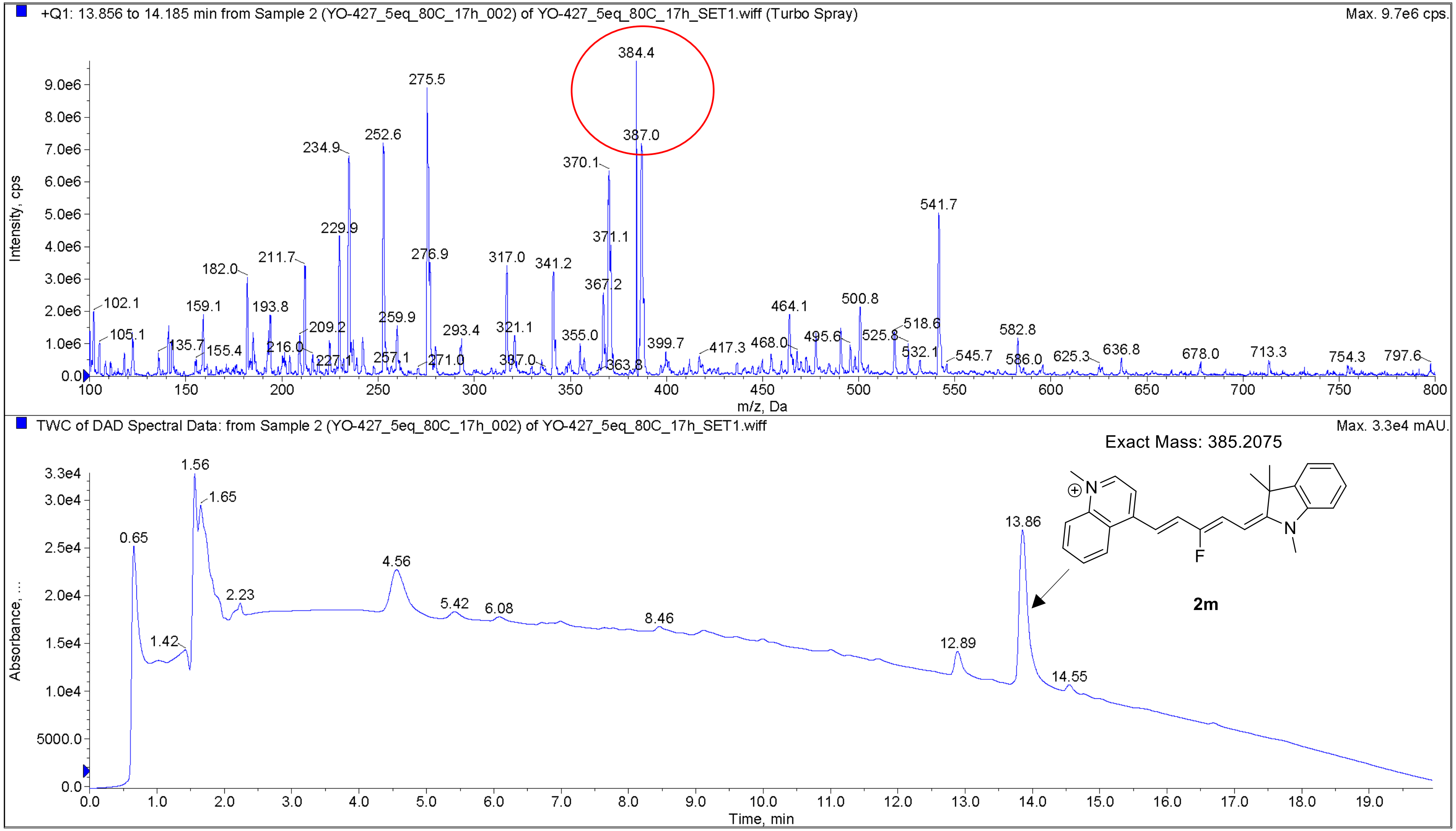

Figure S33. HPLC-MS of the crude reaction mixture of the reaction of **2c** with 1,4-dimethylquinolinium iodide (5 eq), DIPA (10 eq) at 80 °C in acetonitrile after 17 h.

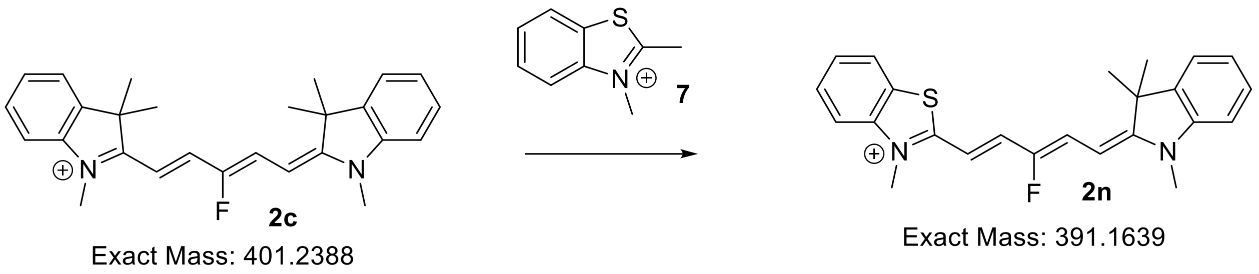

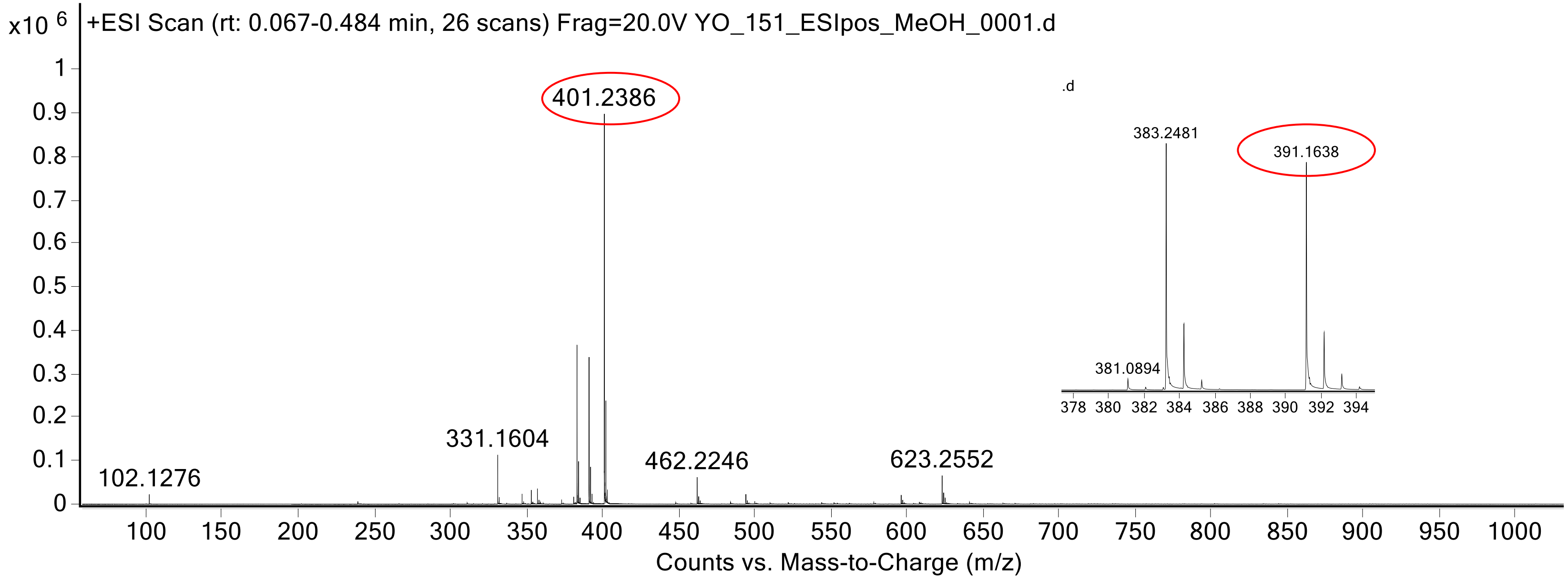

Figure S34. HRMS of the crude reaction mixture of the reaction of **2c** with indolinium salt **7** (1 eq) and DIPA (2.5 eq) at 50 °C in acetonitrile after 21 h.

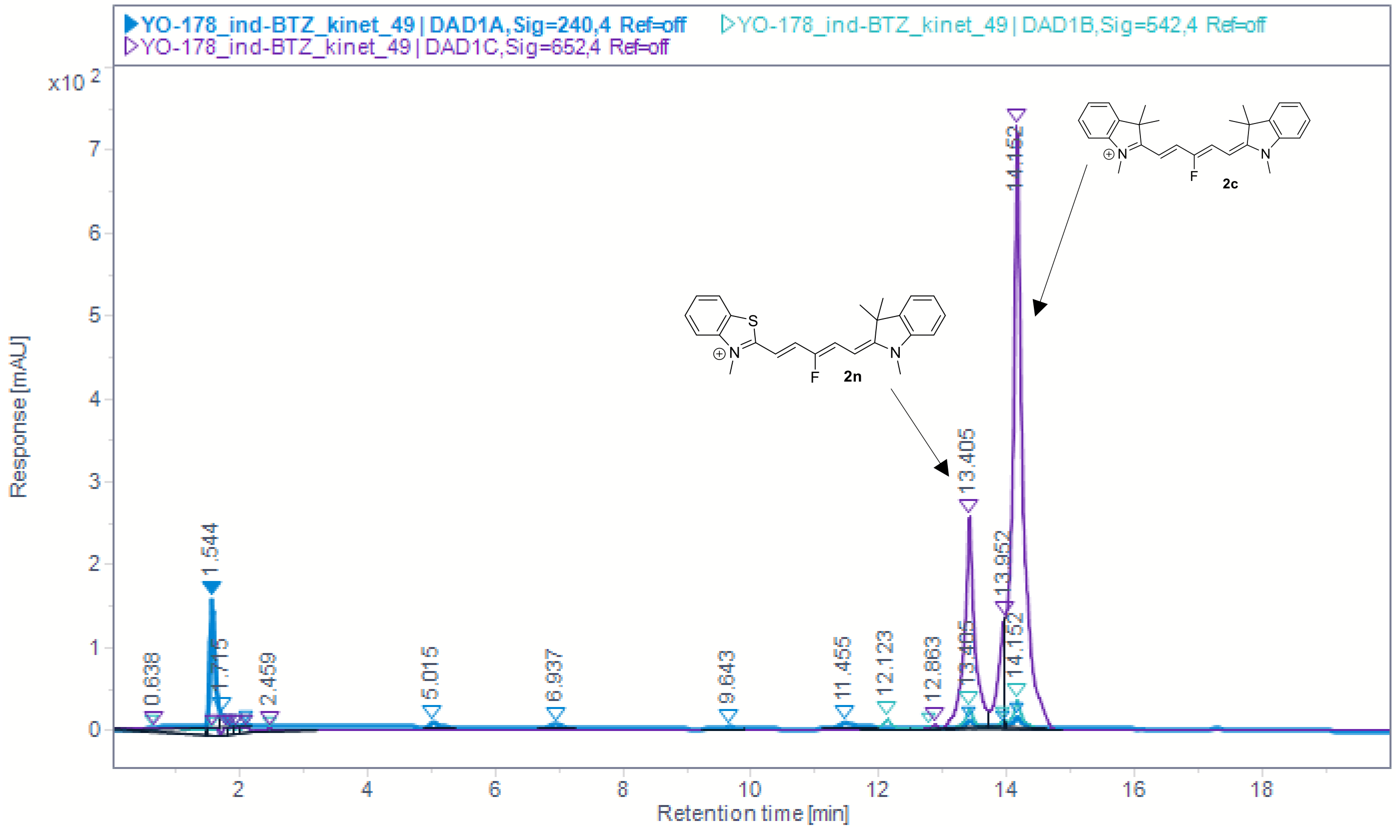

Figure S35. HPLC of the crude reaction mixture of the reaction of **2c** with indolinium salt **7** (1 eq) and DIPA (2.5 eq) at 50 °C in acetonitrile after 21 h.

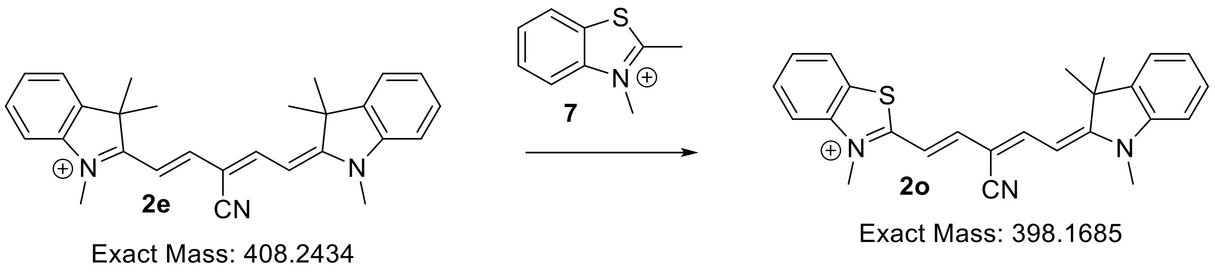

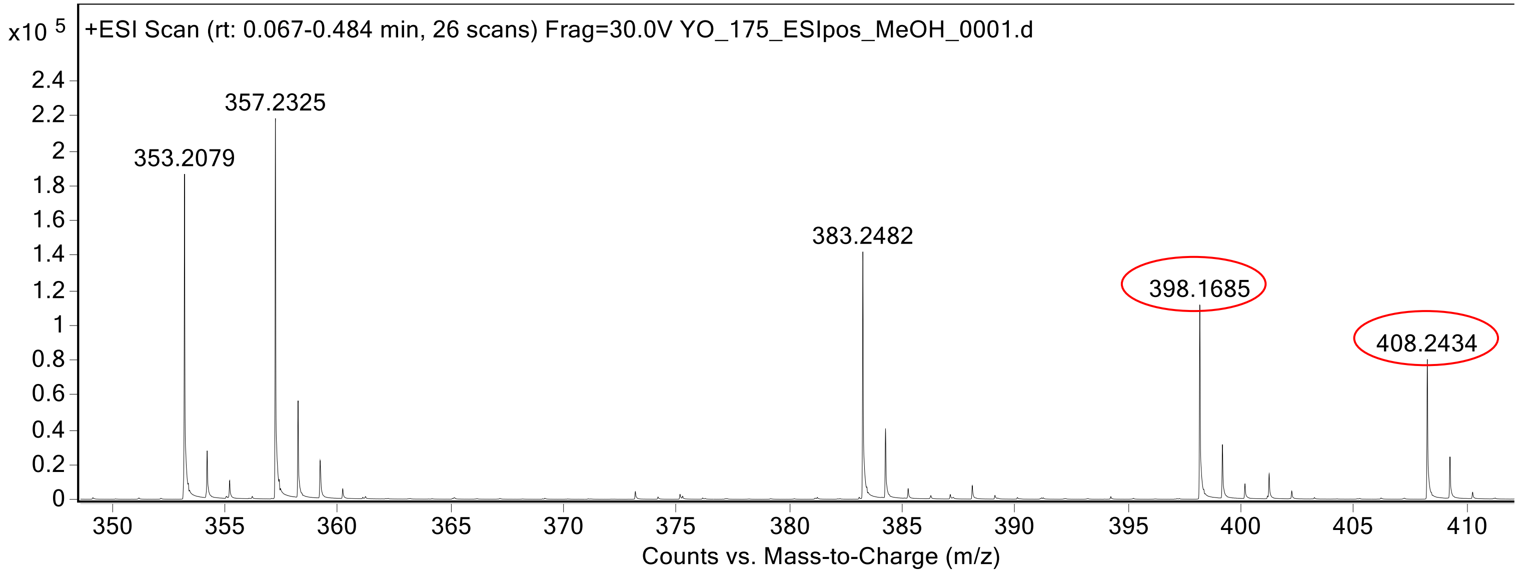

Figure S36. HRMS of the crude reaction mixture of the reaction of **2e** with indolinium salt **7** (1 eq), DIPA (2.5 eq) at 50 °C in acetonitrile after 21h.

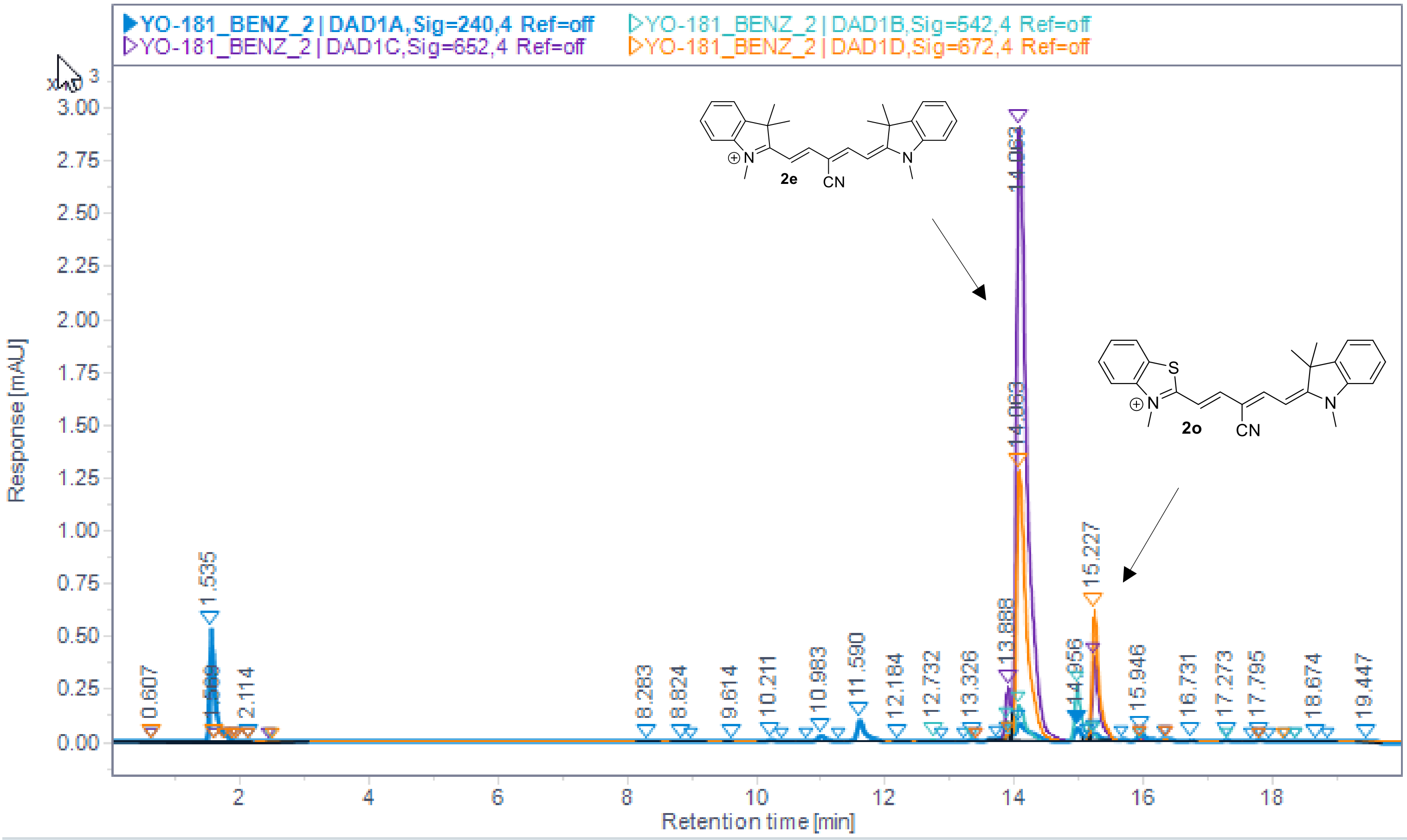

Figure S37. HRMS of the crude reaction mixture of the reaction of **2e** with indolinium salt **7** (1 eq), DIPA (2.5 eq) at 50 °C in acetonitrile after 21 h.

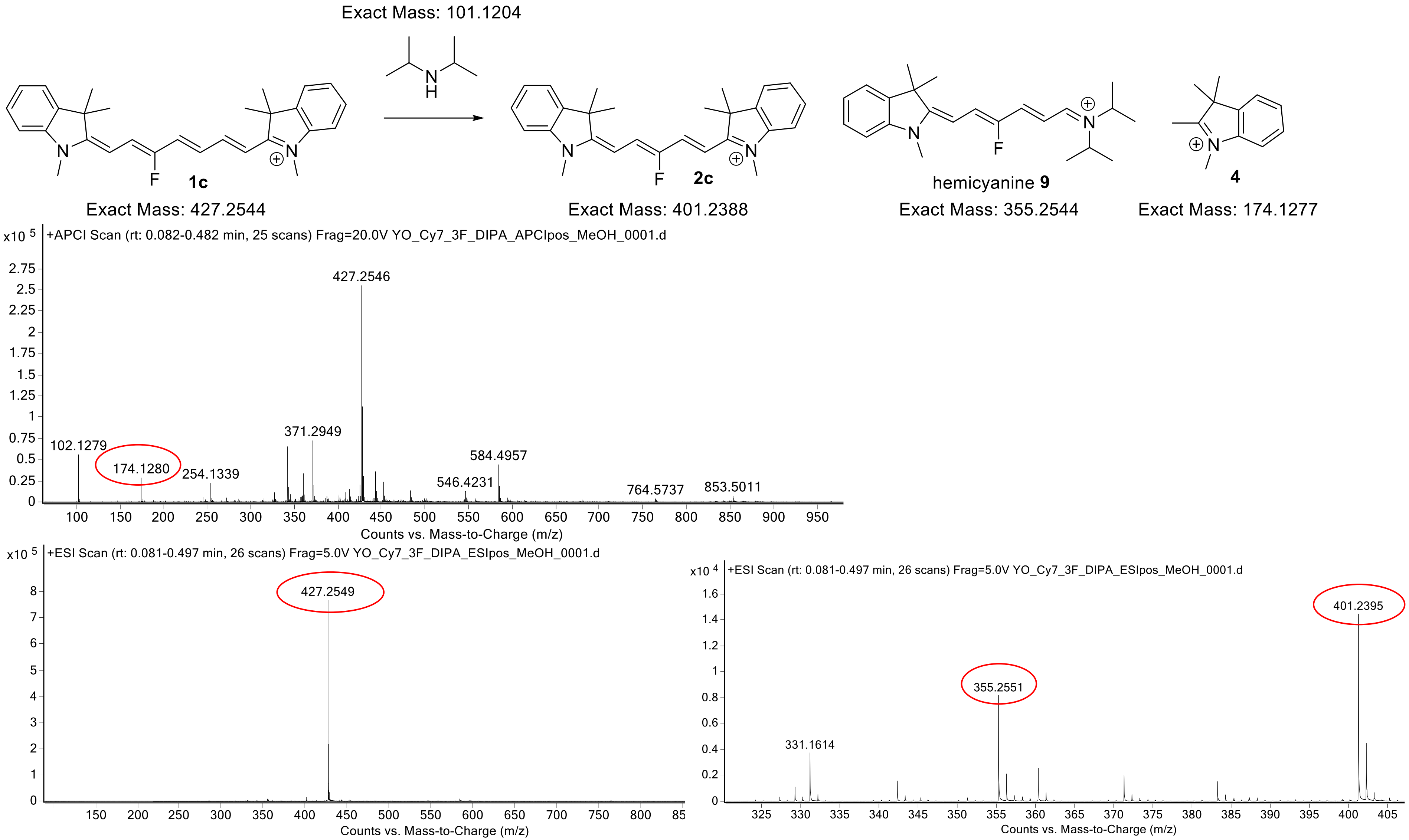

Figure S38. ESI$^+$ and APCI$^+$ HRMS of the crude reaction mixture of the reaction of **1c** with DIPA (2.5 eq) in acetonitrile at 50 °C after 21 h.

Table S2. APCI$^+$ and ESI$^+$ HRMS data of the crude reaction mixture of chain-shortening reactions of **1** with **4** (1 eq) and DIPA (2.5 eq) at 50 °C in acetonitrile after 21 h, indicating trimer intermediates.

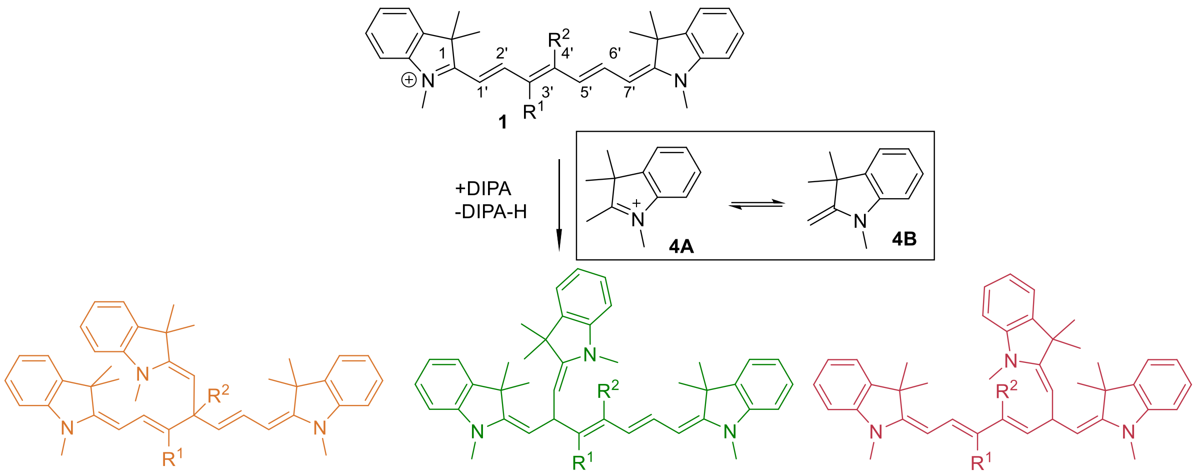

<table>
<tr><td></td><td>Signal ESI$^+$</td><td>Signal APCI$^+$</td></tr>
<tr><td>

1c

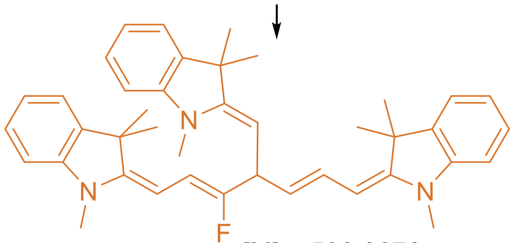

[M] = 599.3676

</td><td>

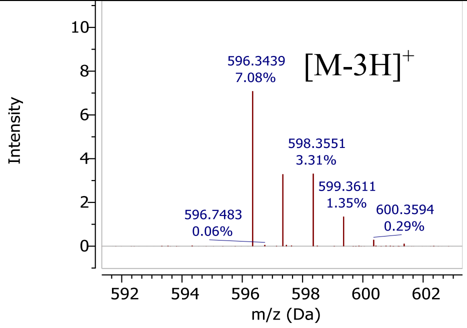

</td><td>

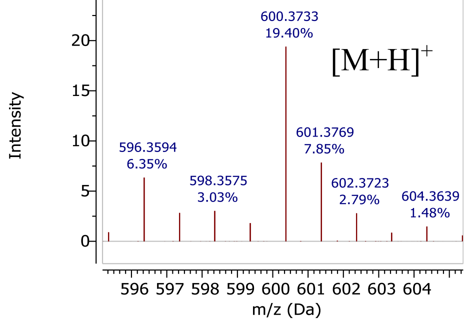

</td></tr>
</table>

1e

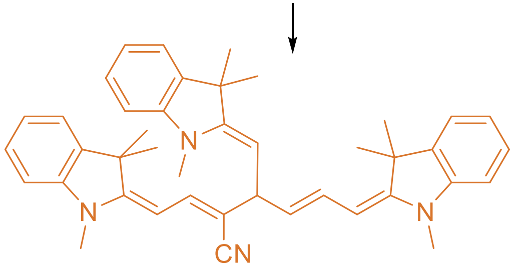

[M] = 606.3722

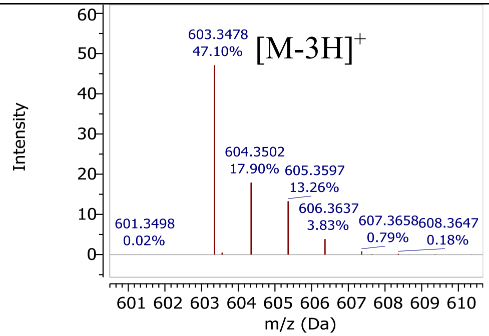

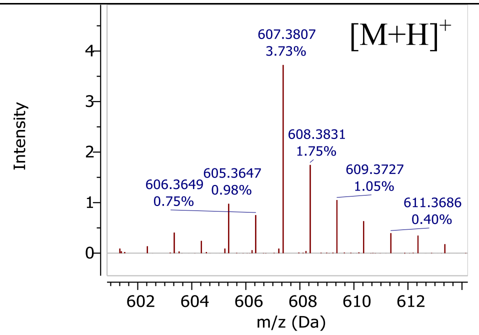

1b

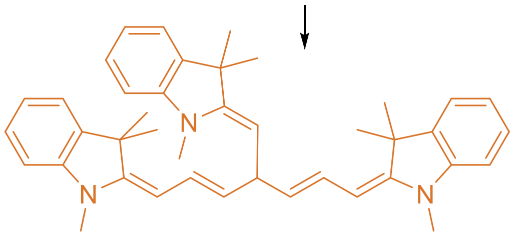

[M] = 581.3770

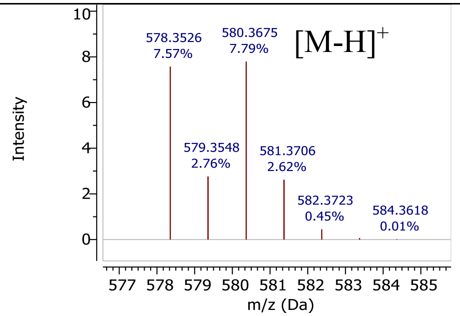

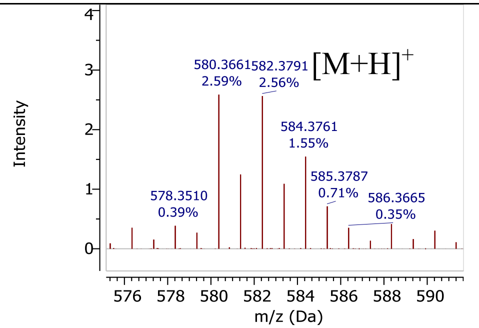

1d

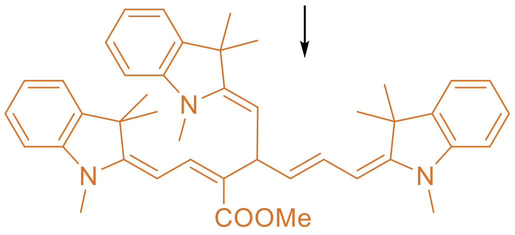

[M] = 639.3825

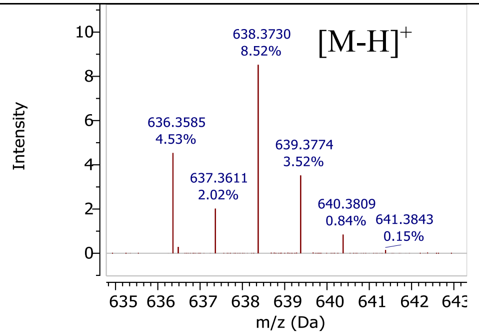

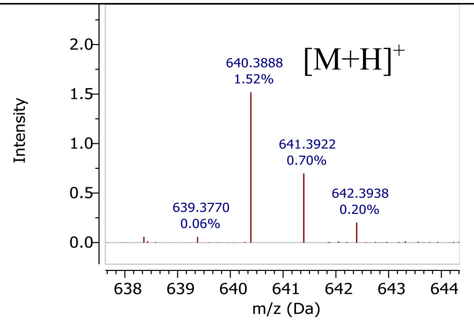

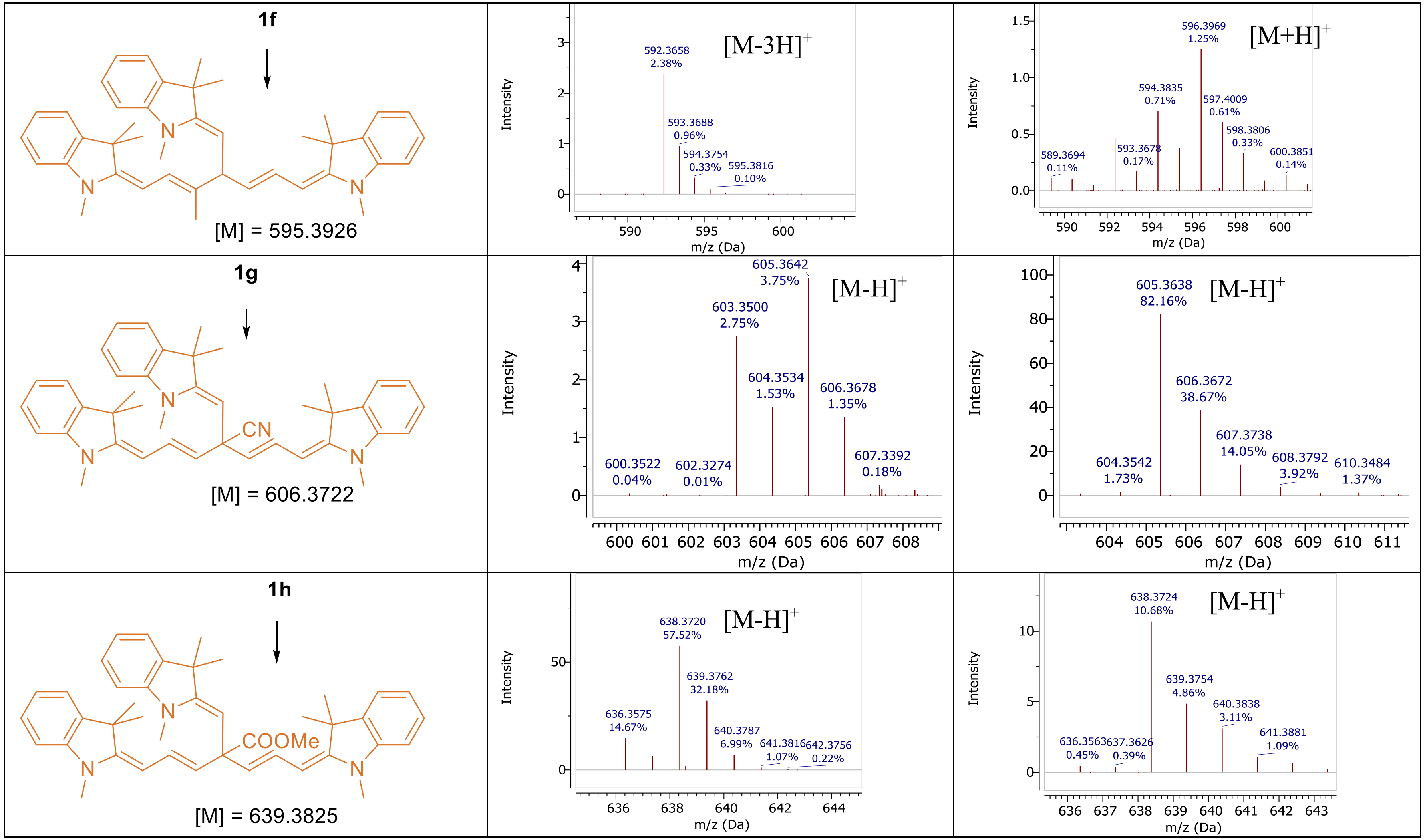

1f
[M] = 595.3926
[M-3H]+
592.3658 2.38%
593.3688 0.96%
594.3754 0.33%
595.3816 0.10%
Intensity
m/z (Da)
[M+H]+
596.3969 1.25%
594.3835 0.71%
597.4009 0.61%
598.3806 0.33%
589.3694 0.11%
593.3678 0.17%
600.3851 0.14%
1g
CN
[M] = 606.3722
[M-H]+
605.3642 3.75%
603.3500 2.75%
604.3534 1.53%
606.3678 1.35%
600.3522 0.04%
602.3274 0.01%
607.3392 0.18%
605.3638 82.16%
606.3672 38.67%
607.3738 14.05%
604.3542 1.73%
608.3792 3.92%
610.3484 1.37%
1h
COOMe
[M] = 639.3825
638.3720 57.52%
639.3762 32.18%
636.3575 14.67%
640.3787 6.99%
641.3816 1.07%
642.3756 0.22%
638.3724 10.68%
639.3754 4.86%
640.3838 3.11%
636.3563 0.45%
637.3626 0.39%
641.3881 1.09%

NMR Spectra

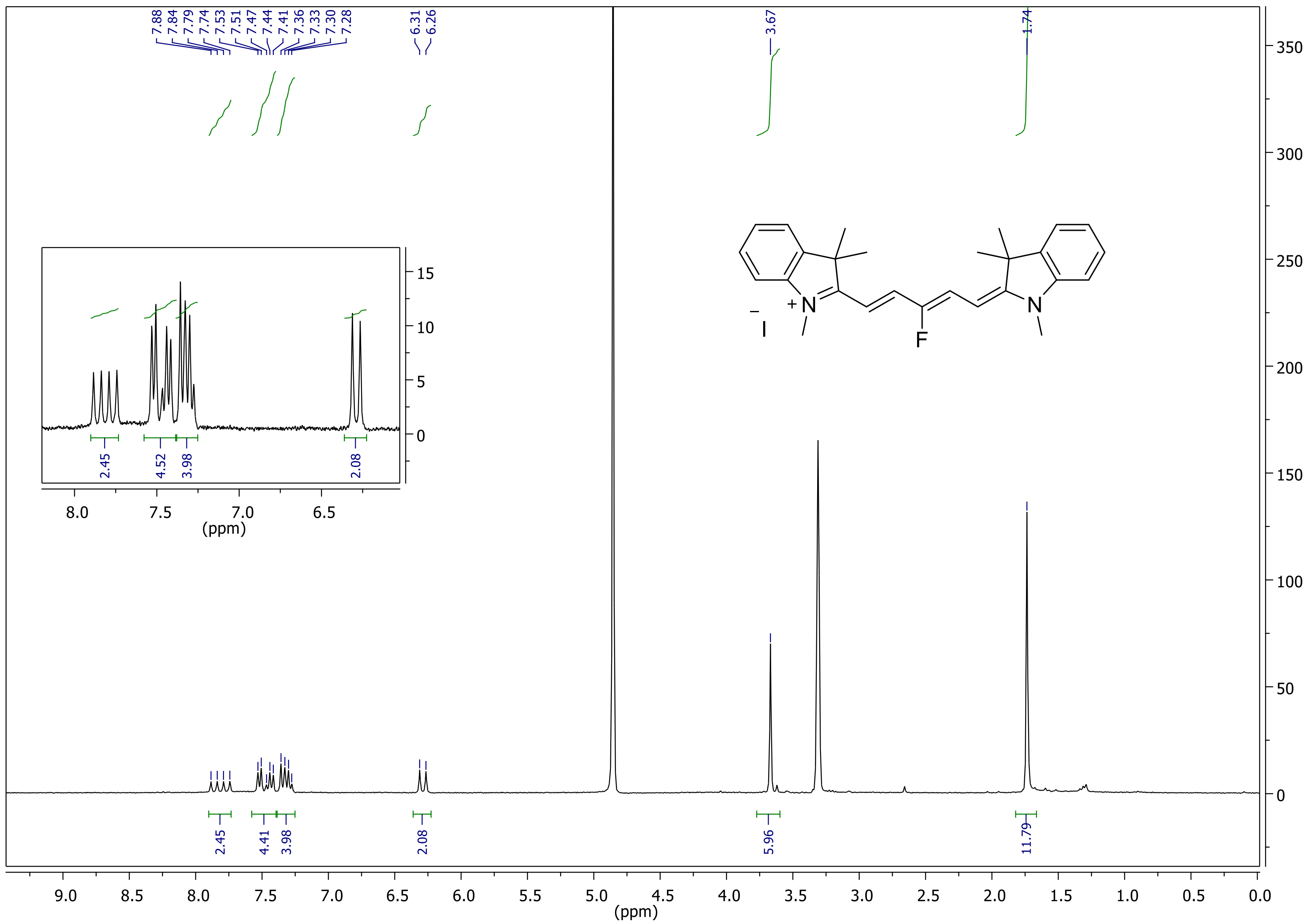

Figure S39. ^{1}H NMR (300 MHz, d_4–CD_3OD): **2c.**

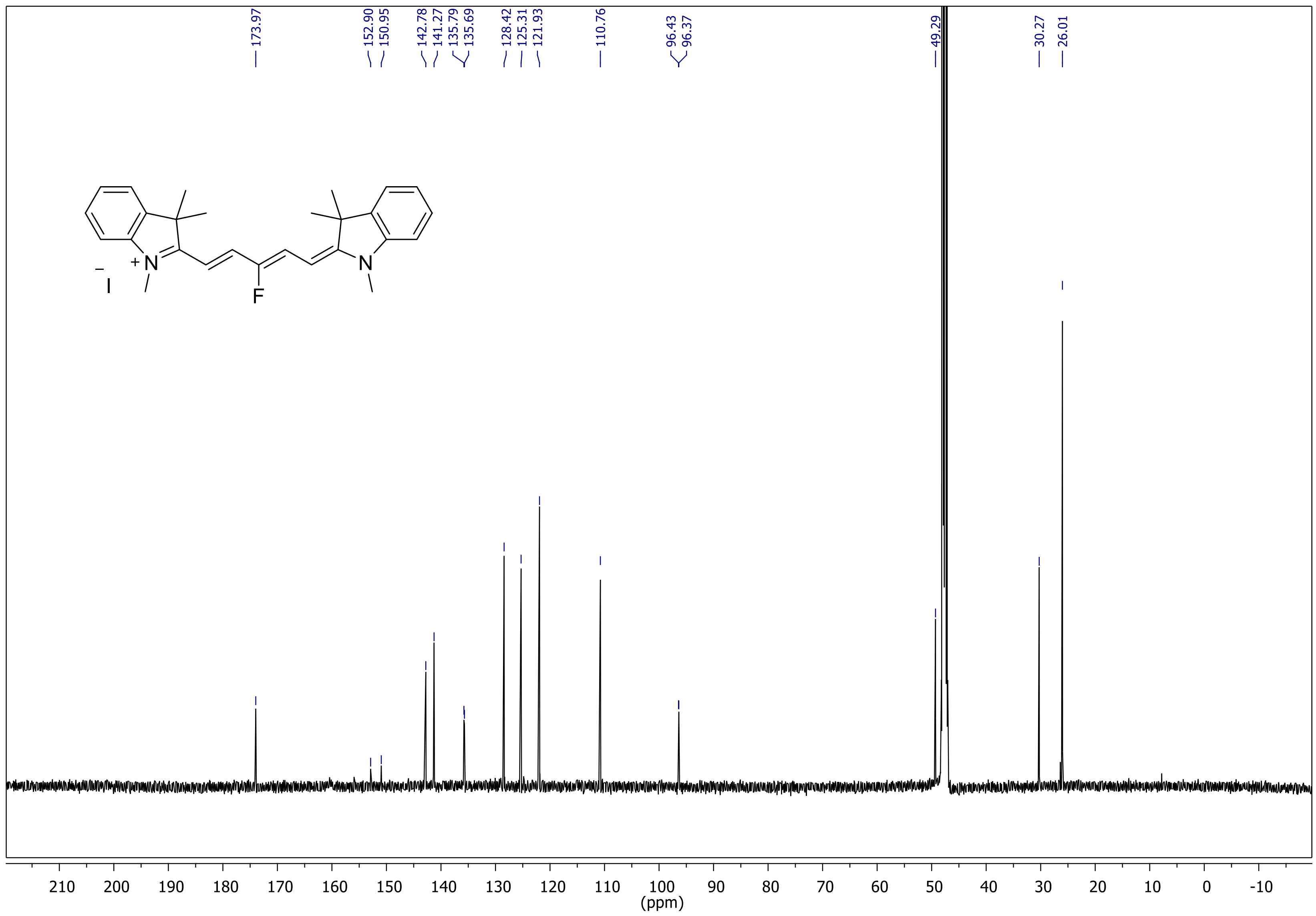

Figure S40. ^{13}C NMR (500 MHz, d_4–CD_3OD): **2c.**

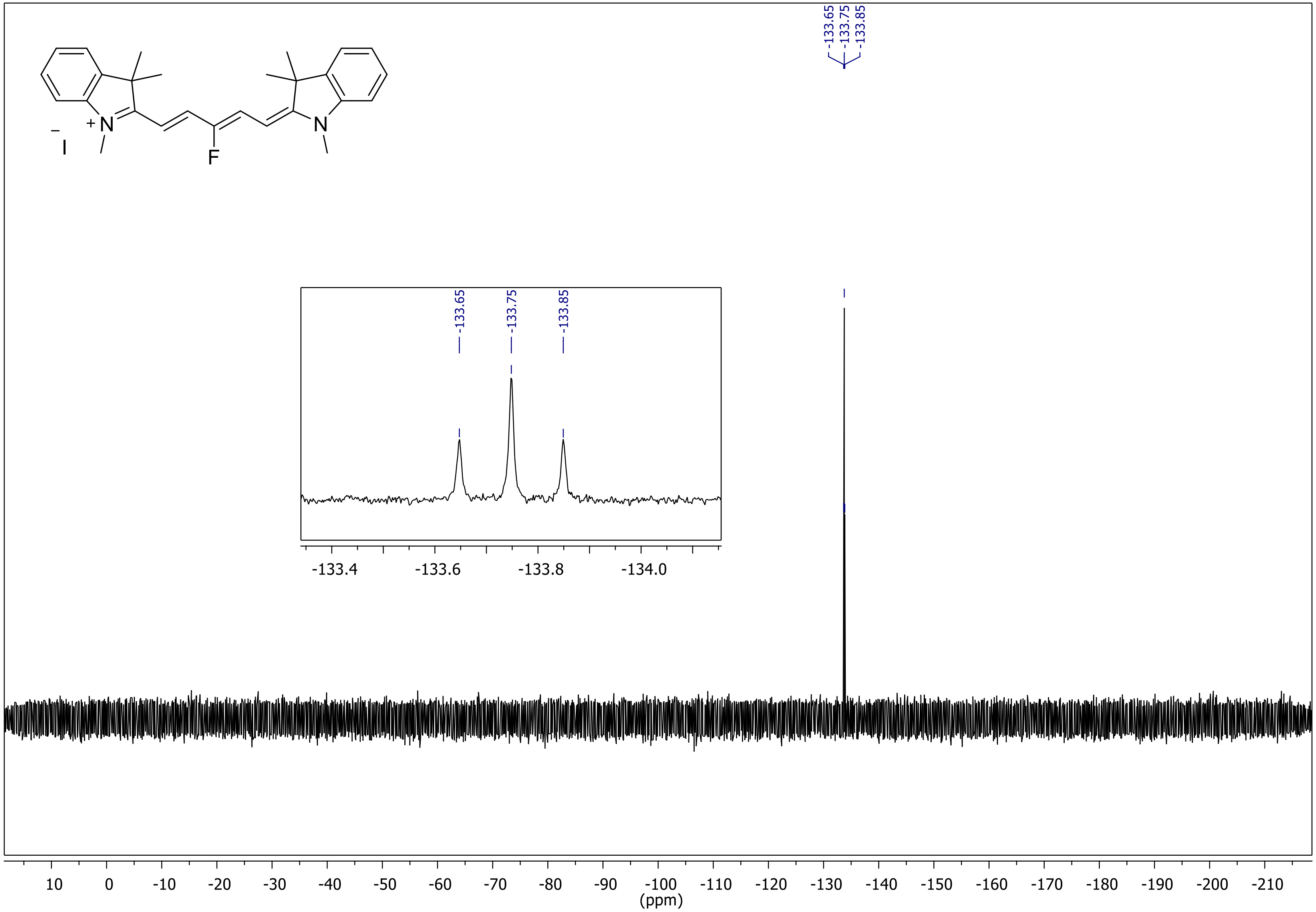

Figure S41. ^{19}F(H) NMR (300 MHz, d_4–CD_3OD): **2c.**

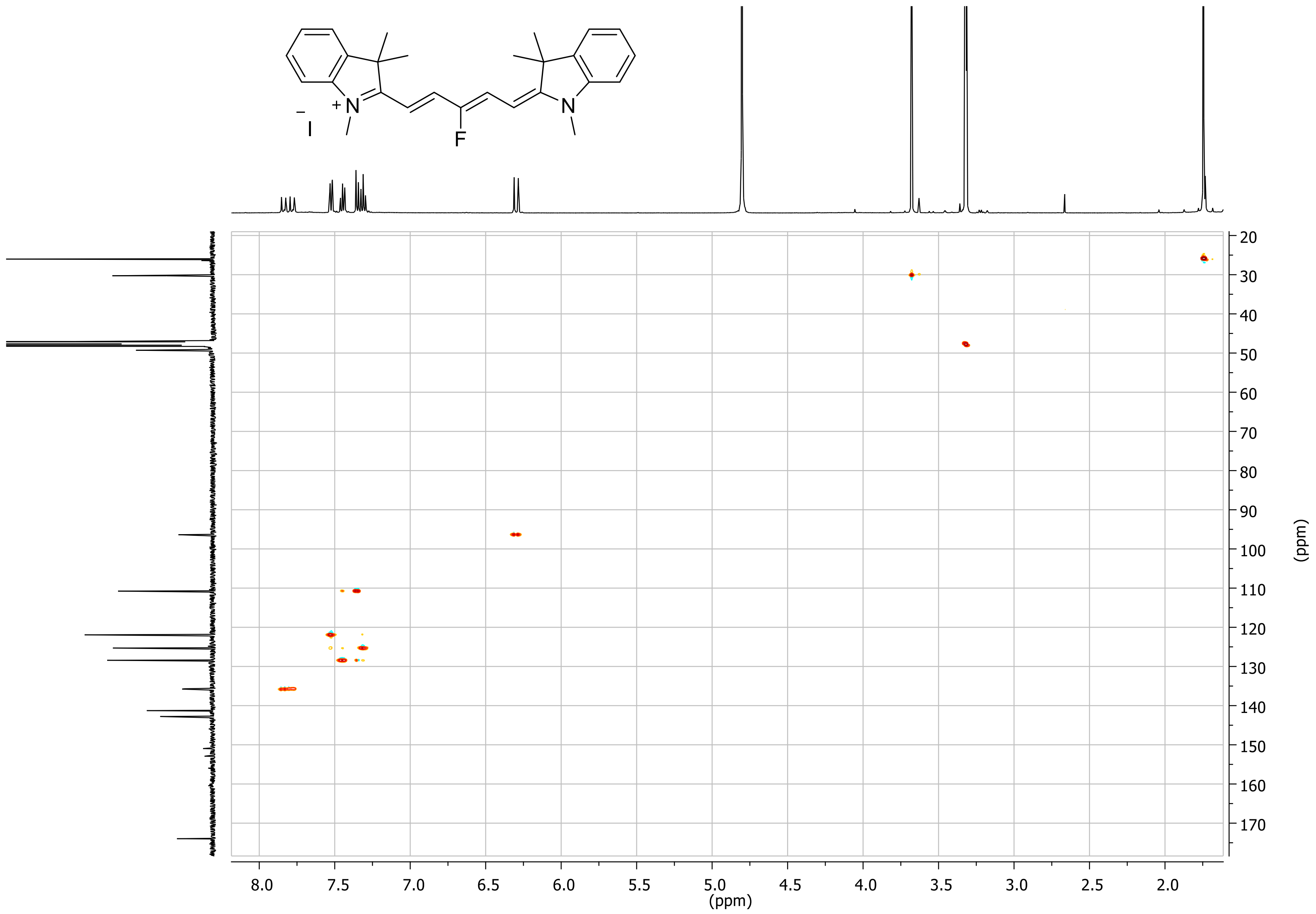

Figure S42. ^{1}H-^{13}C gHSQC (500 MHz, *d*$_4$–CD_3OD): **2c.**

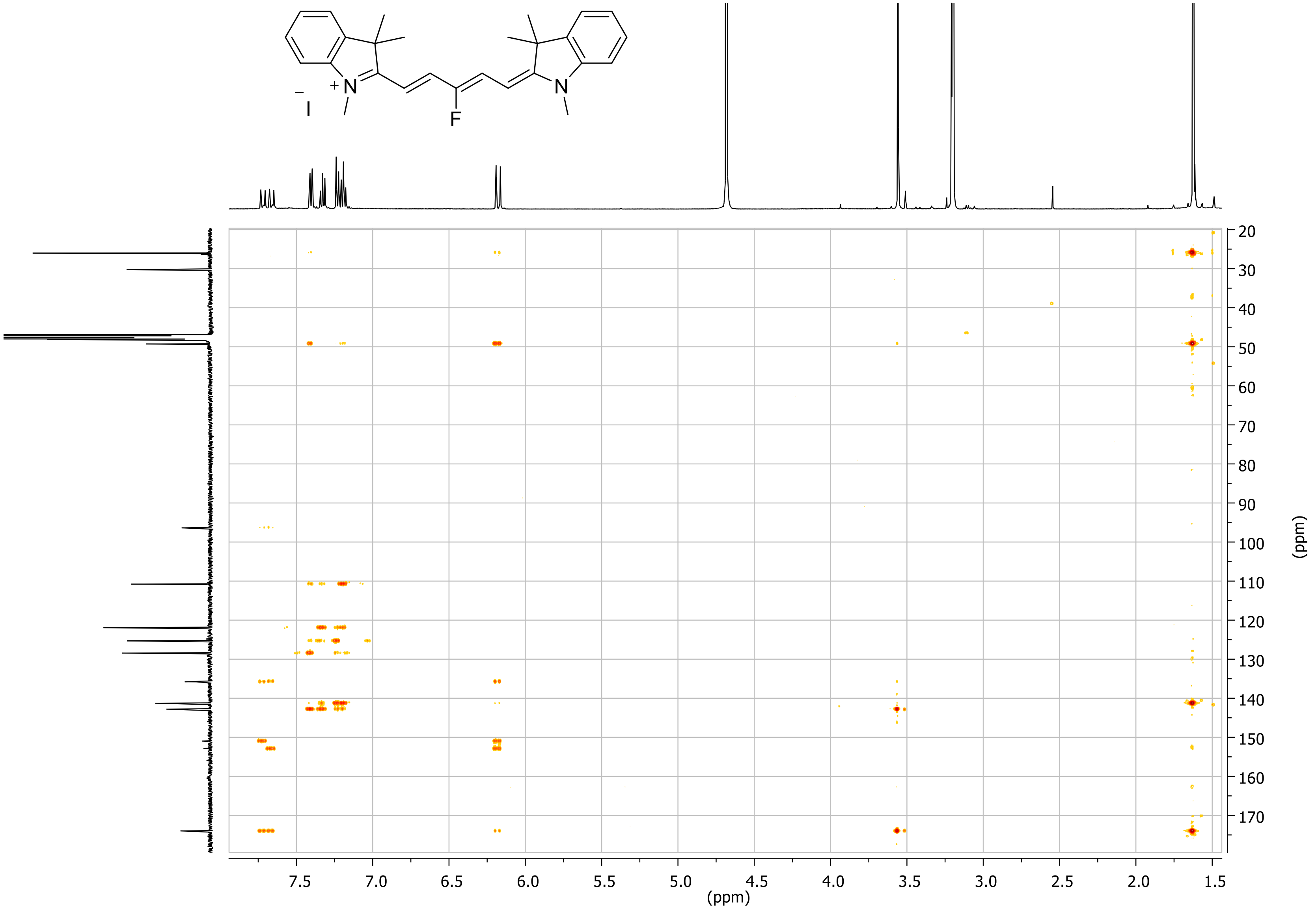

Figure S43. ^{1}H-^{13}C gHMBC (500 MHz, d_4–CD_3OD): **2c.**

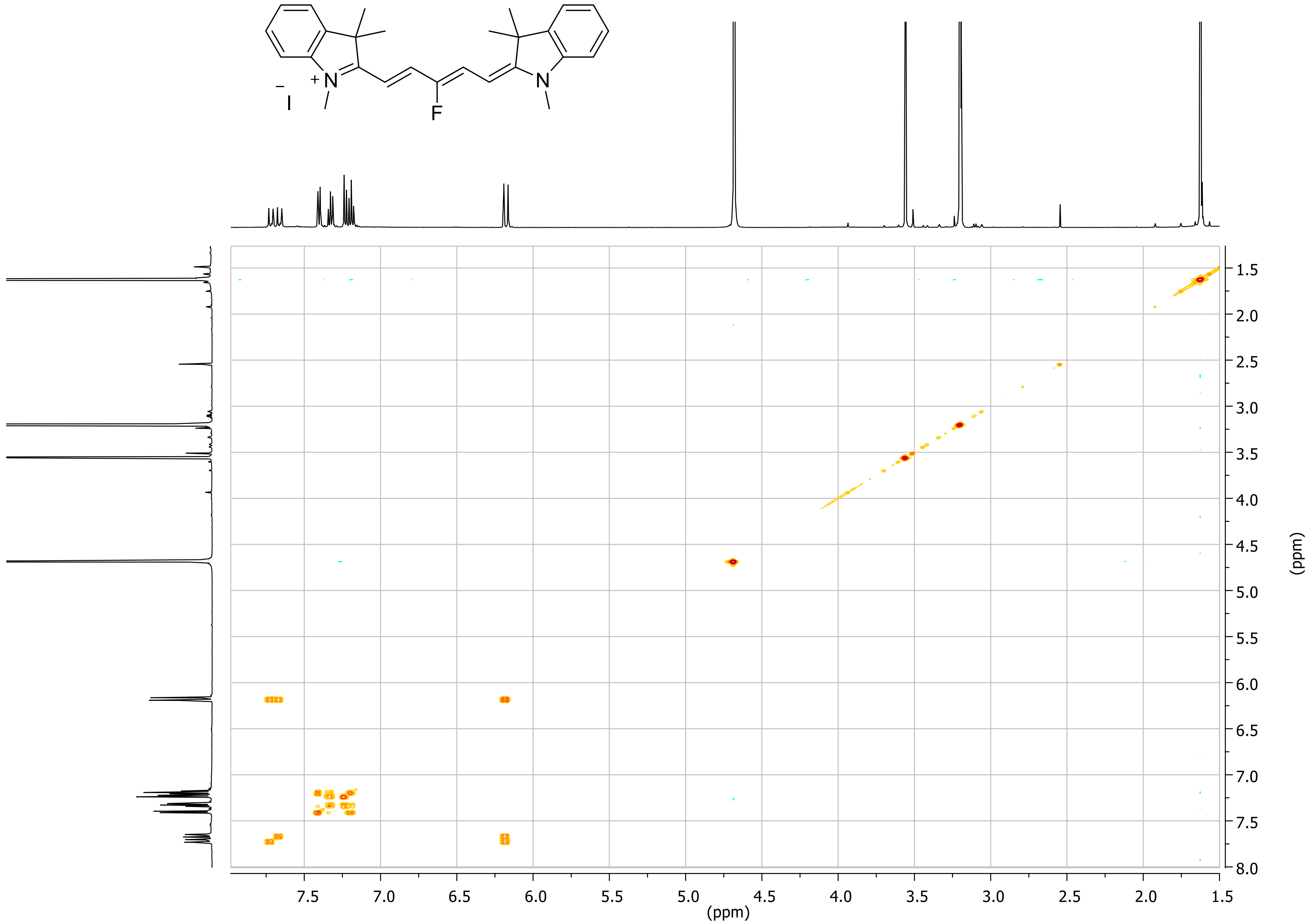

Figure S44. ^{1}H-^{1}H COSY (500 MHz, d_4–CD_3OD): **2c.**

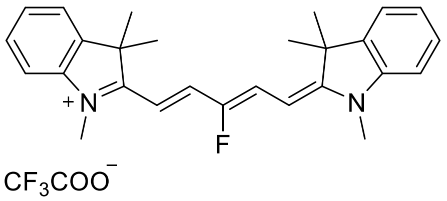

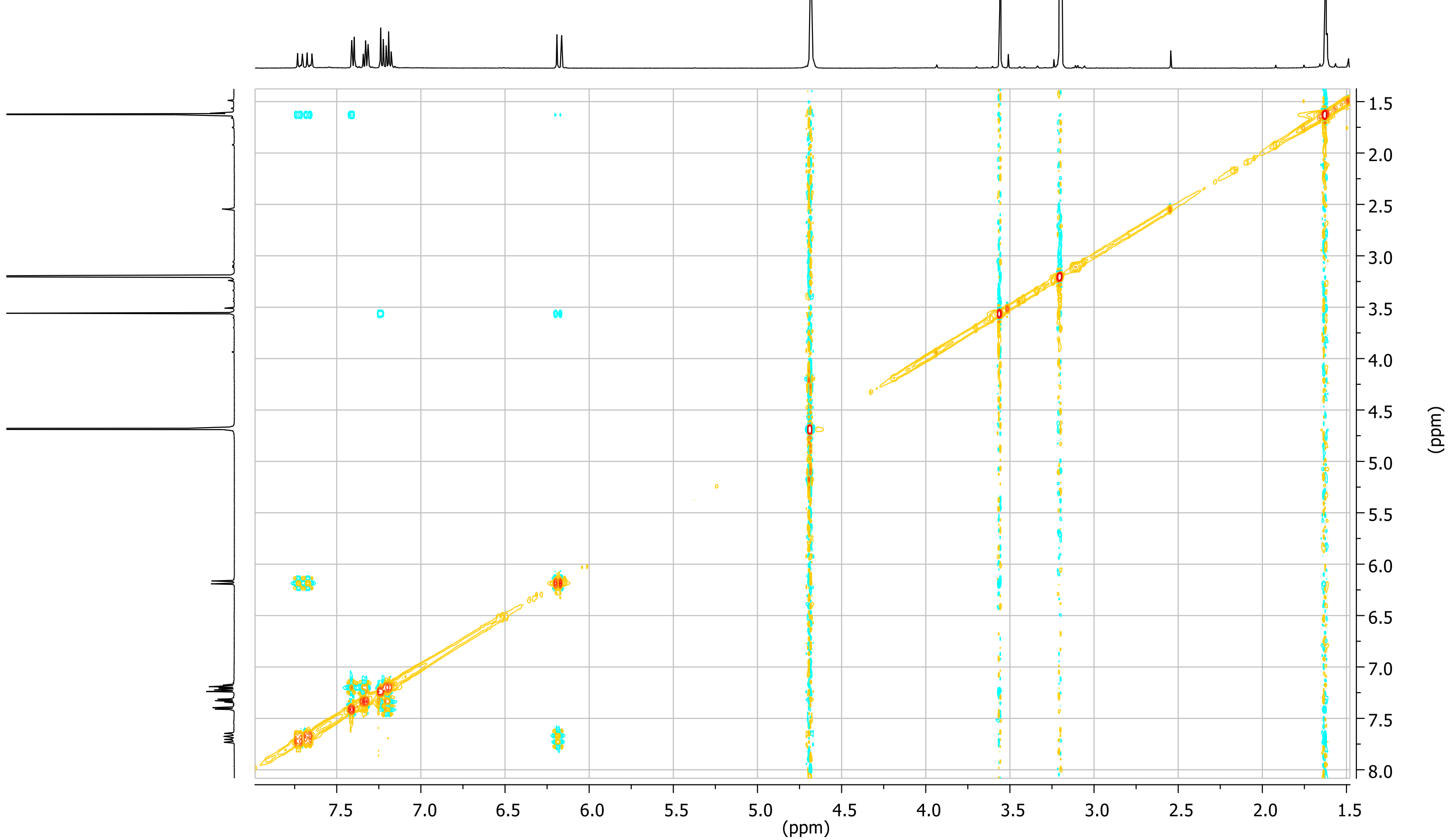

Figure S45. ^{1}H-^{1}H NOESY (500 MHz, *d*4–CD3OD): **2c.**

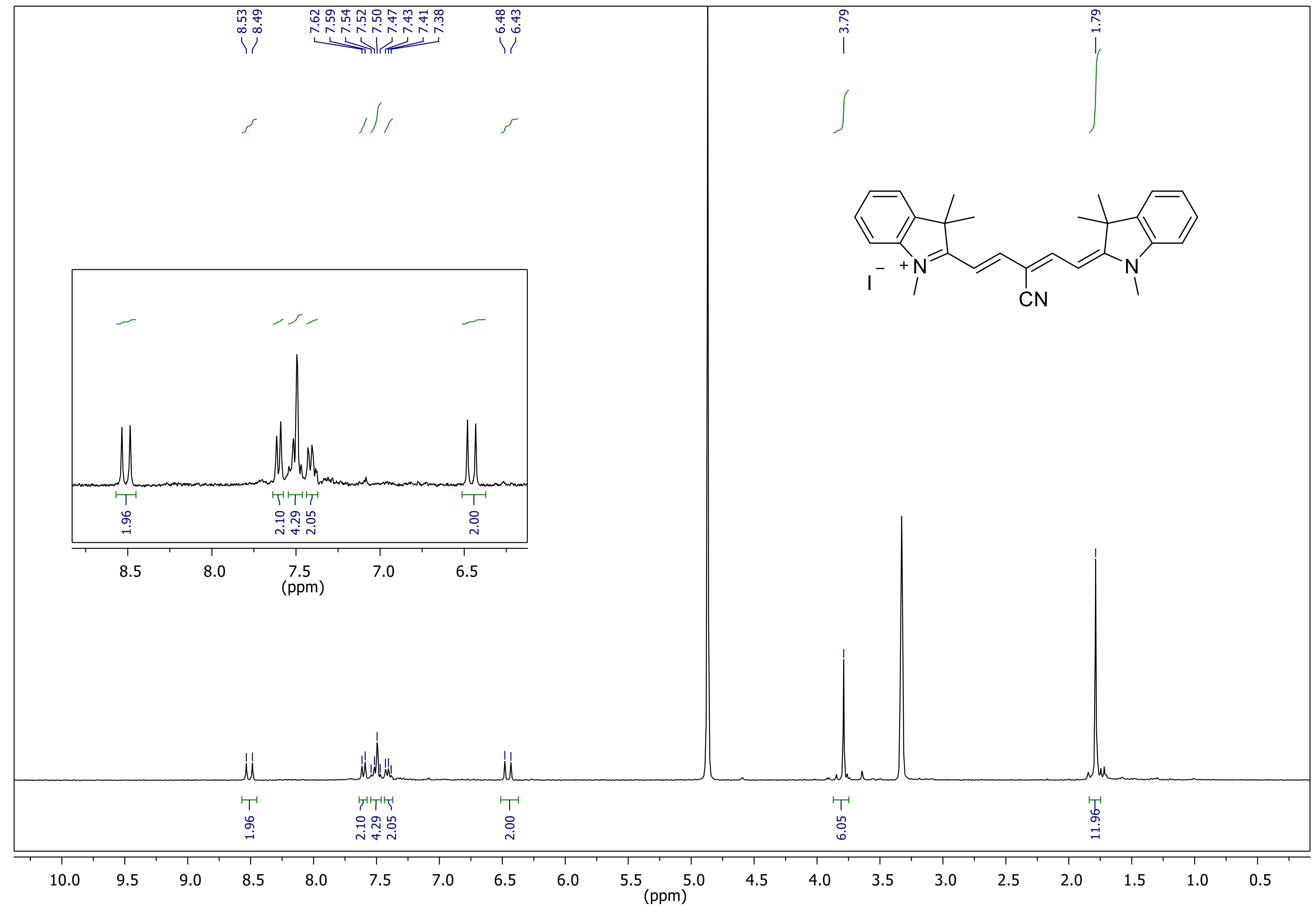

Figure S46. ^{1}H NMR (300 MHz, d_4–CD_3OD): **2e.**

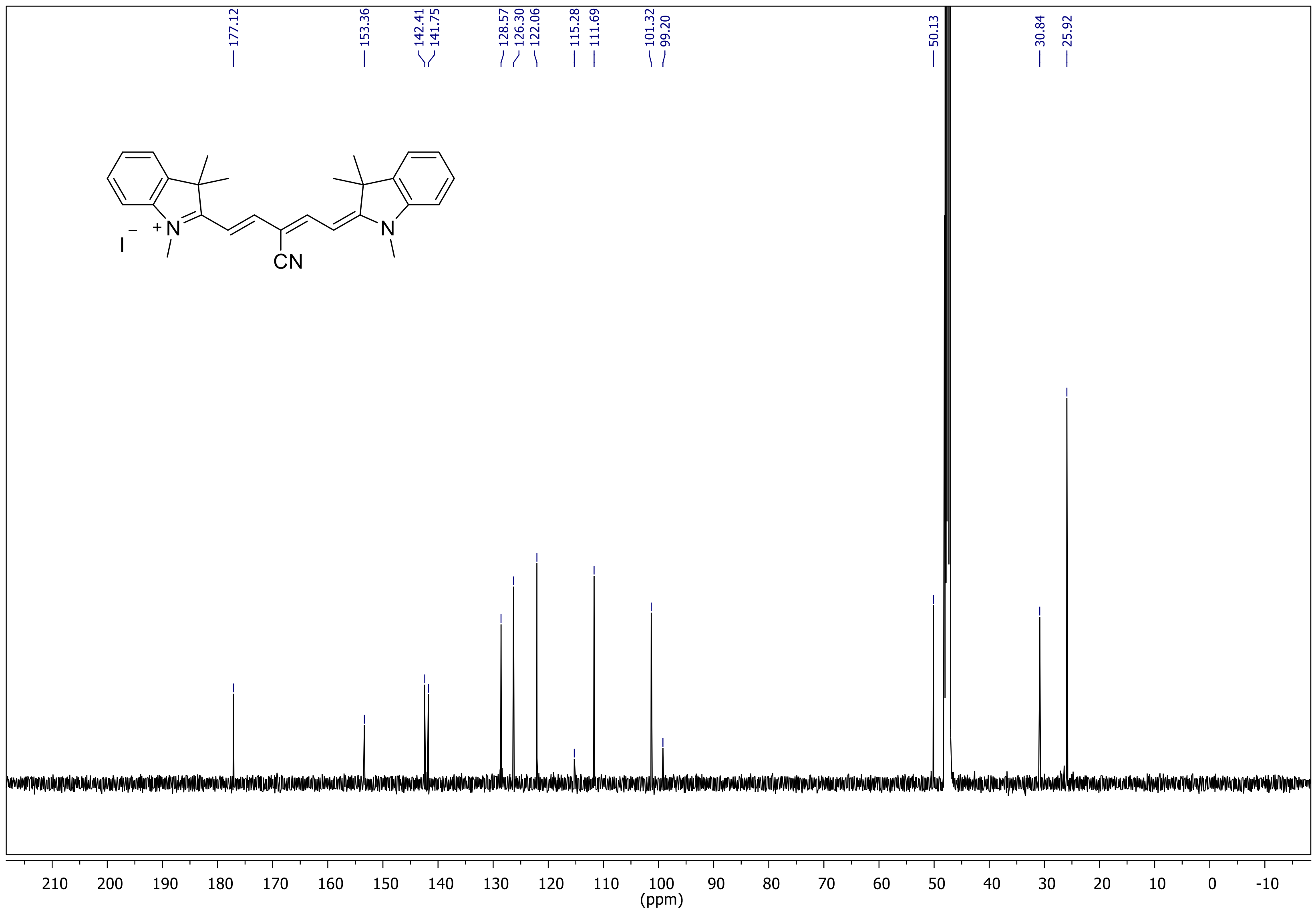

Figure S47. ^{13}C NMR (500 MHz, d_4–CD_3OD): **2e.**

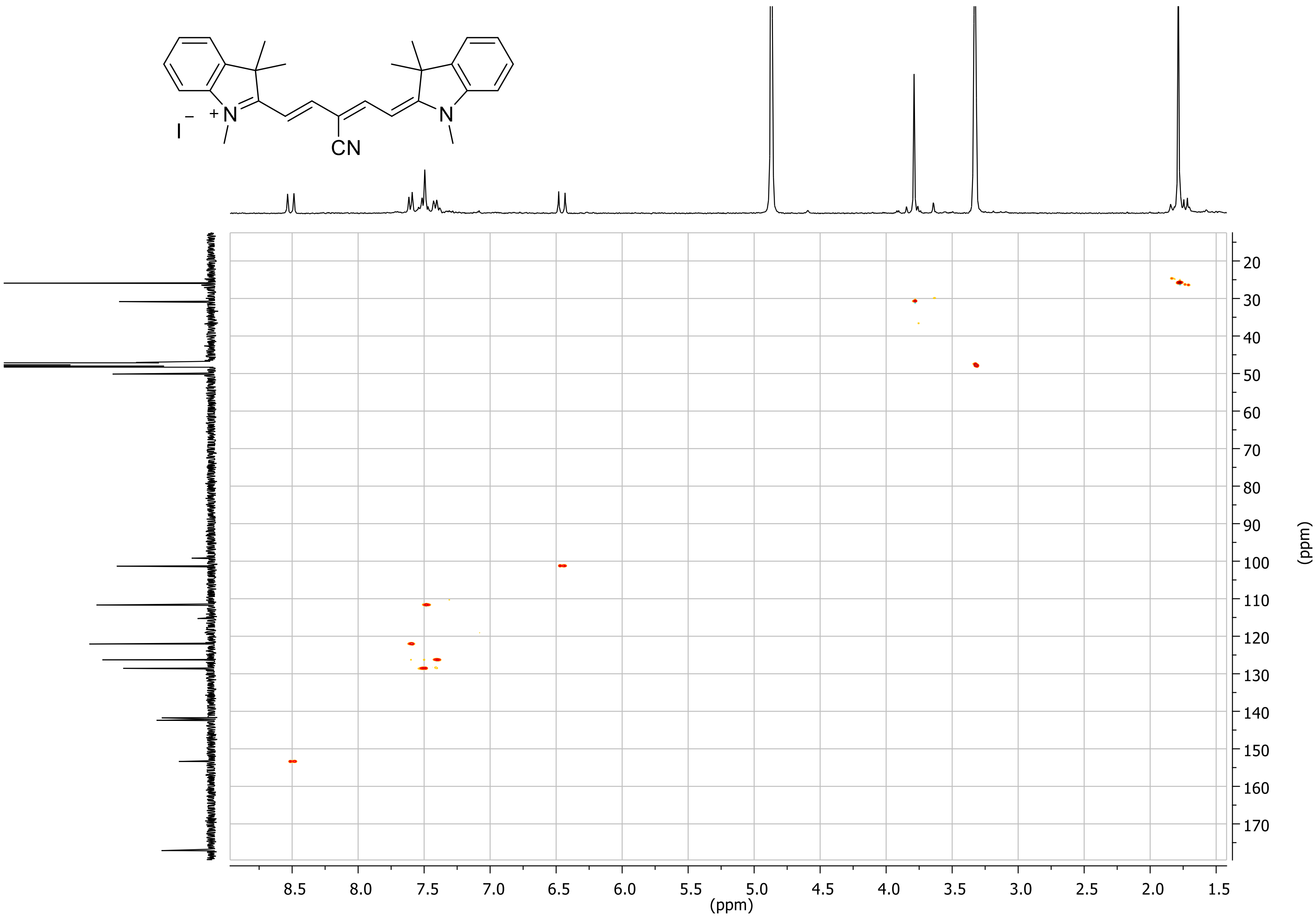

Figure S48. ^{1}H-^{13}C gHSQC (500 MHz, d_4–CD_3OD): **2e.**

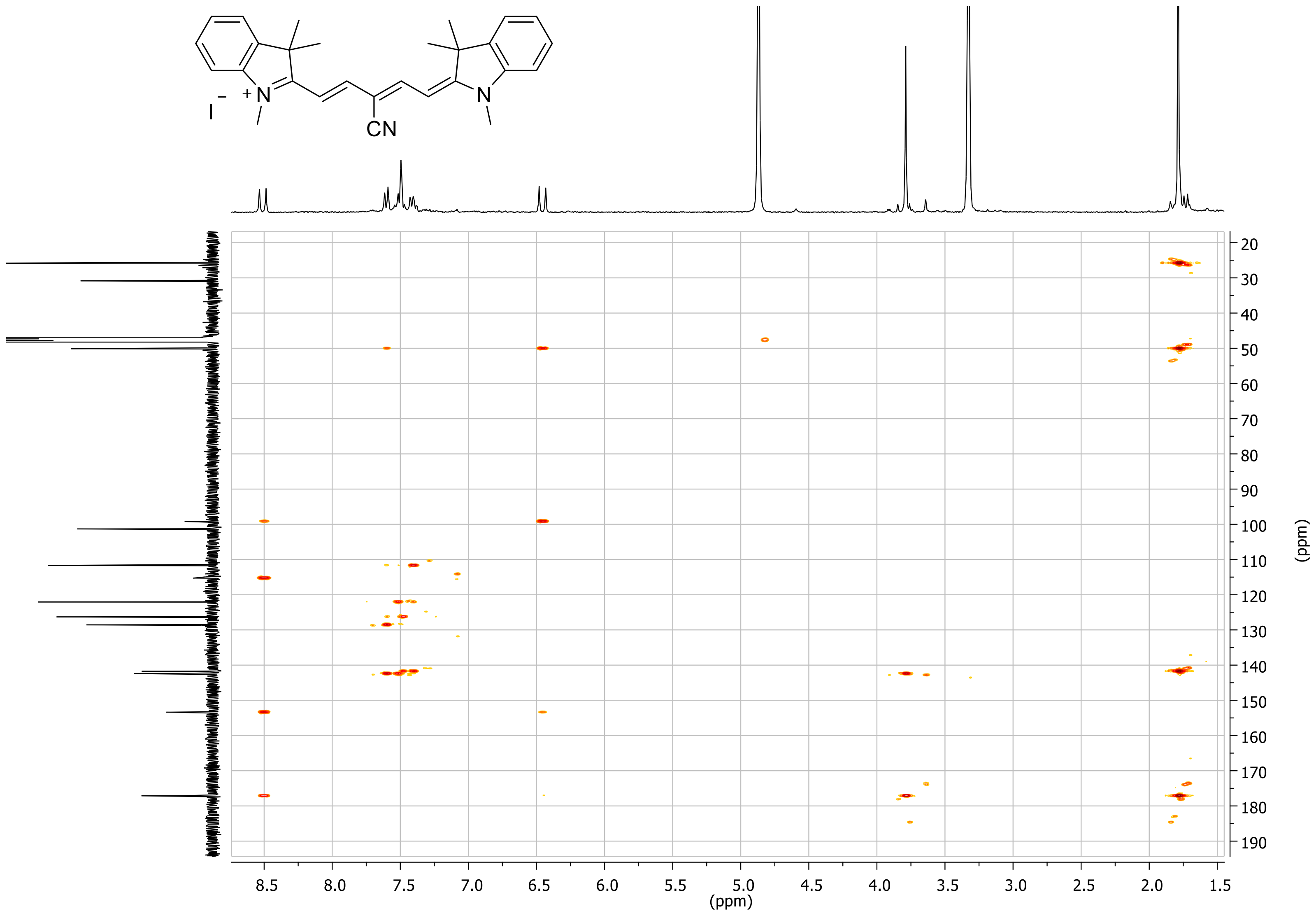

Figure S49. ^{1}H-^{13}C gHMBC (500 MHz, d_4–CD_3OD): **2e.**

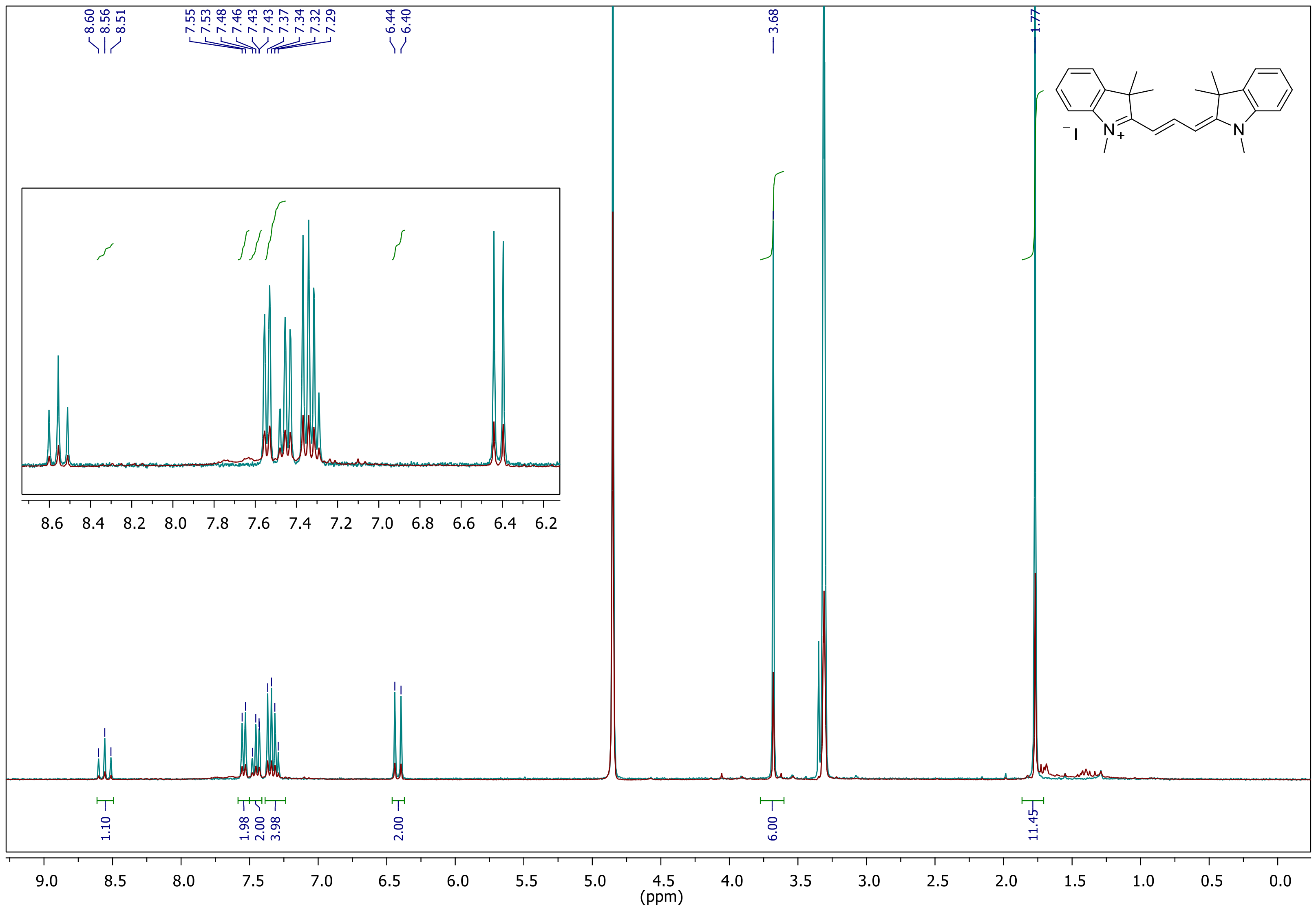

Figure S50. ^{1}H NMR spectra (MeOD). Green: independently synthesized **3a**. Red: **3a** isolated from the chain shortening reaction of **1c** with indolinium iodide **4A** (1 eq) and DIPA (2.5 eq) at 50 °C in acetonitrile after 21 h.

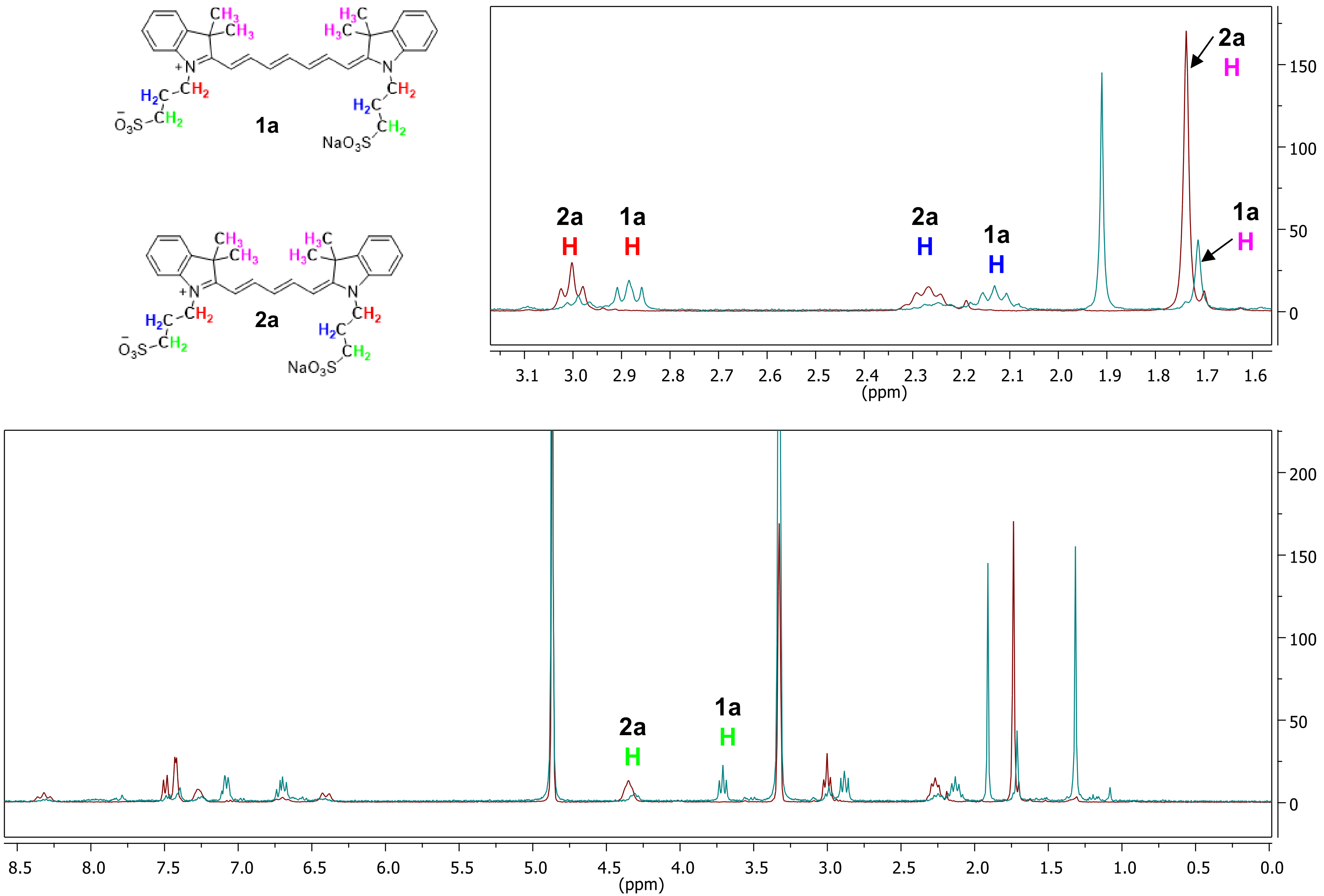

Figure S51. H NMR spectra. Red: independently synthesized **2a**. Green: reaction mixture of **2a** and **1a** observed after stirring of **8** and **5** in ethanol at 80°C for 24 h.

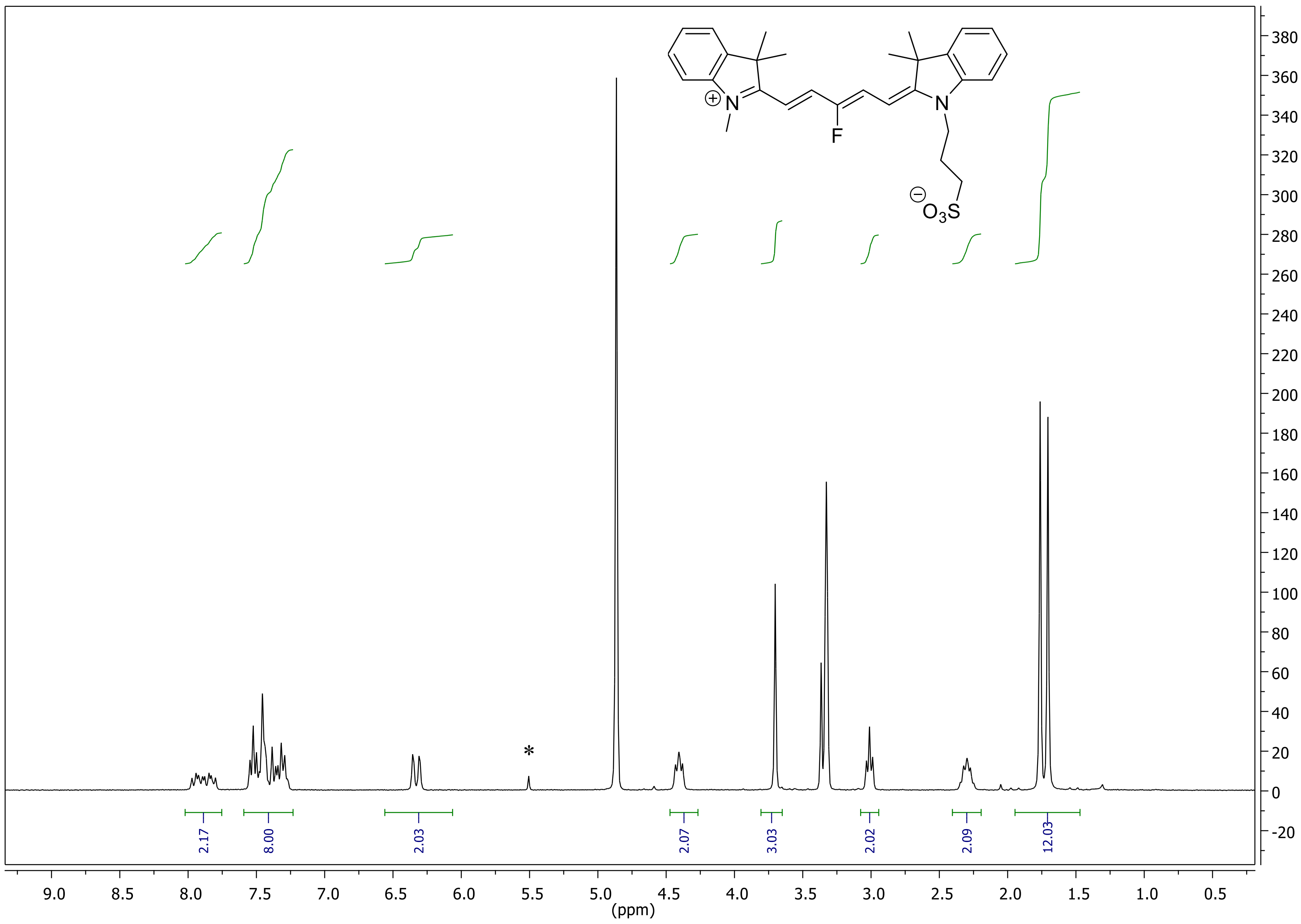

Figure S52. ^{1}H NMR (300 MHz, d_4–CD_3OD): **2i** (* DCM).

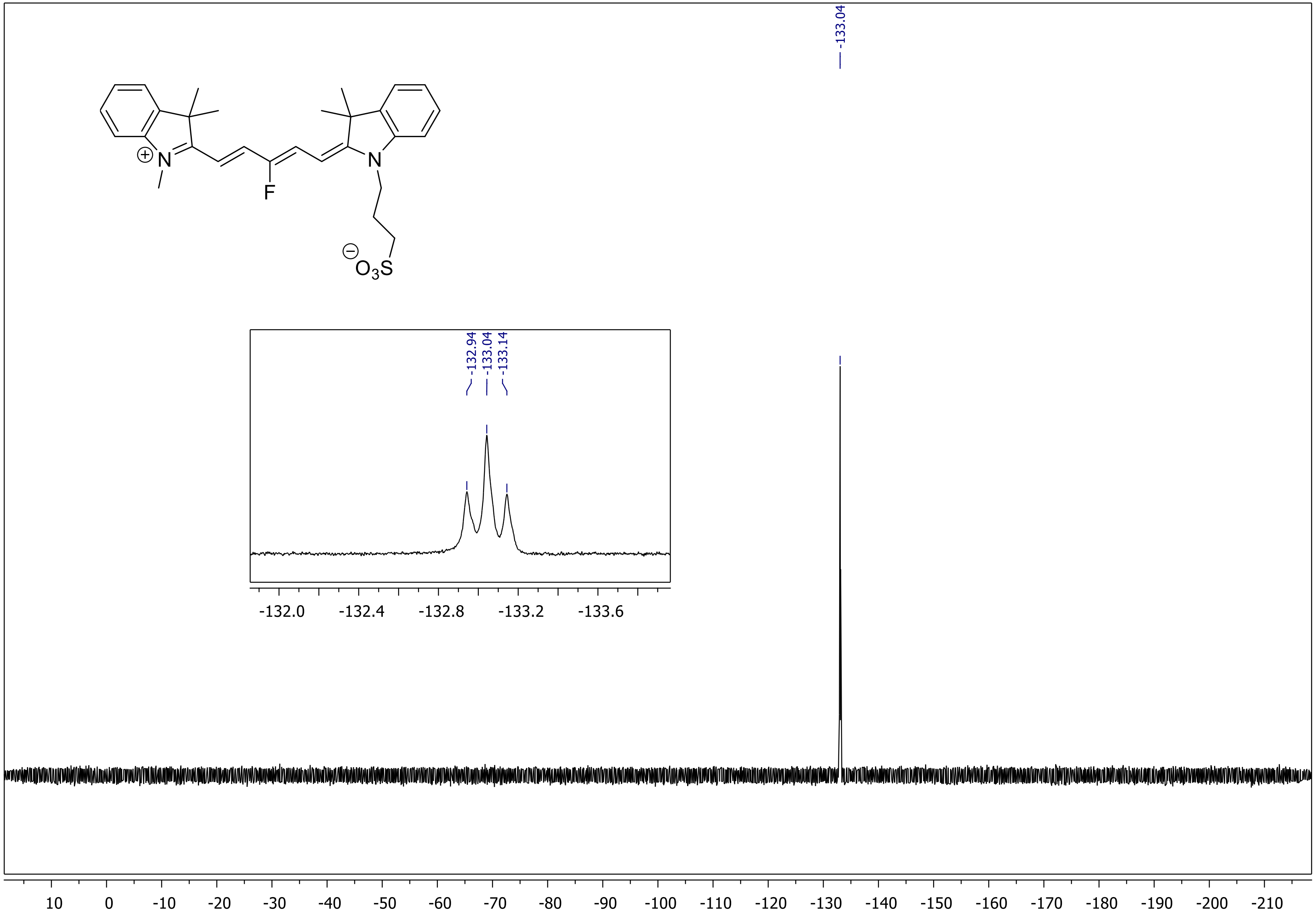

Figure S53. ^{19}F(H) NMR (300 MHz, d_4–CD_3OD): **2i.**

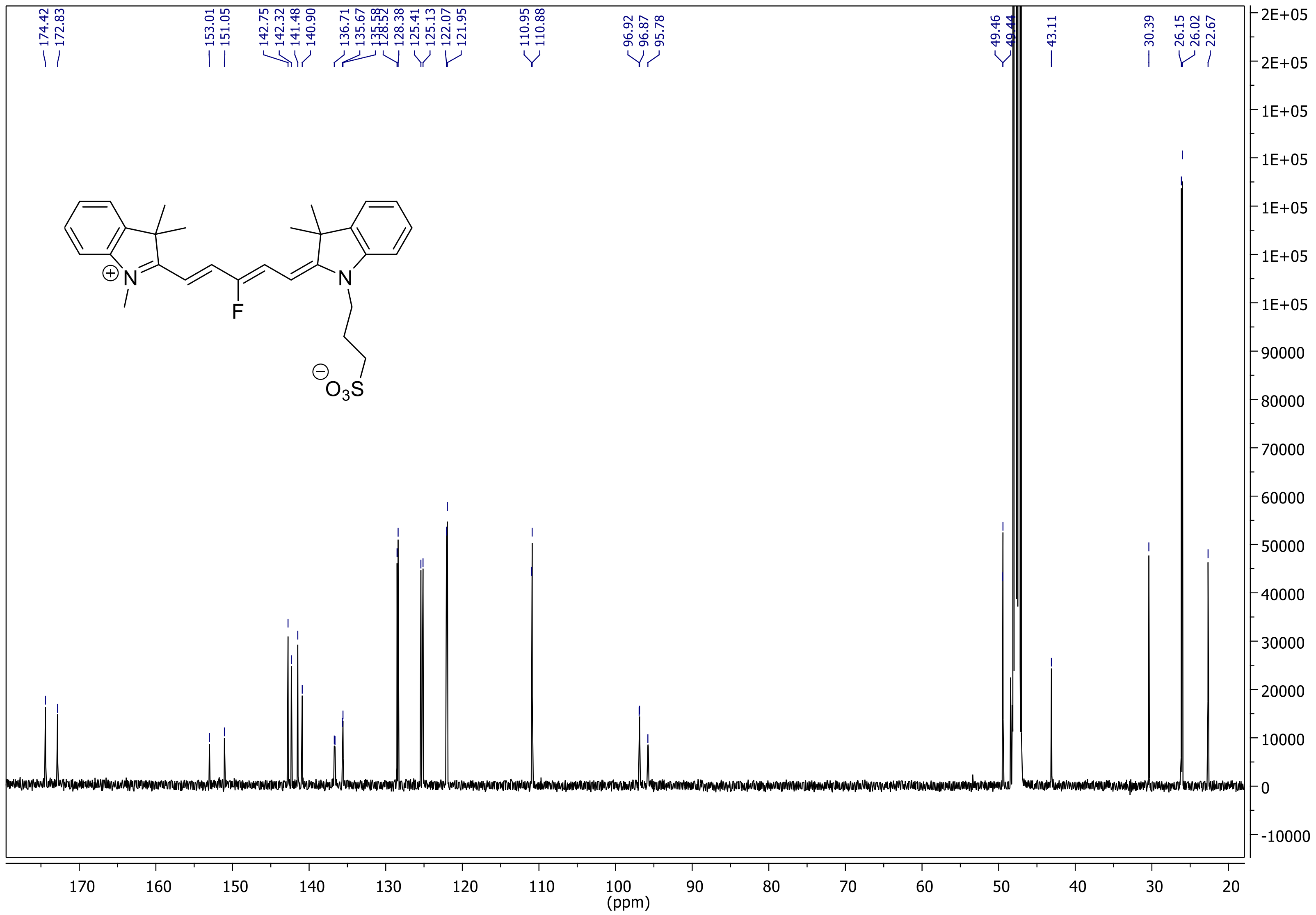

Figure S54. ^{13}C NMR (500 MHz, d_4–CD_3OD): **2i.**

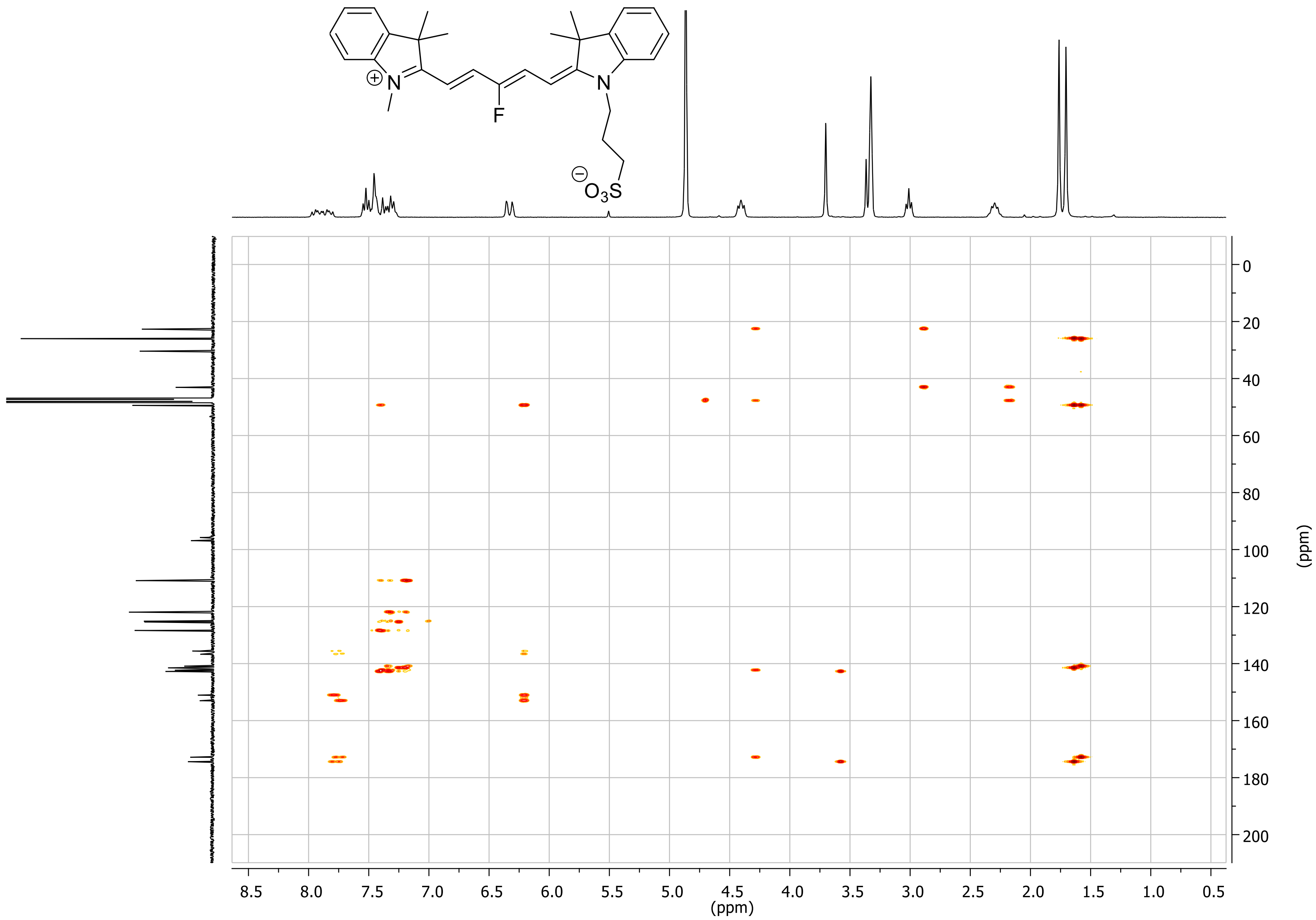

Figure S55. ^{1}H-^{13}C gHMBC (500 MHz, d_4–CD_3OD): **2i.**

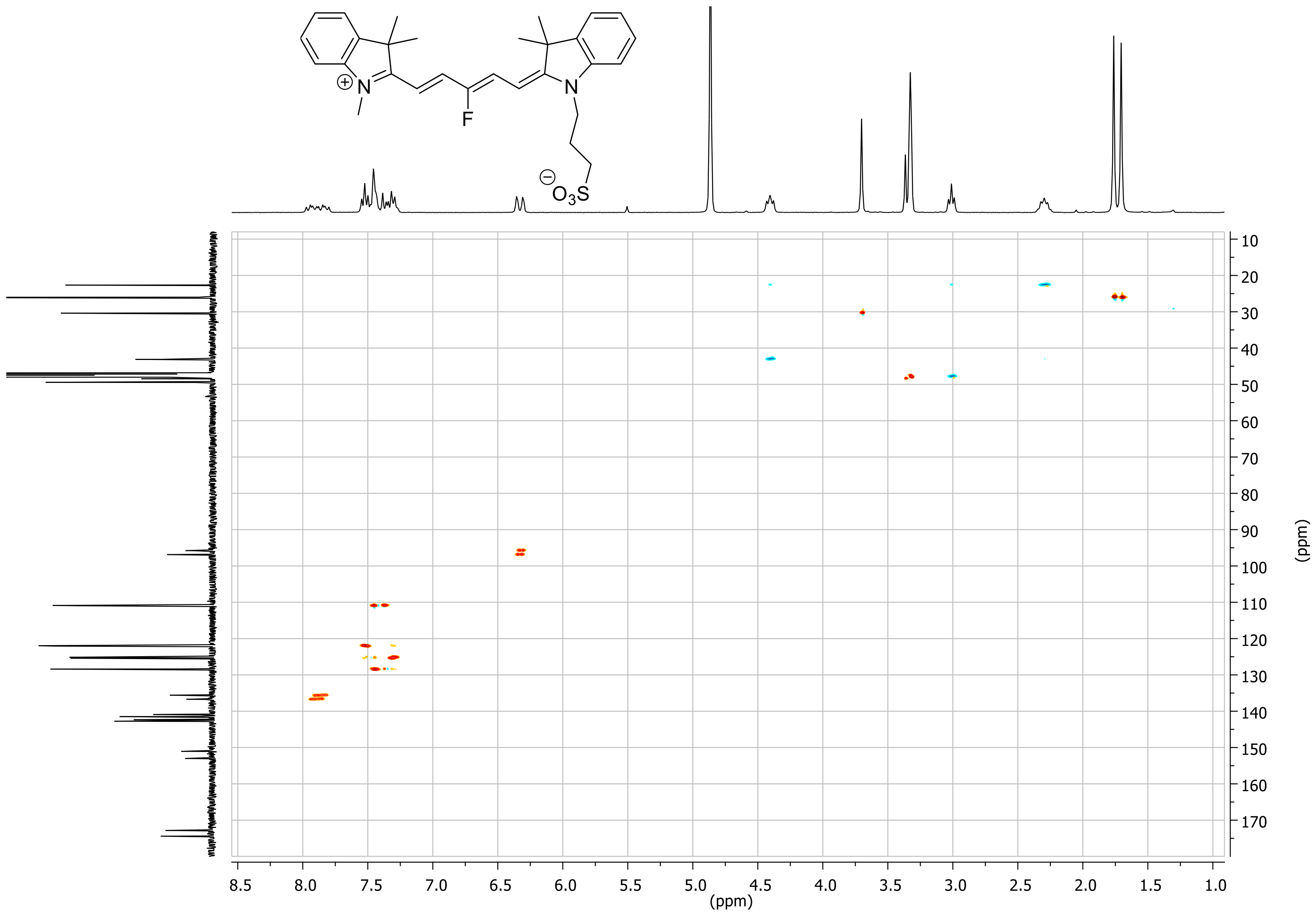

Figure S56. ^{1}H-^{13}C gHSQC (500 MHz, d_4–CD$_3$OD):): **2i.**

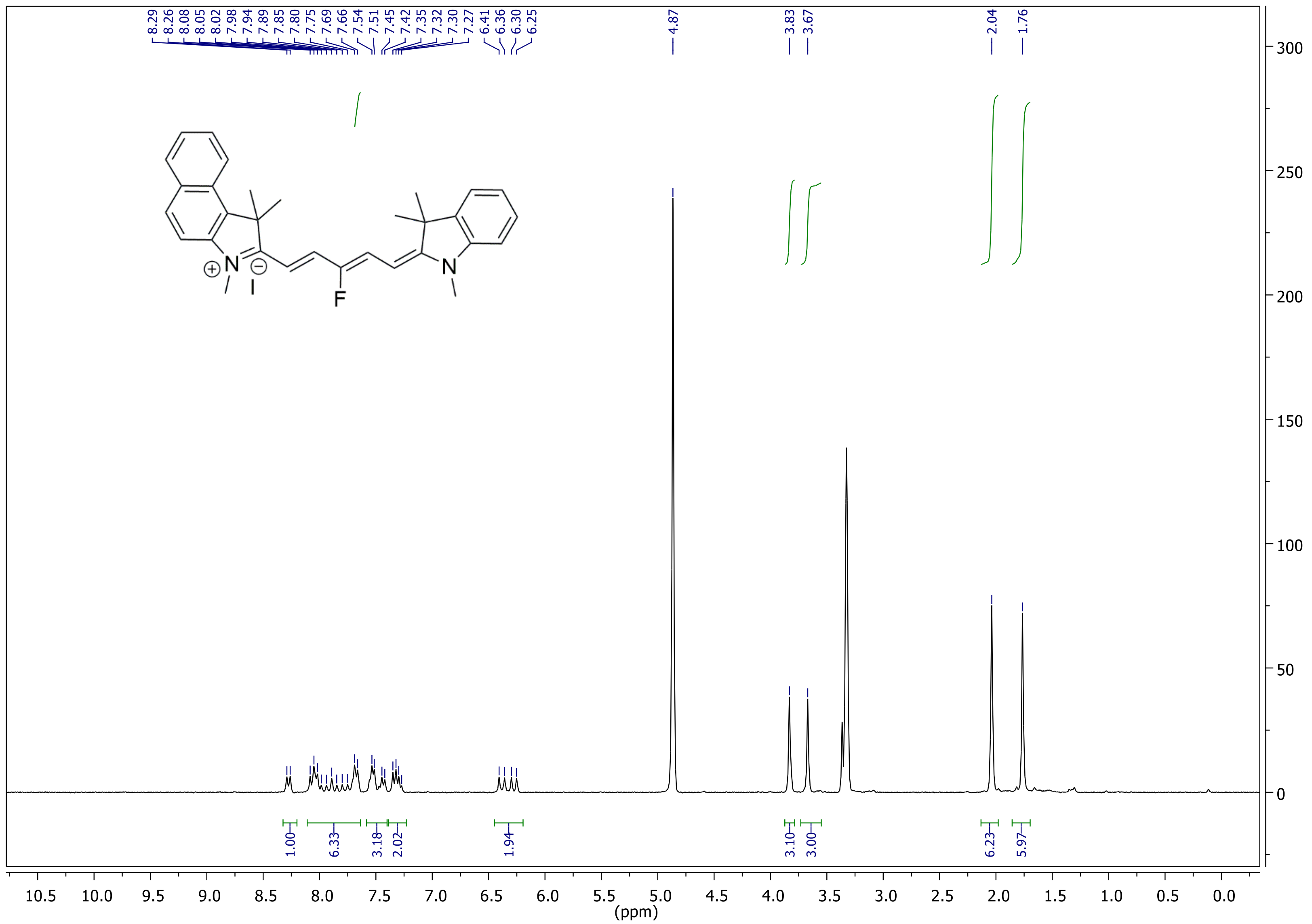

Figure S57. ^{1}H NMR (300 MHz, d_4–CD_3OD): **2k.**

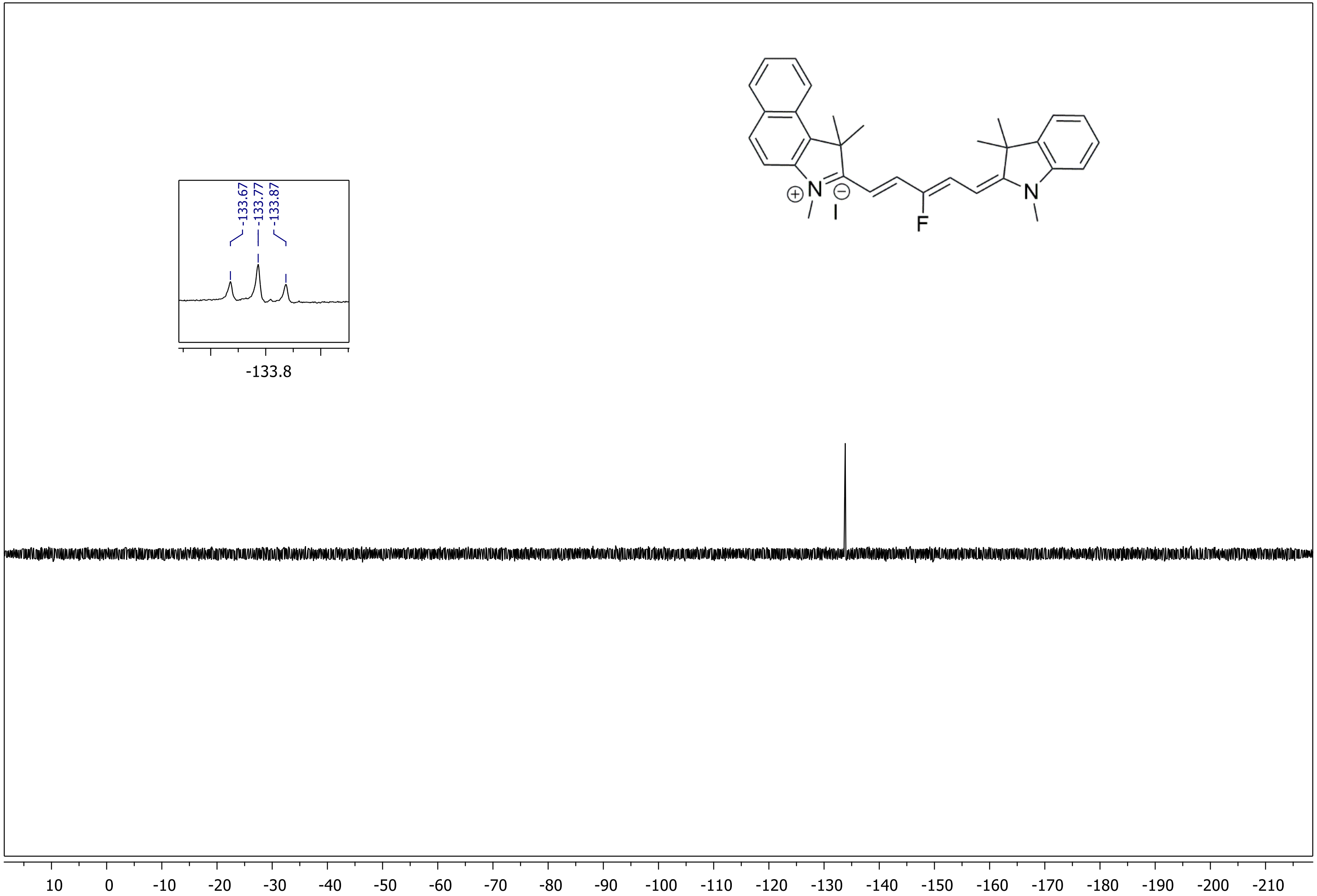

Figure S58. ^{19}F(H) NMR (300 MHz, d_4–CD_3OD): **2k.**

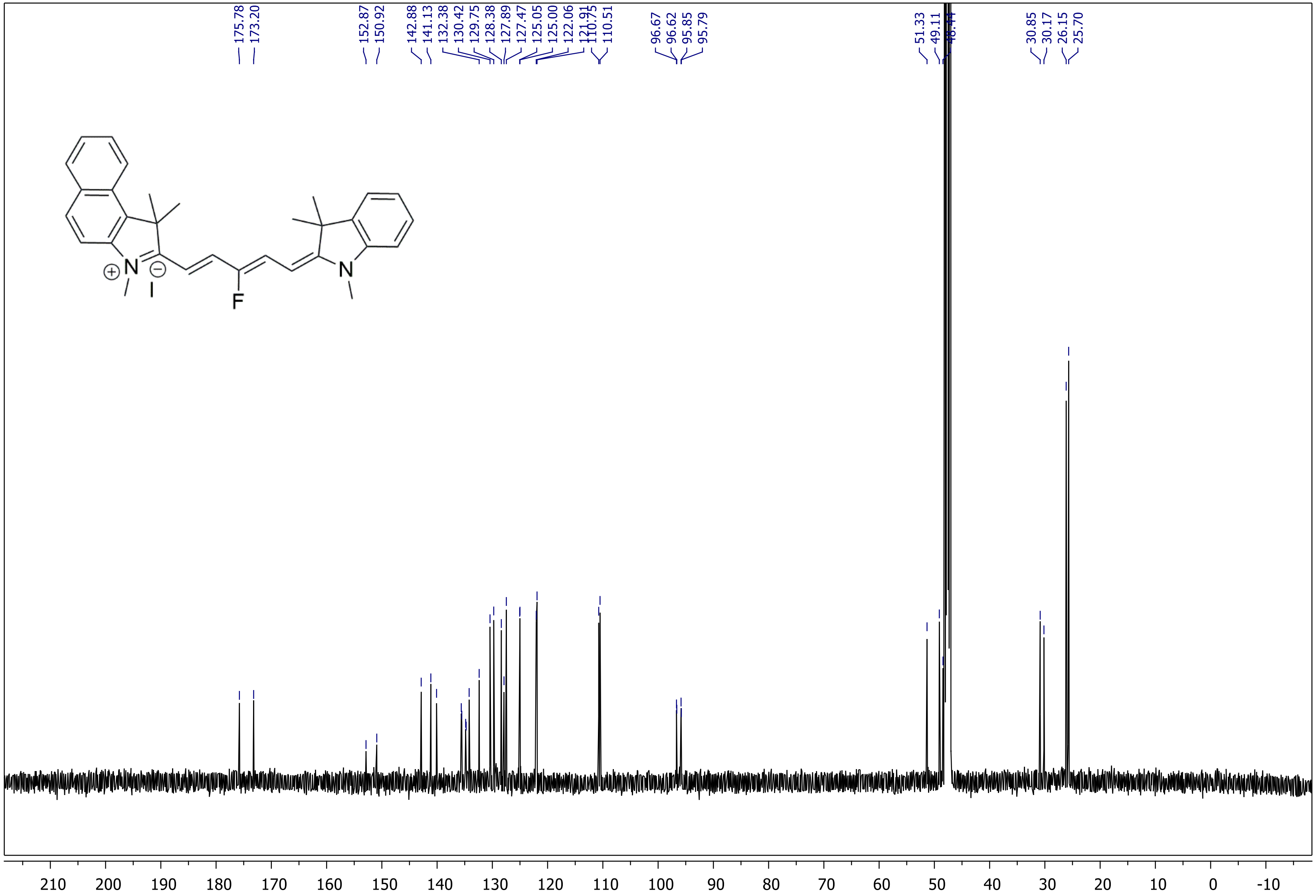

Figure S59. ^{13}C NMR (500 MHz, d_4–CD_3OD): **2k.**

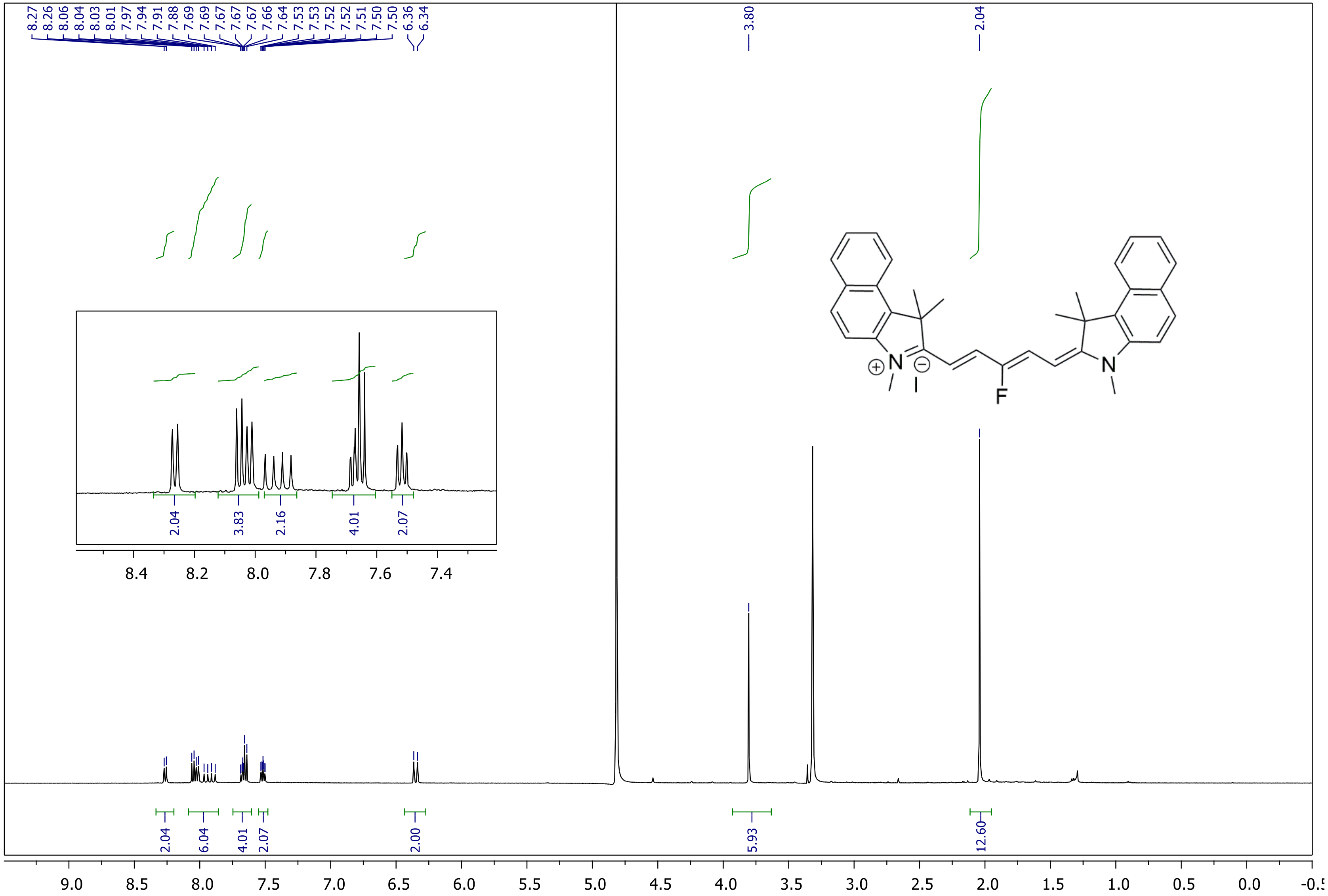

Figure S60. ^{1}H NMR (300 MHz, d_4–CD_3OD): **2l.**

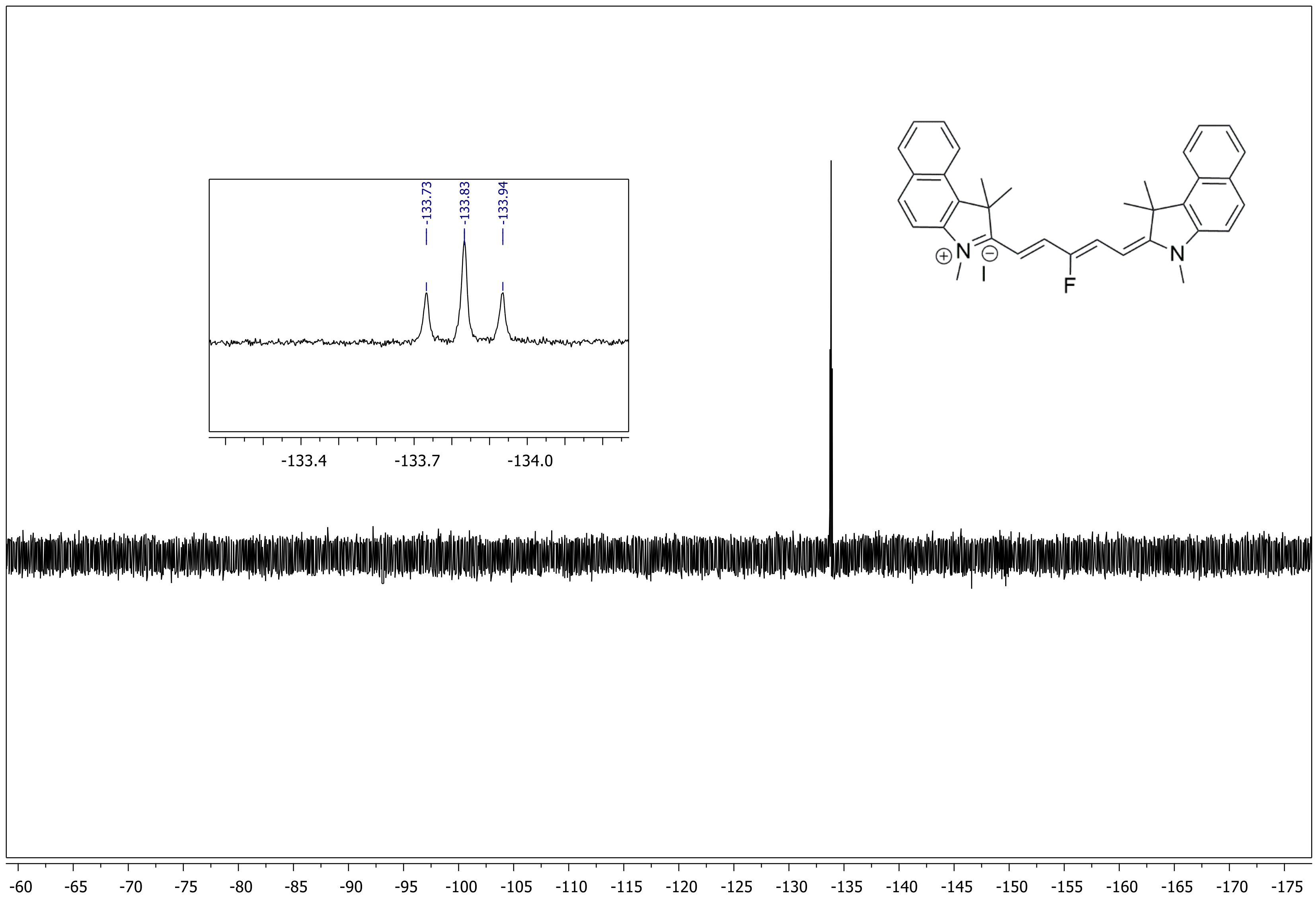

Figure S61. ^{19}F(H) NMR (300 MHz, d_4–CD_3OD): **2l.**

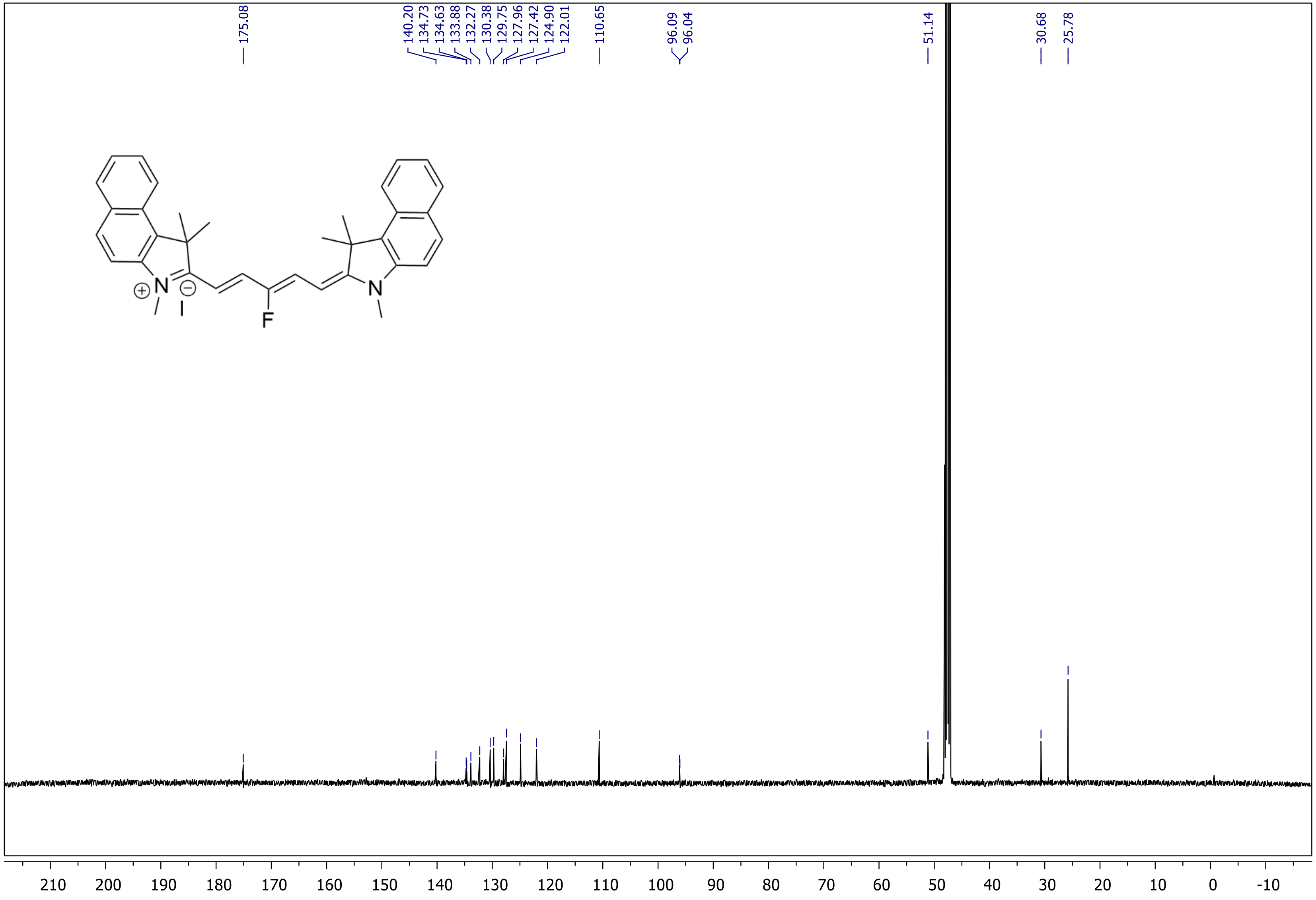

Figure S62. ^{13}C NMR (500 MHz, d_4–CD_3OD): **2l.**

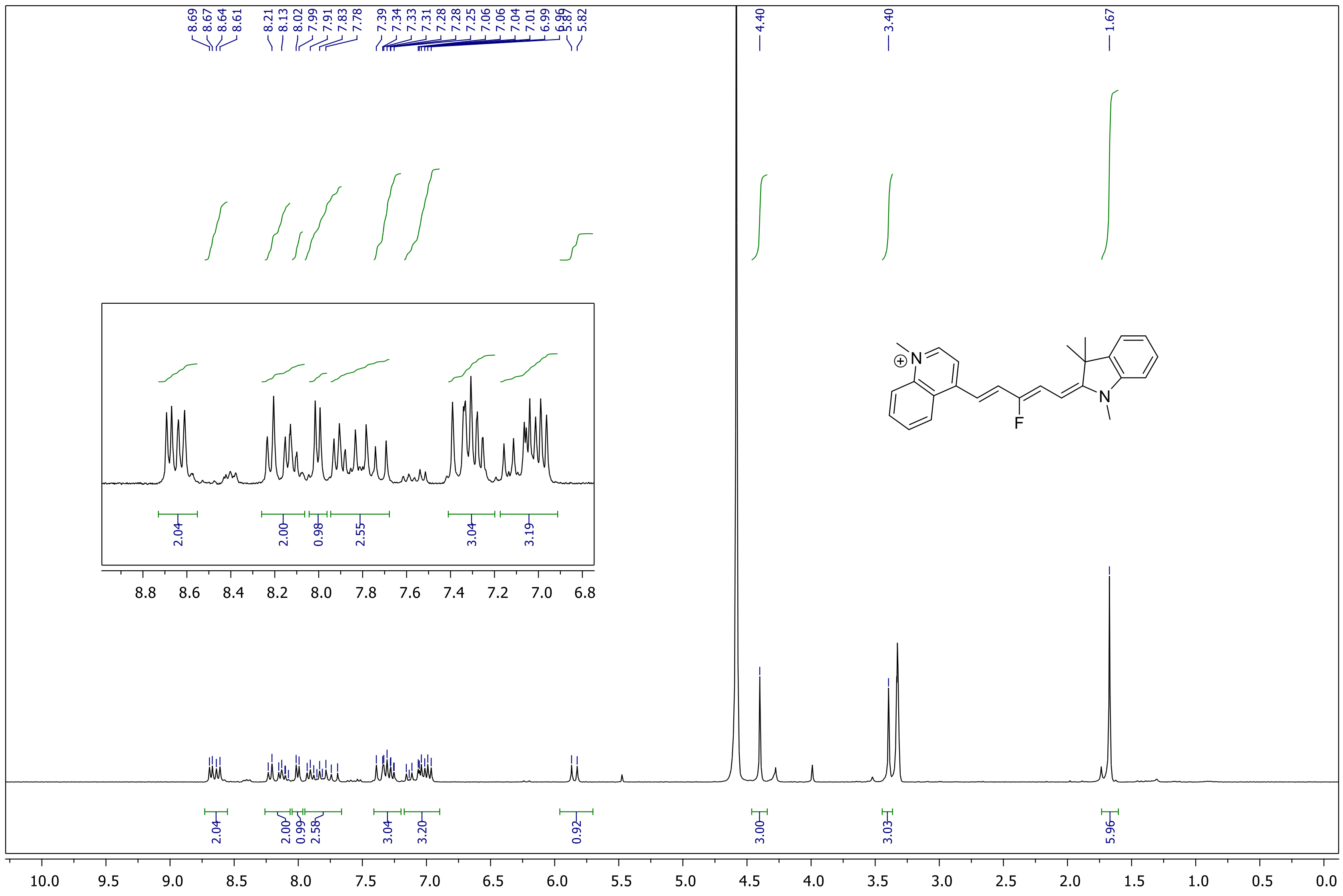

Figure S63. ^{1}H NMR (300 MHz, d_4–CD_3OD): **2m.**

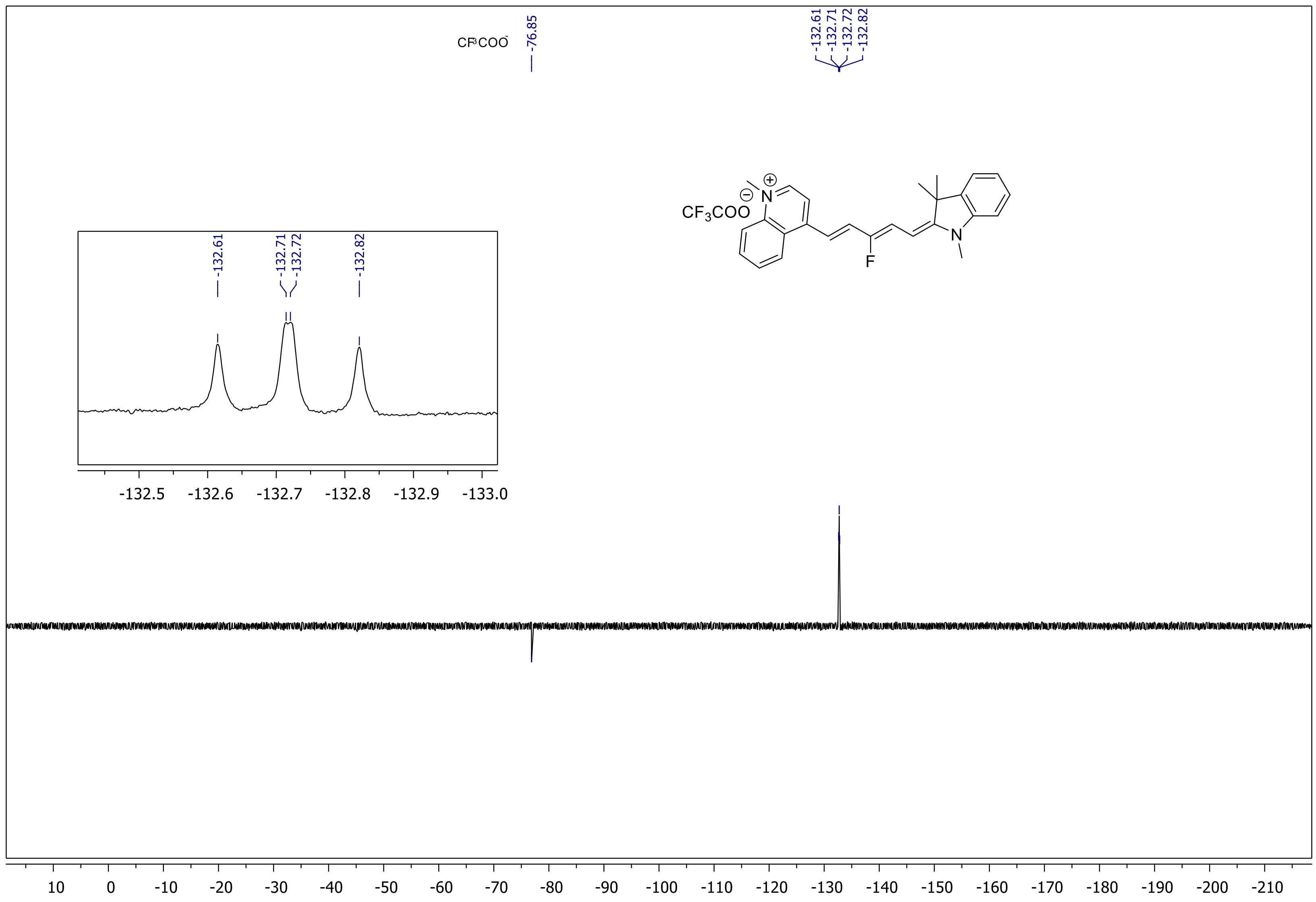

Figure S64.^{19}F(H) NMR (300 MHz, *d*4–CD_3OD): **2m.**

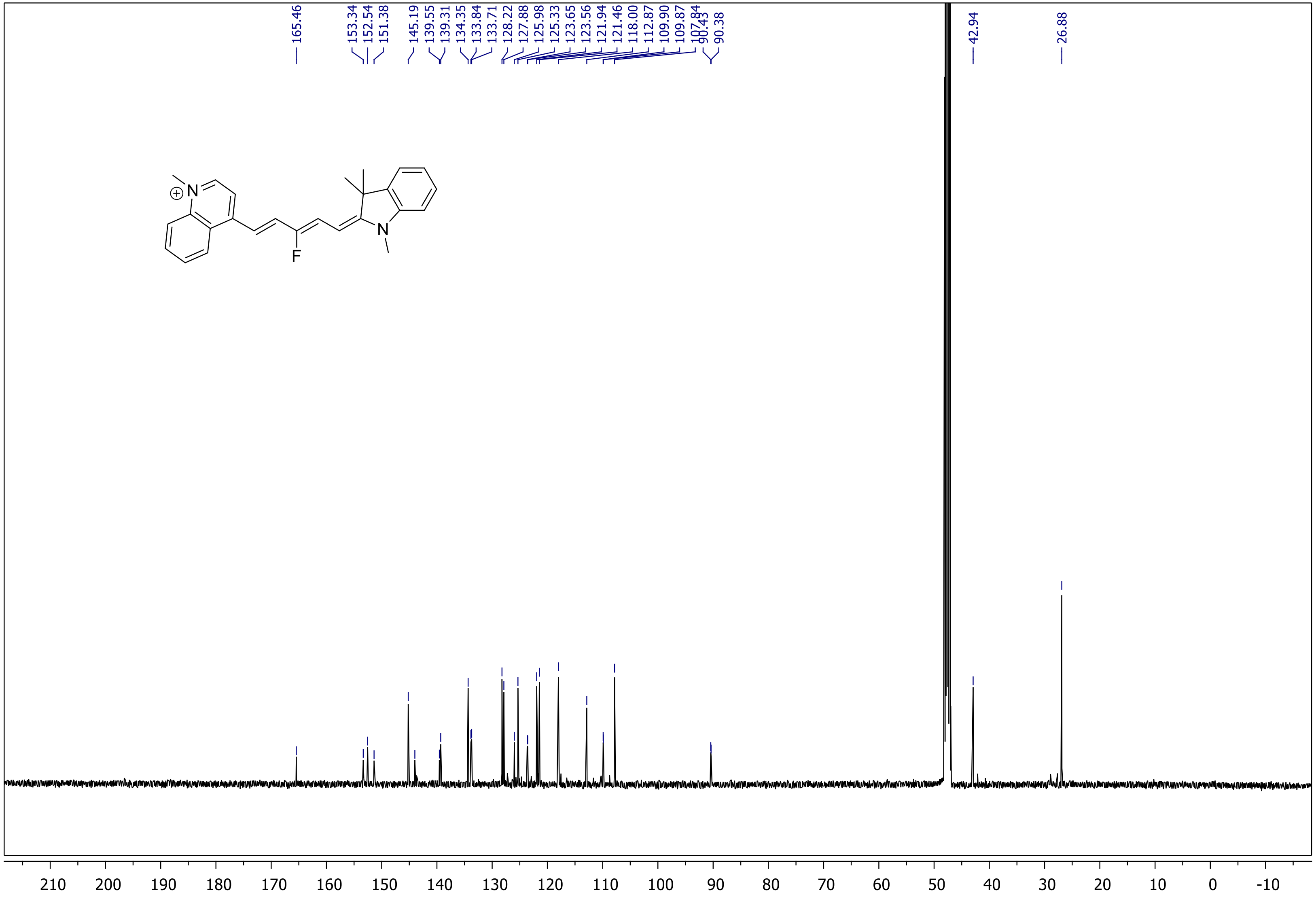

Figure S65. ^{13}C NMR (500 MHz, d_4–CD_3OD): **2m.**

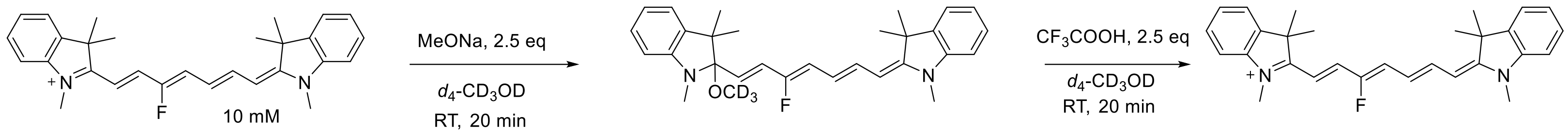

1c

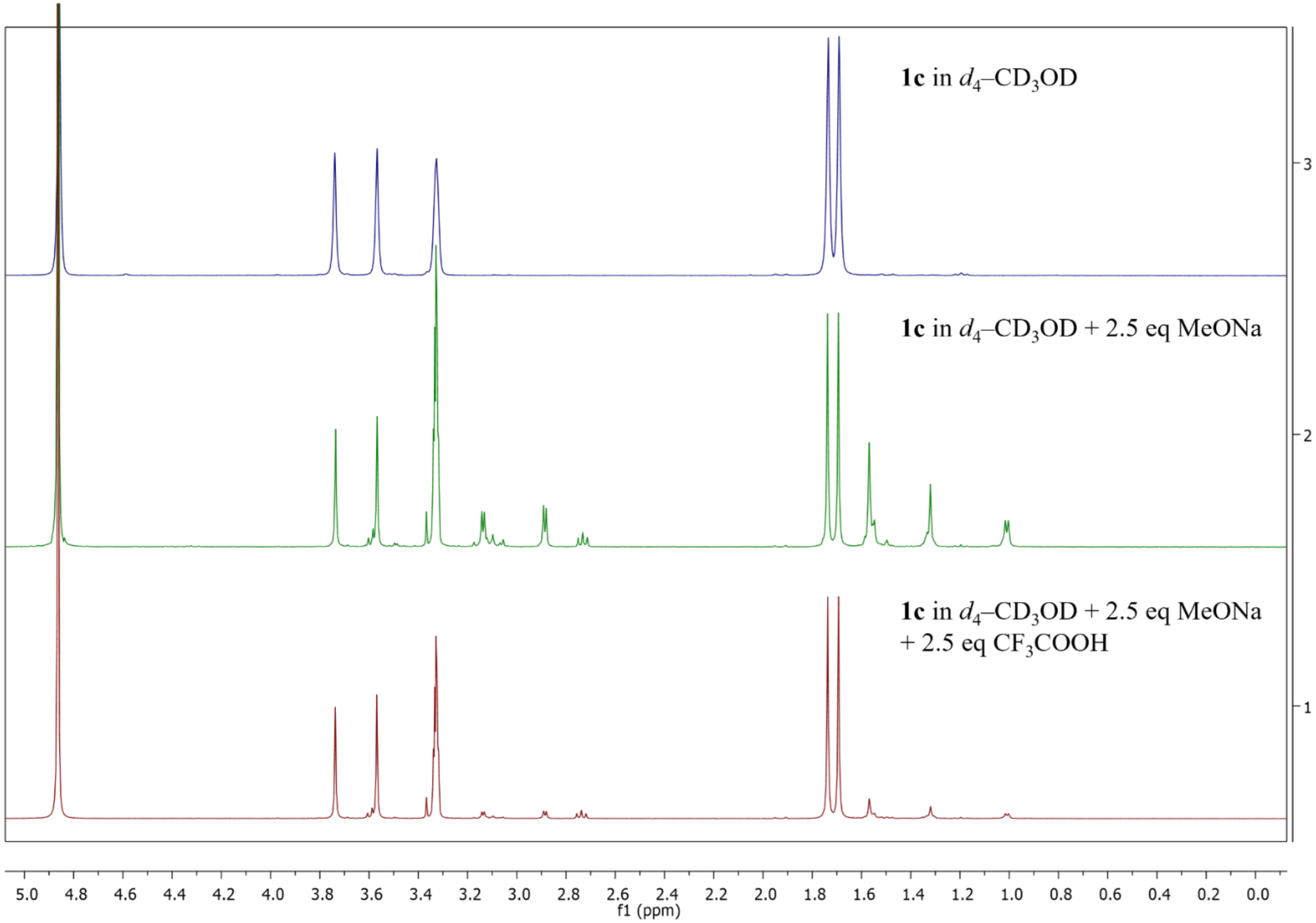

Figure S66. The addition of MeONa to **1c** (10 mM) in d_4–CD_3OD (^{1}H NMR, 300 MHz).

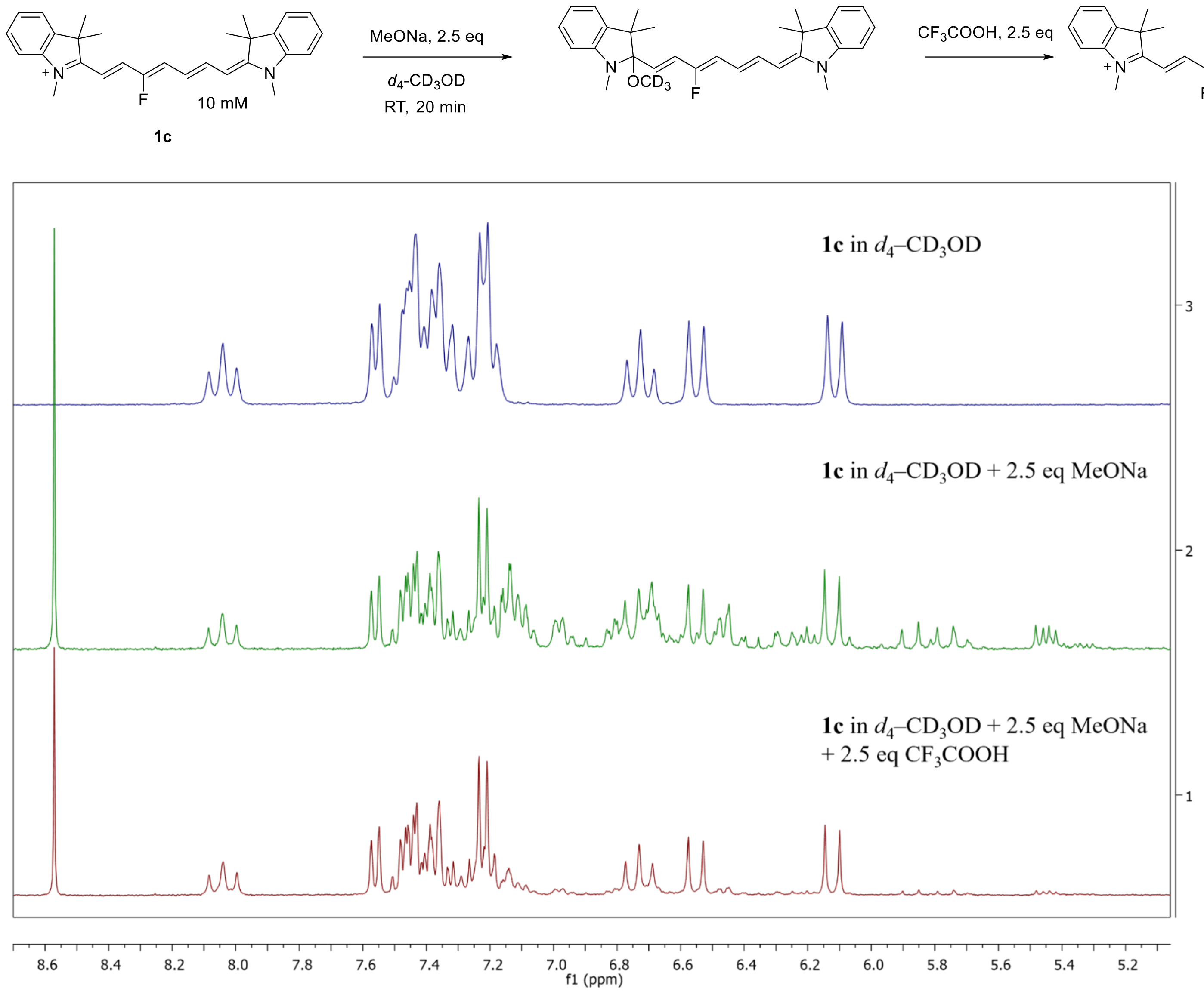

Figure S67. The reversible addition of MeONa to **1c** (10 mM) in d_4–CD_3OD (^{1}H NMR, 300 MHz).

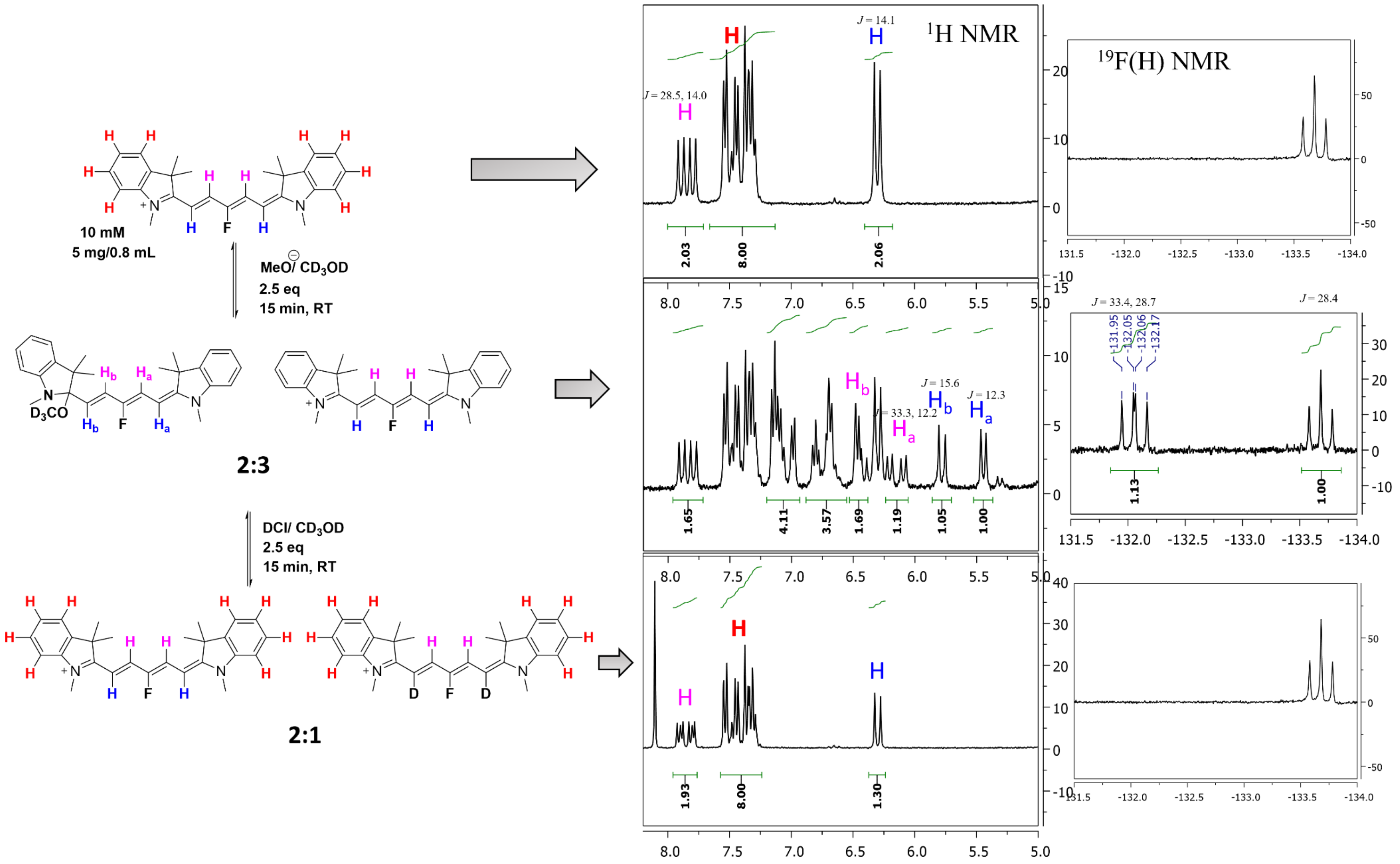

Figure S68. The reversible addition of MeONa to **3c** in *d*4–CD3OD ([1] H NMR, 300 MHz).

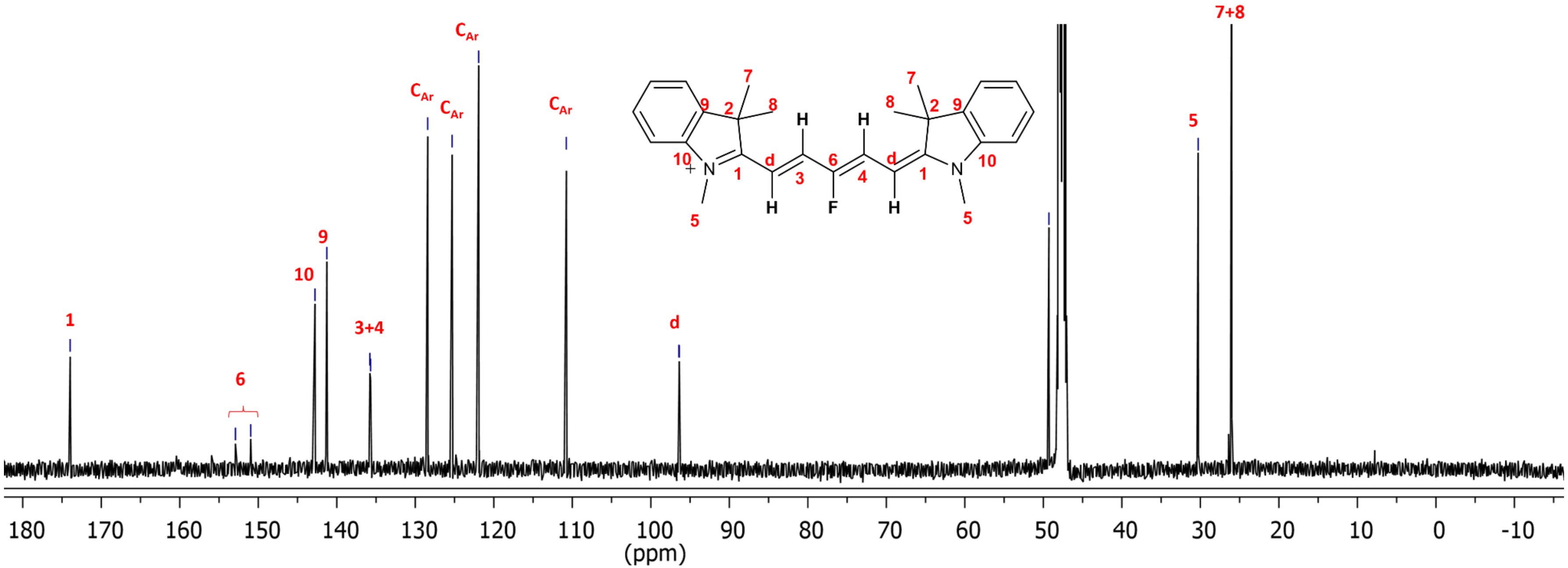

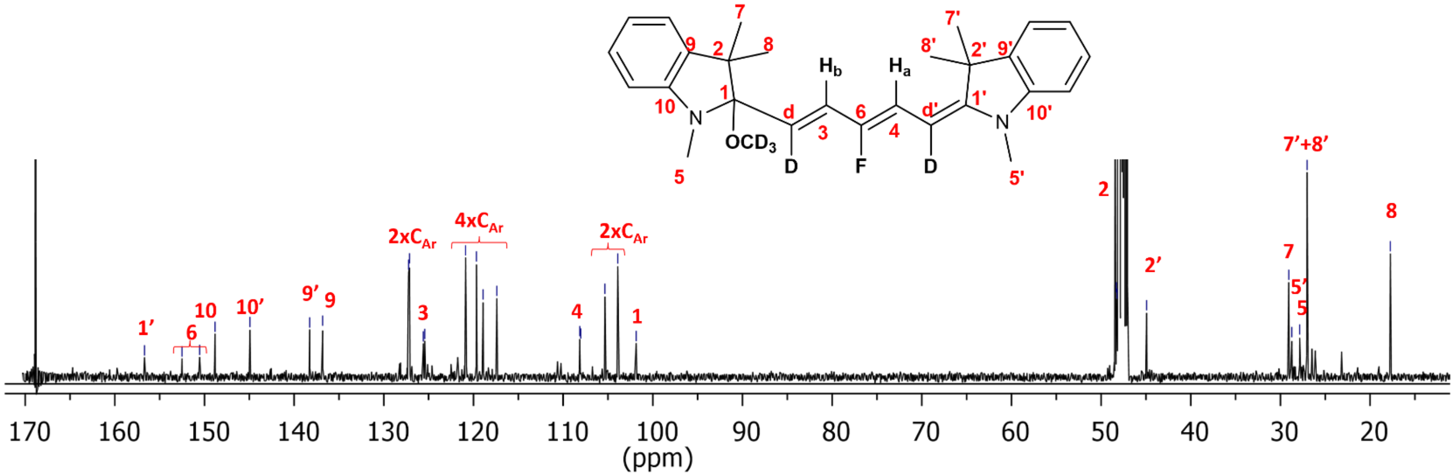

Figure S69. The addition of MeONa to **3c** in *d*$_4$–CD_3OD (^{13}C NMR, 300 MHz).

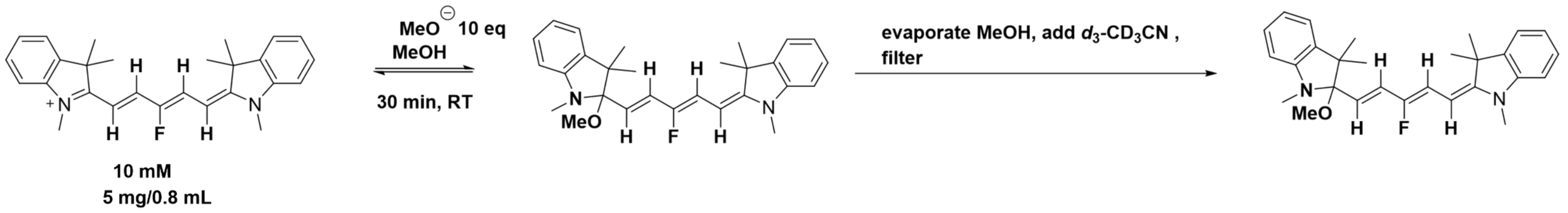

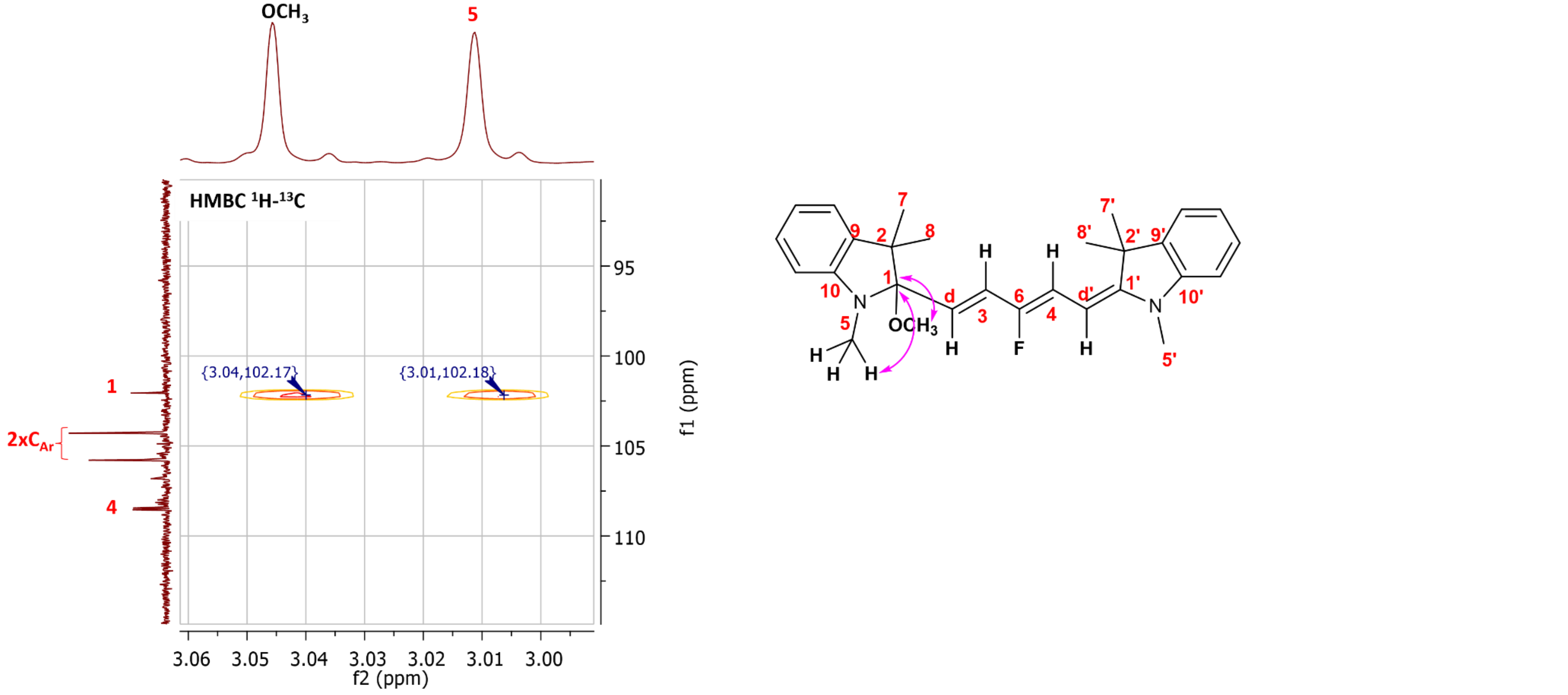

Figure S70. The addition of MeONa to **3c** in d_3–CD_3CN (^1H-^{13}C gHMBC, 500 MHz).

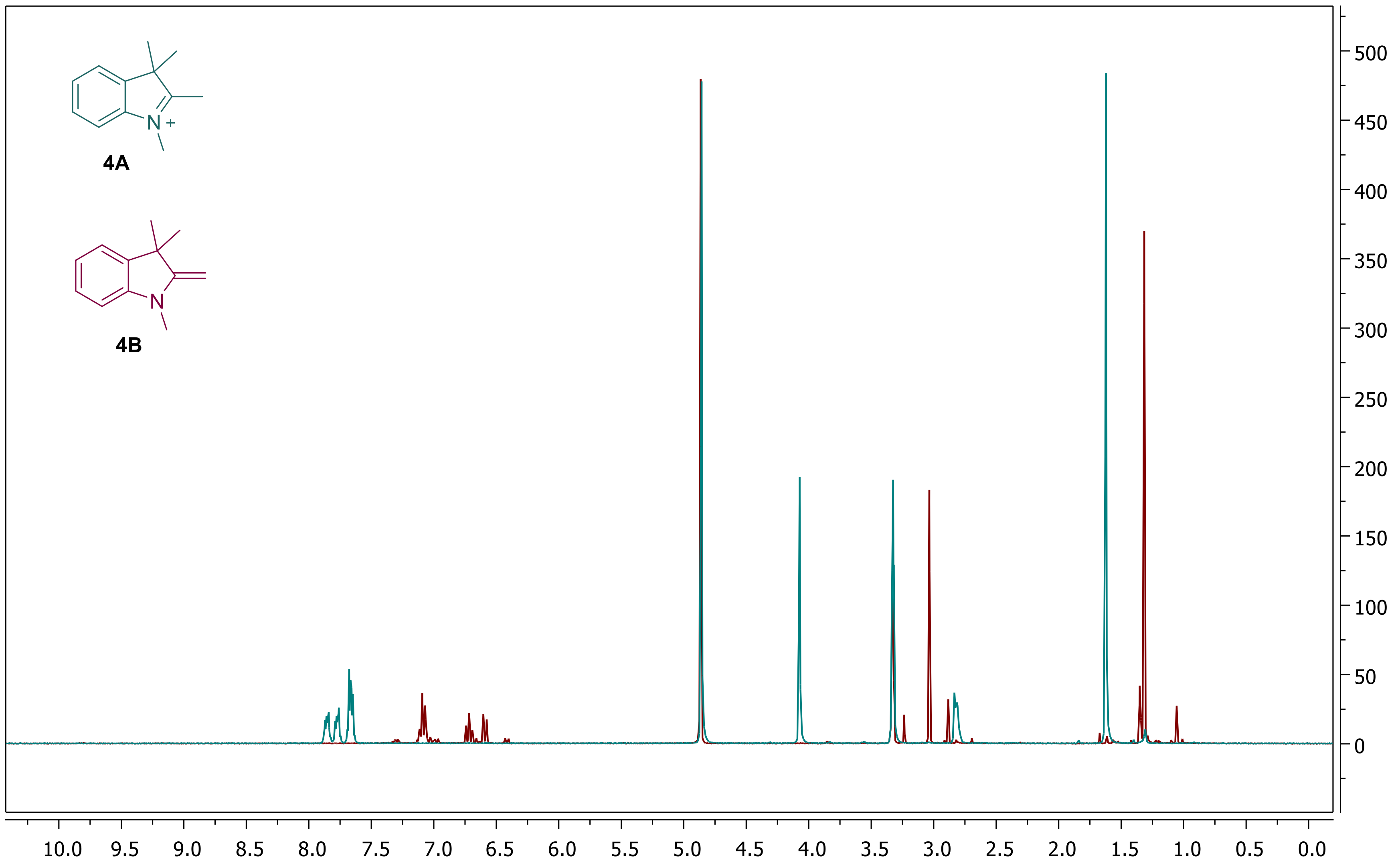

Figure S71. ^{1}H NMR (300 MHz, d_4–CD_3OD): **4A** (green) and **4B** (red).

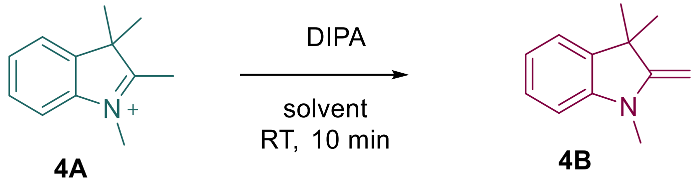

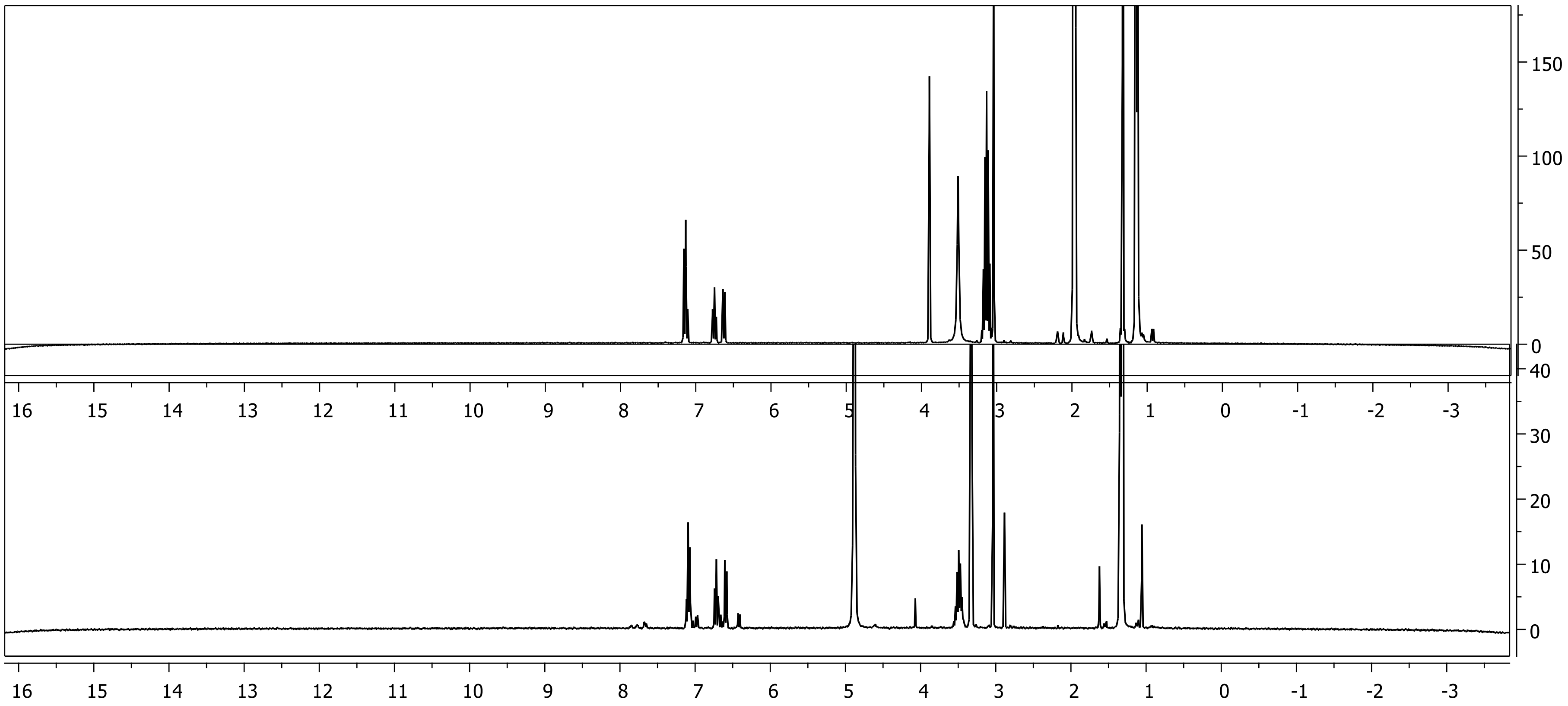

Figure S72. ^{1}H NMR (300 MHz): **4A** reacted with 1 eq DIPA in d_4–CD_3OD (bottom) and with 2 eq DIPA in d_3–CD_3CN (top). The spectra indicate the formation of **4B**.

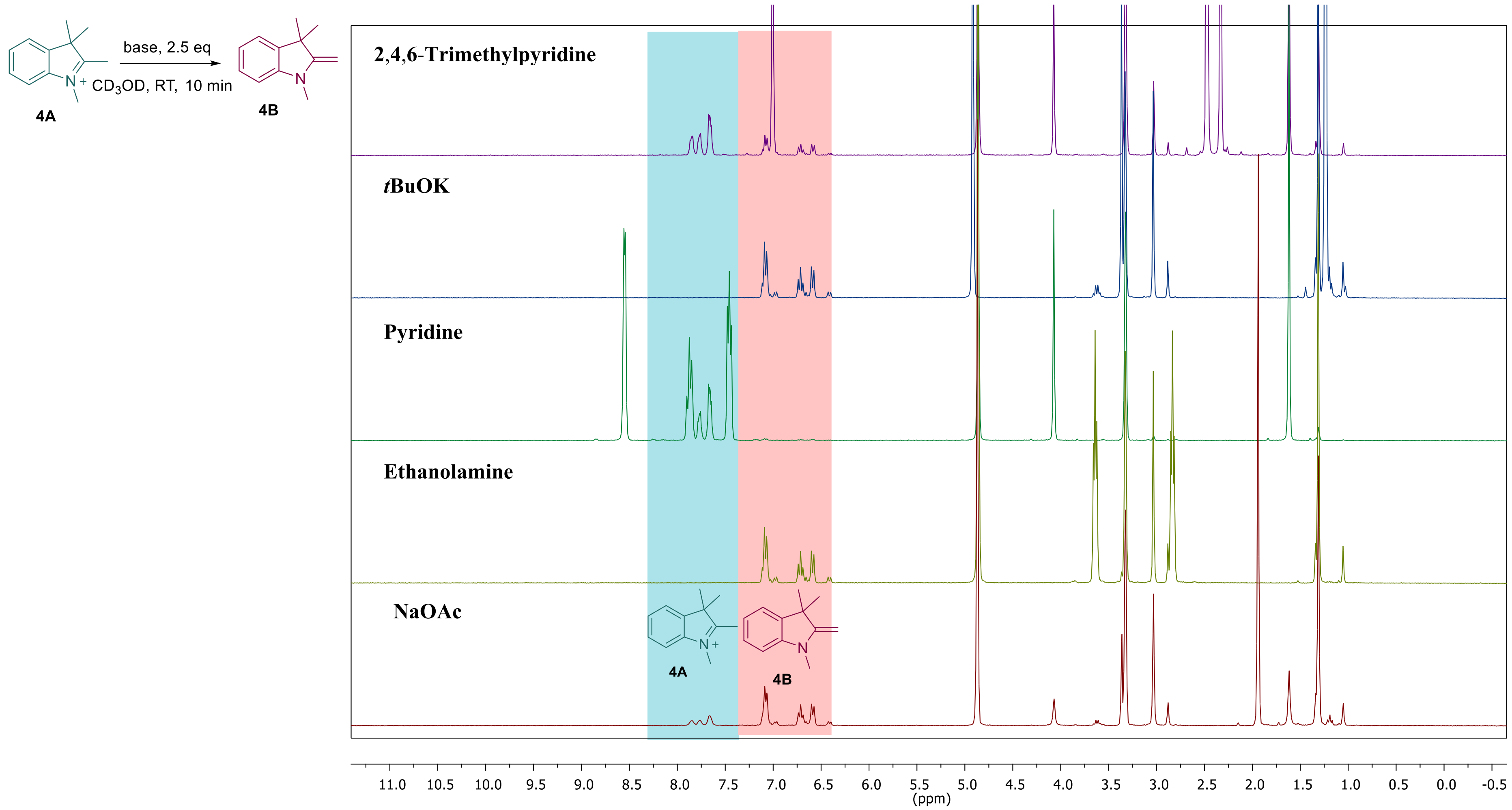

Figure S73. ^{1}H NMR (300 MHz): **4A** reacted with 2.5 eq of different bases in d_4–CD_3OD. The spectra indicate the conversion of **4A** to **4B**.

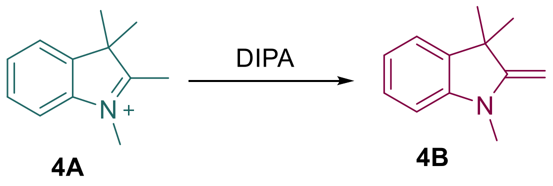

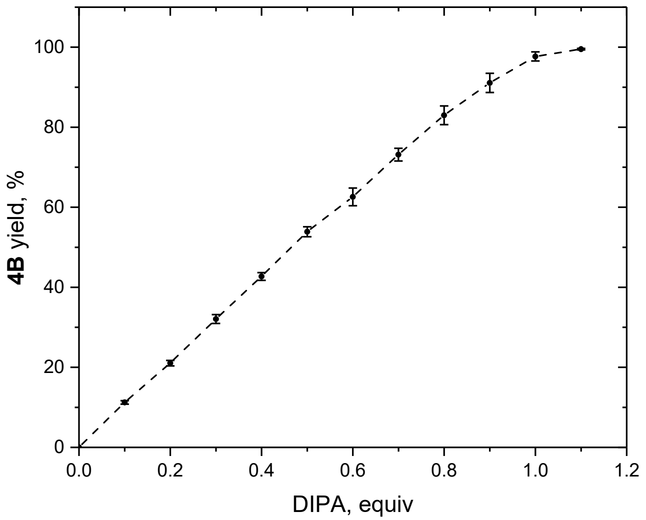

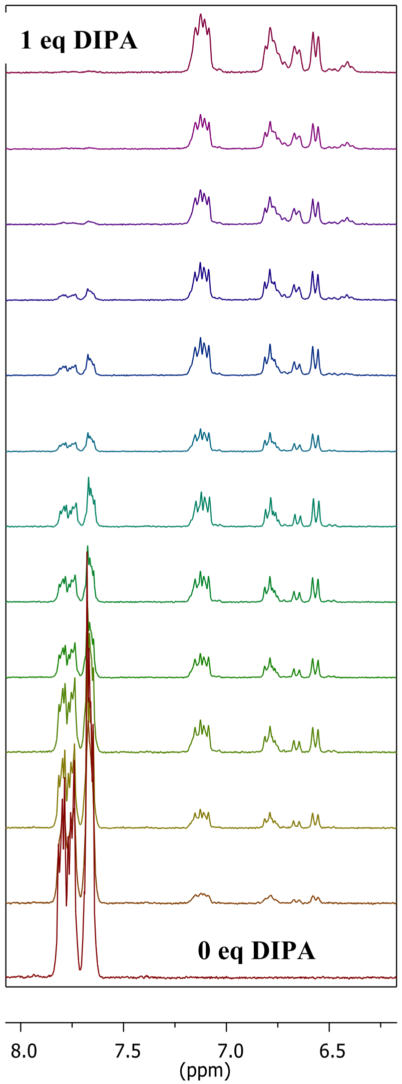

Figure S74. Titration of **4A** (15 mM) in d_4-CD_3OD:D_2O/d_6-DMSO (1:1:0.05) using diisopropylamine (DIPA).

Computational Analyses

We optimized all the structures at the DFT/PBE0[11] level with the triple-zeta def2-TZVP basis set[12] and the Grimme's D3 dispersion correction enhanced with the Becke–Johnson damping (D3BJ)[13]. The optimization was performed in the gas phase. All of the quantum chemical calculations were done with the ORCA 5.0.3 package.[14]

Electronic structure method: DLPNO-CCSD(T)

To describe the electronic structure of the studied molecules, we used the domain-based local pair natural orbital coupled cluster theory with single and double excitations and perturbative treatment of triple excitations (DLPNO-CCSD(T)) method as it is implemented in the ORCA 5.0.3 package[14]. We tested the convergence of both the basis set and the parameters of the localization and pairing of orbitals. For such benchmark, we compared the reaction energy for a reaction of **1c** with **4B** resulting into **A1**. The results are shown in Table S3. We observe that the energy is converged with the cc-pVTZ basis set and already with the "normal" setting of the DLPNO parameters.

Table S3. The reaction energies ΔE_{el} for a reaction of **1c** with **4B** resulting into **A1** calculated at the DLPNO-CCSD(T) level with three different basis sets and with three different sets of the DLPNO parameters: "normal" and "tight" correspond to the keywords "NormalPNO" and "TightPNO", respectively, used in ORCA 5.0.3[14], and "very tight" was performed by setting the variables $T_{cutPNO}=5\cdot10^{-8}$, $T_{cutPairs}=5\cdot10^{-6}$, and $T_{cutDO}=2\cdot10^{-3}$ (see ORCA 5.0.3 manual the literature,[14] pages 91–98). The geometries were optimized at the PBE0/def2-TZVP level with the D3BJ correction.

	basis set			PNO parameters (with cc-pVTZ)		
	cc-pVDZ	cc-pVTZ	cc-pVQZ	normal	tight	very tight
ΔE_{el}	−0.61	−0.56	−0.55	−0.56	−0.55	−0.55

Gibbs free energies and solvation at the DLPNO-CCSD(T) level

The Gibbs free energies in solution (ethanol) were calculated according to the equation

$$G_{\mathrm{EtOH}} = E_{\mathrm{el}}^{\mathrm{gas}} + \mathrm{TC}^{\mathrm{gas}} + \Delta G_{\mathrm{solv}}$$

where $E_{\mathrm{el}}^{\mathrm{gas}}$ is a DLPNO-CCSD(T)/cc-pVTZ gas-phase electronic energy, $\mathrm{TC}^{\mathrm{gas}}$ is a thermal correction calculated at the PBE0/def2-TZVP/D3BJ level in a gas phase, and ΔG_{solv} is a solvation Gibbs free energy calculated at the DLPNO-CCSD(T)/cc-pVTZ level as the difference between the PCM and the gas-phase electronic energies (PCM stands for Polarizable Continuum Model). The structures for such calculations were optimized at the PBE0/def2-TZVP/D3BJ level in the gas phase.

The gas phase thermochemistry data were evaluated with the usual approximations of rigid rotor/harmonic oscillator, with low frequencies treated as a hindered rotor.[15] The calculated thermochemical data were adjusted for a standard state of a 1 mol L^{-1} solution at 358 K (ΔG_{1M}). For a bimolecular reaction with one product molecule forming, the Gibbs free energy within the described standard state ΔG_{1M} is recalculated from the standard state of 1 atm (which is an output from the computational program) ΔG_{1atm} as follows

$$\Delta G_{1M} = \Delta G_{1atm} + k_B T \ln \frac{V_{1M}}{V_{1atm}} = \Delta G_{1atm} - 0.1 \text{ eV}$$

where k_B is Boltzmann constant, T is temperature, and V_{1M} and V_{1atm}, are volumes of an ideal gas with a concentration of 1 mol·L^{-1} and with a pressure of 1 atmosphere, respectively.

Activation barriers: DLPNO-CCSD(T) vs. DFT with the Δ*G*50% approach

We compared the Gibbs free energy profiles calculated with three different approaches: (I) DLPNO-CCSD(T) (described above), and (II) PBE0/def2-TZVP/D3BJ with both full inclusion of the translational entropy (ΔG_{full}) and (III) the $\Delta G_{50\%}$ approach. The PBE0 Gibbs free energies were calculated as described for the DLPNO-CCSD(T) case above, just the electronic energies were calculated at the PBE0/def2-TZVP/D3BJ level. The PBE0 Gibbs free energies within the $\Delta G_{50\%}$ approach were calculated according to the expression

$$\Delta G_{50\%} = \Delta H^{\text{gas}} - T \cdot \left(0.5 \cdot \Delta S_{\text{tr}}^{\text{gas}} + \Delta S_{\text{rot}}^{\text{gas}} + \Delta S_{\text{vib}}^{\text{gas}}\right) + \Delta G_{\text{solv}} = \Delta G_{\text{full}} + 0.5 \cdot T \Delta S_{\text{tr}}^{\text{gas}}$$

where T is temperature, ΔH^{gas} is enthalpy, $\Delta S_{\text{tr}}^{\text{gas}}$, $\Delta S_{\text{rot}}^{\text{gas}}$, and $\Delta S_{\text{vib}}^{\text{gas}}$ is translational, rotational, and vibrational entropy, respectively.

The results of such a comparison are shown in Figure S75. The highest activation barrier is given by the **TS-A2** transition state. This is, thus, the rate-limiting step.

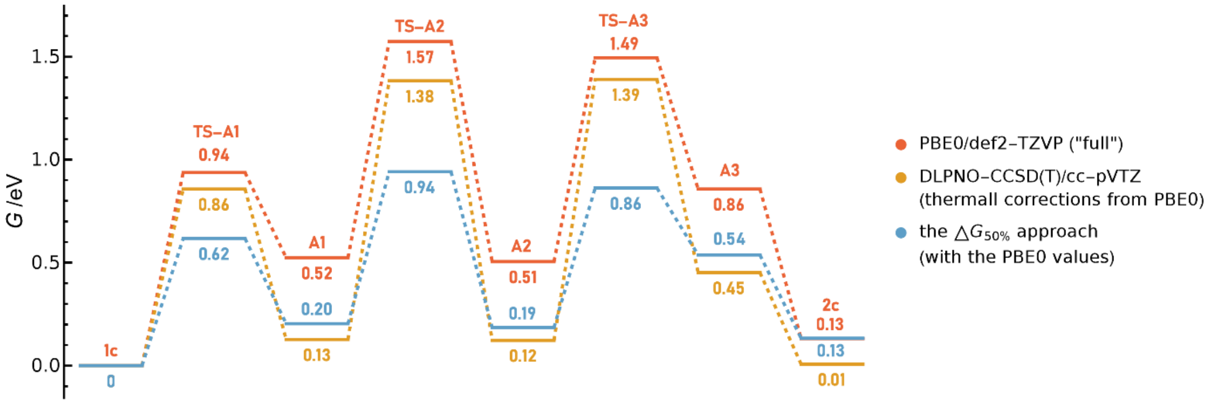

Figure S75. The comparison of Gibbs free energies (at 353 K) for a reaction of **1c** with **4B** at the C4' carbon calculated with three approaches: DLPNO-CCSD(T)/cc-pVTZ, full PBE0/def2-TZVP/D3BJ, and $\Delta G_{50\%}$ PBE0/def2-TZVP/D3BJ, all three in ethanol. The structures were optimized in the gas phase at the PBE0/def2-TZVP/D3BJ level. The correction to the quantum tunneling and the entropic correction for the 4 different transition states **TS-A1** (all described in the main text) are included.

Below, we also show the comparison between the full PBE0 and DLPNO-CCSD(T) Gibbs free energies for all the three paths following the attack of **4B** to **1c** on the C2', C4', and the C6' carbon atom. The data are shown in Figure S76.

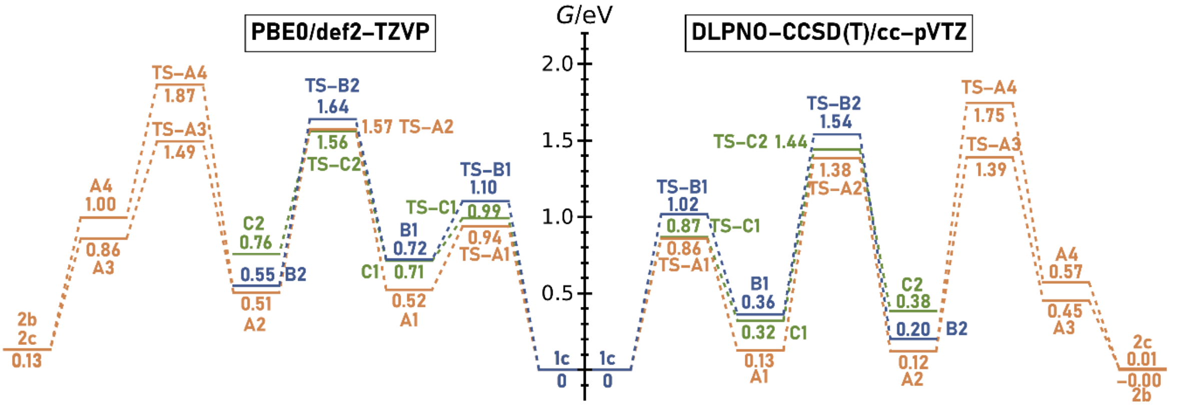

Figure S76. The comparison of Gibbs free energies (at 353 K) for a reaction of **1c** with **4B** calculated with the DLPNO-CCSD(T)/cc-pVTZ method and the full PBE0/def2-TZVP/D3BJ, both in ethanol. The structures were optimized in the gas phase at the PBE0/def2-TZVP/D3BJ level. The correction to the quantum tunneling and the entropic correction for the 4 different transition states **TS-A1** (all described in the main text) are included.

Truncation of 1c with a secondary amine, and of 1g

The activation Gibbs free energy and the overall Gibbs free energy change for the reaction of **4B** with **1g** is shown in Figure S77.

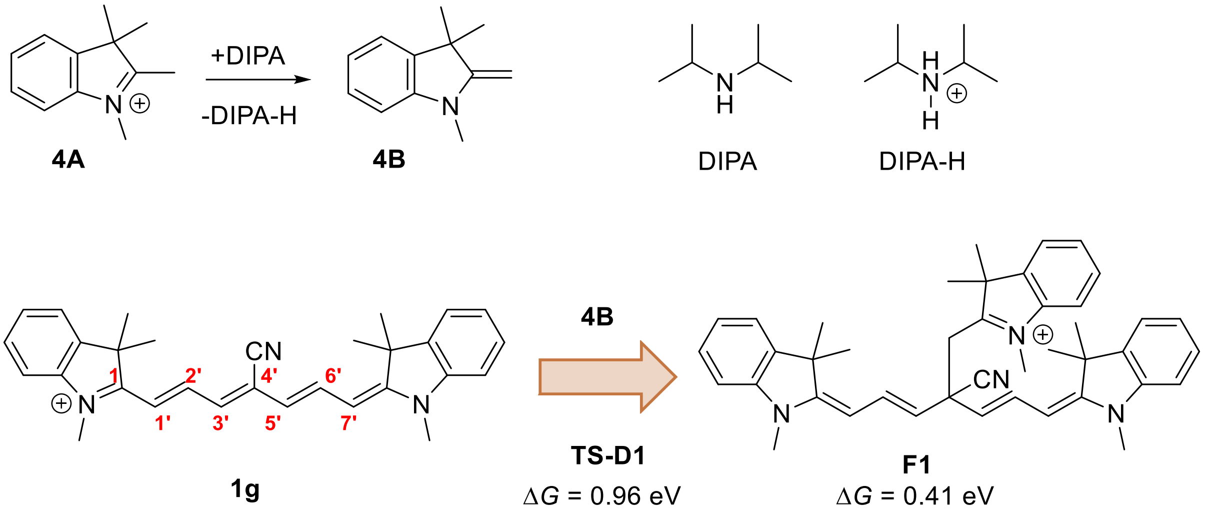

Figure S77. Gibbs free energies of the addition of **4B** to **1g** in ethanol caclulated at the DLPNO-CCSD(T)/cc-pVTZ level with the standard state of 1M at 358 K. The structures were optimized in the gas phase at the PBE0/def2-TZVP/D3BJ level.

The Gibbs free energy diagram for a path of truncation of **1c** with DIPA leading to **2c** and **3a** is shown in Figure S78.

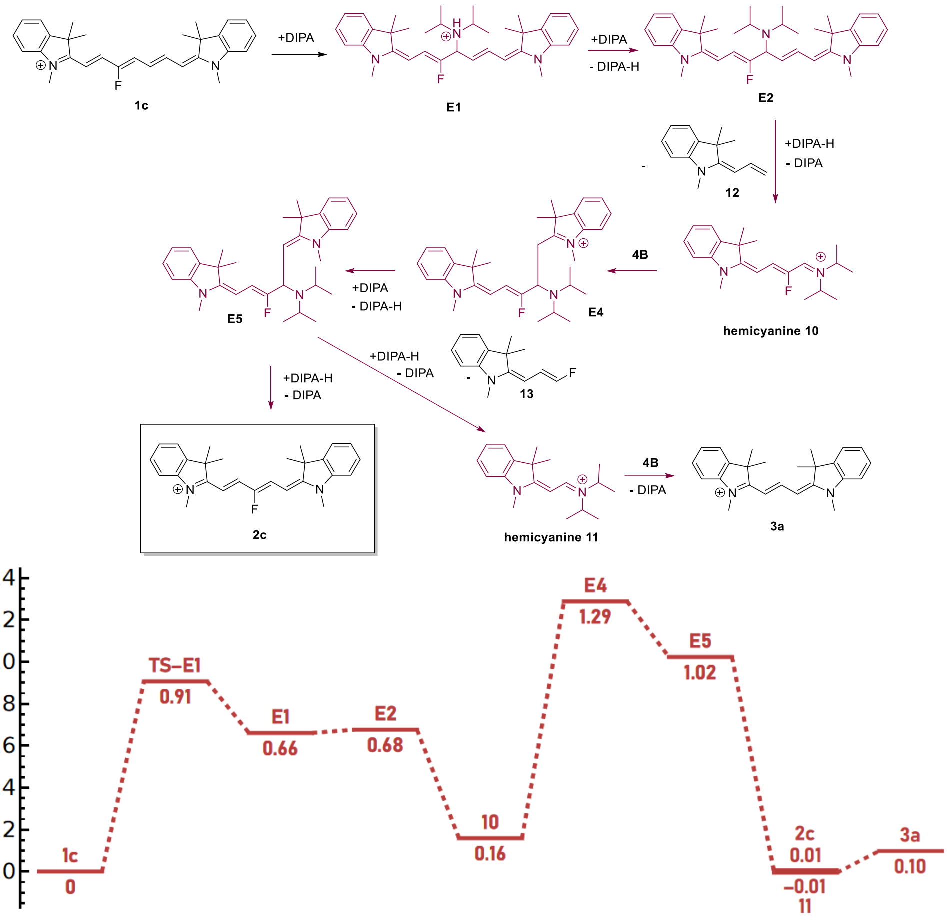

Figure S78. The Gibbs free energies (at 353 K) for a reaction of **1c** with DIPA calculated with the DLPNO-CCSD(T)/cc-pVTZ method in ethanol. The structures were optimized in the gas phase at the PBE0/def2-TZVP/D3BJ level.

Cartesian coordinates of optimized structures

Structures were optimized at the PBE0/def2-TZVP/D3BJ level in a gas phase in the ground singlet state. The character of the states were confirmed by frequency analysis.

1c

64

C	-8.98939827861660	-1.71638308074271	0.03388579285005
C	-7.63449308556901	-2.03662384865232	0.04301876608598
C	-6.71160386071530	-1.01370319461817	0.02373893361943
C	-7.14066403817070	0.30645848616672	-0.00253172297510
C	-8.47998213101681	0.64918325175496	-0.01455420279955
C	-9.40141969675385	-0.39102564997464	0.00459142237078
C	-5.20366774272432	-1.04851835633808	0.02378598315853
C	-4.87007335612751	0.43684327461711	-0.00540841544481
N	-6.01449669094114	1.14267357190129	-0.01356783154274
C	-3.63162304614087	1.05593011736939	-0.02350024080910
C	-2.40145052304453	0.41328687123075	-0.01203717891497
C	-1.18718100567360	1.07172112678355	-0.02758747549406
F	-1.21180465180079	2.41514941008626	-0.05220291076641
C	-6.07911981248012	2.58316434848487	-0.03531973304636
C	-4.69122989497728	-1.76630934976995	-1.23005014427122
C	-4.68801019792071	-1.71546620987420	1.30397111139086

C	0.05535954584410	0.46033031730602	-0.01943831748471
C	1.26790140847449	1.12804612788090	-0.03489016017782
C	2.48508325893405	0.46540349687816	-0.02543512785198
C	3.71414066525972	1.11119454920205	-0.03920145825373
C	4.95817916804087	0.50168881678962	-0.02827547116600
C	5.30202892550466	-0.98169512335176	0.00575252782168
N	6.09790496661909	1.21449762154149	-0.04616008533058
H	2.45522595556941	-0.61881532451046	-0.00592239153671
H	1.26081949415620	2.21295120546210	-0.05476170592199
H	0.04792274731868	-0.62520329263596	0.00060864213297
H	-2.33665542652393	-0.66621960985935	0.01057733464589
H	-3.61070963407018	2.13812833494111	-0.05073148870999
H	-7.11553442815238	2.89621094829683	0.04574057834800
H	-5.52284300763422	3.00050119952526	0.80596128167066
H	-5.66472417922877	2.97411515706609	-0.96731621335417
H	-5.05017787162693	-2.79678876829606	-1.22780452592745
H	-5.05828176621048	-1.28190752339155	-2.13588087057892
H	-3.60210168645550	-1.78676039284999	-1.26965984077271
H	-5.05142275707798	-1.19387135432218	2.19044175976937
H	-5.04801348751264	-2.74480300168802	1.34515330731167
H	-3.59874269018975	-1.73543557858929	1.34051526312229
H	-8.81780217544424	1.67703603843659	-0.04000349588530
H	-10.45914885936574	-0.15868252604234	-0.00409305320638
H	-9.72865429666557	-2.50734160411274	0.04857495317397
H	-7.31950234117005	-3.07389803085857	0.06447953332169
C	6.80967003265873	-0.93651474387005	0.00825245877843
C	7.23011820905584	0.38605168380766	-0.02289532742677
C	6.15440905173270	2.65503458503145	-0.08266609027410
H	5.70046824448318	3.08086419521760	0.81477844192516
H	7.19172885207279	2.97138374746582	-0.13181017072304
H	5.63233755014825	3.03466863630952	-0.96310594388807
C	8.56706601219147	0.73782689771052	-0.02779748523767
C	9.49530346825082	-0.29614613284500	-0.00145365977239
C	9.09194731156243	-1.62412882774799	0.02919269120303
C	7.73923750698147	-1.95330129342083	0.03445564188455
H	7.43097658895532	-2.99251556013255	0.05877168215848
H	9.83639928374583	-2.41006567111745	0.04951280425530
H	10.55146922648351	-0.05668068549592	-0.00452428747997
H	8.89862434632007	1.76780635726434	-0.04968878907893
C	4.79019775840611	-1.64993176062073	1.28668992066366
H	5.15917043559341	-2.67596705345076	1.33067540749224
H	5.14811578509421	-1.12323146294652	2.17240210917191
H	3.70120947375325	-1.67884076061061	1.32127729576763
C	4.79689329756560	-1.70592757002336	-1.24757916774125
H	3.70804862785470	-1.73474733675565	-1.28774493682760
H	5.16133364412093	-1.22033184485840	-2.15386485867915
H	5.16440001759706	-2.73337784710960	-1.24277701515411
H	3.69078375965259	2.19538199695575	-0.05854084958838

TS-A1

92

C	-4.21928229048246	4.07359187487670	1.10314186172698
C	-3.51882292334594	3.16832132459579	1.89387978114485
C	-2.18866952821703	2.96488517936031	1.58306834827901
C	-1.54863433631386	3.62565240761641	0.54340822313782
C	-2.25430176741979	4.51652120386242	-0.23578305654822
C	-3.60087150003354	4.73418650083469	0.05052386434370
N	-1.27327607165721	2.10601033473019	2.21112823225421
C	-0.06634145888132	2.17086213067840	1.63437182199500
C	-0.10306969382970	3.20063609740364	0.51662202317380
C	1.00813596118047	1.33778530424957	1.92442340975862
C	0.73882571846665	-0.39064044580517	0.90285264030166
C	-0.51659550810478	-0.88918021603539	1.37865099109838
C	-1.70659426129301	-0.73523254709178	0.75790300331995
C	-2.95968098336173	-1.18457387361930	1.23703006923267
C	-4.13966087294517	-1.09347805685346	0.55137455142141
C	-4.39323506902476	-0.56990655868058	-0.86202923338823
C	-5.88307227453181	-0.78019113050765	-0.99463491732884
C	-6.37611416929803	-1.34862623064046	0.17371149417108
N	-5.31943655503139	-1.52796584686401	1.06989713061666
C	-7.71657220378256	-1.65280261261865	0.32910953532329
C	-8.56575768526727	-1.36798309141852	-0.73536313608311
C	-8.08747537333340	-0.80197311063755	-1.90795171395372
C	-6.73339356113873	-0.50341450850192	-2.04207754836206
C	-4.06179398652025	0.91665465464281	-1.01310631664122
C	-3.64828767124151	-1.41391937962733	-1.90158358164308
C	-5.44591882653136	-2.10316105102105	2.37918125378293
C	0.81646536188520	4.37297897553570	0.88794460698819
C	0.30708196395122	2.62000844190141	-0.83489178663341
C	-1.64360631032928	1.35505370250433	3.38441947623350
C	1.95670533460488	-1.10621599731976	1.15376302808706
C	3.01495073492553	-1.01968961138662	0.30441352069435
C	4.23452485947663	-1.71583923023394	0.43610038592825
C	5.28252868678493	-1.65786956978744	-0.44304496670078
N	6.41819769993331	-2.38416360428655	-0.27236059575857
C	7.35120438966045	-2.13137016849158	-1.28097036089463
C	6.81240661328638	-1.21160720625956	-2.17246267446345
C	5.42418626514718	-0.82774374957396	-1.71762046278945
C	8.61878116453271	-2.65847999355179	-1.45095864206061
C	9.34498331520865	-2.23625107838582	-2.55942098920498
C	8.81959352449908	-1.31997338346775	-3.45877272953658
C	7.54119689432604	-0.80086438300841	-3.26676938107535
C	6.62801279916357	-3.29234551564174	0.82135850049316
C	4.39619864829939	-1.23516375355555	-2.77806526186757
C	5.36409512573104	0.67461345206992	-1.42644567856598
F	-0.47241239433133	-1.50908469232538	2.57963375404398
H	2.89078782955203	-0.37301754170386	-0.55900480515673
H	2.01400808908683	-1.72741694659299	2.04155178285954
H	-1.65433218791564	-0.22570202969125	-0.19454044831030

H	-2.96512349343763	-1.66955162144316	2.20589041197859
H	-6.48430236224601	-2.36381510699546	2.56333532676586
H	-4.83965270133020	-3.00898203521717	2.46570673407178
H	-5.12423815926802	-1.39316970536020	3.14682822709009
H	-4.37412544444201	1.25901767765387	-2.00151214658767
H	-4.58566100526932	1.51378247644846	-0.26709891065710
H	-2.99465239903800	1.11608188833334	-0.91361564141884
H	-3.89964685330962	-2.47031175483902	-1.79760392321326
H	-3.93549694885228	-1.09410753878876	-2.90513915136192
H	-2.56659132702837	-1.31144980632570	-1.80653763923014
H	-8.10841924918123	-2.09783321394002	1.23475376161624
H	-9.62055414246748	-1.59647571711468	-0.64242824920171
H	-8.76961937214977	-0.59368206027713	-2.72257073876015
H	-6.36056346526525	-0.06287771533246	-2.96031968022381
H	5.81233490192741	-4.01716906053195	0.87525192293651
H	7.55747196069759	-3.83367409620824	0.66827482261779
H	6.68691557584214	-2.75543321948781	1.77241766209283
H	7.13326554004020	-0.08497114525918	-3.97209003445975
H	9.40634491128796	-1.00676893328914	-4.31315737535451
H	10.34056131576283	-2.63248284107696	-2.71836250604363
H	9.04726786607964	-3.37087524983228	-0.75786424406833
H	4.60486127204874	-0.70614329723707	-3.70998757255812
H	4.44902789496730	-2.30619351930669	-2.97807543570803
H	3.37749198180331	-0.99611499796789	-2.47134374874564
H	4.37514273309950	0.98123783133377	-1.08387622475502
H	6.09165714286773	0.95108059242483	-0.66207544477942
H	5.59776489635434	1.23334925384806	-2.33480418871641
H	4.33448869518327	-2.36137700570632	1.30184608465325
H	1.07583764283764	0.88768217770392	2.90688884469883
H	1.96278371184517	1.62954870878887	1.50716953032442
H	-1.95341065271625	2.04233070209846	4.17492055052743
H	-2.47052485816530	0.68371242496466	3.14191988799448
H	-0.80606182512712	0.76008655447662	3.73256194908057
H	0.73081907981402	5.15798254326378	0.13448807216808
H	0.54268005344705	4.79617487979106	1.85525252554489
H	1.85799037858100	4.04873379009074	0.93188080726857
H	0.25438015656579	3.39874273161087	-1.59749231585754
H	1.33450882061932	2.25098348612516	-0.80718508867867
H	-0.35443654973485	1.80845159529478	-1.14240689903536
H	-1.77836281055183	5.04628327232247	-1.05334994350131
H	-4.17032279013061	5.43182851018803	-0.55080129633882
H	-5.26510964111697	4.26189741695230	1.31284203747639
H	-4.00430533093936	2.64891600596229	2.71011715298783
H	0.65983133455614	0.03238365218529	-0.09204091608220

A1

92

C	6.80381272576189	-1.85730746723939	-3.74213581948165
C	6.28998534346584	-1.86927189128588	-2.46484728327618
C	6.96237170135463	-2.53432096615220	-1.44502404298143

C	8.15565119428546	-3.19668874700661	-1.67520911020288
C	8.66483004979893	-3.17726204787844	-2.96988082816782
C	8.00363606647351	-2.51927950061064	-3.99584825186096
C	5.02626924899796	-1.25085595333531	-1.91272223669905
C	5.09874318210686	-1.66866613183524	-0.44340788431377
N	6.24007054131653	-2.40276123479379	-0.26168840948437
C	4.22717136859875	-1.40862847657468	0.56607395563520
C	3.00715018147411	-0.67696603428449	0.45353512238788
C	2.11717752913307	-0.49365064179895	1.44394456627532
C	0.83686748669449	0.23968221760763	1.24388181545793
C	-0.36711254384128	-0.58195514176043	1.51032109093635
F	-0.34829268267466	-1.17139488612927	2.73240576769079
C	6.62215111491936	-2.96771704577635	0.99942362832585
C	3.80357399169519	-1.84842770789951	-2.61436263455060
C	5.07500893845984	0.27104515672320	-2.07344936577448
C	-1.44133008412578	-0.71247328302971	0.72616316540552
C	-2.62614548802286	-1.44974201412588	1.04168479106059
C	-3.74549950334297	-1.50432428458488	0.27286254420294
C	-4.03787914832379	-0.79668017103471	-1.05128576079113
C	-5.45640851888826	-1.24178482894826	-1.32311937357772
C	-5.87452390283984	-2.09746550948520	-0.30953438048044
N	-4.84084352712795	-2.25195265399428	0.61179789386116
C	-7.13982467427596	-2.65781858388866	-0.30979952936645
C	-7.98548094234805	-2.33903344388709	-1.36805650216999
C	-7.57961686245176	-1.48964730211152	-2.38617892105634
C	-6.30165177108093	-0.93429832592451	-2.36551800246603
C	-3.98348892857761	0.72646224533653	-0.91445640871229
C	-3.11372601321411	-1.28957407067382	-2.16933856836147
C	-4.90342763006414	-3.06593180640319	1.78972248890477
C	0.82175102313648	1.54323267349603	2.15825397242712
C	-0.29203210139659	2.42592284800574	1.81758082839193
N	-1.51883846801556	2.29363667028121	2.24898767554539
C	-2.39007669551516	3.22533698136512	1.62764303083936
C	-1.64444730647583	4.00920447248800	0.76302095435371
C	-0.20732038032412	3.57254420097012	0.83791540316180
C	-2.27161249753896	4.99485791970223	0.02954007138762
C	-3.64454789529564	5.16174816297327	0.18571057245262
C	-4.37173683101648	4.36135284724419	1.05900892010717
C	-3.75029203521858	3.36781237616176	1.80563358895025
C	-2.02053098882239	1.37251683437510	3.24879193772771
C	0.67901085866689	4.67538500769161	1.44026974548428
C	0.34382178139899	3.13458521626876	-0.52144787211988
H	2.76421518276837	-0.24903809460929	-0.51481933328004
H	2.29761138835710	-0.91139722852530	2.43058400933860
H	-1.37415865498254	-0.19595321051691	-0.22234826355513
H	-2.60416621491818	-2.02341698637160	1.96056320413889
H	-5.81835814990807	-3.65254011690608	1.78017753494225
H	-4.05415748143607	-3.75354162509894	1.82407490717299
H	-4.89125150050296	-2.45299177055028	2.69760583894733
H	-4.30608936631570	1.19199125477883	-1.84803904932610

H	-4.65064621434726	1.06845557817032	-0.12191266831739
H	-2.97663437135513	1.08269969902299	-0.69119676095168
H	-3.16755585863671	-2.37486793298517	-2.26344513381079
H	-3.42246922103570	-0.85194750317741	-3.12095512622234
H	-2.07279780378324	-1.01705059212470	-1.99085994423756
H	-7.47668481377142	-3.32199120099231	0.47597175617888
H	-8.98051255272925	-2.76689271258789	-1.39249514800667
H	-8.25649366234606	-1.25965178889538	-3.19931651735123
H	-5.98227696151293	-0.27187390654626	-3.16311082388056
H	5.85218388399963	-3.65236925854910	1.36733846419824
H	7.54869565265317	-3.52343540706668	0.88295375119292
H	6.77876769042026	-2.18502005515977	1.74790317429037
H	6.28592087984700	-1.34343739845602	-4.54497814481048
H	8.42068281741579	-2.52037679894122	-4.99503928150640
H	9.59720906269603	-3.68937840380872	-3.17543820132159
H	8.68783398969422	-3.71693180159310	-0.88906192185868
H	3.84854967421987	-1.62652393801684	-3.68268145206011
H	3.78152481686407	-2.93248367971825	-2.49464008505285
H	2.87025692237685	-1.44471329327791	-2.22016870057954
H	4.18204593371967	0.75066486918066	-1.66961031611123
H	5.94565727469271	0.68556297679358	-1.56319192319040
H	5.14932897464811	0.52744928110901	-3.13243510943735
H	4.45383590638658	-1.80966188860065	1.54827400202063
H	0.79061549398090	1.21581741156081	3.19874572570122
H	1.76405370974045	2.06523632163684	1.99671056645244
H	-2.55039370754565	1.94590022928932	4.01011242064810
H	-2.70040630557535	0.66248369429266	2.77293787268352
H	-1.20260545192181	0.82296088434720	3.70096282832328
H	0.67110887036287	5.53909804231446	0.77408112328947
H	0.31208510265447	4.99562029848904	2.41657227688748
H	1.71035169991785	4.33434780818068	1.54309259722458
H	0.35556101362504	3.99493726436489	-1.19199011489237
H	1.36744730770903	2.76682204081773	-0.43194523138693
H	-0.27756699453710	2.36144661288740	-0.97544974006966
H	-1.71821399432896	5.62712972924763	-0.65504802315366
H	-4.15643364848545	5.92794820831234	-0.38327670352001
H	-5.43927579138271	4.51144674742955	1.15977575378973
H	-4.31621998710407	2.73824608347011	2.47994430973843
H	0.77790955279275	0.56188087914209	0.20329225565308

TS-A2

114

C	-6.84145935160925	0.98236681990472	0.75571756898253
C	-5.99522556788104	-0.07942497880458	0.52866396007999
C	-6.39579974619924	-1.37687737157772	0.83062310571127
C	-7.64785532111348	-1.63940268497963	1.36095676494660
C	-8.49418006566323	-0.55821877176505	1.58674285203646
C	-8.10483670562415	0.73951508049100	1.29170498341090
N	-5.36904589792780	-2.25824719406248	0.51385773473584
C	-4.27766353202493	-1.60399762670713	-0.01005146023244

C	-4.58967929152188	-0.10402377955802	-0.02615470140303
C	-3.15918500348296	-2.26491872681058	-0.39829220237437
C	-1.96813837581274	-1.69063297325796	-0.95608601254848
C	-0.87067400743870	-2.37685194461835	-1.28824039349560
F	-0.85819939348054	-3.71597966362370	-1.09024802712525
C	-3.64942930134868	0.67735960807430	0.89311845020742
C	-4.56871083116422	0.49232726931872	-1.43380504686490
C	-5.41479140801606	-3.67718764026955	0.70575769946911
C	0.40561024588932	-1.92628549379667	-1.93844137203295
C	0.37058668924102	-0.49113809559738	-2.42251634652225
C	0.24876587175042	0.67988102278517	-1.64222728306122
N	0.00602346531570	0.85094262640333	-0.33998650628050
C	-0.18912953124493	2.21625556193475	-0.02523713186585
C	-0.00034454096828	2.97862466737705	-1.16309500104129
C	0.37576445388474	2.06250635361483	-2.29391468319453
C	-0.12753281303436	4.35022577846448	-1.10192856368466
C	-0.45546298723296	4.93304829290515	0.11943125645021
C	-0.64196822014005	4.15096722971853	1.25126372232364
C	-0.50906911411213	2.76726519381001	1.19939424136982
C	0.08874847767113	-0.10582836375755	0.74841574033626
C	1.83905739781289	2.30311979632893	-2.69186917162070
C	-0.53208171187967	2.24165390413334	-3.50567643007074
C	1.62772462264118	-2.34128861679710	-1.15830230497235
C	2.67235513074689	-1.55203964831707	-0.87033015595860
C	3.82474194948639	-1.96034249507447	-0.12190127533778
C	4.83587782956704	-1.15867062914297	0.29254731557139
C	5.01746697530242	0.34688034144568	0.09095467161547
C	6.30337969622177	0.60147954063668	0.84346630535140
C	6.77910938009631	-0.59926947577117	1.36170453979699
N	5.90670035657226	-1.62494586472587	1.01535208480181
C	7.95371049605161	-0.65512980176362	2.09263312634519
C	8.64832176579940	0.53381941478875	2.29225756086613
C	8.18497438167989	1.73615125027919	1.78016615931322
C	7.00000436390880	1.77127755437267	1.04705451710235
C	5.20333187094421	0.69664202836327	-1.38780958159130
C	3.87418866649895	1.14973137022356	0.71563054597985
C	6.06767446926732	-3.00054663427897	1.38330741281575
N	-1.26779427661258	-0.88700013575426	-4.59590652630383
C	-0.57514087239976	-1.79157803687147	-5.54561383761503
C	-1.22170782487543	-1.80680319031298	-6.92144624963084
C	-2.67596201387829	-1.19334273938151	-4.20896707185620
C	-2.94965665517866	-2.67881309105535	-4.10447755514487
C	-3.68467981400008	-0.46732264408925	-5.08265061630617
C	0.88693078873415	-1.39175983772087	-5.66039830982252
H	2.63680376107112	-0.52273820714209	-1.21310417003010
H	1.63613156197144	-3.37938027864490	-0.83804708380929
H	-1.93177351751474	-0.62596796668636	-1.13517801236257
H	-3.14454759384904	-3.34073222386913	-0.27507281823019
H	-6.36455271818767	-3.95375001527784	1.15577186288958
H	-5.31510630735395	-4.20641577325033	-0.24746421206651

H	-4.60868500616216	-4.00647894843765	1.36867037810226
H	-3.98685891534615	1.71235386115757	0.98002791447991
H	-3.62898535106996	0.24094739176744	1.89282838405014
H	-2.63521293806661	0.69175846587081	0.49479369472923
H	-5.17785320403882	-0.09898495943275	-2.11882812322776
H	-4.97293568562486	1.50638177656599	-1.41029052709229
H	-3.55293801030152	0.55131493808815	-1.82864443264882
H	-7.97397486638654	-2.64405881197773	1.59792101391296
H	-9.47831628038969	-0.74019690491479	2.00180513893836
H	-8.78239950789309	1.56318247861352	1.47779450979655
H	-6.53543641178641	1.99733528084910	0.52387228739244
H	6.04689115317355	-3.64127515442825	0.49705372108142
H	7.02499109864902	-3.13500928342804	1.88002631667318
H	5.27365174786751	-3.32413516382686	2.06432763139686
H	6.63809984636325	2.71152199147334	0.64428565979213
H	8.74390785991034	2.64760490802266	1.95059308777240
H	9.56972128406676	0.51415966179516	2.86201511419277
H	8.33280714573739	-1.58220359827943	2.50334118130144
H	5.42640294447359	1.76111238695474	-1.49175659183447
H	6.03579191275347	0.13451653954524	-1.81287172606308
H	4.31088801363663	0.47287704777432	-1.97358977143559
H	2.92709714247901	0.96405737791715	0.20748619362310
H	3.75229015974120	0.89113056931002	1.76860971522424
H	4.09214708300151	2.21839839415667	0.65374715276651
H	3.86461413279810	-3.00911985666708	0.15291314458716
H	1.14991986913892	-0.30511508430593	-3.16521356150456
H	-0.54926407383540	-0.61618298161469	-3.44888358509088
H	0.70784772794596	0.34048575248397	1.52830227985673
H	-0.89853355231858	-0.32601360584910	1.15259868182168
H	0.55992494713766	-1.02011141295050	0.41033617205466
H	1.94257371318653	3.31175168860589	-3.09543166715951
H	2.50274097831718	2.21462355680438	-1.83162957617899
H	2.15997121095080	1.59379093776113	-3.45774037945212
H	-0.54277677858008	3.29149580324832	-3.80205347958942
H	-0.15561621127289	1.66932453837046	-4.35484953636895
H	-1.55930229928579	1.94400581062647	-3.28590466644684
H	0.02387176496107	4.96927141730314	-1.97917327480351
H	-0.56562405973211	6.00809874449639	0.18829193089987
H	-0.89612659988729	4.62290064213777	2.19231875669357
H	-0.66057106722584	2.16076655828600	2.08271708468236
H	0.44097321802200	-2.52578224099693	-2.86694717014892
H	-2.76730658539426	-0.78805684911789	-3.19953724199777
H	-3.92677191105554	-2.81745769903790	-3.64030391408109
H	-2.97156396685506	-3.17018509695457	-5.07837236626971
H	-2.21981833754014	-3.17984908428166	-3.46816620957261
H	-4.68504966953782	-0.59271684683360	-4.66497552447346
H	-3.48034942249962	0.60693649412132	-5.11642808156227
H	-3.70265032729170	-0.84371374510389	-6.10541775687866
H	-0.62917522074368	-2.79191935543156	-5.11197429534741
H	-0.65223527670185	-2.45950329474240	-7.58533213259410

H	-2.24612746359323	-2.17377694446732	-6.90015305683182
H	-1.22295192613515	-0.80571830024644	-7.36318095841079
H	1.36991368925092	-1.99815393971431	-6.42715161453858
H	0.99044179529094	-0.34490489939430	-5.96332698695888
H	1.43760674019393	-1.54466038816503	-4.73274549230435
H	-1.27137760785013	0.03897234623342	-5.01719251300579

A2

91

C	7.01516095402871	1.37160928208763	0.58410868115776
C	6.20833168323425	0.26080168066711	0.49762411594256
C	6.60958198054516	-0.94677869325821	1.06764346137414
C	7.82034670977801	-1.05955226017449	1.73361560368389
C	8.62676019328434	0.07224901826597	1.81254308747044
C	8.23813474359692	1.27751737898798	1.24902258353679
C	4.85912525688772	0.08388175452109	-0.16141313423818
C	4.56123225895989	-1.38586317163195	0.14208918010558
N	5.63623701447811	-1.90468578630578	0.84281738180972
C	3.47207449220317	-2.11783846711753	-0.17669304765293
C	2.32575056503616	-1.64724970280543	-0.90588466623450
C	1.22598734595539	-2.36556495861743	-1.14868479150678
C	0.03005322532286	-1.86564478976775	-1.92842723447402
C	-1.25628305979661	-2.38644147625854	-1.35453534261303
F	-1.24910498765363	-3.73415539929664	-1.22101011492952
C	5.69757480359496	-3.26361000830594	1.28249841452404
C	4.97792497667327	0.34046786572602	-1.66668193702150
C	3.82984955836082	1.02154767715821	0.47419760648278
C	0.00666750828281	-0.38578876720327	-2.18866792376630
C	0.06318052356008	0.67681587221134	-1.36981695504861
N	0.12195952142357	0.77298496935688	0.01524394047484
C	0.25448605352777	2.09369659172558	0.41693126781251
C	0.23454782420974	2.94059700035722	-0.68709635787345
C	0.08712764467215	2.10883091761366	-1.93428774538405
C	0.33985206868816	4.30219639124113	-0.52012091240483
C	0.47353385847176	4.82138188528408	0.76868543027271
C	0.50026514224108	3.96764883343234	1.86023019527290
C	0.39224622917189	2.58798837599054	1.70467814610614
C	0.05558157591369	-0.27464361924441	0.99961923032471
C	1.27873645185403	2.31127336155207	-2.86872434334793
C	-1.22065384116983	2.43455992594286	-2.65580706363212
C	-2.34053029156236	-1.69578504487576	-1.00416595046160
C	-3.54051291882043	-2.23977353303539	-0.43428052801807
C	-4.60242251901005	-1.51317210740342	-0.02619491608846
C	-4.81647403454823	0.00056777886709	-0.08793634060107
C	-6.18901170271959	0.13613034093052	0.53229863661209
C	-6.66942569601661	-1.12631681704248	0.87905091064084
N	-5.72699868122344	-2.08258929388612	0.54512786713722
C	-7.91568041585261	-1.28359895688701	1.46586552756894
C	-8.67244519985217	-0.13966029018507	1.70277786326836
C	-8.20376539833567	1.12005728280791	1.36419955696167

C	-6.94836640795432	1.25792492719360	0.77112082036289
C	-3.78518283475039	0.76857483589741	0.74363636526201
C	-4.84377062195565	0.50681828531184	-1.53287893117937
C	-5.86143390015408	-3.48974189506659	0.75973928037840
H	2.33933230720851	-0.62939503709741	-1.27698703185936
H	1.16426277854266	-3.39451398620631	-0.80385117659804
H	-2.25362188544636	-0.63146856201238	-1.16187102479034
H	-3.57270879003944	-3.31591718326784	-0.31136158160906
H	-6.81196313414044	-3.69734668010156	1.24532357528527
H	-5.82721152367501	-4.03701843764210	-0.18839474888827
H	-5.05535561507452	-3.86511039288641	1.39909790163575
H	-4.04868687803200	1.82829859588333	0.77355170715022
H	-3.76274847118608	0.39613382409341	1.76906269569283
H	-2.78147666104888	0.68369815381870	0.32733793100930
H	-5.59160851771684	-0.03369924174260	-2.11527599897444
H	-5.10271287024580	1.56840195473217	-1.54855229692776
H	-3.87745368890116	0.38427697212664	-2.02311453986384
H	-8.30280679668441	-2.25783294158781	1.73706214635772
H	-9.64899699106038	-0.24249459232010	2.16184492486210
H	-8.81099953674219	1.99523463824500	1.55951408817549
H	-6.57817511235864	2.24207490746121	0.50302061440206
H	5.57890414101106	-3.94953071156801	0.43761548584497
H	6.66234170857944	-3.45451298000529	1.74653432408987
H	4.91053625964795	-3.48196377916659	2.01295518547932
H	6.70678691510639	2.31318624814176	0.14169335969691
H	8.88253212289098	2.14453225608777	1.32547221459093
H	9.57737196999222	0.00404003616869	2.32893812659339
H	8.14319502105719	-1.98997111896705	2.18376693122878
H	5.30463990871926	1.36866960489337	-1.83981968101515
H	5.71312318891540	-0.32951940403618	-2.11529815129447
H	4.02623272811715	0.19385277794415	-2.17821326921109
H	2.84162607286279	0.89248327906201	0.03409868982928
H	3.75175518003995	0.84016928059552	1.54740272148743
H	4.13100072498649	2.06162499374644	0.32925255043908
H	3.44086001967428	-3.15332139663452	0.14819822502875
H	-0.05525439989343	-0.11967945426519	-3.23727072038574
H	-0.61456035379620	0.03757487092588	1.80538609811823
H	-0.34607820374577	-1.17921855511228	0.55953520515414
H	1.04108068220754	-0.49396092222812	1.42113360109910
H	1.32196240592924	3.34961553769094	-3.20661860899292
H	2.21589612766791	2.07953133794016	-2.35980587236692
H	1.19685360452399	1.66776402364665	-3.74713013473052
H	-1.21135542882548	3.46880498218861	-3.00868026711358
H	-1.35984932691178	1.77689561512327	-3.51648111208814
H	-2.07476140237787	2.31196625860605	-1.98729928888719
H	0.32107312813413	4.96777638350328	-1.37693442839812
H	0.56012947489632	5.89093308194071	0.91575575159576
H	0.61081569325135	4.37748184863751	2.85763470749006
H	0.42601080956577	1.93136331144066	2.56454123511582
H	0.09907396179388	-2.34538359255706	-2.91906898152513

TS-A3

114

C	-7.27643774315120	0.97311741147319	0.55572021995589
C	-6.45608478664789	0.01209855070524	0.00952515848946
C	-6.94281971951025	-1.26829479652624	-0.23627948777628
C	-8.25005280324393	-1.61484013918710	0.05956970100417
C	-9.06908442780737	-0.63439674650811	0.61146825151991
C	-8.59689988006750	0.64537241661075	0.85897716470083
N	-5.92876021567047	-2.04346634897849	-0.78904462025086
C	-4.75988400302995	-1.33523315549977	-0.91339604245660
C	-5.00712631975518	0.08983759224735	-0.41425693602510
C	-3.62041811663027	-1.88065093104974	-1.40512559868112
C	-2.38515619344522	-1.17804391556050	-1.56600428268862
C	-1.24081087557437	-1.67605615098886	-2.03493267678487
F	-1.18252325671335	-2.98327707089735	-2.43306176854201
C	-4.12569627755067	0.44132689616527	0.78757645642752
C	-4.84458814826240	1.11936183312230	-1.53642177686973
C	-6.06070753616527	-3.41468050974764	-1.18060094720284
C	0.06255541926314	-0.98391797024003	-2.24615827731941
C	-0.00610326592489	0.49938048311934	-2.02689502128219
C	0.20501094690833	1.23812917795820	-0.92831544974440
N	0.47585994587535	0.82997078573973	0.37286986868267
C	0.93520036172614	1.90497026238867	1.12851014334413
C	0.87116274448810	3.07782596359200	0.38206054082720
C	0.22860039114374	2.77106333623316	-0.95013660785497
C	1.30724306995455	4.26557348477421	0.92393560332625
C	1.81437978755859	4.27204861954727	2.22426475862702
C	1.85453864114459	3.10035947717375	2.96374520645288
C	1.41148123046532	1.89263061604799	2.42768728382111
C	0.04050197168089	-0.37951914903161	1.01846926867222
C	0.99487555462174	3.35146021808029	-2.12956824093972
C	-1.21612634319584	3.29077900034454	-0.94842106417800
C	1.18879904130632	-1.73230040543489	-1.49947862601425
C	2.37482628232571	-1.05514941693252	-1.18882860932631
C	3.31958160232337	-1.48099528806701	-0.26972617083652
C	4.51983882461858	-0.83890491442052	0.00090094740631
C	5.07298272237288	0.45266696667232	-0.58882205757318
C	6.38584111681813	0.57478516118912	0.14245597695714
C	6.52997516196964	-0.49557021661185	1.01259342601624
N	5.39381093256398	-1.31684205933237	0.89962645440104
C	7.64323476593253	-0.65103661893375	1.81623612463232
C	8.63440826065272	0.31955940841575	1.72111504110310
C	8.50533396071142	1.39782436720999	0.85672843821587
C	7.37318561781499	1.53251722949288	0.05823675672374
C	5.31825702861199	0.33419194311580	-2.09683005091239
C	4.16712188361539	1.64396862896460	-0.26124698917809
C	5.19484927217823	-2.52502836058604	1.66037759303707
H	2.57402344951252	-0.15358316888621	-1.75902027076757
H	0.84219229582996	-2.42006061131022	-0.72656199091384

H	-2.32444663376083	-0.13196052562671	-1.30083995984501
H	-3.63361549217682	-2.92124313585465	-1.70554953749799
H	-7.08933496229117	-3.73618285828759	-1.03994787359002
H	-5.79945240808304	-3.54152848546007	-2.23563538695577
H	-5.40919996842940	-4.06036966975043	-0.58261559754565
H	-4.42770458774030	1.40970155392090	1.19214521023056
H	-4.23252211127497	-0.30408431339394	1.57704283673385
H	-3.07136371524199	0.50306941709394	0.51470860692679
H	-5.47924663217400	0.86729201625881	-2.38714461432664
H	-5.13943926583990	2.10691418536811	-1.17470084410002
H	-3.81334367914281	1.17940753242574	-1.88594643575188
H	-8.63817333878809	-2.60862366591116	-0.12423513968853
H	-10.09643725160888	-0.88162346868261	0.85111148391942
H	-9.25459218984746	1.39049886417068	1.28851275342598
H	-6.90599066458935	1.97420858134205	0.74948587723024
H	5.05668517700181	-3.37836507056990	0.99302956191412
H	6.06794832036206	-2.70745651275777	2.27947538682952
H	4.32018661846352	-2.43291902310507	2.30810787944967
H	7.27572124387341	2.37705740907634	-0.61452631343805
H	9.29197532901606	2.13998946741422	0.80460077800516
H	9.52120135565631	0.22883713202383	2.33611493141076
H	7.75936794802038	-1.48382349315507	2.49774092514808
H	5.83473805278681	1.22936125469241	-2.44686382501632
H	5.94550355991688	-0.52861017465324	-2.32690484516473
H	4.38542893144514	0.24556135389292	-2.65350978257054
H	3.20133643040501	1.55760285157068	-0.75642669500482
H	3.98692624018178	1.72815136504557	0.81098477255387
H	4.64435675116722	2.56553936499632	-0.59844245942372
H	3.09513810916910	-2.37966149794055	0.29480907068101
H	-0.23556366704353	1.06999490493728	-2.91890624055123
H	-0.50419430884477	-0.12519134310139	1.93281740072288
H	-0.63113955594647	-0.92742444327587	0.36253409630447
H	0.88067851606751	-1.02623984265406	1.29082085912477
H	1.03681844684067	4.43983318462087	-2.05161158446437
H	2.01794110414276	2.97412794222445	-2.16448184042082
H	0.50732708991573	3.10931928405001	-3.07566487982650
H	-1.22905041622195	4.37697142365833	-0.83500623088776
H	-1.71451826869831	3.03084457488137	-1.88527885678933
H	-1.78434252529876	2.85755413371822	-0.12286061751276
H	1.25596122183106	5.18808457991478	0.35573415504286
H	2.16769341091607	5.19774060190827	2.66111155079844
H	2.23762872789522	3.12005433988924	3.97722389565750
H	1.44968273410725	0.98030708582885	3.01046023810600
H	0.27309380982763	-1.09077729647536	-3.32341722559672
H	1.54796505876600	-2.64402164587823	-2.37615076912287
N	1.66172848335662	-3.58973048431622	-3.42448020099995
C	1.99008786982127	-4.96195224911530	-2.97259380917287
H	0.70683986572469	-3.58324926607552	-3.77068851743846
C	2.53411764034788	-2.97816445838327	-4.44656602002514
C	2.37444356029724	-3.64605331750035	-5.80228166721051

H	2.17996573775215	-1.94343757965339	-4.53134579213147
C	3.98134599707865	-2.93702980946774	-3.99306334872313
H	2.68536681735484	-5.39781113484603	-3.69702366979989
C	2.66411632862972	-4.94711126262063	-1.60981541427165
C	0.72751464655366	-5.80320425986614	-2.95479670175091
H	0.94906118931952	-6.81313657030656	-2.60753684426312
H	-0.01731713625379	-5.36883581640091	-2.28325973706194
H	0.28803533365187	-5.87996800818353	-3.95211129679091
H	2.96999249076226	-3.12376028445782	-6.55303583212557
H	2.71167485811349	-4.68487024881763	-5.77924417093335
H	1.33303069939503	-3.63062762069239	-6.13197988190220
H	4.57024446861055	-2.37091019010320	-4.71604411540184
H	4.08360185241297	-2.45407948726533	-3.02002227107266
H	4.41515463408907	-3.93746355255614	-3.93284775054496
H	3.01260412464874	-5.95056976360327	-1.35929568910513
H	3.52155318802461	-4.27354911456101	-1.58919605887486
H	1.95950843393281	-4.63567334524999	-0.83529874574709

A3

92

C	-7.24280168480478	1.51823331989716	-0.44496407076829
C	-6.52792736225224	0.35938111925618	-0.24287516299823
C	-7.17848477262837	-0.80642124599018	0.15002183125823
C	-8.54799132917645	-0.83896634432388	0.35009325299382
C	-9.25908691258153	0.33861472358608	0.14239258193637
C	-8.62282676433790	1.50660435194446	-0.24966658249031
N	-6.24403073221509	-1.82851179027565	0.28151566299120
C	-4.97610400980870	-1.39611698286436	-0.00839474860482
C	-5.04782461630367	0.08628113334419	-0.38159590888874
C	-3.89233125012160	-2.21181506990630	0.04677154459480
C	-2.55692645459180	-1.80789892436314	-0.25347346962155
C	-1.46698230943549	-2.58256705396428	-0.22917335583381
F	-1.56722225237518	-3.89059621745925	0.10159149691296
C	-4.25924303080198	0.96279198031923	0.59602385326573
C	-4.59923337095764	0.33412116491351	-1.82482220665293
C	-6.54642713052831	-3.17744801758789	0.66002755443757
C	-0.07610991300214	-2.19691407211338	-0.59028315985820
C	0.07951902653862	-0.78324446271977	-1.07033702439528
C	0.23769316923083	0.36935835975371	-0.40482354639120
N	0.17460149794379	0.64249737211164	0.96134252491340
C	0.70651537339556	1.89905038226436	1.22740288676583
C	1.04945370978106	2.53959133956935	0.03940363800349
C	0.62081256083559	1.67350348509698	-1.11953111093598
C	1.63968008223182	3.78309794309525	0.06955747986270
C	1.88611369213925	4.38841036707515	1.30353186861425
C	1.51967754393723	3.75055560239886	2.47870361408388
C	0.91896286047879	2.49261187155830	2.46170277741017
C	-0.57489600435157	-0.04722997291108	1.97981795582542
C	1.73570420117471	1.48387724342312	-2.13850371664061
C	-0.61899039661090	2.27885858470499	-1.78900452292314

C	0.93632128344020	-2.58189881230340	0.54695926309619
C	2.30191707730999	-2.22397130874265	0.13464920556314
C	2.99467647569574	-1.18435155751228	0.64356398250785
C	4.26549524202424	-0.75039653654846	0.18720562629661
C	5.19151268752313	-1.42410632978901	-0.81088311778248
C	6.37481814399515	-0.49329932559271	-0.78686239855623
C	6.11131174443740	0.55437569877705	0.08082648764329
N	4.82682323899786	0.36041049009083	0.63273328354608
C	7.00504487448632	1.58141478614741	0.31671961654772
C	8.21150984437839	1.52769111601535	-0.36919627363580
C	8.49374450080860	0.48717302323608	-1.24674966385292
C	7.57535192334092	-0.53500235058666	-1.46432736872046
C	5.59288818350071	-2.82639026736998	-0.33789729382789
C	4.58522413947344	-1.45204605086824	-2.22209775971238
C	4.25002048009383	1.29600254435082	1.57970793236653
H	2.72607555766598	-2.80325863732435	-0.67830551150819
H	0.84988725112999	-3.66146678561053	0.69541775343305
H	-2.36143563427998	-0.78664906117121	-0.54721518870038
H	-4.03792708061765	-3.24777063504294	0.32742269258974
H	-7.61534668759080	-3.27793321732883	0.82816111193226
H	-6.25151526937439	-3.87787893104992	-0.12722174653757
H	-6.02284128920842	-3.45092781671040	1.58118237193813
H	-4.44346695844910	2.01639001718559	0.37497156874693
H	-4.57106279868129	0.77327775868331	1.62432959540148
H	-3.18555696914492	0.78411922870520	0.52318703638175
H	-5.16186781273841	-0.29284138159953	-2.51776801098425
H	-4.77813644461631	1.37825758325543	-2.09101605991452
H	-3.53750315348574	0.12566946951898	-1.96160038012892
H	-9.06442871925803	-1.73990296812683	0.65594321542515
H	-10.33225026836528	0.33749312156207	0.29150517440910
H	-9.19918358133781	2.40993777276634	-0.40492716431012
H	-6.74512318601073	2.43169135137973	-0.75322923147768
H	4.48622091756439	0.99342918609519	2.60146991443920
H	4.66457698098172	2.28279069858594	1.39183010469784
H	3.17299869940438	1.35857334788530	1.44879481443903
H	7.80723609851164	-1.34096184509012	-2.15092909165646
H	9.44374546015686	0.47180299079411	-1.76622602789042
H	8.94357386813886	2.31021550739041	-0.21380830922010
H	6.79595674182228	2.39060584065383	1.00395930878285
H	6.34270065597922	-3.23145237286677	-1.01868901088248
H	6.02576950856693	-2.79480910885184	0.66300400297244
H	4.74100194043469	-3.50570870251554	-0.32381729700238
H	3.71692113911146	-2.10610188423810	-2.28471889373304
H	4.28853260507080	-0.45314935346298	-2.54353309371093
H	5.33875125371765	-1.82125586843601	-2.91887769803549
H	2.52835088140925	-0.60603085631440	1.43238141762316
H	0.14273229004232	-0.68534731192505	-2.14748257012010
H	0.07424675876618	-0.44546452811200	2.76802406558257
H	-1.27991092502006	0.64535312033086	2.44962433160934
H	-1.14153976888229	-0.86339814491797	1.54072703814645

H	2.02783962057179	2.44614348744578	-2.56412165781743
H	2.61375202744515	1.03341875929661	-1.67222361006491
H	1.41636884519440	0.84213335959389	-2.96134803885919
H	-0.38360397940078	3.25870046868450	-2.21026457336193
H	-0.97148514461442	1.62909162910345	-2.59326627171825
H	-1.42855880280039	2.40533152173777	-1.06758355487542
H	1.90281808960265	4.29577903408714	-0.84946845059332
H	2.34702622001921	5.36755792018402	1.34225823916970
H	1.69763710449473	4.23713866243319	3.43046020664367
H	0.63605082203434	2.00336104804915	3.38563199205837
H	0.20633436854129	-2.83826358354238	-1.43659123899959
H	0.67512850719086	-2.08983920030758	1.48057426733486

2c

60

H	2.44818234368241	-1.68087423603354	-1.30468377796128
C	3.56883230065905	-1.70136392000836	1.25337603508223
H	3.87861019358236	-1.10285595332257	-2.16843648115915
C	3.53716456755302	-1.63754446814187	-1.28104693086656
H	3.91823065297919	-2.65864901747889	-1.33341421759825
H	2.48065943451771	-1.73971562341599	1.30605552348466
H	3.94424146100172	-2.72578187031058	1.24049693205319
H	3.93916856064521	-1.21646142414857	2.15760267895443
C	4.05470599604570	-0.96845975725299	-0.00280892709280
C	5.56160235606767	-0.90625289861080	-0.01945962145170
C	6.50275201665312	-1.91198768830433	-0.06028361065682
H	6.20700934831625	-2.95474229074226	-0.08713100949227
C	7.85116127486302	-1.56605692537601	-0.06697673966929
H	8.60490693723855	-2.34267885619188	-0.09914104252086
C	8.23908741476076	-0.23344155341279	-0.03385572437578
H	9.29231638784879	0.01843433007790	-0.04096122008766
C	7.29912565912246	0.78933444435480	0.00734606940719
H	7.61838993218296	1.82316317207621	0.02966132986724
C	5.96689999226802	0.42084803280659	0.01486880562612
N	4.82424753268205	1.23578684280097	0.05446669512744
C	4.86380985099000	2.67750402797777	0.10017786639204
H	5.89586686684959	3.00467072406137	0.17977951594226
H	4.42814825550616	3.10383592368363	-0.80602816827857
H	4.31436243100560	3.04429139829078	0.96891375010562
C	3.69527798781701	0.51035386342352	0.03998403212200
C	1.22874157792534	0.44319998121396	0.05040302264096
C	2.44416312650515	1.10852080499063	0.05801090175006
H	2.40390616578653	2.19031675308482	0.07456285035627
H	1.18545843329182	-0.63766936424565	0.03578689227811
C	0.00034402115120	1.07835376817767	0.05863158952527
F	-0.00117136354250	2.42260707174128	0.07599324082545
C	-1.22660596989281	0.44043041235682	0.04598119165664
H	-1.18079304536902	-0.64035145322882	0.03271155405181
C	-2.44349423320101	1.10305992911462	0.04798075579628
H	-2.40553579846700	2.18495615108102	0.06216128553284

C	-3.69326369939172	0.50218338615919	0.02601766000189
N	-4.82403778522646	1.22490641573700	0.03669443365624
C	-4.86800849222287	2.66639897584447	0.08415468991979
H	-5.89899733470163	2.98947577314432	0.19099145659452
H	-4.45672894163195	3.09598954495275	-0.83199682840594
H	-4.29885724201352	3.03396353531259	0.93954875898935
C	-5.96449198985114	0.40737411484471	-0.01143950256272
C	-7.29741790138207	0.77302754390950	-0.02806718624186
H	-7.61885018004792	1.80624850551335	-0.00889626534186
H	-9.28856633880396	-0.00230685568737	-0.08973500170195
C	-8.23484943193446	-0.25182448616401	-0.07541783078796
H	-8.59554939555133	-2.36186783194262	-0.14297075058077
C	-7.84375086957009	-1.58357559149588	-0.10588969600912
C	-6.49464225526783	-1.92657000141651	-0.09072495052080
H	-6.19647140135201	-2.96867354739915	-0.11596815616666
C	-5.55598666368680	-0.91877079104045	-0.04386036187698
H	-3.85986325787884	-1.11238219775340	-2.18298895847170
C	-4.04907901593555	-0.97748837359451	-0.01853042268517
H	-3.94543379443263	-1.22413551888095	2.14266898247478
H	-3.90212130951385	-2.66789206203763	-1.34762515957412
H	-2.43374008007872	-1.68799256326500	-1.31057103524479
C	-3.56855304012415	-1.70848965128235	1.24085320121702
C	-3.52288968267852	-1.64619945704483	-1.29337054928520
H	-3.94109287580555	-2.73393504602111	1.22632824347496
H	-2.48059068994249	-1.74377515148057	1.29982618176190

TS-A4

114

C	-6.65316093619774	1.02882670786352	1.27695466826979
C	-5.84151252765666	-0.05221440000741	1.00915861305425
C	-6.16842909698482	-1.30447254393804	1.50768826580599
C	-7.30157257163515	-1.52782208422397	2.26590268450743
C	-8.11488891409152	-0.43071913050967	2.52766315210099
C	-7.79820118630648	0.83111419919089	2.04373778982369
N	-5.17863075543760	-2.21796979408810	1.10513538059411
C	-4.23539071350763	-1.63301583440973	0.35196854442723
C	-4.55781621069461	-0.14670609922518	0.22283174458956
C	-3.18838883555386	-2.36048186387032	-0.19852187738540
C	-2.16954878437230	-1.83354678853245	-0.97096813413469
C	-1.23036471721515	-2.58914283850890	-1.69029399628620
F	-1.24842745312701	-3.93593548870394	-1.39111658609572
C	-3.47838863965112	0.73159639451542	0.86311679311905
C	-4.79331255613078	0.25817833272666	-1.23547662686693
C	-5.19276386878775	-3.61879612509251	1.45054562602051
C	0.18776541012451	-2.10104159651113	-2.03918692487824
C	0.16937987986599	-0.63932427235772	-2.37788276601334
C	0.08111186854487	0.46651914257699	-1.61243269643270
N	-0.03923683738040	0.63741051966531	-0.24352827790202
C	-0.03689436625900	1.99335321101382	0.08915383344683
C	0.02094691623918	2.77124798616469	-1.05947844467594

C	0.08645337465112	1.86372686686858	-2.25911384970147
C	0.02098027988495	4.14518375759275	-0.96682241445015
C	-0.03392139434702	4.73750634068394	0.29454852023775
C	-0.08181276407649	3.94941230481126	1.43398226961180
C	-0.08277996503111	2.55919443415397	1.35186600070524
C	0.00139425285444	-0.33579435002727	0.82022437104283
C	1.36593334037371	2.10431965354386	-3.05854959468510
C	-1.14322729136145	2.05087234835113	-3.14812327706724
C	1.23376208502735	-2.54806326202226	-1.05020828677528
C	2.31972878126317	-1.82019363634439	-0.75768162315459
C	3.36678196996915	-2.18820867744679	0.14555199395832
C	4.42228102251798	-1.40231655719547	0.47499502928318
C	4.76188676005654	0.00893632657668	-0.01248515613298
C	6.03756138161343	0.29130148182686	0.74772547873884
C	6.36055605983290	-0.80928177210723	1.53595941884263
N	5.39887967697132	-1.79743702518407	1.35669819011363
C	7.48966900162931	-0.81771478592452	2.33744241354665
C	8.29815756777728	0.31493118885803	2.32765786693514
C	7.98778347188962	1.41702188221973	1.54592997971230
C	6.84531065132575	1.40604047619236	0.74739045276037
C	5.03498731103739	0.04056734429465	-1.51911616121291
C	3.68658954412297	1.02712220732646	0.37582632719487
C	5.40130440420046	-3.07556547856957	2.00292936188171
H	2.39228689858874	-0.85023161280182	-1.23565805135292
H	1.10006552839619	-3.53856394792140	-0.62455016229902
H	-2.10348822025889	-0.76780497180948	-1.14802717818124
H	-3.17337983433773	-3.43056556685356	-0.03365139087780
H	-5.94472721409929	-3.79127284944318	2.21503029385239
H	-5.42701397724160	-4.23329289124807	0.57803326680639
H	-4.22191643559669	-3.91777652684713	1.84741811524298
H	-3.81923816463699	1.76792405900239	0.88165069140577
H	-3.28236849232805	0.42472305505860	1.89136230766506
H	-2.54584668441341	0.69978914441679	0.30155498750057
H	-5.54919362325348	-0.37403074210699	-1.70465424235564
H	-5.14765680115294	1.28951476665477	-1.27006336290388
H	-3.87431553736744	0.19966757164441	-1.81919972507704
H	-7.56419794888961	-2.50645797269150	2.64603531615445
H	-9.01105367328780	-0.56804774877981	3.12005070119731
H	-8.44875248896571	1.66818697210662	2.26374079257309
H	-6.41144559959315	2.01685097441434	0.90147235744827
H	5.39184681738857	-3.88296861272776	1.26451330558690
H	6.29736166054066	-3.17564554528248	2.60990681836664
H	4.52685230744066	-3.19048686667311	2.65185372274609
H	6.60192610035201	2.26787645464114	0.13495391196217
H	8.63359757967369	2.28604546380449	1.55542615543134
H	9.18805934811151	0.33030805092872	2.94563328667735
H	7.75099532358310	-1.66695115863340	2.95603893808780
H	5.39790670878789	1.03022444759801	-1.80563046991784
H	5.79973670618319	-0.68970510308257	-1.78719922053342
H	4.13838857096046	-0.17625957884167	-2.10120721336105

H	2.74637296810321	0.84129423433537	-0.14312535216019
H	3.49732283896690	1.00107324212964	1.45024932504404
H	4.02035535347598	2.03513508036525	0.11987599342984
H	3.29472096672629	-3.16854377079277	0.60521179428084
H	0.28178861226909	-0.41751973853098	-3.43354241235174
H	-0.79142849763125	-0.11836910607902	1.53948018977047
H	-0.13362758496376	-1.33803873643307	0.43614035351734
H	0.96328189191494	-0.30733779084205	1.34048457144301
H	1.37341653153186	3.12131769846548	-3.45648377502758
H	2.24815310373327	1.98066502289649	-2.42893921472790
H	1.44354129063091	1.41056080713506	-3.89838900041219
H	-1.17023652182497	3.06575224542888	-3.55033945294899
H	-1.12195712621795	1.35194388418117	-3.98803100642913
H	-2.06259291631860	1.88993062645945	-2.58063866512356
H	0.07009188496317	4.76300691192422	-1.85707772824398
H	-0.03017077950622	5.81653151688981	0.38496671031152
H	-0.11238139359076	4.41996689873126	2.40950984061961
H	-0.10384858964245	1.95413363118223	2.24908723023456
H	0.41532483675472	-2.62502761451338	-2.97960674166501
H	-1.73962315659995	-2.49493857770475	-2.88693446568271
N	-2.20647349386569	-2.37818509387675	-4.23813006856184
C	-1.55611863155267	-3.30325122370467	-5.20065352077026
C	-3.66595097935806	-2.28909543315443	-4.02212956995812
H	-1.88189731580487	-1.43888953994545	-4.44961300154431
C	-2.14271784706679	-3.26542866814030	-6.60310423992680
H	-0.53612787499962	-2.90833147189210	-5.26202441133708
C	-1.44582240182572	-4.72507285764372	-4.67637198809755
C	-4.40521164257918	-1.49924373310139	-5.09104628866739
C	-4.30905661934688	-3.63143371985952	-3.74044807208361
H	-3.73687449224085	-1.70756362362902	-3.09447516132143
H	-4.47917920826854	-2.04228113978751	-6.03238238847817
H	-5.42062140228512	-1.28776066222319	-4.74910759222995
H	-3.91823188385997	-0.53980170570675	-5.28514088949116
H	-5.32853837673982	-3.46630043044709	-3.38744945140123
H	-4.37248580412445	-4.25246771121823	-4.63493010694694
H	-3.76728161538341	-4.18209928791940	-2.96975831966865
H	-1.49605351151693	-3.83126913774942	-7.27632370159325
H	-3.13161349991275	-3.72527252537565	-6.63986268124046
H	-2.21704105684995	-2.24718501144531	-6.98920834203341
H	-0.75311345187097	-5.27955386502503	-5.31173030164522
H	-1.06275390252909	-4.75217704138253	-3.65634828164625
H	-2.40028718134385	-5.24952654874299	-4.70100084355217

A4

92

C	-7.71717359607753	0.71726900931486	-1.00365310893605
C	-6.79292163787448	-0.13598537618687	-0.43806216911899
C	-7.20315773579322	-1.36872084244271	0.04145315902376
C	-8.51310725991947	-1.80466276159498	-0.00749278724829
C	-9.43490297614745	-0.93635812583470	-0.57765726260759

C	-9.04397717951079	0.30412298954713	-1.06955208551433
N	-6.07393252591394	-2.04077182550928	0.55913274075931
C	-4.98048973960940	-1.30670552884208	0.46159999158575
C	-5.31082126961473	0.00886579411357	-0.22052756390468
C	-3.71777710002791	-1.75164611189630	0.93521714005289
C	-2.58651734081815	-1.02604384033811	0.87744904815403
C	-1.27821123522273	-1.52090503124664	1.33855636563248
F	-1.42045534417899	-2.75254983128768	1.94488698291905
C	-5.01886712255753	1.22015365516797	0.67473526205842
C	-4.58123428900305	0.10720429235076	-1.56899054563980
C	-6.19220430178381	-3.36997331533758	1.12440385905294
C	-0.26712740483668	-1.64767864698729	0.15945862160663
C	-0.22543776836087	-0.41625817226199	-0.70591615475337
C	0.15505707505262	0.85793602976049	-0.48616868187316
N	0.63278769107004	1.49768562243127	0.64202563353123
C	0.80758239729815	2.86188465106939	0.41040296642642
C	0.51282115080755	3.16250271570825	-0.91330637357765
C	0.11227978150946	1.89584583583371	-1.62328070565241
C	0.63097070279495	4.45461462526224	-1.37425013540189
C	1.04670124981138	5.45177457965617	-0.49257235193928
C	1.33272267592996	5.13963842765614	0.82723869707397
C	1.21548646699216	3.83673903246806	1.30469198522135
C	0.88883631512410	0.94737577630297	1.94311199520792
C	1.13273039449062	1.54056555853817	-2.70630003072933
C	-1.28281759175028	2.01904782107819	-2.22830214111897
C	1.05060263892006	-2.17273262854878	0.64529487470595
C	2.24054839931457	-1.66151355888368	0.29206745508773
C	3.51508590006538	-2.14607749675032	0.71850977291111
C	4.71105619550853	-1.59077479683497	0.39463044410424
C	4.99695806633968	-0.36452922720140	-0.47409584728432
C	6.50385737257199	-0.28392242427697	-0.39539871573952
C	6.98225349492486	-1.32659908261634	0.39206954208355
N	5.90840500126502	-2.09009438953316	0.83914332979912
C	8.33569621857990	-1.49017866142371	0.63310999537948
C	9.20944375139365	-0.57282140490128	0.05752038934886
C	8.74551217442288	0.46908661235416	-0.73103734514015
C	7.37858710173006	0.61646827983354	-0.96048117209991
C	4.56020689226037	-0.59131557274481	-1.92424579333577
C	4.37377026025026	0.90937463789237	0.10392406388439
C	6.01587410647153	-3.24816480136908	1.67615042903102
H	2.22232612076330	-0.80415921629271	-0.37063987713086
H	1.00317276983378	-3.04595848465995	1.29049714491091
H	-2.55617613650667	-0.03475052075828	0.44800859008827
H	-3.65445823223654	-2.73187664169349	1.38984975683879
H	-6.59964190054047	-3.30988865025063	2.13511305797400
H	-6.86674709489495	-3.95335407461201	0.49969692920050
H	-5.22673912852195	-3.86399717776907	1.14767667494520
H	-5.42452381132144	2.11387727986712	0.19911202009767
H	-5.49234616460070	1.11365038879191	1.65173730423870
H	-3.95108051333847	1.37516314150570	0.82092797256477

H	-4.81679742323561	-0.74794070975456	-2.20445360199187
H	-4.90840331782930	1.01192817468435	-2.08308663358010
H	-3.50059244736576	0.15819159335446	-1.44150425576858
H	-8.82276564536051	-2.76609310247532	0.38116362083284
H	-10.47465603138414	-1.23227291004524	-0.63767720221690
H	-9.78604953689751	0.95911295508358	-1.50878530389411
H	-7.42598390948919	1.68724434893442	-1.38955949118437
H	5.55971184035673	-4.11784990901522	1.19400601887949
H	7.06405942513923	-3.46815225475698	1.86069381606550
H	5.52030358695492	-3.08503645586735	2.63848741704744
H	7.01466167938400	1.43212913268441	-1.57629051283820
H	9.44629126837367	1.16957206091998	-1.16761474305291
H	10.27336920480760	-0.67946320097912	0.23265309572088
H	8.71875086044520	-2.29572353515561	1.24664736568037
H	4.86052501377273	0.26131877835585	-2.53754006549749
H	5.03300924734687	-1.48524432043462	-2.33353698083238
H	3.47955364694500	-0.71064078746013	-2.00821434478109
H	3.28450913288323	0.88114056005796	0.07401101510745
H	4.68533265673547	1.05428557326905	1.13966943638444
H	4.70516160320427	1.77666070410945	-0.47114385858685
H	3.51444740097196	-3.02044380051653	1.36104141452829
H	-0.52367829514251	-0.59941714568536	-1.73188115371951
H	0.06904954428100	1.15672497661701	2.64233664934069
H	1.06621663884303	-0.12219042677126	1.87362162460952
H	1.79710576937676	1.39494772084159	2.35125591839836
H	1.13628087172452	2.30545655360028	-3.48582883510452
H	2.13817239856264	1.48062186858984	-2.28759714592429
H	0.89204298373111	0.57910777956284	-3.16521527886958
H	-1.29603974262098	2.80796152602949	-2.98329781738066
H	-1.58480939022320	1.08705822164910	-2.71141275228191
H	-2.01847640787428	2.27565269038594	-1.46252648869232
H	0.40880423019143	4.69897215158257	-2.40749999770051
H	1.14599300356479	6.47271177981423	-0.83936961178376
H	1.65273471015804	5.92193634097883	1.50516380775744
H	1.43502020132032	3.61040116688853	2.34021651275932
H	-0.72553666922411	-2.43499856455985	-0.45474059620049
H	-0.88656106696203	-0.82928407013396	2.09152961201633

2b

C	-4.00379063829735	-0.96071891253658	-0.00808626207949
C	-3.68563883338186	0.52859704439369	0.02144538178117
N	-4.83639834853372	1.22151706004328	0.02335724469949
C	-5.95507666095871	0.37358827714440	-0.01152277051803
C	-5.51186537583864	-0.94133985201751	-0.03073226910410
C	-6.42396765972648	-1.97379553482609	-0.06477763931366
C	-7.78180757961580	-1.66703592569298	-0.07984663419576
C	-8.20788672936411	-0.34599996961218	-0.06154555552164
C	-7.29730078170317	0.70349848443464	-0.02734615312520
C	-2.45133845562298	1.15941874453596	0.04067891225485
C	-1.21385781301251	0.53095434663361	0.04242996329565

C	0.00005872867341	1.19910150640146	0.05297275965631
H	-0.00114987267121	2.28609315992497	0.06289663919631
C	-4.91786240140879	2.66119546123599	0.05448895844088
C	-3.50369549354807	-1.66767215266354	1.25721167739629
C	-3.46415155569441	-1.62949358160254	-1.27726851670076
C	1.21545944234918	0.53362792436021	0.04565792766338
C	2.45144152134545	1.16504624306709	0.04751664730449
C	3.68741698941124	0.53745617197292	0.03092994788149
C	4.00963691417983	-0.95100220736786	0.00196313035724
C	5.51766401415544	-0.92757109505480	-0.01743371303094
C	5.95732615951122	0.38853688692013	0.00276789461110
N	4.83626973096554	1.23348646497953	0.03523313975332
C	7.29868827316909	0.72204032433296	-0.01021165638641
C	8.21215653113264	-0.32499880763879	-0.04255589384386
C	7.78965784728370	-1.64716607338970	-0.06187552666373
C	6.43263313319905	-1.95755817666415	-0.04966674495886
C	3.50840156491664	-1.65995960355072	1.26575695890589
C	3.47433348494868	-1.62090084044106	-1.26849635355064
C	4.91401230487440	2.67336819077429	0.06636136781397
H	2.38471447469232	-1.64310177653332	-1.28745908265988
H	3.82306829509122	-1.10163480402997	-2.16218821693868
H	3.83520690818160	-2.64977757868016	-1.31251491998450
H	2.41967798624307	-1.67372606939211	1.31802465258845
H	3.86160630204860	-2.69239324941945	1.26335791850857
H	3.88948065348362	-1.17422220860776	2.16506528415377
H	6.10997072394259	-2.99254755506700	-0.06580572034482
H	8.52271990752661	-2.44359521318367	-0.08733982138969
H	9.27154273577626	-0.10055943628251	-0.05345574901912
H	7.64437418362659	1.74752488584964	0.00204329918327
H	5.95542051465912	2.97454031286196	0.12436699271466
H	4.47507386263511	3.10296270487320	-0.83681458354043
H	4.39005254987326	3.06350863232528	0.94088089441209
H	2.44438533052130	2.24950420511884	0.05793259889246
H	1.17614272673038	-0.55061572099723	0.03575210289117
H	-1.17188967167372	-0.55320271764543	0.03314624362771
H	-2.44699909422658	2.24388732221967	0.05057277505608
H	-5.96010574603713	2.95978277159334	0.11113664064077
H	-4.47894686664554	3.09208292694485	-0.84808658121047
H	-4.39601256213480	3.05242002985084	0.92977107509854
H	-7.64574572601905	1.72805847184307	-0.01598710050419
H	-9.26784791823706	-0.12442489121994	-0.07480265391524
H	-8.51267665774533	-2.46542691097841	-0.10684227261546
H	-6.09842713903187	-3.00789192679445	-0.08005336654008
H	-3.81364086773227	-1.11194361104038	-2.17167598566325
H	-3.89206051586637	-1.18501036573766	2.15507147305276
H	-3.82119342750515	-2.65969614922271	-1.32135915288967
H	-2.37445194840475	-1.64753677406674	-1.29490276814240
H	-3.85055622519198	-2.70226395809940	1.25224584683495
H	-2.41518522931781	-1.67466090457902	1.31497931568386

TS-B1

92

C	8.46042693349572	-0.08845181576390	1.12630723004429
C	7.36274884553310	0.28712759403083	0.38405865893259
C	7.51900405530063	1.10207414590696	-0.73095494261385
C	8.76105823163911	1.56053130854809	-1.13232649025592
C	9.86211752489177	1.17431801245861	-0.37496949867792
C	9.72097915674681	0.36185636101105	0.74039529121874
C	5.90058811206889	-0.04829223994206	0.55781270233534
C	5.27430238805149	0.71206871505885	-0.60784781812340
N	6.26530911699055	1.33347583924397	-1.30143755353642
C	3.95670705307712	0.81440026213628	-0.96317223885070
C	2.87809961671005	0.17292614404991	-0.32053340200571
C	1.57330051756183	0.29271111915520	-0.68279374511113
F	1.26194304607014	1.10161007259702	-1.71714471156932
C	6.03773704441750	2.12382806587288	-2.47921707111706
C	5.39311618731540	0.47019646487466	1.90608871580221
C	5.69382525499682	-1.56047884592351	0.42653705965638
C	0.48110733139456	-0.36733651695247	-0.07514451507122
C	-0.80926326829157	-0.26924327750353	-0.46604286015722
C	-1.88912470688722	-0.96946031431628	0.18760513980242
C	-2.97456370025540	-1.42768506179771	-0.63015480626174
C	-3.73660335425090	-2.53802877018765	-0.42370640518355
C	-3.62936846079766	-3.59933514583716	0.67116653290947
C	-4.76177394512252	-4.52868250851679	0.30160566250383
C	-5.36631706615448	-4.07746164866509	-0.86571684954642
N	-4.73990343583550	-2.89816950216312	-1.27440220170762
C	-6.42034261009849	-4.75577705006237	-1.45079693732131
C	-6.86341078107747	-5.91590344894485	-0.82391070931867
C	-6.27246361182431	-6.37546705191967	0.34364341522174
C	-5.20984289906938	-5.67799165292082	0.91413711234471
C	-2.29779377134852	-4.35261061090273	0.56418751582016
C	-3.82909235653368	-3.03146383316760	2.07598407963457
C	-5.09384598489211	-2.14775064582004	-2.44608295937594
C	-2.63848872185449	0.30090198819420	1.58206087219194
C	-2.49949935494651	1.63031707165955	1.17972639471498
N	-1.38381858313869	2.35872439498131	1.27752414201083
C	-1.53472179916141	3.61581097164057	0.66338818864780
C	-2.82201792815990	3.71753251809976	0.15740268662632
C	-3.57279977014899	2.45528999689716	0.48819440355595
C	-3.20937687501778	4.86527940020812	-0.49950073084112
C	-2.28529680216324	5.89864987581876	-0.63748601789628
C	-1.00257863933717	5.77888831196447	-0.12009171579277
C	-0.60081775091353	4.62582836580296	0.54644288992061
C	-0.18573719339995	2.03481399951591	2.01263280207848
C	-4.14263091354543	1.77338223105425	-0.75046604182638
C	-4.69338189973851	2.73915479960193	1.50002845369213
H	-1.05141932062430	0.34874355863838	-1.32511444337185
H	3.05713767014228	-0.48838308428225	0.51685384089343
H	0.73900036049279	-1.00359105783487	0.76784155215642

H	-1.55554430662164	-1.66253977614572	0.95198810461284
H	-3.17306913542060	-0.84614932391973	-1.52216027308236
H	-5.38628786119699	-1.12788458126211	-2.18097842747722
H	-4.25537931173619	-2.10031648131304	-3.14684663768144
H	-5.93266131136672	-2.62576167779018	-2.94422852998110
H	-2.28746360183854	-5.18049100911759	1.27572132373802
H	-2.16583993977270	-4.76465568850274	-0.43703245025755
H	-1.44672048329786	-3.70591405987384	0.78106814552896
H	-4.76323942467850	-2.47120443131894	2.14486230693317
H	-3.87677594065677	-3.85003597926996	2.79641943171560
H	-3.00733128993702	-2.37885365029223	2.37193726102837
H	-6.89512907581010	-4.41445006912679	-2.36162738861029
H	-7.68739779887770	-6.46759960423426	-1.25992470400611
H	-6.63708357757547	-7.28093390866480	0.81202281083232
H	-4.74582646082520	-6.04124082616497	1.82463609304422
H	5.52740930180150	1.53313479647458	-3.24439145805924
H	5.42759694254496	3.00198574644522	-2.24951544132252
H	6.99017644890643	2.45918532377820	-2.87986850415317
H	8.35244166690204	-0.72474471258972	1.99793600072396
H	10.59470057394857	0.07656416603344	1.31266196961815
H	10.84744116110659	1.51783687029903	-0.66578108152661
H	8.89147856741556	2.19729203753672	-1.99790661046135
H	5.93803280254382	-0.01898630735329	2.71578654302938
H	5.55198548250206	1.54616714077897	1.99186961786040
H	4.32991143521389	0.26905506611265	2.04156761476669
H	4.64471004957656	-1.83978100561180	0.52693326074079
H	6.05050356896214	-1.91872562879138	-0.54013572316464
H	6.25792293814801	-2.07452872224210	1.20717541794622
H	3.70783900673244	1.42943712984164	-1.81909347527417
H	-2.01006153587859	-0.03769301046255	2.39731117507848
H	-3.65527638061498	-0.06381406576644	1.63825202170179
H	-0.27886377202517	1.05232320426237	2.46338385196570
H	0.67811888018206	2.02968932293892	1.34629339023486
H	-0.03288797935952	2.77930960496590	2.79767991969715
H	-4.88810184461955	2.42485508459820	-1.20919592294245
H	-3.36511152887528	1.57554006712015	-1.48944031408079
H	-4.62764076336788	0.83170769537174	-0.48962959333728
H	-5.19571611492041	1.81399171991689	1.78860027677147
H	-4.30315212703963	3.22089942684963	2.39774744988864
H	-5.43069820664298	3.40516185953007	1.04842710151426
H	-4.21064222007466	4.97047466729738	-0.90161814819448
H	-2.57105407686866	6.80719867965227	-1.15274677914276
H	-0.30023739835327	6.59495211430248	-0.23677788015123
H	0.40154969945853	4.53367329011055	0.94474057675341

B1

92

C	-2.84990443398438	6.21415702580109	-0.44813975397119
C	-1.96322237701428	5.23023414993390	-0.02809721535032
C	-2.51893038908711	4.02821346458622	0.35984405432975

C	-3.87969594974930	3.77547234715784	0.34779214995121
C	-4.75064501208911	4.75954677936661	-0.07190477027664
C	-4.22063468455440	5.98274137266765	-0.47073559152191
N	-1.85324944105042	2.86336765960150	0.82538008871765
C	-2.69682647311418	1.89347608647961	1.06923446455574
C	-4.11281200167410	2.37963057083635	0.85064149328834
C	-2.32833967070560	0.56634237158207	1.57235226806618
C	-1.77435562142287	-0.49285779587509	0.51904009981681
C	-2.80902470732909	-0.98626668797754	-0.43208925666539
C	-3.44666970883567	-2.17105615393421	-0.38910129045402
C	-3.33846483878532	-3.29245031523022	0.64882317394207
C	-4.39602207049299	-4.25850550953859	0.16227947359553
C	-4.93828758091478	-3.78709818380883	-1.02900718985321
N	-4.34155283889956	-2.57465059759284	-1.35778750720925
C	-5.92004801657627	-4.48969918047320	-1.70572529604175
C	-6.34535469041383	-5.69623354651325	-1.15745074751986
C	-5.81039715368203	-6.17881625670876	0.02667669287571
C	-4.82523556936562	-5.45321101379823	0.69485159238547
C	-1.96266948834908	-3.96538331915218	0.57553284362200
C	-3.64603033966297	-2.82330841505766	2.06972882059900
C	-4.62024139145874	-1.82062853621772	-2.54163104095327
C	-4.91959956018738	1.52388466947564	-0.12306904369678
C	-4.80761419538015	2.40947137748221	2.22690112921861
C	-0.42008172535690	2.88638988660830	1.05405462371348
H	-0.77949664581388	0.68480912337828	-1.05970086474810
C	-0.58765427663139	0.02940338449329	-0.21378273172609
C	0.68208655795528	-0.27012390092130	0.09941920238502
C	1.83523621778575	0.25089813977947	-0.55554602333987
C	3.12292334532270	-0.00426729025478	-0.23578254414825
C	4.25239791904745	0.53694824096547	-0.89976994003864
C	5.55473177932239	0.34023702884599	-0.54884515053377
N	6.59266253463236	0.88557576715908	-1.24895516255925
C	7.82348061361842	0.57199908249072	-0.67372187586779
C	7.61014285034451	-0.22234613039170	0.44760972751982
C	6.12771531781621	-0.45541030723487	0.62202006924882
C	9.09605792047744	0.94236640967503	-1.07150976596600
C	10.16632553767076	0.49068183400230	-0.30574886874506
C	9.96761907484264	-0.30079689152469	0.81545370217587
C	8.67753134181062	-0.66308883541971	1.19756258319474
C	6.41864542017259	1.68643448312498	-2.42624720467515
C	5.65983078339372	0.10169963392780	1.96879836124010
C	5.81489344184331	-1.94955170157921	0.49732116278997
H	3.25549956813277	-0.68292193836945	0.59640688565036
F	1.57230470123580	1.10085444529959	-1.57827321244910
H	0.89061498463714	-0.94774552310610	0.92403426417927
H	-1.44629710600995	-1.30572412599192	1.16698820389799
H	-3.01030128275344	-0.33517416904048	-1.27603695525598
H	-5.17659124126065	-2.43791355098098	-3.24340548654391
H	-5.21223363477463	-0.92303834897154	-2.32906954968220
H	-3.68730388416062	-1.51541100491109	-3.02402806231462

H	-1.93698635315751	-4.82918184346826	1.24313334925074
H	-1.75961788175779	-4.31432662123595	-0.43770637477868
H	-1.15943987319197	-3.28621689467194	0.86646175952856
H	-4.59970733560748	-2.29315503218885	2.10825403793999
H	-3.71682013657560	-3.68517823024238	2.73607939788679
H	-2.86303055629701	-2.17232354363510	2.46292650168251
H	-6.35724080937041	-4.12589703257068	-2.62697415154459
H	-7.11270226904462	-6.26462868527559	-1.66926914027245
H	-6.15898617325875	-7.11934074570715	0.43410185017919
H	-4.40613505567897	-5.83306734028102	1.62063505335198
H	5.85898111815998	2.59921447521912	-2.20015107380737
H	7.39096588464750	1.96715031973908	-2.82186145243456
H	5.87820265800830	1.12829330441433	-3.19591713388054
H	8.52365613373436	-1.28257255863000	2.07455145187715
H	10.81775120536957	-0.63813269455935	1.39493592558929
H	11.17333855233523	0.76652296846237	-0.59482826344230
H	9.27233624909561	1.56199575095417	-1.94148630788961
H	6.17207955788687	-0.41947101830358	2.78011728217309
H	5.89168732636623	1.16482596987721	2.04901941992359
H	4.58573794899813	-0.02521171483401	2.10736983035954
H	4.74718361770998	-2.15130421810521	0.58868108408263
H	6.15324004349787	-2.33676699186182	-0.46471467512506
H	6.33338199212504	-2.49929894201391	1.28553983599936
H	4.04693088207146	1.16342030887665	-1.75896491113324
H	-1.55533865369724	0.67537157139658	2.33807176777968
H	-3.20581927579737	0.12158017755773	2.04095920724887
H	-0.10190161198938	1.97133195192564	1.53947338680737
H	0.10298838073328	2.96430448320296	0.10097386021058
H	-0.18842888829046	3.75427489900998	1.67317052642992
H	-5.92800329448434	1.93461381822055	-0.19308400695133
H	-4.47747920826284	1.53164587115360	-1.11921033675212
H	-4.98659958326560	0.49096611186405	0.21830098505926
H	-4.94488288099910	1.39935066781813	2.61514809128535
H	-4.24207535004230	2.99708193128902	2.95198242542258
H	-5.79088125034823	2.86689027829335	2.11089506860490
H	-5.82112518653486	4.59232794274734	-0.09411370265617
H	-4.88601318872911	6.76887801968459	-0.80547052613306
H	-2.46422253792586	7.17496397001504	-0.76494183328375
H	-0.89568910495387	5.40830321171835	-0.01721323744019

TS-B2

114

C	8.02298773050115	-2.08306024227729	-2.04170950010905
C	7.25728434837623	-1.22651410891745	-1.28301972273371
C	7.85052863811899	-0.15489067549753	-0.62402355495530
C	9.21102133600645	0.08532762057178	-0.70612410879946
C	9.97412572484085	-0.78759507651040	-1.47540198634037
C	9.39524157018499	-1.85977522855888	-2.13737657004088
N	6.86927435568834	0.55268426241909	0.06743129459974
C	5.62999175711016	0.00375840958439	-0.11059562388662

C	5.77205990741528	-1.22659758887630	-1.00530806095531
C	4.50722408649747	0.53577223719790	0.44718540305243
C	3.19485146592403	0.01252072970626	0.31966366130230
C	2.07809906119545	0.54861965901216	0.85453893049867
F	2.17782692620900	1.69356695320757	1.57748693219084
C	5.37641843355810	-2.51138531087845	-0.27087786395256
C	4.99242336075018	-1.07151422521994	-2.31364341731521
C	7.10809650591961	1.72237325310265	0.86154845441418
C	0.75866107292071	0.02332030896206	0.74404273066471
C	-0.33919039343201	0.59026997797243	1.26558386554532
C	-1.72360601607131	-0.00513604910039	1.19022917808900
C	-2.02691991102150	-0.67689732589534	-0.11305509214469
C	-2.31231899244435	-1.97951546739206	-0.28413474694943
C	-2.38751269136022	-3.09715316475908	0.76344487209915
C	-2.75441819824041	-4.28948883616962	-0.09168027580444
C	-2.86767828230517	-3.89143305297210	-1.42054690077630
N	-2.60051717235913	-2.53203117261251	-1.51730003722485
C	-3.20245700619768	-4.79464290158205	-2.41546903645202
C	-3.41905005972844	-6.11838248717034	-2.04371391144991
C	-3.30381626861529	-6.52694679960587	-0.72413432987199
C	-2.96830474054096	-5.60211339396491	0.26320230696612
C	-1.03612333206623	-3.33722399718271	1.44096870871784
C	-3.49015580666753	-2.84729320756311	1.79597487226230
C	-2.61762171813557	-1.76847449689360	-2.72534579634813
C	-2.83724008614930	0.95282450082881	1.63616743364794
C	-3.30740581120552	2.10777801238026	0.98809825365487
C	-4.73574507766426	2.60155822686959	1.23542903857411
C	-4.75412754393477	3.90488873603093	0.48794032061387
C	-3.53805190520315	4.05946730876953	-0.15447413626295
N	-2.71570839755351	2.94748063439230	0.13349828450297
C	-5.73144200138111	4.86760854654320	0.35258912265633
C	-5.46112064792224	5.98515643746530	-0.43329203994163
C	-4.23491634261959	6.12465383646548	-1.06782008643864
C	-3.24327403674007	5.15684098110228	-0.93904870603089
C	-5.71644657767918	1.60673234069256	0.59988806634178
C	-5.04400869070012	2.79126330221670	2.71559052159811
C	-1.41999256265778	2.84724071618696	-0.49631042854951
N	-1.63505258306217	1.23400029218603	4.04311081076403
C	-1.54670819279844	2.62739221596932	4.55129010916996
C	-0.15164830675615	2.95508989892011	5.06034350201039
C	-1.96861044087583	0.05260237582029	4.87975109967440
C	-1.07930054188291	-0.10632763200966	6.10151231910965
C	-3.44364895867791	-0.05284128078726	5.22283781062557
C	-2.61523056598638	3.00207677348306	5.55760886729256
H	-0.21945153740337	1.54138620475377	1.77690822425121
H	3.02819432666759	-0.89602861966567	-0.24323207621078
H	0.66418664067748	-0.90004975839742	0.17982754129593
H	-1.75972897106013	-0.78140996895267	1.96002720096597
H	-2.02031246221991	-0.03448119070544	-0.98825688162331
H	-2.85335915173328	-2.41665619971111	-3.56554874183976

H	-3.37156424743962	-0.97441131797504	-2.67936690383362
H	-1.64218993293474	-1.30564043245653	-2.91075160674707
H	-1.10711110367573	-4.19106616382594	2.11852606220265
H	-0.27000662476926	-3.56312245785738	0.69770816905267
H	-0.70175277465518	-2.47301433050743	2.01830443044824
H	-4.44173689422498	-2.64109580473115	1.30347002543490
H	-3.61819185257505	-3.73405594734375	2.42036358948999
H	-3.25325225513779	-2.00877793357303	2.45403300490494
H	-3.29860814979298	-4.49892698538177	-3.45258253756527
H	-3.68316065836603	-6.84109096785640	-2.80661253143511
H	-3.47551047586926	-7.56294033550263	-0.46084332324538
H	-2.87800951988707	-5.91976158422961	1.29681392109182
H	6.75488704318524	1.57141757996905	1.88566038540399
H	8.17440444125718	1.92957465063389	0.89236575642433
H	6.59550017151305	2.59334733735207	0.44163199764937
H	7.57077779581819	-2.92142067754473	-2.56077282325929
H	10.01106364383828	-2.52413144923606	-2.73044623548966
H	11.04165116047520	-0.62122837257171	-1.55610294293495
H	9.68195308332483	0.91821904791085	-0.19962185112422
H	5.59776481618158	-3.37643171576994	-0.89936745206742
H	5.93861474007711	-2.61427170733488	0.65829088560645
H	4.31320965352462	-2.52994532526289	-0.02968810565291
H	3.91549800610116	-1.04478990148449	-2.14490965710773
H	5.27923525340129	-0.15374781696145	-2.82901301940862
H	5.21343062322135	-1.91331709809241	-2.97295709681471
H	4.60988639722921	1.43584063770201	1.04069702057249
H	-2.36303991281684	1.24695235730690	2.88653359558974
H	-3.70184244798503	0.37356463562288	1.96481668013500
H	-1.02765027753154	1.84045796940193	-0.41655157863866
H	-1.52357821792405	3.11086026818432	-1.54956640686180
H	-0.71110967913865	3.53520992117139	-0.02868630979637
H	-6.73034368552946	2.00390804194555	0.66989972236388
H	-5.48627370413042	1.44418239803659	-0.45427027735127
H	-5.68746100152102	0.64373059604806	1.11314602597894
H	-4.96044842986010	1.84952070023062	3.25924920376571
H	-4.37055446413153	3.52219178093844	3.16469285446667
H	-6.06560360212661	3.15574046968831	2.83501117451069
H	-6.69248218305025	4.76420971030775	0.84366370906878
H	-6.21492144927973	6.75364056239637	-0.55066999801253
H	-4.04170880019203	7.00170765870683	-1.67314679923104
H	-2.28678080081427	5.27698246596220	-1.43095614330689
H	-0.72697209801161	1.03012562913742	3.63120137218028
H	-1.71649886974033	3.23709600625246	3.65255527418527
H	-0.06970536642924	4.02742721646370	5.24718367368245
H	0.07228898776630	2.43598605631950	5.99307190165479
H	0.61362026846533	2.68954378535707	4.32568057176918
H	-2.55305305271519	4.07539036007786	5.74404254517791
H	-3.61915612121247	2.78501248986163	5.19696090237531
H	-2.47070682806031	2.49824054614212	6.51373507255288
H	-1.74296333827316	-0.78305988945914	4.20714928763355

H	-1.26728901041853	-1.07861866462551	6.56095334364872
H	-0.02103772268206	-0.06348664116680	5.83677562163979
H	-1.27943018563373	0.65557731428457	6.85582273639204
H	-4.07174207807789	0.09751425079631	4.34389936148671
H	-3.64490478522005	-1.05587992996926	5.60252792510270
H	-3.74733474905542	0.65492371352763	5.99225969209669

B2

91

C	-3.45807770046952	6.40974875305569	0.36735353681646
C	-2.58697154663576	5.34538629605302	0.33697981090572
C	-3.06002873049753	4.05596255954521	0.09702398894525
C	-4.40770736542278	3.81310078560646	-0.11856356235526
C	-5.27746639278547	4.89894623243059	-0.08304008748421
C	-4.81848853898406	6.18503008911269	0.15564829619961
C	-1.08810247964718	5.30225870221821	0.53198092927853
C	-0.80017031909616	3.81160896052918	0.36046416066919
N	-1.99841068450856	3.16751295567761	0.12036208265926
C	0.38238790605029	3.15529685605891	0.41038682089908
C	1.66354243628471	3.73312395499443	0.66710249142302
C	2.82502274859725	3.05341372686956	0.68588209518628
F	2.80843067188617	1.72677013952538	0.43831740848307
C	-2.09884044274825	1.75395803738414	-0.07698213776357
C	-0.72617871169827	5.79607824399920	1.93537488032853
C	-0.39088079369651	6.13953708726313	-0.54325692623576
C	4.12065165774834	3.60648944078504	0.95182716387296
C	5.27158941679971	2.92601347303950	0.96385344799713
C	6.61019552667839	3.54547628408815	1.24582992431042
C	7.40302716747491	3.62957289751468	-0.03137760577500
C	8.03062046348693	4.70973228545291	-0.51304005738016
C	8.15532158292415	6.10987892496144	0.09549155954018
C	9.05073057972245	6.79932858946380	-0.91066290223931
C	9.35232679234693	5.91605414558819	-1.94780109862163
N	8.72974558165238	4.70572121623558	-1.71702349805212
C	10.16553438500571	6.30192353058181	-3.00307331441704
C	10.66596800821161	7.60084332918316	-2.99942695249181
C	10.36953094122674	8.48714810723595	-1.97607764223117
C	9.55282836539588	8.07967350894601	-0.92033747913292
C	6.80453095369807	6.82829509792578	0.16042802031404
C	8.82933376490619	6.07424764692644	1.46874480030599
C	8.82382300720085	3.55101206214447	-2.55139821308608
C	7.39211385248146	2.76745365510223	2.27376414420251
C	7.62773543588454	3.06401747375470	3.55597552923672
N	7.07622378608496	4.08867684739794	4.32380166901919
C	7.69204087666459	4.15951434653049	5.56278605710187
C	8.63812467135237	3.14325530101848	5.68770160512058
C	8.64326315187063	2.31468542806454	4.42628746606869
C	9.36578370366433	3.01291499936506	6.84785736793926
C	9.14835265583063	3.90953783107331	7.89534026050184
C	8.20510643603672	4.91577428771052	7.76019505656089

C	7.46164390630236	5.05981661710211	6.59141216181511
C	5.84315419148297	4.77127004188210	4.04713243501937
C	8.16553190836570	0.88931206739189	4.71013873496054
C	10.02460156457543	2.29557050054661	3.77748709680079
H	5.26272294752548	1.85997729402559	0.75353187881759
H	1.75298696489284	4.79167371557956	0.87037500909423
H	4.12347750509252	4.67523444949432	1.15143188923389
H	6.43986679877978	4.55380459895314	1.61515880668791
H	7.45941163392294	2.70363069583527	-0.59590142516060
H	9.32730035471032	3.80915301719550	-3.48050873382131
H	9.38531838754000	2.74566969206058	-2.06237350570248
H	7.82604071644005	3.17147228533191	-2.79429723024195
H	6.95059569958893	7.86290597177612	0.48017268734563
H	6.32486903575933	6.83733739177873	-0.81948422594492
H	6.12512432619320	6.34909758552330	0.86649930395727
H	9.80268440750576	5.58555597033345	1.40383899159942
H	8.98350818788048	7.09281006339455	1.83367989568192
H	8.23416340208500	5.53239211894398	2.20427787085914
H	10.41491694630301	5.62414159810767	-3.80998483818420
H	11.30364054933390	7.91967137456816	-3.81608878927578
H	10.77291491106298	9.49199674233272	-1.99417264273415
H	9.32102009346127	8.76899485773579	-0.11482182274244
H	-1.68520883730939	1.21341141910830	0.78025708064802
H	-3.14346960949388	1.47425215399925	-0.18949450777341
H	-1.55469299353375	1.44033580406352	-0.97417725028861
H	-3.09506443270427	7.41520718439705	0.55354837708646
H	-5.51497033480303	7.01400441658451	0.17724089248333
H	-6.33531418635725	4.72951456970983	-0.24745649881571
H	-4.78805035223344	2.81768330296473	-0.31070241886973
H	-1.04676472549726	6.83354722719679	2.05588454397875
H	-1.22603980948552	5.19520402636293	2.69657266923129
H	0.34804572361566	5.74812779360449	2.11575119221810
H	0.69442566179587	6.08632817714385	-0.45346346420402
H	-0.66590126545538	5.79200972620964	-1.54020342486483
H	-0.69186529169409	7.18584733306922	-0.45183951943198
H	0.37631567350472	2.08520763131793	0.24082037399504
H	7.90841739162337	1.89334742085409	1.88880136041455
H	5.21298250303670	4.14702868104185	3.41461478143245
H	5.31315314869479	4.94693531821823	4.98608486113163
H	5.99338218957163	5.74016445215311	3.55672096031082
H	8.85294332136561	0.38701337653547	5.39551725080925
H	7.17315276968606	0.89638289861831	5.16403407221765
H	8.11541293612653	0.31170078863706	3.78422408043501
H	9.99982267238814	1.75119391836746	2.83113388946719
H	10.37356672361226	3.30997787746871	3.57779710663046
H	10.74685248641642	1.80594265004588	4.43551398686896
H	10.09929392669601	2.22014843033328	6.95258884268114
H	9.71740516581344	3.81984794625014	8.81249196688966
H	8.04356596693455	5.61136300819516	8.57575856968904
H	6.73931731790175	5.86093772724100	6.49358758094390

TS-C1

92

C	7.62209039484348	1.60094657429298	1.16131147237493
C	6.71668664073918	1.25713102598484	0.18202720842439
C	7.10226454857463	1.25021633235261	-1.15302157201792
C	8.38654351028889	1.58350552496286	-1.54463392415478
C	9.29158399473569	1.92971205929566	-0.54675502459908
C	8.92087930068396	1.94105633717216	0.78984135183400
N	6.00959310503868	0.87413386385236	-1.93752232258056
C	4.90138000145844	0.64695455364843	-1.18219956789473
C	5.26437868016801	0.85597035072349	0.28654568161266
C	3.69771583236642	0.29667966130492	-1.73238387125500
C	2.51008745073154	0.02882454095674	-1.02177735181614
C	1.31509747135220	-0.30708497218188	-1.59001082727979
C	0.15723344932576	-0.55332585803133	-0.81394089035746
C	-1.08357265783084	-0.81873248702789	-1.27810476972231
F	-1.28328284574188	-0.82266248783679	-2.61614651458828
C	4.45168946706061	1.98783958441922	0.92041799856557
C	5.13636828197415	-0.44129255707065	1.09141259283489
C	6.03916464107130	0.75144985916672	-3.36854050709184
C	-2.27012161987160	-1.01171764746165	-0.48784146110972
C	-3.35150096001938	-1.75315126758033	-1.07701874763219
C	-4.24412995032371	-2.56246426928330	-0.44137512898712
C	-4.32803032105871	-2.96021287091788	1.03199130927501
C	-5.49128582627602	-3.92266173401989	1.02135573006695
C	-5.97839210721359	-4.04136336236768	-0.27439705683970
N	-5.23308720869283	-3.21419167840776	-1.11701331099455
C	-7.04336074634224	-4.86978818260252	-0.57964918138204
C	-7.62354734670803	-5.58032716342141	0.46630185141302
C	-7.15141947676371	-5.46621203552590	1.76543247553530
C	-6.07372950746283	-4.63015068749382	2.04922446412123
C	-3.05642966200855	-3.67836232637756	1.49291522831876
C	-4.64317720784477	-1.76232242826601	1.92820748647878
C	-5.44990247623190	-3.08606685300031	-2.53056860754195
H	2.53410565740219	0.09162776429314	0.06138299417650
H	1.23890995239305	-0.39415805948725	-2.66962780954668
H	0.25884291591807	-0.52399984733507	0.26668348690587
H	-2.04508173996410	-1.20254889863106	0.55262506014566
H	-3.43970722705929	-1.66377159739438	-2.15216207597664
H	-6.36327660051669	-3.60658959692857	-2.80513235062787
H	-5.55802070820955	-2.03398144993301	-2.80585701737301
H	-4.61820523172612	-3.51372726430668	-3.09848853343408
H	-3.19616322986091	-4.05304815031035	2.50871238564096
H	-2.83421022927649	-4.52795053391257	0.84625768478893
H	-2.18975293743681	-3.01604326524447	1.49337397083973
H	-5.53548115744268	-1.23726028492721	1.58347225289472
H	-4.82354645743436	-2.10159223062688	2.94986957498999
H	-3.80983231720135	-1.06092167453175	1.95244746669210
H	-7.42342634188712	-4.97649133439360	-1.58750926813841

H	-8.45943784719319	-6.23651526104231	0.25629739998279
H	-7.62029761201924	-6.03162147375415	2.56080628780390
H	-5.70314566377989	-4.54527648411766	3.06509894459647
H	5.38988725357144	1.49512028904556	-3.83964491373575
H	7.05460301351842	0.90383017615701	-3.72329345817713
H	5.71206699940687	-0.24531553511234	-3.67475497383853
H	7.33480798098454	1.60972995454000	2.20715701881059
H	9.64470339818614	2.21504209424947	1.54706091101045
H	10.30446313446129	2.19552515051218	-0.82400938848702
H	8.69387417410023	1.58309952452803	-2.58254253511594
H	4.81672350758237	2.17714925119299	1.93198461519184
H	4.55968817913602	2.90831657002770	0.34493334390474
H	3.39031710772119	1.74859632203606	0.98158939089313
H	4.10042402408533	-0.77347223829047	1.16504055023719
H	5.72002717619233	-1.24142128977716	0.63403492121813
H	5.51608324299760	-0.28098981740648	2.10242240542837
H	3.64309027685946	0.21109436535884	-2.81263956889614
C	-3.02260064057890	0.83547682464130	-0.24866300148049
H	-3.81843791041060	0.61159041990724	0.44903478611266
C	-2.03159042598554	1.70056007080980	0.21781915586978
H	-3.36337118565032	0.95559022740548	-1.26905786857038
C	-1.75444665302041	2.03862174411229	1.67479373358085
C	-0.50053734947056	2.86542143175427	1.56168792981945
C	-0.16202356930780	2.98302038231832	0.22126546931892
N	-1.10016043187256	2.27625972103164	-0.54892293758930
C	-1.04348044837829	2.30317569827440	-1.98960934960948
C	0.93510558392417	3.69954028782032	-0.21514645704572
C	0.27915844755775	3.47671841856649	2.51967528779620
C	-2.91999262312568	2.86675579666896	2.23076752414964
H	-0.07754628891629	1.91585143614630	-2.31992289265021
H	-1.82569232434281	1.68255179202835	-2.41276837464868
H	-1.16345607356753	3.33060829984548	-2.34068222572706
H	-2.69043515963104	3.18371197518755	3.24964720774915
H	-3.09541058276040	3.75824912101816	1.62712260479352
H	-3.83688479777335	2.27468299962252	2.25367820144602
C	-1.53064395129054	0.80113903413266	2.54276107228235
H	-2.43382812449156	0.19384828250156	2.60628796439703
H	-0.71202952043351	0.18546362482529	2.16868065051701
H	-1.27415333465741	1.11371317045646	3.55636959131793
C	1.39165685468684	4.20753984161711	2.10725944094582
C	1.70932397928341	4.31844353206807	0.76035710326378
H	0.03559943530630	3.40118811726370	3.57344102147918
H	2.01312128108100	4.69942362415100	2.84518287789637
H	2.57607058093371	4.89464652213835	0.46117308080609
H	1.18979168936113	3.78064402995123	-1.26386858203579

C1

92

C	8.52814870694534	-1.53408747100013	1.17176972363387
C	7.52804394011979	-0.93277252230295	0.44122727985700

C	7.84353909310424	-0.13447064667586	-0.65313795068827
C	9.15439857584867	0.08015536835076	-1.04178169679597
C	10.15613179387615	-0.53213753618248	-0.29504960013437
C	9.85530978600808	-1.32940089777221	0.79898992820541
N	6.66532882839115	0.35525965607723	-1.21274809988896
C	5.56231188630722	-0.07766030310280	-0.53000525510807
C	6.02770585704180	-0.97868311585898	0.61279449482042
C	4.29652574709628	0.28694050023044	-0.87698760961857
C	3.09866794319813	-0.12354257523660	-0.23585675015395
C	1.85248897943507	0.27952760164381	-0.58659441022067
C	0.67975091602442	-0.18063204131971	0.08772106377016
C	-0.57989779174059	0.18721067840432	-0.16084387324172
F	-0.83381404468411	1.10928655132182	-1.13083877743146
C	5.63604149848363	-0.41297730995276	1.98012588605404
C	5.52726738047244	-2.41610230912043	0.43860316487542
C	6.59823693912384	1.21050936711200	-2.36175459132722
C	-1.81987245148484	-0.26526340025481	0.51118906890858
C	-2.80901551208766	-0.78751595901933	-0.47879658904805
C	-3.51500963828091	-1.93095067969805	-0.37509347465924
C	-3.54870114055892	-2.95489080769996	0.76363037669346
C	-4.65461420308694	-3.88013399043836	0.30544336634794
C	-5.07595861248602	-3.49975243764173	-0.96430588939207
N	-4.35683397989309	-2.37920048779170	-1.36968219264371
C	-6.06986114944617	-4.18936915488126	-1.63580363193321
C	-6.63568213728368	-5.28957016332151	-0.99766987970145
C	-6.22210925500542	-5.68172026721661	0.26573621369883
C	-5.22091510542232	-4.97091750326695	0.92594026753260
C	-2.22774799128003	-3.73454958324467	0.81432397717279
C	-3.87511222318485	-2.34741204476633	2.12537536862022
C	-4.49558726952369	-1.73620543971369	-2.64019728038286
H	3.17477457779353	-0.81508881329131	0.59749573705931
H	1.72570634704504	0.97041966448809	-1.41459992306675
H	0.80736804583064	-0.90796202343821	0.88472543318127
H	-1.52562795381474	-1.05547347209428	1.19982117652824
H	-2.89630349041714	-0.21574903130193	-1.39512589211537
H	-5.02060297848049	-2.39674460972862	-3.32676427157995
H	-5.05796182187915	-0.79746289259103	-2.56928620455129
H	-3.51086801014353	-1.51901227070652	-3.06230364561115
H	-2.30024699842139	-4.53251328465259	1.55590681305898
H	-2.01147818028346	-4.18861540803669	-0.15341583758237
H	-1.38605571019365	-3.09407498027720	1.08446884124419
H	-4.78090421140158	-1.73913472016092	2.07920358102996
H	-4.05060008945295	-3.14479637124088	2.84990349309904
H	-3.05590553911039	-1.73853436111639	2.51174080036052
H	-6.41290255701180	-3.89273583842661	-2.61899637742566
H	-7.41694636286365	-5.84556106065279	-1.50195375500430
H	-6.67908150747033	-6.53990787480449	0.74194367087636
H	-4.89826236929959	-5.28071462008521	1.91431395475034
H	6.12782683745199	2.16578368220387	-2.10997647509512
H	7.60226755034113	1.40738311628163	-2.72777126717728

H	6.02238483917717	0.73927911506714	-3.16371517425587
H	8.29280336332532	-2.15941211203698	2.02633743804364
H	10.65349533495322	-1.79432551367818	1.36382712192668
H	11.19065651623640	-0.37891733001702	-0.57805900047748
H	9.41067429227483	0.69941523108272	-1.89196106676134
H	6.07189696740507	-1.02770404727889	2.77047227775017
H	6.00895612559517	0.60549063179777	2.09862000171338
H	4.55431963275040	-0.39849068681887	2.11660334024713
H	4.44227526548414	-2.48257445501062	0.52577752036496
H	5.81571943141425	-2.80935178394031	-0.53715640507346
H	5.97153784892722	-3.05389351503594	1.20562009421172
H	4.17907763337094	0.95415427713523	-1.72474335217721
C	-2.40073220127861	0.85402639050723	1.47733114503222
H	-1.69907707905658	0.95507284580787	2.30580638048299
C	-2.61314400430278	2.19577020678020	0.91883496885647
H	-3.33923012247793	0.46614121634830	1.87871513935833
C	-1.76700981752370	3.40270675482888	1.25491699298225
C	-2.42087059414536	4.46860923178270	0.42295978201673
C	-3.53690097037016	3.92584194579826	-0.19143086345896
N	-3.61760370331493	2.55460804939233	0.16373847455563
C	-4.75484756205454	1.75399246189538	-0.24254177829285
C	-4.38699306872688	4.64695994349843	-1.00452770143244
C	-2.11768890740622	5.79974356264345	0.22560821696994
C	-2.00107706621369	3.69750166330767	2.75436184700958
H	-4.78867939920144	1.70639876733261	-1.33081156454127
H	-4.65848837088116	0.74377160339582	0.14351074581946
H	-5.66539670437547	2.23119794923372	0.12433262219956
H	-1.50440666773494	4.63646078401908	3.00096443604020
H	-3.06214864687862	3.80170659683984	2.98759799450160
H	-1.57271146390601	2.91142228516731	3.37778590199132
C	-0.27220227209479	3.23782097239220	0.99247690176218
H	0.13815158005892	2.39350392995356	1.54739178162858
H	-0.06439069078074	3.08860422588409	-0.06452219508659
H	0.23911242535469	4.14112629216367	1.32820950795834
C	-2.95419167944427	6.55297278898619	-0.59255093576986
C	-4.07096520397229	5.98637892033265	-1.19627868715199
H	-1.25066922295501	6.25444216559041	0.69013459502925
H	-2.73182830024374	7.59889376731992	-0.76401217853816
H	-4.70456865443939	6.59554565746677	-1.82851673892541
H	-5.25467382679398	4.20583430403693	-1.47812406630927

TS-C2

114

C	-2.60629132399140	5.23796666842409	-0.56403987578755
C	-3.01892353538913	4.06091349186851	0.02870508740672
C	-4.35471886895661	3.74284175733526	0.20957593239814
C	-5.33328581255597	4.61910894380339	-0.20832068467929
C	-4.94501272559595	5.81419094398233	-0.80892900731411
C	-3.60141013544069	6.11556155331854	-0.98336357216431
C	-4.43561791566365	2.39024883171276	0.85668997166315

C	-2.96239264481103	2.06971594891566	1.12053130909483
N	-2.21906475622026	3.02085844123981	0.54950254530790
C	-2.60227896419921	0.94027097310127	1.88162681060576
C	-1.49842249786871	-0.10726113612298	1.58622117669626
C	-1.78129120479021	-0.76082485655205	0.27005268750242
C	-2.09192119339072	-2.05328837282129	0.07069031223083
C	-2.19073881764133	-3.18986915476828	1.09333588325403
C	-2.54519695721670	-4.36371987520855	0.20844120028630
C	-2.64543658276517	-3.93669709179308	-1.11221359134804
N	-2.37777867425551	-2.57396767028727	-1.17536183170626
C	-2.97019065946866	-4.81637073930548	-2.13074466164985
C	-3.19158159817177	-6.14802961806647	-1.79072732904417
C	-3.09020513083669	-6.58564480761378	-0.47941436939664
C	-2.76436831707214	-5.68353071391794	0.53203375328505
C	-0.85339095892259	-3.43659861184034	1.79420238765207
C	-3.31472760465418	-2.95322631968018	2.10590497082895
C	-2.36008437994107	-1.78889226253020	-2.36972650044394
C	-0.78042106358948	3.12492353152958	0.48838798600709
C	-4.95224828160026	1.34960663723022	-0.14984965405023
C	-5.30349487444923	2.39036464262357	2.10819972319781
C	-0.06959294608688	0.30709161246710	1.70603856939322
C	0.96410346992515	-0.02661065799290	0.92927415363545
C	2.33748313520181	0.31425169915515	1.13641626558750
H	2.59813267318016	0.89678979279940	2.01486390949452
C	3.31137258752907	-0.08852039590713	0.28463898894994
C	4.69698475800258	0.18499270742131	0.43472372200915
C	5.68984184135499	-0.23413882514095	-0.39716437947931
C	5.58986863832350	-1.07180612158043	-1.67182665697194
C	7.04145633742743	-1.17719423351564	-2.07781756905254
C	7.82900539054411	-0.48848188933259	-1.16132680589825
N	7.00797774095055	0.06373560144694	-0.18094344651551
C	9.20540619536900	-0.42311417884370	-1.29120774830654
C	9.78170397070089	-1.07298410754405	-2.37838296361298
C	9.00801213873347	-1.76241824230346	-3.29959917881666
C	7.62349480960357	-1.81632449182305	-3.14939608954420
C	5.01911074999497	-2.46613977920920	-1.39919653606060
C	4.79302173978344	-0.34667816570737	-2.76017286067532
C	7.46911138363411	0.84476907920912	0.92913606404491
N	-2.04024966348202	1.71634764499645	4.43296242100817
C	-2.03429777583370	0.50496716288162	5.29190493549730
C	-3.37228343108671	-0.21466569737235	5.32837261712785
C	-2.69020171024562	2.99764890589169	4.79822611417832
C	-4.02557846069936	2.85474967058876	5.49977454674637
C	-1.74869924065102	3.91038906158945	5.56902949109732
C	-1.46996922136448	0.73798239457402	6.68288913098222
F	0.18468450422987	1.04455095189172	2.83671972556775
H	2.99668274176012	-0.67360440913939	-0.57363849732589
H	0.71022344174823	-0.62253354001692	0.05915268034778
H	-1.65146047482431	-0.84658206090114	2.37979418869633
H	-1.75290095467157	-0.09582341708950	-0.58760409084833

H	-2.60326565146944	-2.41565345950259	-3.22392821475699
H	-3.09413514348914	-0.97719971731442	-2.31840613117083
H	-1.37127853471130	-1.34772775967141	-2.53705071735918
H	-0.93326316676106	-4.30605875123506	2.45041303977861
H	-0.06790730332436	-3.63633185832502	1.06413362783247
H	-0.54055116017463	-2.58219750826174	2.39666078435627
H	-4.25853713892216	-2.74925710987511	1.59734540446139
H	-3.44997748862358	-3.84537783268156	2.72084033125063
H	-3.09304050867985	-2.12034564110430	2.77550026357238
H	-3.05503017885223	-4.49757955470542	-3.16205202943595
H	-3.44772310139835	-6.85352869522280	-2.57221119409352
H	-3.26447003418364	-7.62730157767072	-0.24129523837919
H	-2.68407077184871	-6.02343771397083	1.55925213413917
H	7.22624308559902	0.35780734727995	1.87852927444060
H	8.54772357876752	0.96201357678433	0.86771754062282
H	7.01170078648693	1.83860154049256	0.92083625812128
H	7.01826441543134	-2.35599677060915	-3.87003594965731
H	9.48102494921307	-2.26024476031036	-4.13674257786496
H	10.85742964101550	-1.03746734395930	-2.50247840137247
H	9.82766789704355	0.10797525142227	-0.58218094112966
H	5.08125793423655	-3.07148916489577	-2.30592415533321
H	5.58688954468591	-2.97055675422673	-0.61605811266687
H	3.97463969005404	-2.42456376728319	-1.08948483906798
H	3.74637804355222	-0.22340337980489	-2.47971444882669
H	5.21374578600224	0.64133699259011	-2.95282963984513
H	4.83345429670463	-0.91976467761132	-3.68893706510931
H	4.97824397255342	0.77295244991044	1.30235408966031
H	-2.38994132067468	1.38273332813277	3.17092855473062
H	-3.51215446704725	0.36099975525918	2.04308752618590
H	-0.31942348359121	2.16677662071185	0.70296917415613
H	-0.48827349171106	3.42829600408561	-0.51748404281740
H	-0.42657111035059	3.87711717940977	1.19807439824793
H	-5.98044956999904	1.59167495617268	-0.42346871255883
H	-4.34893236052764	1.34792901877123	-1.05924659216840
H	-4.92891665909471	0.34696444226430	0.28106465573802
H	-5.28886193364377	1.41825917400297	2.60391132844128
H	-4.98224383643002	3.15608114240835	2.81402177954343
H	-6.33743895798473	2.60259590593366	1.83126990970550
H	-6.38457843009241	4.38974739285145	-0.07596312016273
H	-5.69858349231398	6.51593740893270	-1.14401935396885
H	-3.31872103304985	7.05054545330213	-1.45114334207895
H	-1.56044762789923	5.48337149945844	-0.69606140389705
H	-1.06571915701690	1.92536488718158	4.23739737959765
H	-2.88164061655573	3.47409670644008	3.82602994758850
H	-2.19111793466605	4.90361760445176	5.66510760766090
H	-1.55199788540461	3.53268630997355	6.57308834134853
H	-0.79237076732994	4.02405358725738	5.05175146716669
H	-4.48450826358285	3.84200709581923	5.57444651800383
H	-4.71383039571086	2.20809852489537	4.95804121854781
H	-3.91389871920696	2.47221033843220	6.51494371436071

H	-1.33310629049026	-0.14933692940700	4.76338030461682
H	-1.35057460409493	-0.22129624518742	7.19027601830094
H	-0.48935302282776	1.21645898710974	6.64382441315811
H	-2.13221339803328	1.35190051150222	7.29524395374579
H	-3.82072029202834	-0.28539917840637	4.33617150037298
H	-3.21489718375139	-1.23110541927729	5.69293949969268
H	-4.08690702339018	0.26323037120982	5.99621090348520

C2

91

C	6.71568082707352	-2.35715834415655	0.66338536026957
C	6.56135693789427	-0.98252604525876	0.50081418276630
C	7.18545369895676	-0.38652490621246	-0.58401455646902
C	7.94433400307953	-1.13137503160442	-1.48307100752533
C	8.09182985416430	-2.48795883584907	-1.30939707821868
C	7.46758672841716	-3.10843903852164	-0.22580046656773
N	7.18644141601438	0.95087365978978	-0.94697202247072
C	7.86237849367723	1.13873878407017	-2.14543775007310
C	8.51361969768284	-0.20368677912224	-2.52644606819274
C	7.97798826948216	2.21086128195276	-2.93945601854894
C	7.35631102147110	3.59309518857227	-3.00549200896467
C	6.02080738271220	3.83187959778758	-2.35401363926690
C	4.81611845399608	3.67296486540661	-2.91763710138996
C	4.44431898014539	3.14696688189202	-4.30683307511915
C	2.93710018854678	3.06515365115885	-4.20882088761945
C	2.52473968067552	3.58379431855513	-2.98182452693333
N	3.63005736480726	3.99201289252444	-2.25977800237920
C	1.18237983709635	3.63380083605693	-2.63857887374545
C	0.25606076680029	3.15880719845057	-3.56344349946670
C	0.65468104249856	2.64711027687569	-4.78795773869186
C	2.01125937192930	2.59978042588546	-5.11289362733976
C	4.82377570822493	4.16217875042430	-5.39185041928945
C	5.04756076129800	1.77737345931951	-4.60399500080640
C	3.58568571042176	4.55622786536105	-0.94870119098186
C	8.12759999582357	-0.65069285291174	-3.93242106867697
C	10.03566334597953	-0.11554846976354	-2.39278369844016
C	6.79190373614089	1.96602642411488	-0.01340356665651
C	8.33798177201666	4.63069709140515	-2.56286907829236
F	8.58231202313029	4.60472860840338	-1.23279054141649
C	8.97643817398437	5.51930481594521	-3.32720558700454
C	9.94204801836928	6.48257467621927	-2.88132027387948
C	10.56547507510045	7.34182595244279	-3.71393718590584
C	11.53964360681795	8.30550260851519	-3.30296488477211
C	12.21989395787766	9.15992021823388	-4.10195609893174
N	13.14489897386058	10.06480323240749	-3.61510010573033
C	13.72158381220008	10.80408594046377	-4.63388543551542
C	13.17201092760412	10.41370740505068	-5.85414407756305
C	12.14627283821213	9.32779697136426	-5.61943427848734
C	14.69252552655760	11.79136358163591	-4.56568584531266
C	15.10109719037750	12.38386433438210	-5.75694960337378

C	14.56073126610926	12.00421979332137	-6.97576668270200
C	13.58541028762435	11.00800910229810	-7.02400240504453
C	13.45761234306337	10.19227164911505	-2.22521501308442
C	12.55193577125549	8.05067494351611	-6.35884648116719
C	10.76314893049468	9.80952236399378	-6.06695488955105
H	10.30570480503850	7.28917350766499	-4.76647720657645
H	10.17676465424752	6.50236426652670	-1.82076143348159
H	8.73391173675065	5.49525188758675	-4.38497205049107
H	7.24176204188641	3.78169021856132	-4.07593289945004
H	6.05220647823740	4.22723294734216	-1.34789869057704
H	2.57848285646827	4.91165167135479	-0.73871205226428
H	3.86619103233847	3.83036904190175	-0.17549198422014
H	4.26820038730869	5.40808762395477	-0.88160280863701
H	4.43679311578263	3.83154014491740	-6.35853151464517
H	4.39567942190815	5.14106935452518	-5.17029294484725
H	5.90557081763953	4.27320675525046	-5.48105000683277
H	4.76965948634074	1.05735143195248	-3.83257550837729
H	4.67745869942851	1.40938812725778	-5.56420907819397
H	6.13583104632160	1.81546173251091	-4.64605765593532
H	0.85063966132931	4.01820635571914	-1.68208472474287
H	-0.79809894561807	3.18705693925865	-3.31240779272653
H	-0.08439844697355	2.27926425057549	-5.48885552076204
H	2.32759223552330	2.19566695744757	-6.06900245435392
H	13.88759377512644	9.26567853311513	-1.82997587528458
H	14.17812960416824	10.99440503334406	-2.08497206818964
H	12.55971227338030	10.42840632741851	-1.64515435759856
H	13.16049666911626	10.70775758398303	-7.97617041423531
H	14.89563060458702	12.47994247288112	-7.88910836988880
H	15.85954137318942	13.15751114581891	-5.72517576307334
H	15.13061775907791	12.10145453172788	-3.62532104838857
H	12.59157286066397	8.24296766586139	-7.43368185813686
H	13.53882891815241	7.71532820944345	-6.03626899093872
H	11.84438421727977	7.24031557544588	-6.18348636303850
H	9.99530528962784	9.06003391722379	-5.87382848369224
H	10.48596797668817	10.72411311120871	-5.54056100847212
H	10.77386842670144	10.02302280662151	-7.13839957298221
H	11.74825816183920	8.33610693014004	-2.23781382535431
H	8.64890160751803	2.04606960052145	-3.77795924610396
H	7.34771142353493	2.87885211939367	-0.21278463851309
H	5.72133014140148	2.18265334213298	-0.05821956670510
H	7.03755084145590	1.62998868777858	0.99817281822091
H	8.54744067458771	-1.63755481043865	-4.14205843204130
H	7.04335070439082	-0.70939800402155	-4.03862518303197
H	8.50910462026427	0.04414552906510	-4.68352502022991
H	10.44090730268428	0.60545688327577	-3.10656540893233
H	10.32009741213493	0.20063599640852	-1.38771060515683
H	10.49002529531715	-1.09053434668560	-2.58717793730076
H	8.68661525581585	-3.07276851819533	-2.00339716853331
H	7.56886712716169	-4.17706187472919	-0.08130634699231
H	6.23096396197572	-2.84587784828897	1.50083777770871

H	5.95919886853207	-0.40939615827127	1.19488110253358

TS-E1

86

C	-7.17887418670247	-2.42579008207950	-1.15795598249294
C	-6.38069827502306	-1.68755825014884	-0.31240813909268
C	-6.85749135311392	-1.29414021487526	0.93148654532728
C	-8.13136208838083	-1.61788937418442	1.36164270907354
C	-8.92874941013531	-2.36318384999401	0.49916869701448
C	-8.46509663165287	-2.76504855162674	-0.74469986978596
C	-4.96785367471959	-1.18334454692262	-0.48933139195444
C	-4.74236390993421	-0.44187705656178	0.82790135229555
N	-5.86133175977704	-0.56687495658785	1.58908494091368
C	-3.63954699557619	0.24737736575925	1.25202173256067
C	-2.42931259609399	0.39181116604495	0.53608948286692
C	-1.31840812990037	1.02061951213188	0.99818187518950
C	-0.07883789721346	1.05858715234553	0.24747486457319
N	-0.07627559400191	2.66239887607212	-0.67850895976072
C	0.87438757211966	3.64926382796057	-0.09445358604504
C	1.23648399860479	4.79324596603431	-1.02762511467184
C	-5.99476327528488	-0.01883457149414	2.91059757667974
C	-4.89339215818461	-0.24341847383206	-1.69565212239509
C	-4.00772637928782	-2.36747977225801	-0.63981576438065
C	1.15288857020690	0.91899216112718	0.98721992463966
F	1.14084764441004	1.43326763398418	2.24000670924413
C	2.25429492886871	0.25387165062404	0.57493372768082
C	3.40750465116601	0.02270760246368	1.35869924093435
C	4.50074653111249	-0.69827633225059	0.96636147560598
N	5.56114057623471	-0.91662138893188	1.78822312843636
C	6.55875388531512	-1.66515549460570	1.15829491116917
C	6.14596170525525	-1.96918887754703	-0.13307075050324
C	4.77798414283202	-1.37563571703971	-0.37441480616582
C	7.78043273606279	-2.08528195007666	1.65220599365650
C	8.59092122800174	-2.83475217144957	0.80565451590265
C	8.19067547461052	-3.14776748368662	-0.48483375308352
C	6.95653399659710	-2.71242095284371	-0.96216752426617
C	5.63131836752088	-0.43877123257697	3.14132471974366
C	3.76862535181594	-2.48941470106528	-0.66880671651161
C	4.84086887619418	-0.35952607657206	-1.51851467575648
C	-0.00427682876685	2.45829321684397	-2.14847325915200
C	-1.24059744203935	1.73745126046313	-2.65412124541599
C	1.27672071361596	1.76435530390042	-2.57194861766021
C	0.27447213495028	4.19711034610889	1.18768211267924
H	-2.37114726670669	-0.05784253049099	-0.44984315851972
H	-1.31720561535925	1.45056582372955	1.99357655592504
H	-0.08013076279466	0.41417155144234	-0.62385525727534
H	2.20459176923145	-0.13884546963514	-0.43121937572086
H	3.40069158364081	0.43707554654951	2.35885957518819
H	6.53231881752465	-0.82022044749347	3.61350785693423
H	5.65683226113526	0.65424418864042	3.17072316457447

H	4.76735489436379	-0.78548460296991	3.71393421175520
H	4.07223457194209	-3.03027609885427	-1.56723409118702
H	3.72644544522213	-3.20147975676055	0.15633524792669
H	2.76397800663084	-2.09788982101147	-0.83185807868239
H	5.55538036796908	0.43391501006886	-1.29440518607797
H	5.16504165901811	-0.85798925300926	-2.43410560917431
H	3.86956447428421	0.09920924411711	-1.70876852480092
H	8.11160942459288	-1.84930944565924	2.65539011758963
H	9.55290426541424	-3.17821840746658	1.16627441893992
H	8.84046702894625	-3.73279143164850	-1.12333450536179
H	6.64510137865672	-2.95914898938885	-1.97140573785892
H	-5.98137941852744	1.07455660681499	2.88696821219409
H	-6.93629949226232	-0.34554090513832	3.34301930958656
H	-5.18189129271195	-0.36768994921304	3.55228562063833
H	-6.81854806050703	-2.74113780645271	-2.13115552181801
H	-9.10644901896527	-3.34394375615477	-1.39718480989501
H	-9.93135505644910	-2.63152887342606	0.80931899826812
H	-8.51300251445589	-1.31039431661193	2.32677000495900
H	-5.19536421088272	-0.78025124595157	-2.59695487703241
H	-5.56542832280483	0.60627338067899	-1.56717664167821
H	-3.88379040331900	0.13823866531244	-1.85202147241815
H	-2.97144802169255	-2.04222493138318	-0.73891919148679
H	-4.07352671705354	-3.03257149161946	0.22242164625104
H	-4.27158870198586	-2.94184023141749	-1.53008071403025
H	-3.67728301881646	0.70923084955381	2.23271131801136
H	1.78053419603077	3.08065243838909	0.13881794738929
H	-0.01300516328106	3.45732496776920	-2.59194828149295
H	1.89598116131239	5.47365895499747	-0.48700332721749
H	1.76833081830773	4.47172742649422	-1.92272697462197
H	0.35428522183986	5.36494520102079	-1.32775328149543
H	1.00229367884200	4.83433518872162	1.69026947946064
H	-0.60501789635752	4.80920614999498	0.96514899106500
H	-0.01050085861585	3.41152427987801	1.88353233507575
H	-1.24700819976522	1.76525752317207	-3.74453150315890
H	-1.26317693568249	0.68795376680509	-2.35642868463733
H	-2.15930971899254	2.21509398075455	-2.30500742061417
H	1.38033869337517	1.84072246886283	-3.65512183003082
H	2.16222407762252	2.21153790033538	-2.11918295111568
H	1.26395353903692	0.70203821621988	-2.32272531612260
H	-1.02320416665696	2.96205746878050	-0.45536664529983

E1

86

C	-6.74586050026118	-2.39494608648060	-1.53623588325319
C	-6.04327715589799	-1.63175121127943	-0.63031128053151
C	-6.58519086780899	-1.35405514933718	0.61877845444591
C	-7.83125441108234	-1.82345183688121	0.99329564781004
C	-8.53180007172006	-2.59332468370819	0.07008583297250
C	-8.00272564726284	-2.87876773165949	-1.17975708492742
C	-4.68280717030959	-0.98420089821910	-0.74180621736799

C	-4.55552555148484	-0.30657009242201	0.62345541074256
N	-5.68293590390040	-0.57052889813411	1.34136195481608
C	-3.52731493722011	0.44092370457123	1.11837101235388
C	-2.32466649894997	0.74348764235478	0.42820803540699
C	-1.28645134136812	1.45593104547659	0.91736585866838
C	-0.05198901912785	1.66962794182908	0.12781584283573
N	-0.10749197395800	3.14497462373165	-0.53837767260167
C	0.75666891564109	4.17030457655112	0.16039283737748
C	1.02978503875536	5.40547557431049	-0.67761456447057
C	-5.89713944571448	-0.10165339552179	2.68120131171272
C	-4.68378154594568	0.03175744974330	-1.88736764814182
C	-3.60849829251349	-2.05672839843678	-0.94389718973795
C	1.20397108994661	1.39822522351819	0.84831370781559
F	1.25593218488419	1.89443270862251	2.10940558481993
C	2.20783540380511	0.61868667353198	0.41538361702871
C	3.35520423627855	0.25555632237636	1.17091505102600
C	4.35458377686871	-0.56430485655313	0.74087022556695
N	5.42207883055345	-0.88907650388528	1.52468938494943
C	6.30381778678738	-1.74736469252245	0.86681549695518
C	5.81587630262278	-2.00700607216739	-0.40871262596096
C	4.51496228465111	-1.26456054311374	-0.60833531913222
C	7.48690700131284	-2.30246874541513	1.32055087330766
C	8.17982218643139	-3.13891083353270	0.45101563109856
C	7.70467298172272	-3.40646766819491	-0.82407160264672
C	6.51077791302880	-2.83621102330993	-1.26083121834465
C	5.59288094522580	-0.40826911935004	2.86607816616841
C	3.38112558622756	-2.25788061887649	-0.87676214784459
C	4.65783487415113	-0.25596474048823	-1.75193274039649
C	0.02821024528287	3.09786657703511	-2.03751715985583
C	-1.14888076268647	2.37240713485930	-2.66163323986710
C	1.36256930800160	2.53357940880791	-2.47759793730921
C	0.08017607094263	4.56792679850989	1.45984910299796
H	-2.22103068435599	0.35640285509843	-0.58081878955133
H	-1.30844526252391	1.83134551219678	1.93430363814497
H	-0.06409805507628	1.02160104187703	-0.74408908003894
H	2.09555147411194	0.23802712385223	-0.59045533029056
H	3.41689072007089	0.65601880387246	2.17478152511687
H	6.51867244157285	-0.80045321593218	3.27779557254395
H	5.64112809888809	0.68426330911243	2.88349489560243
H	4.76463559490247	-0.73142050654103	3.50341617769080
H	3.59968433748433	-2.82776374196312	-1.78204976024207
H	3.27985329910662	-2.96193558709596	-0.04989012437958
H	2.42151915434908	-1.75784650675055	-1.01553658589002
H	5.47329860205374	0.44094688386239	-1.55346026384244
H	4.88060078524898	-0.78295981370975	-2.68188436407824
H	3.74420039232813	0.32203302055899	-1.89970916371571
H	7.87419202424706	-2.10639945532531	2.31216377236252
H	9.10857378518252	-3.58804912176051	0.78161174921979
H	8.26266578312014	-4.06140398270555	-1.48124754325088
H	6.13952857549915	-3.04785136460995	-2.25784782065885

H	-5.89296911055609	0.99148142294102	2.71655150820070
H	-6.86030557437823	-0.45391609711785	3.03982814299286
H	-5.11937698082405	-0.47735695495327	3.35201222151083
H	-6.33333609006052	-2.62044772033702	-2.51379813432808
H	-8.56813103557833	-3.48059308211169	-1.87998251408365
H	-9.50974016930880	-2.97505091462571	0.33739905950164
H	-8.26170329628852	-1.61025364413722	1.96328751943278
H	-4.90289687655792	-0.47788147045869	-2.82777663990705
H	-5.44834070977762	0.79402771914091	-1.73007126644943
H	-3.71940692436266	0.53090853028402	-1.99088947588185
H	-2.60855457701964	-1.62597938052483	-1.00782807604536
H	-3.61751587920006	-2.77208196571120	-0.12056350927413
H	-3.80471307172884	-2.60281245511506	-1.86879207555904
H	-3.62001739167292	0.83105371994173	2.12610424784661
H	1.69713383928872	3.65511752348453	0.36590709037786
H	-0.03530138230468	4.14026263530713	-2.34854705695391
H	1.60105801399411	6.09983515830590	-0.06010292346972
H	1.62147799910640	5.20863409962249	-1.57086822759702
H	0.10731043264620	5.91675721153880	-0.96548066887634
H	0.73938007417996	5.23579325823876	2.01448808074772
H	-0.84778101180014	5.11172388155120	1.25829338096185
H	-0.13677808891479	3.71744034018272	2.09950534203518
H	-1.11238597903564	2.52000295843652	-3.74180149450603
H	-1.12685846365104	1.29671813574294	-2.48179413738134
H	-2.10515230678325	2.76363852665111	-2.30627642224532
H	1.49634385911252	2.74220275870390	-3.53979888146871
H	2.20262978661217	2.97111643924375	-1.93796956229498
H	1.40548945344943	1.45149491583748	-2.35108981657001
H	-1.07617347070477	3.41280858956053	-0.35579572394540

E2

85

C	-8.03895350771251	0.25749125497740	-1.45242397666606
C	-6.94658166887765	0.52516115757156	-0.66024945849202
C	-7.10191043145957	1.20442266425758	0.54758956714005
C	-8.35024459328639	1.62567277121000	0.97903701621801
C	-9.44639945265089	1.34774705219433	0.16753631433943
C	-9.30366437533828	0.67229181021999	-1.03438663767254
N	-5.86710526626402	1.34566447922760	1.15579039357155
C	-4.85897206837987	0.76840214367641	0.40419018229984
C	-5.48575826441460	0.19237916567225	-0.86542656951482
C	-3.56877590616840	0.75628759227661	0.80147283745500
C	-2.46528488866673	0.19139287221924	0.07576218836185
C	-1.20217198329371	0.15501042381490	0.50656958485791
F	-0.91918877180207	0.70295144808748	1.71055571431486
C	-4.95883081604445	0.89067756177949	-2.12219631427938
C	-5.30015994464992	-1.32438671161629	-0.95610502388245
C	-5.62982082465817	1.99022502201546	2.41018444276522
C	-0.01561000308036	-0.40309714960198	-0.23268362865282
N	0.38718267074262	-1.77361022822541	0.11593735759371

C	0.11659911568769	-2.74262397243389	-0.94377803971969
C	-1.35951188431700	-2.93927484941816	-1.29375002502642
C	1.12075701713863	0.58587709380264	-0.23718746389055
C	2.41404414585692	0.26685917572055	-0.14723075641017
C	3.48980746287574	1.21871173630875	-0.22954463059819
C	4.81048433590963	0.94639762231726	-0.17273243249844
N	5.78574187687503	1.92603407071670	-0.26991165423717
C	7.05811446690775	1.38969578825260	-0.18851528138826
C	6.96706976723755	0.00709517726864	-0.02991025340426
C	5.51175056372454	-0.40169561223227	-0.00469082602648
C	8.28925247573492	2.02556134895576	-0.24359108115297
C	9.43324137352462	1.23988802977670	-0.13585438287207
C	9.35406783252471	-0.13483099535189	0.02201267197991
C	8.10615847393253	-0.75680091352128	0.07485398706353
C	5.48358587122079	3.31256724988618	-0.44258142959570
C	5.16832058325888	-1.05526628459854	1.33603511371339
C	5.20998457363243	-1.34685866658677	-1.17042842223262
C	0.08198924646521	-2.17757098406684	1.49198864283282
C	1.01929931873729	-1.48020750412696	2.47095839536118
C	0.18717046626912	-3.67680411418011	1.72198905571159
C	0.93302057968549	-2.43373762666638	-2.19016823687253
H	2.64033941329109	-0.78267790221845	-0.00199675456940
H	0.82837011303603	1.62420550088828	-0.38254966297394
H	-0.38037609956551	-0.44167562147645	-1.26577006951436
H	-2.63450931763557	-0.24839976406392	-0.89641401102608
H	-3.32302819083877	1.20928572152587	1.75443511187500
H	-6.56242957968561	2.39438479556964	2.79643947701220
H	-5.22449002503965	1.28628568354100	3.14494659091071
H	-4.91586642366513	2.81233541133814	2.29549260808034
H	-5.49763020406546	0.52932105662846	-3.00125528827120
H	-5.10577603908398	1.96956823964943	-2.05268758793904
H	-3.89535972779173	0.70198371961922	-2.27164695421222
H	-5.68574059903407	-1.81232316024364	-0.05955864205490
H	-5.84865617268206	-1.71300794496822	-1.81740289471670
H	-4.25104106935089	-1.59954281966913	-1.06736063354940
H	-8.48587356663728	2.15203557724940	1.91557796034375
H	-10.43118211697749	1.66726569647097	0.48841495295605
H	-10.17241649647108	0.46621494998720	-1.64720885395505
H	-7.92214583138233	-0.27234815236555	-2.39210205384498
H	4.87790867389130	3.68784987725022	0.38903665819743
H	6.40663693268608	3.88605933296653	-0.48217337979528
H	4.92950648455569	3.48159505344217	-1.37225935587822
H	8.03883290561241	-1.83285696717801	0.19747233911738
H	10.25879377664744	-0.72444597552990	0.10346063281753
H	10.40455541587745	1.71934402205293	-0.17743272394352
H	8.37628006602658	3.09796405319768	-0.36616139060006
H	5.76264254319380	-1.96230167262351	1.47010472002566
H	5.38888742870591	-0.37793711383514	2.16248673484042
H	4.11343636629666	-1.32425685796046	1.38860162226291
H	4.15751706837377	-1.63033429484330	-1.19159096487081

H	5.45611907190608	-0.87494776913128	-2.12296337627989
H	5.80832506893466	-2.25622556547715	-1.07534049278982
H	3.19353608321615	2.25562555473017	-0.35738898473577
H	-0.95229761176833	-1.89088203009254	1.74667946344077
H	0.49067756258485	-3.69690658833239	-0.57253709900483
H	0.02448352553642	-3.87248617683810	2.78364704944986
H	-0.55349226894469	-4.25354452691619	1.16620994919438
H	1.18416911911502	-4.04761996852581	1.46720775124442
H	0.69262883748545	-1.66558454710042	3.49739730619882
H	2.03277460525822	-1.87418877757413	2.35650676310297
H	1.05538625361860	-0.40490082031419	2.31601963774136
H	0.78358355919197	-3.21602979970337	-2.93830689340686
H	0.64806515909813	-1.48444166869360	-2.65051508465824
H	1.99566799302362	-2.38125507448434	-1.95019801127586
H	-1.48580300147626	-3.81909847760775	-1.92998983770852
H	-1.96945717433788	-3.07484946907414	-0.39800018120309
H	-1.75790807760712	-2.08331883884547	-1.84505408652818

10

54

C	0.01845702426778	0.15375679779980	1.43332128406474
C	0.10450223847455	0.10253762401021	0.05510722053900
C	1.31579439085305	0.16303202775631	-0.61835198485893
C	2.49226174109551	0.27867097079940	0.09013864709650
C	2.43204393631186	0.33263818858870	1.47969698538469
C	1.21099921890919	0.27038018404559	2.13757012060063
N	-0.92769141485493	-0.01096681080703	-0.89344323108341
C	-0.45344675795597	-0.02065169354933	-2.14506722499429
C	1.06540776856238	0.08303287019099	-2.10360347714973
C	-1.29800233123259	-0.10136073644193	-3.24496876159503
C	-0.88634543105738	-0.13152607353191	-4.56515798082616
C	-1.74304605574717	-0.18709960041615	-5.64988231611418
C	1.73373945901359	-1.16898617857552	-2.68315853975719
C	1.55981849288842	1.35922350686824	-2.79344865957711
C	-2.32812115886765	-0.09626262575442	-0.55245181262355
H	-0.92504458253786	0.10870024174081	1.96147631294187
H	1.18248140643752	0.31402298279592	3.21921947196596
H	3.34552435340998	0.42457138741925	2.05365618460206
H	3.44932861048706	0.32864124659975	-0.41677991906160
H	2.81129399658352	-1.10782729027508	-2.52329342492049
H	1.36977576051508	-2.07114854067032	-2.18958857497617
H	1.55651230264923	-1.26926809593362	-3.75414000764486
H	2.63394243522532	1.46257077561425	-2.63209484476173
H	1.38002414296269	1.33752635802030	-3.86845517281759
H	1.06902221603900	2.24205200932170	-2.38155602444166
H	-2.36432455310136	-0.13624533064910	-3.06230465106978
H	-2.43206588482977	-0.12086996578029	0.52777918037157
H	-2.87007335692173	0.76962705401172	-0.93810405163646
H	-2.76483703109586	-1.00743170757754	-0.96499422991085
H	0.16689063231244	-0.11076702305736	-4.81048858327377

C	-1.25878922055017	-0.22309291228270	-6.95020422605828
F	-3.06319117133194	-0.20718260865372	-5.39264847107238
N	-1.84778495651264	-0.26147695144660	-8.12372644849180
H	-0.17530014482121	-0.21988950310713	-6.99028263302962
C	-0.99010655859720	-0.32933882038056	-9.32943809544180
C	-3.30779650348956	-0.24281460949339	-8.32633194490101
H	-1.68669977752343	-0.34257020383970	-10.16679879338758
C	-0.11934791629638	0.90813846292523	-9.46228131531885
C	-0.18747737033831	-1.61896068252204	-9.36283287249350
H	0.41597880219023	0.87835756769766	-10.41233468057387
H	-0.71868441190390	1.81902804275831	-9.43472719853591
H	0.62994648751673	0.96286994397537	-8.66867260627570
C	-3.72664793984004	0.99545286182225	-9.10331019163683
C	-3.77900516529179	-1.53422186348412	-8.97591388616137
H	-3.74927573008355	-0.18268344123290	-7.33659442049080
H	-4.86783380911026	-1.52403813290601	-9.04312169231362
H	-3.39170808567550	-1.65546161596255	-9.98972705491765
H	-3.48473075577295	-2.40284432873229	-8.38465950325674
H	-4.81550219559459	1.02494889471878	-9.16478510072537
H	-3.39154068290165	1.90616834765323	-8.60432434177019
H	-3.34344961852132	0.99539430512321	-10.12557020984971
H	0.35637244370385	-1.68913157534416	-10.30594772180134
H	0.54938849624271	-1.65495757921057	-8.55663031418150
H	-0.83640078429289	-2.49124615063886	-9.27545621178610

E4

82

C	2.74640117080071	-5.22609402334099	-5.68803541829310
C	1.58418019295652	-4.49951409142431	-5.91263527818037
C	1.74452821333097	-3.22350189237263	-6.41316666487073
C	2.97719067982347	-2.65925371851861	-6.68242158558205
C	4.12603980918629	-3.39100300562110	-6.46061660075174
C	3.99707218051191	-4.68189737193075	-5.95894362540276
N	0.73368048625717	-2.26682387655007	-6.71421190111220
C	1.24749057142922	-1.14283297231005	-7.12412187978879
C	2.76391672798658	-1.26334223675839	-7.19044989916747
C	0.61231768802188	0.14745171864109	-7.47962624605065
C	-0.87525133521541	0.51134917294017	-7.38736813840150
N	-1.75746380074388	-0.32386309476640	-8.22416121396143
C	-1.38680515715966	-0.34796864443577	-9.65292119978387
C	-1.06051820282168	1.00883000355027	-10.27054706398595
C	3.25071710080307	-1.12485467110806	-8.64169114491918
C	3.45669867214132	-0.22666811525089	-6.30111176026925
C	-0.66484066598312	-2.60949258301734	-6.53557072005829
C	-1.26471204767512	0.68069148550500	-5.94610113748080
F	-2.32732671164635	1.50141105673525	-5.78700018467406
C	-0.65876622542260	0.24064851178513	-4.83566714746101
C	-1.12163922592949	0.49734032143455	-3.50561413857054
C	-0.51416249721564	0.11063499032475	-2.35503523628213
C	0.81372762949979	-0.62147350782312	-2.16012743589886

C	0.86176344157187	-0.77097597716409	-0.65743984437122
C	-0.24922192120182	-0.14725091098107	-0.09899172520305
N	-1.03723956657997	0.38123220054202	-1.11766070568864
C	-0.44800364765240	-0.11544257262126	1.27044145878403
C	0.50636264233830	-0.72939400296330	2.07553055660466
C	1.61791536551975	-1.35420403589367	1.53075669405084
C	1.79930173819763	-1.37566895252822	0.14932541805869
C	0.85978940784519	-1.99214367443849	-2.83543722260964
C	1.97032239732436	0.26618135891226	-2.63035936934607
C	-2.25779010115077	1.10801817746287	-0.92083475613737
C	-3.20312160416641	-0.05132534187232	-7.95641970622997
C	-4.14070698870915	-0.26771250813453	-9.13399815171510
C	-3.69306881879464	-0.92454117699204	-6.80609336348019
C	-0.34607029389300	-1.40694018008392	-10.01035340727449
H	-1.31035573402259	0.36279547314396	1.71690999445875
H	0.37213820574653	-0.71784948682488	3.15050895403516
H	2.34522453461481	-1.82516241116811	2.17985087964219
H	2.67050868790973	-1.86271344843230	-0.27615613786599
H	1.74485771108635	-2.53792904265967	-2.50223779314101
H	-0.01943709084070	-2.58509724800929	-2.57939638777400
H	0.91993873500083	-1.91089697024308	-3.92141357350894
H	2.92526990747734	-0.23840262701775	-2.46535358007111
H	1.87253628868750	0.50641877999565	-3.69047419392502
H	1.98346682412970	1.20615580060241	-2.07739662297168
H	-2.04991210967725	1.04842032003893	-3.42760412222377
H	-2.42575578035089	1.25602920716014	0.14254261780402
H	-2.20284792414844	2.08914868215434	-1.40089734594856
H	-3.11179610460897	0.56440090228895	-1.33710515627130
H	0.22919945525777	-0.35955884990508	-4.96677617209420
H	-2.28252720473504	-0.69890065440967	-10.15492036010682
H	-1.00970043031355	0.91619221725334	-11.35710884514808
H	-1.82931537224429	1.74871230422992	-10.03716025655094
H	-0.09916768501432	1.41521626248385	-9.94388527791728
H	-3.31436515825306	0.99959748308998	-7.67209197822196
H	-4.69481362446758	-0.61648242431835	-6.50123090816208
H	-3.74588350575502	-1.96849237120055	-7.12857374924970
H	-3.05202338656192	-0.86992118283521	-5.92763342477223
H	-5.15559869444907	-0.07385964352348	-8.78238730164876
H	-3.95920319487430	0.41040444099814	-9.96896739985771
H	-4.12020839986996	-1.29791830992506	-9.49972842046624
H	-0.37258706940205	-1.57943970717748	-11.08844320011905
H	0.68051007653074	-1.13070864094516	-9.77251153349611
H	-0.57673124487053	-2.35518141259434	-9.52135699751688
H	0.96533621269397	0.36544323056274	-8.49035712171678
H	1.13549829039323	0.88625021396801	-6.85991117345072
H	-0.80278182546990	-3.63437658420450	-6.87738527462372
H	-0.92131457738813	-2.53771131366848	-5.47748930392497
H	-1.27635368703922	-1.92835391031753	-7.13105833427828
H	3.29535444339936	0.78654016726587	-6.67184622826340
H	3.11417438136949	-0.28884462865968	-5.26815019539883

H	4.53044242229101	-0.41705378035465	-6.31046832361547
H	4.32917200667558	-1.28555362791545	-8.66479774306857
H	2.78559627621095	-1.86316439815045	-9.29549789051190
H	3.05261053100069	-0.12431122702543	-9.02975914554079
H	5.10693872559274	-2.97784735848149	-6.66468497103535
H	4.88498230088390	-5.27350781859680	-5.77328080961654
H	2.67423262926743	-6.23211734252725	-5.29441356589266
H	0.61259330609686	-4.92125142244286	-5.69201195614624
H	-0.89630145154467	1.54400849936604	-7.77969492432112

E5

81

C	3.02315669122126	-4.90934789288190	-5.32413260608983
C	1.81085562932132	-4.30819161771263	-5.65389070438200
C	1.85468704983019	-3.06813755780933	-6.27100765180774
C	3.06411591474601	-2.44169246490592	-6.56141669729122
C	4.25466091329470	-3.04676153420193	-6.23148249121550
C	4.23320689813540	-4.29273760285197	-5.60165304811612
N	0.79411656771174	-2.27335486548413	-6.67373549369202
C	1.24474415336155	-1.05773848802083	-7.17116611851829
C	2.77824409131216	-1.14173543153625	-7.27137454717377
C	0.57254451318693	0.04590666625746	-7.52816497999373
C	-0.87637700123460	0.46050002180538	-7.42963964414829
N	-1.82460885629637	-0.35977661229678	-8.22627843128932
C	-1.48334250822615	-0.40532087228470	-9.65913423384576
C	-1.10551252559733	0.93742524149481	-10.28009782932212
C	3.20283691617710	-1.25528083232117	-8.73940607469557
C	3.48129535620184	0.04162014038074	-6.61843096655963
C	-0.56342229303019	-2.74031845130284	-6.60081111892560
C	-1.24366143253312	0.67693466272307	-5.98403056190965
F	-2.22777661550513	1.60277572046620	-5.82293510351156
C	-0.67616572045999	0.18966475545483	-4.87809148406199
C	-1.07330986001728	0.53806558295304	-3.54311970721746
C	-0.50486528659717	0.09269309420158	-2.40079775116642
C	0.67175484575307	-0.86444932383686	-2.21089990829718
C	0.78080740999466	-0.91797815122073	-0.70464257699968
C	-0.19755330470530	-0.10059346873513	-0.14006960065556
N	-0.94382299333584	0.48374261722428	-1.14920400930125
C	-0.31534532772168	0.03467452766585	1.23451630030949
C	0.57385637021594	-0.67585993753311	2.03573447038710
C	1.54916799801051	-1.49278295205151	1.48515597920412
C	1.65291147847990	-1.61491195830284	0.09921931924557
C	0.37552876883856	-2.25586829029956	-2.77281382488855
C	1.95697475887353	-0.29850498943330	-2.81826615837716
C	-2.03391045517159	1.38850858287050	-0.95344977357295
C	-3.24086607711857	-0.03537573419940	-7.92240401287446
C	-4.22448521258472	-0.23222144589316	-9.06716084826738
C	-3.72314678934005	-0.88330063418082	-6.74835933060902
C	-0.46367294348732	-1.48056000975662	-10.01915148146381
H	-1.06869556614510	0.66771937247833	1.68645416544602

H	0.49795408561507	-0.58374545609531	3.11309684564467
H	2.22935618697470	-2.03517409394627	2.13013227919898
H	2.41502495332965	-2.25236648887075	-0.33692996002807
H	1.18999260254969	-2.93910854117673	-2.52311909342032
H	-0.55035291644206	-2.65489599942010	-2.35523110895618
H	0.28516035564182	-2.23234548338108	-3.85762215479814
H	2.79648053908525	-0.96417425490900	-2.60502441005887
H	1.86819549102208	-0.19955525977255	-3.89949101443925
H	2.18140096688027	0.68487039346463	-2.40218061249312
H	-1.90285491147058	1.22958429077296	-3.46077565520520
H	-2.20430057101617	1.53130068102144	0.11091633656164
H	-1.82131300053118	2.36295328088480	-1.40601447109074
H	-2.95208799232686	0.99648779483217	-1.40315135336807
H	0.13623278743397	-0.50189237705211	-5.03172319461608
H	-2.39621579143250	-0.72785084461072	-10.15161195969439
H	-1.06644817643775	0.84316520150681	-11.36794930361868
H	-1.83540185045593	1.71465286021063	-10.03831487252413
H	-0.12165010099570	1.27871548081249	-9.94894557817379
H	-3.31701957840198	1.02191585247432	-7.64188738462789
H	-4.71139406150741	-0.55265885539436	-6.41895195154931
H	-3.79668619337340	-1.92992594761165	-7.05764270392496
H	-3.05005438443211	-0.83324931790588	-5.89452055914362
H	-5.22317382539982	-0.00656916438485	-8.68694847596306
H	-4.04386345677919	0.43085954918184	-9.91482701485678
H	-4.24007035933310	-1.26581396911914	-9.42423736620873
H	-0.47302946079189	-1.63105424612525	-11.10254310415488
H	0.54768399387750	-1.21379121288520	-9.72404219588558
H	-0.72236040987155	-2.42866324497735	-9.54473594726206
H	1.21391969762730	0.84518299607727	-7.88185691356624
H	-0.62137255598678	-3.75087069097424	-7.01682696182325
H	-0.92948794325055	-2.77309361339166	-5.56892403379407
H	-1.20479443555007	-2.08073286546261	-7.18418468847127
H	3.23768713925530	0.97465097300004	-7.13019213909928
H	3.19843788296859	0.14343784921455	-5.57087567967136
H	4.56457780959834	-0.09333304412660	-6.66755482962491
H	4.28371629053641	-1.40121161688048	-8.80711695521370
H	2.71578818266363	-2.10347960953210	-9.22372111894406
H	2.94052848268373	-0.34613366319953	-9.28616397987060
H	5.20108601114262	-2.56587363739753	-6.45598186744975
H	5.16265189379707	-4.77833142273085	-5.33064195158477
H	3.01525638037669	-5.87742281012080	-4.83641847037531
H	0.87033238240263	-4.79076952899791	-5.41925331294855
H	-0.90588569523469	1.48689319007633	-7.83286557725603

11

50

C	0.00288229852172	-0.16313545589889	1.44669267523832
C	0.09595631776354	-0.09784888345155	0.07041177174433
C	1.30833673954662	-0.15371910102284	-0.59829249100826
C	2.48205948737189	-0.28010005907177	0.11331089452639

C	2.41583590313045	-0.34890929648485	1.50175767525812
C	1.19238369729489	-0.29038945553708	2.15501687923311
N	-0.93318280375618	0.02862154128099	-0.88393397832061
C	-0.45483349440686	0.05523251838095	-2.13181136868091
C	1.06646962977397	-0.05651708723584	-2.08453309716479
C	-1.30125402925464	0.15881905368976	-3.22872731491425
C	-0.84111205268745	0.20118950763358	-4.53600870251332
N	-1.52712954122642	0.27305043570424	-5.65456153532291
C	1.56076568222436	-1.33083382960666	-2.77888884596634
C	1.74994508568918	1.19794453031437	-2.64113919816757
C	-2.33434853312936	0.11222706696007	-0.54722754426329
H	-0.94230896028873	-0.11998829961400	1.97206357111172
H	1.15896403102004	-0.34487091589499	3.23599350504478
H	3.32679210889386	-0.44905459176472	2.07825854368849
H	3.44136950177228	-0.32666515431725	-0.38966973366137
H	2.63089733315646	-1.44437126299839	-2.59979550421941
H	1.05691624303271	-2.21351376411981	-2.38266789561456
H	1.40383619153405	-1.30151306774427	-3.85756829701166
H	2.82240242170869	1.13414638313740	-2.45196599059123
H	1.60749551884876	1.30636455865530	-3.71674496369448
H	1.37479284358636	2.09815249722606	-2.15244332939616
H	-2.36046492773801	0.20268521503255	-3.03559172862172
H	-2.44469306573126	0.08948155834055	0.53237214722958
H	-2.76210215056878	1.04508921917173	-0.91974365123416
H	-2.88170118934595	-0.73275795155065	-0.97023793689171
H	0.22936025603943	0.17400958235483	-4.69180681133002
C	-0.78948743089260	0.31693374996911	-6.93421633664933
C	-3.00059722088726	0.30450172789887	-5.73405180534152
C	-1.07597487497699	-0.91270655972852	-7.77882024091607
C	-1.05241812966863	1.61380285501114	-7.67974431397195
H	0.26685467176610	0.29695930958059	-6.65512478967024
H	-0.44634543057956	-0.89240646543118	-8.66958872260672
H	-0.85978169159342	-1.83095409379186	-7.23031591733503
H	-2.11350876529409	-0.94550096444386	-8.11760629004738
H	-0.41418069660613	1.65580211472692	-8.56353835667574
H	-2.08577474999902	1.68738719691438	-8.02461204945148
H	-0.82954923057887	2.48220003427953	-7.05778982103172
H	-3.20823897793233	0.34718940821140	-6.80341251238669
C	-3.63553692943977	-0.97937609154601	-5.22075444401672
C	-3.58573225662775	1.57018688000272	-5.12611242643749
H	-4.70302126017276	-0.96024140669380	-5.44511780124398
H	-3.20328747361884	-1.85213492375783	-5.71197015414712
H	-3.52680198764695	-1.10889637242100	-4.14459373720680
H	-4.65533486372143	1.60405784625058	-5.33847099827085
H	-3.46210444999819	1.61969797179566	-4.04485916336479
H	-3.12702079430705	2.45965229160424	-5.55972186371422

3a

56

H	1.36877177371905	-1.80404355509754	-1.19939511688911

C	2.45471951523471	-1.72759853202540	-1.13866470922962
H	2.85991545158934	-2.73716133935655	-1.05429622265816
H	2.82027315494548	-1.29295688631053	-2.06993773249616
H	2.78256454080073	-2.49581293431298	1.51330546983322
C	2.38074029736186	-1.48950440563221	1.38467998595879
H	1.29234160825246	-1.55417060947228	1.39852738556080
H	2.69793285520985	-0.88717226023452	2.23695401109081
C	2.90288551046520	-0.89909682545112	0.07036228290632
C	4.40813842702013	-0.80483733452688	0.10509425925133
C	4.78791596258332	0.52450340382870	-0.01013017201962
C	5.36711936398797	-1.78716000579413	0.22655843434368
H	5.09109043423867	-2.83171189086039	0.31728180679118
C	6.70823632685520	-1.41436203465672	0.23130397419525
H	7.47621846455381	-2.17165015165513	0.32600357128643
C	7.07064728308672	-0.07920111233946	0.11705264491479
H	8.11854131224428	0.19399605753220	0.12418769143112
C	6.11200100179826	0.91990067050584	-0.00540153885434
H	6.41215756312869	1.95627738041924	-0.08952149712244
N	3.62894757743502	1.31195172386163	-0.12193466505692
C	3.64409609442350	2.74807266925668	-0.26262625188551
H	4.67023286090086	3.08658202299746	-0.36841126234106
H	3.20520845843401	3.22714618026332	0.61532905136431
H	3.08808155651558	3.04781823536872	-1.15252604345234
C	2.51444560184223	0.56747337673706	-0.07787305257137
C	1.25040481497045	1.13360073639049	-0.15625642812961
H	1.18953055914713	2.21309721684922	-0.23996186345506
H	0.09633144356941	-0.63911561346391	-0.04020255474269
C	0.05106134809785	0.43598422380206	-0.12974912298393
H	-1.17583877083408	2.14619681358530	-0.17305942954323
C	-1.18652950997939	1.06102616290824	-0.16701811022169
C	-2.46113704707406	0.51068471684947	-0.12232027110554
C	-3.69515073561477	1.37427761264985	0.09191775038300
C	-4.78901028535447	0.34319469859735	0.10963091002552
C	-4.23156628920692	-0.91069761852461	-0.09227436246051
N	-2.83690081603797	-0.76734585003561	-0.25433024151327
C	-1.98587874872234	-1.87430612706324	-0.60827446464177
C	-4.98838858382181	-2.06637854097800	-0.11616780589003
C	-6.15049141978226	0.47013109381442	0.28276927540296
C	-3.60914420539818	2.12713083522646	1.42252531081790
H	-2.59752507841218	-2.67050099315981	-1.02582495942979
H	-1.27134966668457	-1.55534485553828	-1.36786724177058
H	-1.45019306183143	-2.26470436976463	0.26098076490046
C	-3.86774418562003	2.35454494713372	-1.07405456111453
H	-4.53265266855358	2.68514406846004	1.58457393018195
H	-3.47505646997781	1.43939829881460	2.25863465111087
H	-2.78010452326181	2.83684605578668	1.41732325243931
H	-4.79766170025560	2.91011508972836	-0.94353826512050
H	-3.04627778965572	3.07226113561505	-1.10627070433315
H	-3.91409739403825	1.83093070068482	-2.03000790012712
C	-6.36035181594500	-1.92628335364365	0.06172998452853

C	-6.93660139839093	-0.67838648279224	0.25609666754404
H	-4.55210818570171	-3.04719982705657	-0.25497401447666
H	-6.98649863713762	-2.80976849021749	0.05106680837496
H	-8.00778090660424	-0.59909752158961	0.39272389812742
H	-6.60621426851500	1.44130039388652	0.43871479287137

DIPA

22

N	0.32397948579293	-0.23354407759003	0.04166643231072
C	0.09777680916156	-0.15364967178328	1.47755362477508
C	1.20524275566486	-0.76330715512795	2.33251396409795
C	-1.24040861080193	-0.80358418609316	1.78050130277104
H	0.97175606667879	-0.66838323246223	3.39599860302119
H	1.32132285220349	-1.82839816995570	2.10683886452563
H	2.16738196575896	-0.27615847438544	2.16104320559299
H	-2.03180442254977	-0.34513860694468	1.18558020335299
H	-1.21053757796094	-1.87204686120855	1.54082880783434
H	-1.49424531065363	-0.70969118613538	2.83832867735797
H	0.01733836192107	0.90935812570288	1.73124911625671
C	1.55933592682076	0.32121047664426	-0.48815686393733
C	1.62003398059188	0.00708251664142	-1.97214191091274
C	1.61901631557335	1.82056363184862	-0.25185218690201
H	2.44968071423487	-0.12918131763699	-0.01523488591752
H	2.54467768851788	0.38503358179889	-2.41315662842597
H	1.58306541395526	-1.07206961779256	-2.14768965943064
H	0.77230829058662	0.46345886662611	-2.48909309296074
H	2.50981681371064	2.24298811856205	-0.72174609616336
H	0.73579416681439	2.30241138955259	-0.67948285008272
H	1.66047040547157	2.06736866931199	0.81112464007075
H	0.24159590850741	-1.20130681957284	-0.25043726723434

DIPA-H

23

N	0.33564957004583	-0.22620279298991	0.04480761706589
C	0.06998580995915	-0.13037375624101	1.53012628044829
C	1.19394218770605	-0.77973242097272	2.30426310970615
C	-1.28004896894077	-0.76340575497037	1.79390066227877
H	0.95330087221884	-0.74293906574896	3.36687626789221
H	1.31015365501014	-1.83279160776520	2.03218029112277
H	2.14898445550972	-0.27139581729699	2.17043712039455
H	-2.07695416943818	-0.28998324217117	1.21486216234369
H	-1.26958842980864	-1.83419104966511	1.57184688730411
H	-1.53275991613240	-0.64986197117035	2.84801826906305
H	0.02599656570303	0.93848143966420	1.74476313124687
C	1.60117503040638	0.38028004095704	-0.51673843150971
C	1.63224458053492	0.07985943209373	-2.00047651242201
C	1.65287189681719	1.85859877281860	-0.20725753731526
H	2.41648705130982	-0.14395184078415	-0.01584560733070
H	2.56965408787567	0.44113680361003	-2.42300978614401
H	1.57465666475484	-0.99235523001086	-2.20447596477332

H	0.81770662800471	0.58438289006104	-2.52776144603935
H	2.54586789129982	2.28181826838805	-0.66751283300881
H	0.78973260445877	2.38429329744141	-0.62581424117412
H	1.71027999685563	2.06575768081530	0.86152248607932
H	0.31942432121247	-1.21606369654526	-0.21166097171342
H	-0.45646338536299	0.19647362048267	-0.44480995351502

4A

29

C	0.00158146232220	-0.00042662171132	0.06250189468984
C	-0.11153281131522	-0.00048369526242	1.56136841470488
C	1.33640308265110	-0.00016533434893	1.96384761144382
C	2.12173091228374	-0.00001725159651	0.82107242776010
N	1.24804465636623	-0.00014616679268	-0.30593728298265
C	1.93900219679231	-0.00017004853576	3.20482983794742
C	3.32850030992145	0.00001088646210	3.26212510387194
C	4.09586162785955	0.00017229799478	2.10279731902161
C	3.50144163392832	0.00016178740518	0.84738031048533
C	-0.84408171347557	1.26299216710026	2.03658462024967
C	-0.84335503728185	-1.26419010719807	2.03698740196149
C	1.72367086333799	0.00059863096991	-1.68312889649628
C	-1.13862096161689	0.00021140666705	-0.86296664689441
H	4.11038329417458	0.00027238622265	-0.04707138917824
H	5.17596982163271	0.00029638507946	2.17604723691921
H	3.82302491696065	0.00001629392646	4.22550016763777
H	1.35236214320146	-0.00030889002903	4.11595067834072
H	-0.83875872226975	1.28489604131720	3.12662971254840
H	-0.35519052386200	2.17015724706212	1.67776042253637
H	-1.88343567827780	1.26039235883956	1.70479187679365
H	-0.83731924894952	-1.28609858962650	3.12702710450753
H	-1.88294468524509	-1.26194339465455	1.70594816642022
H	-0.35441709734324	-2.17120202069262	1.67784985828194
H	2.80841004870157	0.00111409446171	-1.68116264466479
H	1.37127239625453	-0.89173500313855	-2.19863085218403
H	1.37036190180730	0.89296429249536	-2.19793580604758
H	-0.84610773521064	-0.00706346465502	-1.90981262314281
H	-1.76941426152371	-0.87106736531112	-0.66392877151685
H	-1.75979679182442	0.88076167754928	-0.67399025301435

4B

28

C	-0.04579301551425	-0.00000107567254	0.02528382265143
C	-0.12174682824123	-0.00000135764435	1.40914501429696
C	1.07108051574626	0.00000018149382	2.12713065793823
C	2.30033005577336	0.00000245406408	1.48699660808660
C	2.35724060922718	0.00000277065925	0.09279533283935
C	1.18696319272929	0.00000131993517	-0.63022016036846
H	-1.07086722297059	-0.00000312736100	1.93067758810455
H	1.03126242503277	-0.00000020450820	3.21039561044014
H	3.21452390359448	0.00000371245925	2.06757187509824

H	3.31733223019013	0.00000457634482	-0.41293882805196
C	0.95701767142022	0.00000037048099	-2.12307771092954
N	-1.06585465017594	-0.00000241309914	-0.91079454800955
C	-0.56783832224143	0.00000116084536	-2.20071379826383
C	1.53555985687693	-1.25459684797017	-2.77615779659733
H	2.62212030984983	-1.27492860745686	-2.66140221489485
H	1.12529428802132	-2.15660663219272	-2.31953416610562
H	1.30113320895176	-1.27317260242086	-3.84272957227274
C	1.53556313643570	1.25459406712709	-2.77616094973739
H	2.62212369501625	1.27492277700001	-2.66140592333879
H	1.30113644482795	1.27316797871173	-3.84273276670417
H	1.12530045950270	2.15660620804980	-2.31953936826446
C	-1.30792073935158	0.00000421720645	-3.31324860403740
H	-0.83110802156066	0.00000714036751	-4.28328143683227
H	-2.38902322199168	0.00000404571990	-3.28978482353090
C	-2.46642741690065	-0.00000244020779	-0.62805755271688
H	-2.62634037490084	-0.00000693681844	0.44780101966217
H	-2.95029960507631	0.88694847577631	-1.05139496767616
H	-2.95030058427098	-0.88694921088944	-1.05140234078531

1g

65

C	7.68616540959909	-1.99485978325629	0.05320661090373
C	6.77628343055934	-0.96035287614025	0.01914979234402
C	7.22389246686637	0.35332852511900	0.00213503006894
C	8.56693846392192	0.68099127442193	0.01950707587645
C	9.47453903453528	-0.37052619678139	0.05371118672478
C	9.04445385274467	-1.69070148630233	0.07021402012079
N	6.10722713825154	1.20361688627181	-0.03291140488553
C	4.95637802144327	0.51347898378349	-0.03505665100928
C	5.26840997397412	-0.97681454107771	-0.00503303524794
C	3.72190965568457	1.14802604176491	-0.05985465955115
C	2.49420183481530	0.50496251240573	-0.06105261403084
C	1.27372977696942	1.15842703793827	-0.07892414957017
C	0.04246252361876	0.50036137258665	-0.06802973163816
C	-1.19544819159079	1.14577701198172	-0.07766993932317
H	-1.18530349534909	2.23110048958727	-0.10067321622273
C	4.72571302216390	-1.64586662659169	1.26385054988112
C	4.76655079780332	-1.68494827809004	-1.26988215314821
C	6.19279392091002	2.64368193847630	-0.05885148762503
C	-2.40871514752718	0.47917109506300	-0.05293079170420
C	-3.64359608255436	1.10832034648487	-0.05480366682059
C	-4.87075377299715	0.46018376584032	-0.01959014117626
C	-5.16545976857347	-1.03339227610664	0.01910777554000
C	-6.67343727096226	-1.03436031675668	0.04185759863634
C	-7.13629776267753	0.27390057975142	0.01679796795038
N	-6.02946497228140	1.13707727426083	-0.01531632172179
C	-8.48316676558999	0.58597498963171	0.02487371926032
C	-9.37852872592321	-0.47588503798671	0.06299082049685
C	-8.93305390870025	-1.79077539033616	0.09112987977259

C	-7.57126950275583	-2.07921077045596	0.08005312274200
C	-4.65523845539508	-1.74423830124871	-1.24077253908930
C	-4.61539895412757	-1.68742822376087	1.29264500313076
C	-6.13189306387865	2.57601248513455	-0.04180248955041
H	2.46856319402721	-0.57860891817817	-0.04442960194479
H	1.25207698806385	2.24368017166159	-0.09761993449045
C	0.05006112731000	-0.93210871922402	-0.04079745703382
H	-2.37098994132239	-0.60387819917097	-0.02774845188155
H	-3.64904139900302	2.19240068412726	-0.08479832110508
H	-7.17554504330352	2.86234440362968	0.04427989114991
H	-5.58259668714015	3.01095387819943	0.79498937470630
H	-5.73514750512117	2.97337180158632	-0.97854430104804
H	-5.00329055477430	-2.77823921670706	-1.23346126240185
H	-5.03700385877113	-1.26490488566333	-2.14335023322886
H	-3.56648963747908	-1.75704490719298	-1.28911648776386
H	-4.96316032736730	-1.16340156029393	2.18409245428365
H	-4.96875553059501	-2.71830687671027	1.34652431110202
H	-3.52578760917564	-1.70556786985503	1.30385798417486
H	-8.84562567374648	1.60542603626538	0.00086785810928
H	-10.44165149361732	-0.26981226674770	0.07046173610285
H	-9.65273049273428	-2.59920931656794	0.12099932581247
H	-7.23113264429574	-3.10838517783170	0.10103468819365
H	5.73424872294689	3.07171562917747	0.83501983928411
H	7.23673488115514	2.93967585220925	-0.09024901703690
H	5.69198436617255	3.03884222057287	-0.94460706654669
H	7.35801135767500	-3.02804831862143	0.06681473561797
H	9.77343425812162	-2.49085804015845	0.09701275119460
H	10.53509929544043	-0.15199027803286	0.06810489518945
H	8.91769997637549	1.70475279399814	0.00865320754549
H	5.09111416362653	-2.67286110666567	1.31071875854429
H	5.06715574364245	-1.12397795352849	2.15897160851016
H	3.63639900924806	-1.67704370010288	1.27478967965132
H	3.67798892320180	-1.70851158008683	-1.31884893395330
H	5.14382161786456	-1.19583254904382	-2.16910861180831
H	5.12529020560578	-2.71531648796038	-1.26897220705265
H	3.71495010066137	2.23240869180184	-0.07528301775306
N	0.05576898433069	-2.08357974049768	-0.01890135525743

TS-D1

93

C	7.90942491982475	-1.31272594728060	-3.16978329361116
C	7.11923147134201	-1.52340745602066	-2.06162961956246
C	7.62043772918834	-2.22091546372849	-0.96972016713686
C	8.90867342429034	-2.72417936602049	-0.95153182148507
C	9.69750108691440	-2.50459018119666	-2.07593620104091
C	9.21064394747010	-1.80958557355995	-3.17338410918733
C	5.69560069976777	-1.10573503117744	-1.78177059894315
C	5.48574153781764	-1.67341433551638	-0.37869012444337
N	6.62885290679652	-2.29348041176143	0.01294435825833
C	4.37221247980297	-1.61783600759083	0.41598286912167

C	3.15640885135634	-0.99253342264580	0.06685110221950
C	2.03494504995873	-0.98362212528138	0.82881751248653
C	0.78968788132473	-0.33188808415806	0.44799788612821
C	-0.43678213926305	-0.90093760334484	0.98768221441898
H	-0.32449208618012	-1.47474116672947	1.90257630158972
C	6.78703268958012	-2.94663542951623	1.28278760763016
C	4.74760409975669	-1.74573664851728	-2.80159090601221
C	5.58592887381477	0.42224932774398	-1.80360489382129
C	-1.66171290748153	-0.72357623691383	0.43930175193135
C	-2.87780319077326	-1.20511904945051	0.97727597722999
C	-4.11222226485971	-1.03781649021522	0.40894638922500
C	-4.47699881160502	-0.38329411696592	-0.92327567341158
C	-5.97860027551109	-0.54186276786848	-0.93119594385150
C	-6.38027607364440	-1.20888383568575	0.21938219365085
N	-5.25030240663491	-1.49784637427339	0.99083755223082
C	-7.70923782837765	-1.49453847922589	0.47433424697631
C	-8.64455340884550	-1.08405786466428	-0.47007382062968
C	-8.25879901730240	-0.41589894369873	-1.62303539465529
C	-6.91386492409778	-0.14072308854175	-1.85941687123280
C	-4.10729728881169	1.10069501954146	-0.97924736766025
C	-3.85682511966557	-1.14999691157195	-2.09749698664035
C	-5.27786271275400	-2.18426243537468	2.25148326739049
C	0.98915404725018	1.45069343796620	1.24493632492568
C	-0.19970921337699	2.20547206524046	1.24639620938515
N	-1.22416062158130	1.98745292940434	2.07074482741988
C	-2.32104271004725	2.81165004768775	1.76254555269433
C	-1.97508844295885	3.63676912706521	0.70330317711351
C	-0.54202602435746	3.36779265364546	0.32879767406003
C	-2.90254701572188	4.52554755422315	0.20310079032761
C	-4.16644048805040	4.56837198925934	0.78744811344293
C	-4.48960622021848	3.73874757226440	1.85362515874353
C	-3.56376494005729	2.83562549365041	2.36466285200357
C	-1.26153760089850	1.13067511450858	3.23078767224360
C	0.34423206031005	4.55322319426493	0.76055370738133
C	-0.35426469937980	3.10296323505829	-1.15893758893696
H	3.10115676263314	-0.49602128699711	-0.89587839935398
H	2.02972911962779	-1.47192952567510	1.79918455844651
H	-1.70926519011160	-0.16076920609013	-0.48664764530519
H	-2.81679748303897	-1.76921722629487	1.90262386079110
H	-6.30411636286838	-2.43044263883930	2.50899716172280
H	-4.70081762702742	-3.11174689673529	2.20384295845704
H	-4.86340512752943	-1.55390166156840	3.04400060159646
H	-4.50594427265562	1.53748925759234	-1.89663757262866
H	-4.52666503012219	1.64575171646400	-0.13339208964975
H	-3.02851444895134	1.25062460266897	-0.98297099129842
H	-4.12358464928351	-2.20697726675671	-2.05655152809542
H	-4.23230652942975	-0.74157892785749	-3.03756375124213
H	-2.76913861010670	-1.07068884800213	-2.10255501377360
H	-8.02935906431720	-2.01768663605962	1.36634279830260
H	-9.69319581262513	-1.29451498700370	-0.29846448930598

H	-9.00687047238507	-0.10938577448422	-2.34331565839589
H	-6.61401071610466	0.37853566958381	-2.76308238187403
H	6.08071074415846	-3.77514165612173	1.38450388257421
H	7.79593105342790	-3.34107083447500	1.36384242300230
H	6.62396342899544	-2.24173473621476	2.10231342462650
H	7.52997434823354	-0.77076350927034	-4.02910196499954
H	9.84533310191675	-1.65401037979947	-4.03670551960744
H	10.71092211902887	-2.88689988844815	-2.09055394697106
H	9.30584260639316	-3.27060474290508	-0.10573200295326
H	5.01188368093677	-1.40682297156742	-3.80504593386135
H	4.83040526986812	-2.83328017353343	-2.77863837239419
H	3.70712942187821	-1.47650997257783	-2.61825566309487
H	4.56621031700310	0.75928503403027	-1.61539726257617
H	6.24328221486558	0.87101939389119	-1.05737705712536
H	5.88517627347711	0.79278955440828	-2.78579467192852
H	4.41390450346186	-2.10341263850212	1.38508180596268
H	1.32743042272666	1.08201700628255	2.20558252019634
H	1.79613804655301	1.86688536694141	0.65278192744833
H	-1.47813885005405	1.73639081934141	4.11374644024434
H	-2.04391889758080	0.37919351733278	3.10662356363829
H	-0.30809389409884	0.63114029827586	3.36141092830541
H	0.05338683972763	5.44053613417810	0.19596943464685
H	0.23182349658740	4.77188511781370	1.82377159152082
H	1.39430504775626	4.34042444489263	0.55337295461608
H	-0.62000496379465	4.00506081453173	-1.71283464969277
H	0.68122867089069	2.85897492466768	-1.39451131165075
H	-0.98183940100509	2.28798503896763	-1.51453459377687
H	-2.66154279741493	5.18072233138594	-0.62600470060218
H	-4.90824559307312	5.25931513828310	0.40657564400834
H	-5.47836035716863	3.79056983246883	2.29240104588198
H	-3.81736916589714	2.18299998660879	3.19003596785607
C	0.72301479595700	-0.01557732993393	-0.96076541248458
N	0.68573370642910	0.21052980207476	-2.08810281716983

D1

93

C	-6.85357502000979	-0.52224396426139	-1.61200691539243
C	-5.89110821756177	-0.96788317135603	-0.73363502023655
C	-6.19993079515541	-1.94253079046756	0.20776319110728
C	-7.46684719966728	-2.49031781823953	0.29983965792819
C	-8.43120191647346	-2.03187958908836	-0.59248730110250
C	-8.13615817050277	-1.06146525238872	-1.53831299168073
N	-5.05965323261514	-2.22068450487957	0.96364328088185
C	-3.99989525644130	-1.45180347971974	0.58023688059314
C	-4.43968940852702	-0.57256000004819	-0.59196654551524
C	-2.78111740623230	-1.51990613856127	1.18685722816559
C	-1.63350886153763	-0.76770345572588	0.79944376891991
C	-0.42967614084840	-0.80874518652094	1.39281917351994
C	0.73843192242510	0.04428281771234	0.98822820210319
C	2.03982840226780	-0.66516909148764	1.23258536923795

C	3.06058363245072	-0.72922698958978	0.36049924137259
C	4.29113241405969	-1.40928817359330	0.58144110749581
C	5.31218611800629	-1.49316045872053	-0.31506740798353
C	5.41749989456356	-0.89169524389377	-1.71788883876118
C	6.79649583002407	-1.34723206652929	-2.13552790638477
C	7.36518308720717	-2.09976377789367	-1.11450530120142
N	6.46452593796740	-2.17784771707046	-0.05259580534864
C	8.63268942748333	-2.64265869271417	-1.23012905243867
C	9.32755921277545	-2.40910158955400	-2.41257210579915
C	8.77198994058409	-1.66090268887661	-3.43931046082867
C	7.49388494997824	-1.12319618273436	-3.30143267767414
C	4.36445890750573	-1.47110897279156	-2.66770054721852
C	5.35965282290000	0.63828664404298	-1.69353233909430
C	6.70549998922038	-2.88241433926671	1.17299421994983
C	-4.33500540917160	0.92000155100330	-0.26933136727015
C	-3.67545291799482	-0.91782372107430	-1.87479613748022
C	-4.99152635773000	-3.19495390877152	2.01376931224070
C	0.78684547853826	1.40959158304461	1.85123193920462
C	-0.43264339413307	2.22729072561046	1.86611027778753
C	-0.63375466640957	3.52640026351590	1.11738253740484
C	-2.06071989419175	3.85021053228337	1.45657437081797
C	-2.52542391209427	2.90931058528658	2.35872495587510
N	-1.49042763561424	1.96898998360375	2.59284381677509
C	-2.89835158018458	4.86631365183401	1.04482156689077
C	-4.19003381098609	4.90580070032749	1.56052815783467
C	-4.63180624652403	3.95304352640386	2.47203804021391
C	-3.79752140108066	2.92553901794957	2.89322056700564
C	0.31313116214812	4.55728014033455	1.77293898176760
C	-0.37300117441162	3.46908568188854	-0.38401418602785
C	-1.66961448387677	0.94014999545956	3.59676858209499
C	0.62504377339112	0.38678889783997	-0.42677185279035
N	0.53603726748615	0.64072639813573	-1.54441202642031
H	-0.24252289510784	-1.47639187716696	2.22885198264467
H	2.92641252112939	-0.24005854775131	-0.59802697198550
H	2.11033529902722	-1.14617512887657	2.20492038262253
H	-1.74220667161692	-0.10270845237545	-0.05090418696841
H	-2.65260252753508	-2.22537669347134	2.00179999642260
H	-5.92628019000458	-3.74725356618740	2.05947475046073
H	-4.18150675483992	-3.90480114475484	1.82617501450688
H	-4.82131494581523	-2.71939488333254	2.98526655901026
H	-4.78346547520789	1.50100786444539	-1.07750861845843
H	-4.86651311603375	1.15960352903842	0.65272915606566
H	-3.29853528995054	1.24186496440673	-0.16300756226540
H	-3.74943196631938	-1.98388330173821	-2.09353388300705
H	-4.10785851367446	-0.37030889732379	-2.71462517180289
H	-2.61901227212141	-0.65561241777014	-1.80919619253075
H	-7.71754901019535	-3.24831071110937	1.03102544209788
H	-9.43118428580711	-2.44577789284735	-0.54393025298109
H	-8.90443750160373	-0.72412683588113	-2.22258826185242
H	-6.62141401158804	0.23436850315614	-2.35372215417502

H	5.93772768787664	-3.64347073694987	1.33978565306624
H	7.67240829256186	-3.37572222976041	1.12380300481798
H	6.70857327470386	-2.19642902348014	2.02560756125363
H	7.06052116726593	-0.53778414871933	-4.10528620482758
H	9.33352862578941	-1.49386105306942	-4.34989640734229
H	10.32209422059744	-2.82292467733000	-2.52840878576583
H	9.08478370031072	-3.22834670478621	-0.43989370645827
H	4.56232458878637	-1.12480391137686	-3.68413493825087
H	4.40221585661286	-2.56137031703312	-2.66860846091990
H	3.35401537160567	-1.16221257022882	-2.39800691384909
H	4.37105125283341	1.00429344062535	-1.41412792799514
H	6.09095151194753	1.04361554142732	-0.99259822475180
H	5.59203438111950	1.02620130896466	-2.68723995917308
H	4.40915838914985	-1.90719066887198	1.53794041608710
H	1.04654712179700	1.08535522858706	2.86026735075996
H	1.61792561234213	2.00158174416484	1.46943041552812
H	-2.01430006036415	1.41905135954533	4.51438066851447
H	-2.41321272782267	0.22121740548935	3.24707529429673
H	-0.73439033796446	0.42280763283137	3.77626353790173
H	0.11890596270350	5.53540306032953	1.33151149808076
H	0.14954914337332	4.62959086640204	2.84952168917563
H	1.35619233042076	4.29821506894674	1.58584098055102
H	-0.55508817514576	4.46066838062029	-0.80134894290838
H	0.65898816989748	3.19741696798584	-0.60152183509260
H	-1.02551470383956	2.75827854536663	-0.88768052134075
H	-2.56855735203676	5.61595480365321	0.33524248688491
H	-4.86517876754128	5.69226200004971	1.24663918710099
H	-5.64245988950206	4.00777180095367	2.85664265311313
H	-4.14410170122120	2.17490164474528	3.59163676319953

12

32

C	-0.50971226655438	-0.00009672030013	-2.24557681890734
C	1.01557809193553	-0.00009581026282	-2.13167372650777
C	1.20854417652273	0.00000063467982	-0.63181524151009
C	-0.03636732181719	-0.00003004296784	-0.00469087151033
N	-1.03322426777778	-0.00009527657981	-0.96547364817148
C	2.36310204065465	0.00007446935018	0.11646033251328
C	2.27447370551832	0.00012508412195	1.50872556828334
C	1.03126093920444	0.00010087693946	2.12158376504464
C	-0.14488465672363	0.00002003981442	1.37722965581149
C	1.63053069295175	-1.26277438084964	-2.74058430490211
C	1.63049485167426	1.26249874544238	-2.74077878693800
C	-2.43782077115170	-0.00011262787148	-0.69549469387831
C	-1.28508737781723	-0.00002454667176	-3.35111625261763
H	-1.10493195836735	0.00000554348460	1.87799530751283
H	0.96736271456305	0.00014186858392	3.20362719899925
H	3.17537961056777	0.00018477346489	2.10966999658184
H	3.33454540230260	0.00008516048144	-0.36689758327590
H	2.70648643829685	-1.27853867118160	-2.55128055317847

H	1.19357658117647	-2.15751634392396	-2.29450472380932
H	1.47190248903326	-1.30813078326586	-3.81821499124493
H	2.70645356108447	1.27830983658981	-2.55149918373112
H	1.47183744585441	1.30768156047433	-3.81842021691023
H	1.19353168346599	2.15730076292988	-2.29482718672297
C	-0.83794704507955	0.00018295326449	-4.71858627211617
H	-2.36217424515859	-0.00004641522383	-3.21377587131153
H	-2.60308587518945	-0.00010819106210	0.37918719762231
H	-2.91905532665455	0.88719151956606	-1.12009996000671
H	-2.91902698668604	-0.88743864682954	-1.12008695397892
C	-1.65093691410282	0.00024718059206	-5.78037711832658
H	0.23087829571538	0.00031807176092	-4.90260573851367
H	-1.25973903941256	0.00040635061817	-6.78959428174888
H	-2.73031566802914	0.00013302483164	-5.66713104255045

13

32

C	-0.14377574033563	-0.00000156877227	1.38089462957467
C	-0.03718468528596	-0.00000399946992	-0.00120474536341
C	1.20731381534630	-0.00000034167566	-0.62950176700921
C	2.36254966446047	0.00000593447147	0.11758570845670
C	2.27575462521470	0.00000918838409	1.50986561920850
C	1.03306534395358	0.00000484542864	2.12397561306669
N	-1.03525843222518	-0.00000572630244	-0.95999440745817
C	-0.51068600643888	-0.00001279662172	-2.24116363372542
C	1.01418167217106	-0.00001281861754	-2.12925368243928
C	-1.28370643274970	-0.00000295500583	-3.34594275451360
C	-0.82595281967991	0.00003553891404	-4.70967044601928
C	-1.64786028819779	0.00002692066726	-5.75418360372998
C	1.62901502117428	-1.26196016360809	-2.73970682175456
C	1.62899854789421	1.26192864848156	-2.73973295208163
C	-2.43909957644582	-0.00000779265959	-0.69150039203405
H	-1.10325771436992	-0.00000511434796	1.88279190258886
H	0.97012358117210	0.00000614829072	3.20603356990742
H	3.17748265010275	0.00001221531691	2.10945855491683
H	3.33364275936956	0.00000532600935	-0.36649069471751
H	2.70489191965225	-1.27838632933299	-2.55046502430599
H	1.19208085510762	-2.15776645226162	-2.29587508018476
H	1.47288237873080	-1.30653797862922	-3.81786118600623
H	2.70488092293470	1.27835576008328	-2.55052285811651
H	1.47283065968094	1.30649547736104	-3.81788483042718
H	1.19207756598675	2.15773872224872	-2.29589532187288
H	-2.36056350330601	-0.00001120548351	-3.20999681286178
H	-2.60635092036802	0.00002462227445	0.38288027649295
H	-2.92080916993585	0.88751229144952	-1.11604029285034
H	-2.92079996557513	-0.88755886095170	-1.11598721272623
H	0.23472584172091	0.00007291753317	-4.92866500609609
F	-1.17784094580788	0.00004971514432	-7.01096781705847
H	-2.73114662395130	-0.00001016831842	-5.71598453085995

References

1. Grycova, L.; Dommisse, R.; Pieters, L.; Marek, R., NMR determination of pKa values of indoloquinoline alkaloids. *Magn. Reson. Chem.* **2009,** *47*, 977-981.
2. Stackova, L.; Muchova, E.; Russo, M.; Slavicek, P.; Stacko, P.; Klan, P., Deciphering the structure–property relations in substituted heptamethine cyanines. *J. Org. Chem.* **2020,** *85*, 9776-9790.
3. Stackova, L.; Stacko, P.; Klan, P., Approach to a substituted heptamethine cyanine chain by the ring opening of zincke salts. *J. Am. Chem. Soc.* **2019,** *141*, 7155-7162.
4. Owens, E. A.; Hyun, H.; Tawney, J. G.; Choi, H. S.; Henary, M., Correlating molecular character of NIR imaging agents with tissue-specific uptake. *J. Med. Chem.* **2015,** *58*, 4348-4356.
5. Peng, Z.-H.; Qun, L.; Zhou, X.-F.; Carroll, S.; Geise, H. J.; Peng, B.-X.; Dommisse, R.; Carleer, R., Synthesis and physical performance of indole and benzimidazole cyanine dyes. *J. Mater. Chem.* **1996,** *6*, 559-565.
6. Mahapatra, A. K.; Maiti, K.; Maji, R.; Manna, S. K.; Mondal, S.; Ali, S. S.; Manna, S., Ratiometric fluorescent and chromogenic chemodosimeter for cyanide detection in water and its application in bioimaging. *RSC Adv.* **2015,** *5*, 24274-24280.
7. Halbritter, T.; Kaiser, C.; Wachtveitl, J.; Heckel, A., Pyridine–Spiropyran Derivative as a Persistent, Reversible Photoacid in Water. *J. Org. Chem.* **2017,** *82*, 8040-8047.
8. Zhang, D.; Su, J.; Ma, X.; Tian, H., An efficient multiple-mode molecular logic system for pH, solvent polarity, and Hg^{2+} ions. *Tetrahedron* **2008,** *64*, 8515-8521.
9. 10.5281/zenodo.7887568.
10. Virtanen, P.; Gommers, R.; Oliphant, T. E.; Haberland, M.; Reddy, T.; Cournapeau, D.; Burovski, E.; Peterson, P.; Weckesser, W.; Bright, J., SciPy 1.0: fundamental algorithms for scientific computing in Python. *Nat. Methods* **2020,** *17*, 261-272.
11. Adamo, C.; Barone, V., Toward reliable density functional methods without adjustable parameters: The PBE0 model. *J. Chem. Phys.* **1999,** *110*, 6158-6170.
12. Weigend, F.; Ahlrichs, R., Balanced basis sets of split valence, triple zeta valence and quadruple zeta valence quality for H to Rn: Design and assessment of accuracy. *Phys. Chem. Chem. Phys.* **2005,** *7*, 3297-3305.
13. Grimme, S.; Ehrlich, S.; Goerigk, L., Effect of the damping function in dispersion corrected density functional theory. *J. Comput. Chem.* **2011,** *32*, 1456-1465.
14. Neese, F., Software update: The ORCA program system—Version 5.0. *Wiley Interdiscip. Rev. Comput. Mol. Sci.* **2022,** *12*, e1606.
15. Grimme, S., Supramolecular binding thermodynamics by dispersion-corrected density functional theory. *Chem. Eur. J.* **2012,** *18*, 9955-9964.
